# Supplementary material for: Predictive validity of A-level grades and teacher-predicted grades in UK medical school applicants: a retrospective analysis of administrative data in a time of COVID-19
Source: BMJ Open. 2021 Dec 16;11(12):e047354. doi: 10.1136/bmjopen-2020-047354 (PMC8678544; doi:10.1136/bmjopen-2020-047354)
Supplement: Supplementary data [file bmjopen-2020-047354supp001.pdf]

The predictive validity of A-level grades and teacher-predicted grades in UK medical school applicants: A retrospective analysis of administrative data in a time of COVID

Supplementary information

Contents

1. Supplementary literature review, including a summary of events from March to November 2020. 2

2. Supplementary Methods including a table of measures 11

3. Supplementary Results including the Extended Project Qualification (EPQ) and SQA Advanced Highers. 13

4. Supplementary Tables 1, 2, 3, 4, 5 & 6 and Supplementary Figures 1, 2, 3, 4 & 5 20

5. *Appendix*: Are independent (private sector) schools more accurate in their A-level predictions?.. 32

6. Appendix: Appendix Tables 1 & 2 and Appendix Figures 1, 2, 3 & 4. 36

References 43

Note: The Supplementary Material contains extended versions of the Literature Review from the main paper, the Method section of the main paper, and the Results section of the main paper. In order to maintain the flow and continuity of the supplementary material, some of the material is duplicated from the main paper. In addition to the supplementary literature review, methods and results, there is also an appendix which looks in depth at the issue of whether independent (private sector) schools are more accurate in their A-level predictions.

1. Supplementary literature review, including a summary of events from March to November 2020.

Overview of literature on predicted, forecasted and attained A-level grades. The majority of studies reported here are also discussed in the main paper, in much more abbreviated form, but here are described more discursively.

University applications in general

Petch in 1964 [1] did what Wilmut has described as "one of the earliest and most celebrated studies of teacher estimates of examination result" [2] (p.60), describing how Petch found, "grade agreement in about 43% of cases, but the examination grade was higher than the teacher estimate in 18% of cases, but lower in 39% of cases, sometimes heavily so".

Two other early studies were by Murphy in the first of which in 1979 he compared actual and predicted grades both for A-levels and, unusually, for O-levels (the predecessor of GCSEs) [3], including two-way tables of predicted vs actual grades. Of 291 results the predicted grades were accurate in 27% of cases, over-predictions in 44% and under-prediction in 29% of cases. Teachers were also asked to provide a rank order of students, and overall these correlated 0.6 with rank order in the examination, although individual teachers showed a range of correlations from just less than zero through to more than 0.9. Murphy's 1981 study drew on application forms submitted to UCCA (now UCAS) by 15,109 candidates, of which "a large number included teachers' pre-examination estimates of A-level grades" (with predicted grades being A, A/B, B, B/C, C etc). Results were broken down by exam board and also by subject. The overall correlation of predicted and actual grades was 0.66, with Physics, Chemistry and French showing the highest correlations. The study also looked at A-level – O-level correlations [4]. Although described as predicted grades, these data are actually best described as being *forecasted grades*.

More recent studies have mostly been concerned with the relationship of attained A-level grades and the predicted A-level grades entered on UCAS application forms by teachers. UCAS changed the way it collected such data in 2009, so that for UK-domiciled applicants subject-level predicted grades were available, rather than as earlier when predicted grades were only available as total point scores [5]. For various reasons, not all A-levels have predicted grades. Most analyses are for candidates across all ability levels. Note that A* grades were only introduced in 2010.

In a study of 2009 applicants [5], overall *accuracy* at the subject level for A-levels for 219,744 A-levels was 52%, with predicted and attained grade being the same. In 42% of cases predicted grades were over-estimates, and in only 7% were they under-estimates. A grades tended to be predicted more accurately but that in part reflects that A grades cannot be under-predicted (or E grades over-predicted).

Female candidates showed a slight tendency for grades to be more accurately predicted (52.3% vs 51.1% in males). Socio-economic group showed strong relationships to accuracy, with 58% accurate predictions in the Higher Managerial group and 43% in the Routine group, but that in part reflects different actual A-level achievement (58% of Managerial candidates receiving an A grade compared to 33% of Routine candidates). The Higher Managerial group had the greatest over-prediction and the Routine group the highest under-prediction. Considering ethnicity, 53% of White applicants had accurate predictions compared with 47% of Asian ethnicity, and 39% of those of Black ethnicity. Centre (school) was related to accuracy, with 64% accuracy in Independent schools, 47% in state schools, and 40% in those in Further or Higher education. The authors note that multivariate analyses are probably needed to tease apart the relationships between the various correlates of accuracy. Other analyses looked at disability, region, and nation within the UK. Number of choices also related to accuracy, applicants making four choices being more accurate than those making five choices, but it was suggested that was because of the majority of the former being higher attainers applying to Medicine, Dentistry or Veterinary Medicine. The paper concluded that it is difficult to

separate out the various factors involved in accuracy, not least because of the ceiling and floor effects for high and low attainers [5].

Wyness [6] analysed aggregated data provided by UCAS for the applicants from 2013-15, and hence A* grades were included in the analysis. Overall only 16.1% of grades were accurately predicted, a much lower figure than the earlier study using 2009 data [5], perhaps because of the inclusion of the new A* grades. 8.54% of grades were under-predicted, while 75.4% of grades were over-predicted. As with the 2009 data, there was a clear relationship between over-prediction and attained grade, although it is noted that there are strong ceiling effects at work. As with the 2009 study, independent schools provided the most accurate predictions. Applicants from disadvantaged backgrounds showed moderate to severe over-prediction. Asian and Black applicants were also more likely to be severely over-predicted. There were no differences between male and female applicants. The report is particularly interesting as it looks at prediction in high ability students, defined as AAB or above. The difference between the most and least disadvantaged in this group is much smaller, with 44.0% overpredicted in the most disadvantaged and 47.4% in the least disadvantaged. There was some evidence that under-predicted applicants tended to show under-matching (i.e. entering less competitive universities than their actual grades might predict). Further analyse and discussion of these data are provided elsewhere [7 8].

UCAS in 2017 provided some limited data on over-prediction and under-prediction of A-levels since the introduction of A* grades, with data for 2012, 2016 and 2017 [9]. Overall 19.5%, 16.3% and 16.0% of predictions were accurate, with over-prediction in 68.4%, 74.3% and 73.3% of cases, and under-prediction in 11.8%, 9.0% and 10.4% (figures from EoC17_Figure7_9_database.csv[a]). UCAS commented that, the gap between achieved and predicted A-level grades, “continues to widen” (p.23), although a comparison of 2016 and 207 results concluded that there was little effect effect due to the reforms in A-levels that took place in 2017.

Not all studies have used the *predicted grades* provided to UCAS for use by universities in selection, which for medical school applicants would have been by mid-October). Until 2015 teachers were also asked, by the end of the following May, just before A-levels were sat, to provide *forecasted grades* to Awarding Organisations, and those grades then contributed in part to decisions on grading. Forecasted grades are clearly of particular interest given proposals for calculated grades to be based on estimates of performance by schools during May. Three analyses are available, for candidates taking A-levels in 2009 [10], 2012 [11] and 2014 [12] which are before and after A* grades were introduced. A primary interest must be the comparison of these forecasted grades with the more usually studied predicted grades, described earlier for 2009 [5] and 2012 [9]. Note that the studies of forecasted grades are only for OCR (Oxford, Cambridge and Royal Society of Arts Examination Board) and hence include all A-level candidates, whereas the studies of predicted grades are for university applicants. Supplementary table 1 compares the two sets of predictions. In 2009 there is little difference between predicted and forecasted grades in accuracy, with a small diminution of over-predictions. The picture three years later, in 2012 after A* grades have been introduced, is rather different. Forecasted grades have an accuracy of 48% compared with only 20% for predicted grades. Taken overall it is difficult to reconcile the two studies which are only three years apart. Based on the 2009 data it would seem that predictions in May are no more accurate than those in October, whereas the 2012 data suggest that May predictions are much more accurate than October predictions. Having said that, even in May 2012, slightly less than a half of forecasted grades are accurate, with the same grade as in October.

It should be noted, as pointed out earlier, that the early studies by Murphy should probably be regarded as being of forecasted and not predicted grades.

[a] https://www.ucas.com/file/140426/download?token=tUxAGXtt

Grade point predictions. The analyses described so far have been at the level of A-level subjects. Students mostly take three or sometimes more A-levels, and universities usually look at the three best grades attained. Scoring grades as A*=12, A=10, B=8, C=6, D=4 and E=2 then a candidate passing three A-levels will score between 6 and 36 points for their three best grades[b]. Two studies [10] [11] have pointed out the difficulty of using totalled points. As an example, a candidate predicted AAA will be predicted 30 points but may attain grades AAA or grades A*A*D; both are equally accurate in point terms but not in grade terms. Total predicted points are important in that UCAS for a number of years only provided total predicted points for the best three A-levels, without subjects or individual grades being specified[c].

UCAS in 2016 reviewed predicted and actual A-level grade points in applicants from 2010 to 2015 [13] considering the best three grades attained. Achieved grades were one or two grades in total lower for attained than predicted grades. About a half of applicants in 2015 missed predicted total grades by two or more grades (e.g. ABB rather than AAA), a proportion that had increased by a third since 2010. Simple analyses in particular showed that missing predicted grades was associated with having *lower* predicted grades overall (as in the earlier analyses at the subject level). Multivariate analyses i.e. taking other factors into account, found missing predicted grades was associated with having *higher* predicted grades, lower GCSE attainment, taking biology, chemistry and maths, having Asian, Black, Mixed and Other ethnicity, coming from disadvantaged areas, being female, and having '[pre-A-level]unconditional offers'. Of particular interest is the relationship to GCSE grades, which have a strong relationship to A-level attainment [14] which is clearly seen in the UCAS data (see their figures 5 and 6).

What are predicted grades and how are they made?

UCAS, in its document, "Predicted grades – what you need to know"[d] says that "A predicted grade is the grade of qualification an applicant's school or college believes they're likely to achieve in positive circumstances." Later the document says predicted grades should be, "**in the best interests of applicants** – fulfilment and success at college or university is the end goal ", and "**aspirational but achievable** – stretching predicted grades are motivational for students, unattainable predicted grades are not" (all emphases in original). It also says that grades should be "determined by professional judgement" and be data-driven, including "past Level 2 and Level 3 performance, and/or internal examinations to inform your predictions".

Gill [15] has described the relatively sparse literature on how teachers estimate grades. Gill's own study followed the methodology of Child and Wilson[e] although that study is not in the public domain. Gill sent questionnaires in May to selected OCR exam centres concerning Chemistry, English Literature and Psychology, and as well as estimating grades teachers were also asked to rank within grades, the method currently being adopted by Ofqual for calculated grades[f]. Teachers also

[b] Some studies, including my own earlier ones, score A*=6, B=5, etc.. Such schemes became less popular with the advent of AS-grades, which were scored as half of an A-level, and hence it made sense to double the points available for a full A-level so that totals remained integer. With the near disappearance now of AS-levels that rationale makes less sense.
[c] Earlier studies, such my 1991 cohort, had to extract predicted grades from UCAS references, and hence they are often embedded in free text, making it difficult to match them up with specific A-level subjects.
[d] https://www.ucas.com/advisers/managing-applications/predicted-grades-what-you-need-know [Accessed 13th April 2020].
[e] Child S, Wilson F. An investigation of A level teachers' methods when estimating student grades. Cambridge: Cambridge Assessment (Unpublished document, October 2015).
[f] One teacher refused to take part because of the difficulty of ranking 260 students sitting one exam. Another teacher commented, "it was easier for smaller centres to make predictions because they know individual students better" (p.42). The paper in fact comments that, "Responses to the questionnaire were more likely to come from smaller centres. … [T]he maximum centre size amongst the sample data was only 40 for Chemistry

indicated the evidence they had used for each decision. The response rate was extremely low (2.8%). About 45% of forecasted grades were accurate (which is similar to the 48% in supplementary table 1). Detailed A-level raw marks were also available and could be correlated with rankings, giving correlations of .87, .76 and .83 for the three subjects. Those correlations are high, and certainly are higher than a meta-analytic estimate of the effect size for teachers predicting academic achievement in pupils of 0.63 (SE=.03), although there was substantial heterogeneity. They are also higher than Murphy's 1979 estimate of 0.66 for the correlation of rankings and exam marks [3]. The most important information said by teachers to be used when predicting grades was performance in mock exams, and observations of quality of work and commitment, with oral presentation also important. Amongst other topics written in, the most important was the opinion of other teachers both in the same subject and other subjects, including the head of department. Other teachers raised concerns about the lack of high stakes for mock exams which meant that students did not treat them seriously. There were also concerns about the loss of AS-levels to help in prediction.

Other examinations. We know of no studies that have looked at accuracy of prediction of Scottish Highers or Advanced Highers, of the EPQ (Extended Project Question) used in England, or of other examinations carried out in the UK.

Applications to medical school

Relatively few studies have looked at predicted grades in medical school applicants, although those studies do show a tendency to ask rather more stretching questions, perhaps because of the different interests of the researchers, and the specificity of the course and its outcomes.

Lumb and Vail pointed out that predicted grades are particularly important in the shortlisting phase of medical student selection [16]. They studied 1661 applications in 1995 to a single medical school who had estimated grades for 5053 A-levels, 52% of predictions being accurate, 41% were over-estimated and 7% under-estimated [16]. The authors presented an ROC curve (but not the area under the curve), and concluded that, "… selectors for medical schools can have some confidence in the accuracy of predictions and we should therefore continue to use them … [for] selecting the doctors of the future." (p.311).

Richardson et al, studied 721 entrants from 1991 to 1994 to a single medical school [17]. Unusually they looked at predictive validity, assessing how well predicted and actual A-level grades related to a composite outcome on the pre-clinical course. Predicted and actual A-level grades showed a minimal correlation (r=0.024), but selection would have imposed range restriction. Pre-clinical exam performance correlated 0.318 (p<.001) with attained A-level grades, but only 0.041 (NS) with predicted A-level grades. This is a rare study in which predictive validity was assessed and it implied that selection should be on actual grades rather than predicted grades, concluding in contra-distinction to Lumb and Vail that, "medical school admissions panels would be well advised to take the predicted grade with a sizeable pinch of salt" (p.296).

A third study, by one of the present team, took a different approach, using path modelling to assess the causal inter-relationships between GCSE grades, predicted A-level grades, receipt of an offer, actual A-level grades, and acceptance at medical school in an original sample size of 6901 applicants to five English medical schools [18]. A-level estimates were predicted by GCSE grades (beta=0.89), with attained A-level grades predicted by both GCSE grades (beta=0.44) and predicted A-level grades (beta=0.74). A substantive question of interest was whether the paths in the model differed between White and non-White candidates, with it being shown that none of the relationships described showed ethnic differences (although non-white candidates were significantly less likely

(compared with 423 amongst all centres), 26 for English Literature (compared with 180) and 32 for psychology (compared with 378)."

than White candidates to receive an offer based on predicted A-level grades). Although the study reported no follow-up into the medical course, this dataset is analysed further below to assess predictive validity for postgraduate examination performance.

A comment on issues in studying predicted A-level grades.

Although predicted A-level grades have been an integral part of university application and selection in the UK for four decades, obtaining data on them is less than easy. Early studies, including my own, as well as those of other medical researchers, simply resorted to having researchers transcribe grades from paper UCCA and UCAS application forms, although often that was not easy in earlier forms as the predictions were often embedded in the free text of the Referee's Statement. Until 2009 UCAS only recorded the summed score of the best three A-levels, so that study of specific subjects was not possible. Even now obtaining UCAS data on predicted grades is less than easy, and Boliver in 2013 comments, "It would have been desirable to include predicted A-level grades… . Unfortunately UCAS are unable to provide this information in microdata form because of uncertainty about its validity in the case of applicants whose application is not linked to a school or college … (personal communication from UCAS)." [19]. Similarly Wyness in 2016 in her study of three years of UCAS data comments that, "The data are aggregate (for reasons of privacy)" [6], which means of course that proper analyses at the level of individual participants are not possible. There is an irony here in that of course all universities have access to predicted grades provided by UCAS as a part of the admissions process, but subsequently obtaining those data for research is often very difficult. The data for the present study are the result of an important collaboration between UKMED and UCAS, with UCAS providing detailed information on applicants to UK medical schools for inclusion in the database, which is hosted in a safe haven to ensure strict controls on access; we are very grateful to UCAS for that collaboration without which the present study would not be possible.

A summary of events surrounding the cancellation of Alevels from March to November 2020.

The research for the present paper was carried out in April and May 2020, in parallel with the study of attitudes and responses of medical school applicants to the cancellation of A-level examinations and their replacement by 'Calculated Grades'[20 21]. The main bulk of the present paper was written between March and June 2020, with a preprint being published in June 2020[22]. Key findings from this paper and the accompanying applicant attitudes paper were presented to UK medical admissions tutors at a meeting of the MSC-SA (Medical Schools Council Selection Alliance) on 6th May 2020, and drafts of the two papers distributed. The present paper is in large part a statement of how we understood the situation in May 2020, with a few amendments for clarity.

Inevitably events, in large part political but also with many practical ramifications for medical schools and student selection, continued onwards from June 2020, particularly with the publication of Alevel results in August 2020, and the abandonment of the algorithm for calculating A-level grades, with its inevitable fallout. The following paragraphs provide a summary of events, both going forward and also, to some extent, looking back to March 2020 as a result of documents published in September and October 2020.

In July and August 2020 things moved rapidly, with dramatic changes taking place. It would have been extremely confusing and probably misleading to have tried to incorporate those changes into the text of the main paper. Instead we hope this postscript will give readers a sense of what happened, to what extent events were correctly or incorrectly predicted by us, what impact the present paper may have had, and what may be the implications for the future.

The story is best told chronologically and we mostly use reports from newspapers, and refer interested readers to a brief summary on Wikipedia[g] and a journalistic review on the BBC website[h].

The awarding of A-level grades in 2020: the story from March to November 2020

As described in our main paper, on 20th March 2020 public examinations in the UK including A-levels were cancelled. On 3rd April *Ofqual* announced that exam grades in England would be replaced with Calculated Grades. Calculated Grades were to consist of Centre Assessment Grades (also called Centre Assessed Grades or CAGs), estimated by teachers that centres (mostly schools and colleges) would submit to *Ofqual*. *Ofqual* would moderate these CAGs using an algorithm – the details of which had not yet been published but, it was stated, would be based on the prior performance of pupils within schools. The Scottish Qualification Authority (SQA), Qualifications Wales, and the Northern Irish Council for Curriculum, Examination and Assessment (CCEA) also announced that they would use a broadly similar approach to that of *Ofqual*.

Schools (centres) had to return their teacher-estimated CAGs to *Ofqual* in June 2020. On June 16th a report in The Times said that "Teachers have marked too generously in allocating GCSE and A-level grades this year, research suggests" (The Times, 16th June), the article being based on a report from FFT Education Datalab, which actually had only asked about GCSEs, and had no data on A-levels[i] . In July a Guardian article reported a statement from *Ofqual* that "a substantial number of students would receive at least one adjusted grade – usually downwards – as a result of a standardisation process" although they "sought to allay fears that certain groups of pupils, … could be disadvantaged by calculated grades. *Ofqual* said their analysis had found no evidence of widening of gaps in attainment".(*The Guardian (G),* 21st July).

SQA results in Scotland are announced a week before those in England, so the SQA results announced on August 5th 2020 gave a preview of what was to come the following week in the rest of the UK. The Scottish results were immediately controversial when it emerged that the moderation of teacher-estimated grades (CAGs) by an algorithm had resulted in a quarter of grades being adjusted downwards. The Scottish Education Secretary, John Swinney, said that without those adjustments the pass rates would be up on the previous year by 14% for Highers and 13.4% for Advanced Highers. He added that, "… these robust processes mean we have upheld standards… All exam systems rely on an essential process known as moderation to uphold standards. This ensures an A grade is the same in every part of the country, making the system fair for everyone, and across all years." *(G*, 4th August).

Teachers, students, parents and the media were unhappy with the moderation. A *Daily Telegraph* editorial entitled "Exam moderation is a gross injustice" attacked statistical modelling in general, and the SQA process in particular, which "gives poorer marks to children living in deprived areas … [without] recognition of individuals who buck the general trend" (*Daily Telegraph (DT)*, 5th Aug), and asked "Is the same fiasco about to be inflicted on A-level students in England and Wales?". By 11th August, students in Scotland were protesting on the streets, Nicola Sturgeon, the First Minister, was apologising for the exam results debacle, and the Scottish government was facing a vote of no

[g] https://en.wikipedia.org/wiki/2020_UK_GCSE_and_A-Level_grading_controversy

[h] Coughlan,S. "Coronavirus: The story of the big U-turn of the summer", https://www.bbc.co.uk/news/education-54103612

[i] https://ffteducationdatalab.org.uk/2020/06/gcse-results-2020-a-look-at-the-grades-proposed-by-schools/

McManus IC, *et al. BMJ Open* 2021; 11:e047354. doi: 10.1136/bmjopen-2020-047354

confidence (*G*, 11th Aug). Expectations in the media of problems with A-levels were also growing. On 12th August it was announced in Scotland that the teacher-estimated CAGs downgraded by the SQA algorithm during moderation "would be reinstated" (*G*, 12th Aug).

In an attempt to prevent problems with A-levels, on August 12th 2020 the English education secretary, Gavin Williamson announced "a triple lock" for A-level students, whereby students could accept their Calculated Grade results, use the results of mock exams (practice exams which students take in schools), or use the results of real exams due to take place in Autumn 2020 after the start of the university academic year (*The Times*, (T), 12th August; *T*, 13th August). Protests were immediate as mock exams vary immensely, and many schools had been encouraged to cancel mock exams as a part of the Covid lockdown in March 2020.

A-level Calculated Grades (i.e. the teacher-estimated CAGs adjusted by the algorithm during moderation) were announced on 13th August 2020, and UCAS announced which students had obtained places at their chosen university based on these Calculated Grades. An immediate problem arose: following the Scottish government's reversal, students in Scotland now had unadjusted SQA grades, which were higher on average and gave them an advantage over applicants with A-level Calculated Grades, which had been adjusted (*G,* 13th Aug). University admission processes were also becoming embroiled in confusion, and although universities had, "reassured ministers that they will 'soften' the grades they normally require" (*T*, 13th August), by the next day universities were accused of being inflexible (*G*, 14th Aug).

It soon became apparent that schools in the private, fee-paying sector, had probably benefited from the algorithm, primarily because statistical predictions were less accurate for the small class sizes more likely to be found in private schools, and in those cases the teacher-estimated CAGs had been allowed to stand unadjusted. Although the Prime Minister Boris Johnson defended the system saying, "Let's be in no doubt about it: the exam results that we've got today are robust, they're good, they're dependable for employers" (*G*, 14th August), many backbench MPs were in revolt, having been deluged with complaints from constituents (*T*, 14th August). The next day Gavin Williamson said, "No U-turn. No change" (*T*, 15th August), and although he did agree to waive fees for appeals against Calculated Grades, he insisted that the grades themselves would not change in order to avoid the grade inflation that had occurred in Scotland (*T*, 15th August).

Meanwhile the *Ofqual* algorithm, published in a document over 300 pages in length, was being dissected carefully, and when one headteacher anonymously shared their school's results, the problems became particularly apparent[j]. In the previous three years at their school, 12.5% of pupils had achieved A* and none had got a U; however the algorithm meant only 3.7% of students (equivalent to just one student) received an A* - much below the historic 12.5%. The algorithm also resulted in one student being awarded a U, despite no students at that school having received a U previously. The weekend newspapers attacked the government, "which deserved a U grade for this debacle" (Sunday Times (ST), 16th August). GCSE results, due on Aug 20th, were also on the horizon, with similar problems predicted (*T*, 15th Aug, p.14; *Observer (O)*, 16th August). Students in England demonstrated outside the Department for Education in London. *Ofqual* also announced guidance on the role of mock exams in appeals only to withdraw it a few hours later (*T*, 17th August).

[j] Hern,Alex. (2020) "Do the maths: analysis shows why England's grading system is both imprecise and unfair", *Guardian*, 15th August, 2020, p.13; the analyses are based on Thomson, Dave, "A-Level results 2020: How have grades been calculated?", 13th August 2020, https://ffteducationdatalab.org.uk/2020/08/a-level-results-2020-how-have-grades-been-calculated/.

On August 18th the government scrapped *Ofqual's* algorithm and reverted to unadjusted teacher-estimated CAGs (*G*, 18th August). *The Times*, normally a supporter of the Conservative Party, simply called its main editorial, "Another Fine Mess" (*T*, 18th August). The chairman and chief executive of *Ofqual* were criticised for having little experience of education (*T*, 19th August), and the Chief Executive eventually resigned on August 25th.

Several other problems now emerged, not the least being that universities would not know the (now unadjusted CAG) grades for several days. Once universities did receive these grades, they found that they did not have enough places to honour all the offers they had made students months earlier. This was because universities typically make more offers than they have places, knowing that a significant number of students will not meet those offers when they achieve lower exam grades than the Predicted Grades estimated by their teachers and submitted to UCAS when they apply to university. But now with the teacher-estimated CAGs replacing exam grades, and the resulting increase in the percentage of A and A* grades from 28% to 38%, many more students than expected did in fact meet their university offers (*T*, 18th August). This caused problems for some students whose Calculated Grades (the grades adjusted by the algorithm) had been too low for their first choice university and so had accepted offers from their second choice, but now they found their unadjusted teacher-estimated CAGs enabled them to meet their first choice offer they inevitably wanted to go there. Other students seemed to have been left in limbo, needing to delay entry until the next year, with a potential knock-on effect for students taking A-levels in 2021 (*T*, 19th August). Some groups of A-level students had clearly fallen through the net and were in limbo, such as who were home-schooled or private A-level students, having no teachers to estimate their grades (*G*, 21st August).

Regarding medical schools specifically, *The Guardian* reported that the Norwich Medical School at the University of East Anglia had 185 places and a possible overshoot of 50, a 27% increase, emphasising that with medical school numbers more strictly limited than other university places and costing £50,000 a year this would have clear financial implications (*G*, 19th August). A news story based in part on the current research as published in pre-print on *medRxiv,* suggested that more medical students may be liable to drop out, a co-chairman of the Medical Schools Council suggesting that "we are going to have, on average, students with lower grades than in previous years" (*DT*, 20th August). A similar concern was also raised by headteachers in relation to GCSE grades, which it had now been announced would also be based on unadjusted teacher estimates, with fears that students, "could end up on unsuitable courses post-16 which could set them up for failure" (*G*, 21st August).

The cap on university student numbers was released on 17th August, the medical school cap following on 20th August, resulting in a bulge of new undergraduates. There was also a bulge in admissions to sixth form colleges as a result of unadjusted teacher-estimated GCSE grades being higher than the expected exam grades (*G*, 21st August). There are financial implications for educational institutions but no-one would know the size of the problem until UCAS released its entry statistics for October 2020.

The controversies rumbled on into September 2020 as the House of Commons Select Committee on Education heard evidence from *Ofqual* and other bodies. *Ofqual* put out a lengthy statement on 2nd September in evidence to the Committee[k]. It set out the history according to the regulator, stating that in March its advice to the Secretary of State had been that, "the best option in terms of valid

[k] https://www.gov.uk/government/news/written-statement-from-chair-of-ofqual-to-the-education-select-committee, "Written statement from Chair of Ofqual to the Education Select Committee", 2nd Sept 2020.

qualifications would be to hold exams in a socially distanced manner"; however, "The decision to use a system of statistical standardised teacher assessments was taken by the Secretary of State and issued as a direction to *Ofqual*". In reviewing the failure of the system, the conclusion was reached that, "a 'better' algorithm would not have made the outcomes significantly more acceptable. The inherent limitations of the data and the nature of the process were what made it unacceptable…. *To try to deliver comparable qualification results in the absence of students having taken any assessments (examinations) proved to be an impossible task*" (our emphasis). Cambridge Assessment's submission to Select Committee provides a detailed timeline of collaborative efforts to inform decision making.[l] With the model running and results being calculated, from late July through August, Cambridge Assessment worked with Ofqual and DfE to understand possible unfairness in the outcomes, and to put in place adequate remedy. No doubt the post-mortem will continue for a long while.

In autumn 2020, the needs of the next year began to be considered. One group, including several university Vice-Chancellors, argued that A-levels should be cancelled once more and replaced by teacher-estimated grades (*T*, 2nd October). Others argued more radically that teacher-estimated grades should permanently replace A-level examinations. On 12th October 2020 the Secretary of State announced that A-level exams would go ahead in England in summer 2021 with some minor changes, including being three weeks later than usual, and with results announced a few days later than typical[m]. Contingency measures would be in place for possible disruption, but were yet to be described, although a leaked newspaper report suggested that they might include more formal mock exams as a back-up when following earlier leaked reports (*G*, 10th October). Perhaps the most interesting comment by Williamson was that, "Exams are the fairest way of judging a student's performance …", with its tacit acceptance that teacher-estimated grades perhaps actually were not fair to many students.

To date other UK countries have taken different approaches. On 7th October the Scottish Government announced the cancellation of National 5 exams in 2021 although Higher and Advanced Higher examinations would be taken (BBC, 7th October). A month later on 10th November the Welsh Government announced that GCSE and A-level examinations in Wales would be cancelled, with grades being based on classroom assessments instead (BBC, 10th November). On the same day Education Minister Peter Weir announced that GCSE and A-levels examinations would be taken in Northern Ireland (BBC, 10th November). In November 2020 the Secretary of State announced his intention to consider post-qualification university admissions (http://researchbriefings.files.parliament.uk/documents/CBP-8538/CBP-8538.pdf).

This postscript has been relatively brief, given the complexity of the events, and it has not attempted to summarise events occurring in 2021, with teachers now mostly but not entirely responsible for awarding Alevel grades, but under some control by Ofqual. That story is probably too long, and not yet complete, for it to be included here. The history of the events of 2020 may however help those new to the issues to navigate through the major changes that occurred. In research terms, in medical education, higher education more generally, and in secondary education, there seems little doubt that researchers will be following in detail the outcomes for the cohorts affected by the dramatic changes which resulted in a giant, unplanned, experiment, where notional grades awarded

[l] https://committees.parliament.uk/writtenevidence/11358/default/, "Written evidence submitted by Cambridge Assessment", 2nd Sept 2020.

[m] https://www.gov.uk/government/news/students-to-be-given-more-time-to-prepare-for-2021-exams, "Students to be given more time to prepare for 2021 exams", 12th October 2020.

were probably different in many cases to what students would have been awarded in normal circumstances.

2. Supplementary Methods including a table of measures

Data for the present study comes from two separate primary sources:

"P89". UKMED project UKMEDP089, "The UK Medical Applicant Cohort Study: Applications and Outcomes Study", approved Dec 7th, 2018, with Dr Katherine Woolf as principal investigator, is an ongoing analysis as a part of UKMACS (UK Medical Applicant Cohort Study). Data are primarily concerned with the process of selection. In particular in the upload of 21st Jan 2020[n] there is detailed information from UCAS on all applicants to medical schools from 2007 to 2018, including all attained Key Stage 5 (Level 3) qualifications (e.g. A-levels and SQA) as well as teacher predicted grades for individual Key stage 5 qualifications.

"P51". UKMED project UKMEDP051, "A comparison of the properties of BMAT, GAMSAT and UKCAT", approved Sept 25th, 2017, with Dr Paul Tiffin as principal investigator, is an ongoing analysis of the predictive validity of admissions tests and other selection methods such as A-levels and GCSEs in relation to undergraduate and postgraduate attainment. A major feature of the study is the inclusion of data from UCAS, although in the13th May 2019 data upload, which was used here[o], UCAS predicted grades were only available as a composite, 18-point score, for application years 2010 to 2014. A new upload of the data in late April 2020 will provide more detailed information, but that will require quite extensive coding, etc., making it similar to the qualifications data for applicants in P89. For the present data upload, predicted A-level grades are in the old UCAS format consisting of a single number from 6 to 18 (i.e. 3 Es to 3 A*s using A*=6 coding). Outcome data for the P51 dataset are more extensive, and in particular include data for end of undergraduate training, including the UKFPO EPM measures, the UKFPO SJT as well as PSA (Prescribing Safety Assessment). Some data are available for later postgraduate examinations, but numbers inevitably are small for cohorts entering medical school in 2011 onwards.

A-level grade scoring. In both P89 and P51, A-level grades are expressed numerically on a standard scale of A*=12 points, A=10, B=8, C=6, D=4 and E=2, or have been rescaled to that score.

The table below provides a detailed description of the source and coding of the measures used in the analyses:

Measure name	Description	Derivation	Source
GCSE grades	Average GCSE score from the best 9 GCSEs	The sum of the nine best grades (counting double science as two separate GCSEs)/the number of GCSES in include – i.e. 9 or fewer. This is the methodology used by the UKCAT-12 study (McManus, I.C., Dewberry, C., Nicholson, S. and Dowell, J.S. (2013) "The UKCAT-12 study: educational attainment, aptitude test performance, demographic and socio-economic contextual factors as predictors of first year outcome in a cross-sectional collaborative study of 12 UK medical schools". *BMC Medicine* 11 (1), p. 244. ISSN 1741-7015. http://bmcmedicine.biomedcentral.com/articles/10.1186/1741-7015-11-244	GCSEs supplied to UKMED by UKCAT from data obtained from UCAS. We used the GCSEs associated with the first application.

[n] OUTPUT_UCAS_QUALS_DEC_20200121_1.TXT and OUTPUT_UCAS_QUALS_DEC_20200121_2.TXT (both dated 21/1/2020), and OUTPUT_UCAS_QUALS_VER.TXT (dated 5/12/2019).
[o] UKCAT51_APP_ALL_DATA_13052019_FILE1.SAV and UKCAT51_APP_ALL_DATA_13052019_FILE2.SAV (both dated 13/5/2019).

		Further details are available in McManus IC, Dewberry C, Nicholson S, Dowell J: *The UKCAT-12 Study: Technical Report. 2012*, UKCAT Consortium: Nottingham, https://www.ucat.ac.uk/media/1185/ukcat-technicalreport-march2012-withbackgroundandsummary-sep2013v3.pdf	
Predicted A-level grades	Predicted grades are provided by an applicant's teachers on the UCAS form the autumn prior to sitting A-levels.	UCAS supplied a score from the A-level grades declared by the applicant's teachers on the application. The highest three grades are considered only, adding up the following points per grade: A* = 6, A = 5, B = 4, C = 3, D = 2, E = 1. AS levels are not included. This variable was only available for 18-year-old applicants domiciled in England, Northern Ireland and Wales. To bring this into line with attained grades the number was multiplied by 2 to give a maximum of 36.<br><br>The value is taken from the 1st UCAS application present in the UCAS extract.	UCAS supplied data
Attained A-level grades	Total score from the best 3 A-levels.	Sum of the three highest A-level grades. Assign point scores to A-Level Grades in 2-point increments (A*=12, A=10, B=8. C=6, D=4, E=2, else=0). This is the methodology used by the UKCAT-12 study (see above). By using the best three A-levels we are able to accommodate the differing numbers of A-levels taken by applicants.<br><br>All grades were from 2010 onwards, when A* grades were available at Alevel, prior to that the maximum possible mark being A.	HESA qualifications data
UKCAT total	Total score on UKCAT test	A total scale score is generated by summing the individual scale scores of Verbal Reasoning, Decision Making, Quantitative Reasoning and Abstract Reasoning.<br><br>We used the score from the last available attempt which was therefore that associated with admission.	UCAT known as UKCAT at the time these data were created.<br><br>UCAT publish technical reports with the details. See: https://www.ucat.ac.uk/about-us/technical-reports/
BMAT sections 1 and 2	Combined BMAT score	BMAT_SECTION1 measures Aptitude and Skills.<br>BMAT_SECTION2 measures Scientific Knowledge and Applications.	BMAT published details of the test. See: https://foundationprogramme.nhs.uk/faqs/educational-performance-measure-epm-faqs/
UKFPO EPM decile	Medical school performance relative to peers ranked in deciles.	Students in a graduating cohort are ranked on their medical school performance (Educational Performance Measure, EPM). Individual schools decide which assessments to include in the EPM that meet the specified criteria and are required to consult with students and publish on their website the assessments included in that score.	UKFPO. Contained in the FP table in UKMED. See: Educational Performance Measure (EPM) 2019 Framework 2019. http://www.foundationprogramme.nhs.uk/sites/default/files/2018-07/UKFP%202019%20EPM%20Framework%20Final_0.pdf
UKFPO SJT score		The Situational Judgement Test (SJT) is a final year undergraduate test that assesses individuals' reactions to a number of hypothetical role-relevant scenarios, which reflect situations candidates are likely to encounter as a doctor. It seeks to provide a reliable measurement of the following non-academic domains: Coping with pressure, Working effectively as part of a team, Effective communication, Problem solving, and Commitment to professionalism.	UKFPO. Contained in the FP table in UKMED. See ISFP Project. Situational Judgement Test https://isfp.org.uk/sjt/ 14th February 2019.
PSA score	Score relative to pass on	The British Pharmacological Society and MSC Assessment developed the Prescribing Safety Assessment (PSA) that allows all UK medical	MSC Assessment provide these data to UKMED. See:

	first attempt at PSA	students to demonstrate their competencies in relation to the safe and effective use of medicines.<br>We used the score relative to the pass mark, as the pass mark varies by diet.<br>We used the first attempt at the exam.	https://prescribingsafetyassessment.ac.uk/
MRCP (UK) Part 1	Score relative to pass on first attempt at MRCP (UK) Part 1	MRCP Part 1 is the entry-level examination accessible to doctors with a minimum of 12 months' postgraduate experience in medical employment. It covers a broad range of topics to ensure the level of knowledge is appropriate to physicians at the beginning of postgraduate training.<br>We used the score relative to the pass mark, as the pass mark varies by diet.<br>We used the first attempt at the exam.	The Royal Colleges provide data annually to the GMC for quality assurance purposes. The collection notices are published by year (see: https://www.gmc-uk.org/education/reports-and-reviews/progression-reports/downloads-resources-and-briefing-notes ; e.g. Medical Royal College & Faculty Exam Data (2015) available at: https://www.gmc-uk.org/-/media/documents/exams-data-project---data-submission-briefing-note_pdf-56793364.pdf<br><br>The data in UKMED is the variable EXAM_TOTAL_MARKS ; see https://www.ukmed.ac.uk/documents/UKMED_data_dictionary.pdf
MRCS Part A	Score relative to pass on first attempt at MRCS Part A	The Intercollegiate MRCS Part A is designed to test knowledge of both applied basic science and principles of surgery in general to a level that a surgical trainee should have. It is a five-hour MCQ exam consisting of two papers taken on the same day.<br>We used the score relative to the pass mark, as the pass mark varies by diet.<br>We used the first attempt at the exam.	Details of data source etc are the same as for the MRCP examination (see above)

Rounding and suppression criteria. All data from HESA are required to be reported using their rounding and suppression criteria (https://www.hesa.ac.uk/about/regulation/data-protection/rounding-and-suppression-anonymise-statistics) and although not all data in the current study use HESA measures we have nevertheless applied the HESA criteria to all UKMED-based tables and values reported in this study. It should be noted in particular that the presence of a zero or a zero percentage may not always mean that there are no individuals in a cell of a table. All Ns are rounded to the nearest 5 which should easily flag up that rounding has been applied, all counts ending in 0 or 5. Percentages are only reported when the number of participants is greater than 22.5.

3. Supplementary Results including the Extended Project Qualification (EPQ) and SQA Advanced Highers.

Predicted and actual grades for Key Stage 5 qualifications.

Predicted and actual grades for individual A-levels. Supplementary table 2 shows the relationship between predicted and attained A-level grades for 237,030 individual examinations from 2010 to 2018. Supplementary table 2.a shows frequencies in the various combinations, with bold values in grey boxes on the diagonal indicating accurate prediction of grades, green and blue indicating under-prediction by 1 or 2 grades, and orange and red indicating over-prediction by 1 or 2 grades. Overall 48.8% of predicted grades are accurate. Under-prediction occurs by one grade in 35.7% of cases, and by two or more grades in 9.0% of cases. Over-prediction is by one grade for 6.3% of A-levels, and 0.1% by two or more grades. It should be remembered that since the median grade for actual A-level grades is A, then over-prediction in such cases can only be by a maximum of one grade, since A* is the highest grade.

Supplementary tables 2.b and 2.c show the data of supplementary table 2.a as percentages. As has been pointed out [5] percentages within predicted grades and percentages within actual grades have different interpretations and uses. Both are presented here, but from the perspective of admissions tutors perhaps the most useful are those in supplementary table 2.b of percentages within predicted grades in relation to actual grades, as they show the likelihood that a predicted grade will actually manifest as particular actual grades. About a half of A* predictions actually gain an A grade, and over a third of predicted A grades result in a grade B or lower.

Allocating points on the basis of A*=12, A=10, B=8, C=6, D=4 and E=2, predicted grades show systematic *bias*, the mean prediction of 10.53 points being systematically higher than the mean actual grade of 9.55 points, the difference of 0.98 points being about half of an A-level grade, and can be seen in the greater numbers in red and orange cells in supplementary table 2.a (over-prediction, 45%) than in the blue and green cells (under-prediction, 6%).

Despite the bias, predicted grades overall show a reasonable *correlation* with actual grades, with a Pearson r_p of 0.624 and a Spearman correlation r_s of 0.581. Both predicted and actual grades are skewed because of censorship, values above A* not being possible. A tetrachoric or polychoric correlation fits an underlying latent normal distribution into account, accepting that row and column totals may not be equally spaced, being ordinary in nature [23]. Using the *polychor()* function in *R* the polychoric correlation, r_t is somewhat higher at 0.716 (SE 0.002), and is probably the best estimate of the true extent of correlation.

Differences between A-level subjects. A-levels in different subjects may show differences in their degrees of bias or correlation. Subjects were divided into 26 broad groups (see supplementary table 3), with the Modern Languages group including 21 languages.

Supplementary table 3 shows the mean predicted points, the mean actual points, actual minus predicted points, and the Pearson correlation of predicted and actual points. Subjects are sorted by the number of examination entries, and values are colour coded on a green-yellow-red scale, green indicating higher predicted and actual grades, a smaller difference between predicted and actual grades (i.e. less bias), and higher correlation of predicted and actual grades.

Considering the four major subjects of chemistry, biology, maths and physics, differences between actual and predicted grades are very similar (-1.15 to -0.98) indicating a bias of about 1 point (i.e. half of a grade) and very similar correlations of 0.600 to 0.635. Amongst other subjects there is inevitably greater variation in those subjects taken less frequently. Of particular interest, given that some medical schools use it for selection, is General Studies, which has the largest difference of predicted and actual grades of -1.96 points, equivalent to a whole grade. The smallest bias is for art and design subjects at -.57 points, perhaps indicating the role of an incourse portfolio in these subjects giving teachers a better sense of how students are performing. Correlations of predicted and actual grades are mostly very similar, although the lower correlations are for general studies, modern languages, geography, history, economics, music and classics, and, as mentioned, for general studies.

Total predicted and actual points, correlations between grades and reliability of measures

Reliability of actual and predicted A-levels. The reliability of total points from the three best actual and predicted A-levels was calculated by randomly sampling a pair of grades from the best three and finding the correlation. Cronbach's alpha for the three totalled grades could then be calculated from the standard formula, Alpha = 3.r/(1+2.r) where r is the mean correlation, and is equivalent to a single randomly sampled correlation between a pair of grades since any pair should give similar results. Analysis was restricted to the 66,006 candidates who had at least three paired predicted and actual grades. For actual grades r=0.615 (SE .003) giving alpha=0.827, while for predicted grades r=0.550 (SE = .004) and hence alpha =0.786. Given the standard errors, the correlation between grades is clearly substantially lower for predicted than actual grades, and the same must be true of alpha. Interpreting the difference is not entirely straightforward, since on the one hand more predicted grades are at A*, meaning that there should be fewer non-identical grades, but range restriction might also result in a lower correlation. In terms of mechanism, teachers may collaborate in producing predicted grades [15], and such non-independence would increase correlations and increase alpha. However teachers may also spend less time making judgements than do A-level examiners, and hence there should be lower correlations. On balance it seems that the most likely conclusion is that estimated grades are somewhat less reliable than actual grades, but there is clearly a need for more complex modelling of the reliability of actual and estimated grades.

Predicted and actual grades for Extended Project Qualification (EPQ). The English EPQ has become popular qualification for medical school applicants, being taken by 18616 applicants over the years 2018 to 2018, about 2100 applicants a year (perhaps 10% of all applicants). There is evidence that it has predictive validity for degree outcomes [23]. At present it is not known if it predicts outcomes in application or at medical school. Supplementary table 4 shows the relationship between actual and predicted grades. Grades are over-estimated in 33.7% of cases, under-estimated in 14.0% and accurate in 52.3% of cases, the mean score difference, the bias, being 0.805, which is a little under half a grade. Pearson's correlation is r_p=.459, Spearman's correlation is r_s=.457, whereas the polychoric correlation is somewhat higher at r_t=.569.

Predicted and actual grades for SQA Advanced Highers. SQA Advanced Highers, as with SQA Highers, are scored both as simple literals (A, B, C D) and as a more extended scoring (A1, A2, B3, B4, C5, C6, D7), although predicted grades are only in terms of literals. Supplementary tables 5.a and 5.b show, that A grades are more frequent in predicted than in attained grades. Using literals, 59.8% of predictions are accurate, 37.7% are over-estimated, and 2.6% are under-estimated, and for literal grades the bias was 0.976 points, equivalent to half a grade. Correlations of predicted grades with literal attained grades were r_p=.407 and r_s=.357, whereas with extended grades were r_p=.409 and r_s=.355. Polychoric correlations were r_t=.575 for literal grades and r_t=.587 for extended grades, again showing the similarity across the two grading schemes.

Summary. Taking all the exam types together, A-Levels, EPQ and SQA Advanced Highers, it is generally clear that predicted grades are usually about a half-grade higher than actual grades. Where grades are not accurate there are about four times as many grades over-estimated as under-estimated.

Predictive validity of predicted and attained A-level grades.

A key question throughout discussions of calculated grades is whether grades estimated by teachers are better or worse at predicting outcomes than are actual A-level grades. That question is answered not in terms of how well predicted grades relate to actual A-level grades, but by assessing how well predicted and actual grades predict subsequent outcomes during undergraduate and postgraduate training. It should also be said that it is not entirely self-evident that teachers' grades will be less good, and in the context of GCSEs rather than A-levels, Thomson said, "It is possible, in theory at least, that teacher judgements may be more reliable than exam grades, particularly in those subjects where exam reliability is lower" [24], with "more reliable" being somewhat ambiguous and perhaps

also meaning more valid as well as more reliable in the narrow statistical sense. Questions about predictive validity can be answered by the P51 dataset.

Predictive validity in P51. The P51 UKMED data includes only applicants applying for medical schools. Predicted A-level grades were available only for the UCAS application cycles of 2010 to 2014, and consisted of a single score in the range 2 to 18 points, based on the three highest predictions scored as A*=6, A=5, etc.. The modal score for 38964 applicants was 15 (equivalent to AAA; mean=15.88; SD= 1.79; Median = 16; 5th, 25th, 75th and 95th percentiles= 13, 15, 17 and 18). Some older applicants had only pre-A* A-levels, and it was also desirable to restrict the analysis to standard applicants in their first year of application, and so only those aged 18 in the UCAS year were included. For multiple reasons not all applicants had both predicted grades and attained A-level grades, and analysis was restricted to the 22954 applicants with both predicted and attained grades. Other selection measures which were included in the analysis are GCSEs (mean grade for best eight grades), as well as U(K)CAT and BMAT scores, which are based on the most recent attempt which in most of the present cases is also the first attempt. For simplicity we used the total of the four sub-scores for U(K)CAT, and for BMAT the total of the Section 1 and 2 scores. No GAMSAT scores were available for this age-group.

Outcome measures are complicated as different application cohorts enter medical school and graduate at different times, and lags within the system mean that not all outcome measures are available. In this UKMED data extract, applicants to UCAS in 2010 entered the medical register from 2015-18, 2011 applicants in 2016-8, 2012 applicants in 2017-18 and 2013 applicants in 2018. Applicants for 2014 would only have qualified in 2019 but the UKMED dataset did not yet include that years, and some earlier entrants would also be expected to qualify after 2018. For simplicity, outcome measures were restricted to the deciles of the UKFPO's Educational Performance Measure (EPM), the raw score of the UKFPO's Situational Judgement Test (SJT), and the score relative to the pass mark of the Prescribing Safety Assessment (PSA), all at first attempt, as these are the main outcomes from undergraduate training. Insufficient numbers of doctors had progressed further in postgraduate training to make analysis meaningful in this data extract.

Supplementary table 6 (presented also in the main paper) summarises the correlation matrix of the various measures. It is important to note that the large differences in Ns are primarily because some measures are present in applicants and used during *selection*, and others are undergraduate outcome measures from medical school, which of necessity are only present in *entrants*, and some are postgraduate outcome measures, only present in *graduates*, not all cohorts yet having reached that stage. The three parts of the correlation matrix are separated to clarify the distinction. Correlations of selection and outcome measures necessarily show range restriction because candidates have been selected on the basis of these measures, and in the case of graduates, selected and self-selected, so that they are less variable than would be the case in an unrestricted population of applicants. The most important question for these data is the extent to which Predicted and Attained A-level grades (shown in pink and green in Supplementary table 6) differ in how much they predict the three outcome measures, which typically are taken five or six years later.

Prediction of Educational Performance Measure (EPM). EPM is probably the most important outcome measure since it integrates educational performance across assessments for all but the final year of the undergraduate course[p]. Note that deciles are confusing, as UKFPO scores them in the reverse of the usual order, the first decile being highest performance and the tenth the lowest. Here for ease of interpretation we reverse the scoring in what we call *revDecile*, so that higher *revDeciles* indicate higher performance. EPM is a summary of outcome across assessments within a medical school, expressed as deciles of achievement within each school. EPM is predicted r=0.297 by attained A-level grades but only r=0.198 by predicted grades. Although in absolute terms those

[p] https://foundationprogramme.nhs.uk/wp-content/uploads/sites/2/2019/11/UKFP-2020-EPM-Framework-Final-1.pdf

correlations may seem small it must be remembered that they are range restricted, and the construct level predictive validity, taking into account range restriction and measurement error is likely to be much higher [25]. N is large for these correlations and hence the differences are highly significant using Meng and Rosenthal's test for correlated correlations [26], Z = 12.6, with $p<10^{-33}$. Although predicted grades predict less well than attained grades, they may predict differently, and hence contribute something over and above attained grades in predicting outcome? Entering predicted grades after attained grades in a multiple regression shows a highly significant but small additional prediction of predicted grades (beta=.052, compared with beta=.269 for attained grades). Attained grades are therefore substantially better at predicting undergraduate outcome, but predicted grades may have a small amount of variance which is not shared with attained A-levels.

Can other measures replace attained A-level grades for predicting EPM? In the absence of attained grades, to what extent can other selection measures such as GCSE grades, U(K)CAT and BMAT replace the predictive variance in attained A-level grades? Regressing EPM on just predicted grades gives multiple R = .198, compared with an R of 0.297 when regressed on just actual grades. Adding GCSEs to Predicted grades increases R to .225, while also including U(K)CAT and BMAT increases R to .231, although that is still far short of the .297 from A-levels alone. Interestingly if Actual Grades are now added in to the equation as well, R increases to .308, which is higher than the R for just actual grades. Exploration suggests that the effect is due to the additional effect of GCSEs grades compared with just having attained A-level grades in the model (R=.306; Beta(attained grades)=.268, beta(GCSES)=.077). Overall therefore if only Predicted Grades are available, an improved prediction is obtain by including GCSEs and U(K)CAT/BMAT, although the model still falls short of that of actual A-levels in terms of prediction.

Private and State Sector schooling and EPM. The UKCAT-12 study [27] found that medical students educated in the private sector performed less well at medical school than those educated in the state sector with equivalent A-level grades. It is important to replicate that finding in the present data, and to explore the extent to which there are effects related to predicted as opposed to attained grades. Overall 6149 (26.8%) of students were educated in the private sector, compared with 16805 (73.2%) in the state sector. Supplementary figure 1 plots *revDecile* in relation to attained and predicted grades, separately by private and state education. Visually it is immediately clear that there is an overall main effect of schooling, the lines for private sector schools (pale green and pale red) being below those for state schools. Note that the point for private schools with predicted grades <AAA is missing, as N was very small, because of few private schools predicting grades below AAA. Considering just attained grades, regression showed effects of both A-level grade (b=.299 (SE .008)[q], beta=.301, t=35.24, $p<10^{-100}$) and private schooling (b= -.292 (SE=.053), beta= -.047, t=-5.478, $p=4\times10^{-8}$), but the addition of an interaction was not significant (t=0.746, p=.455) meaning that the slopes in supplementary figure 1.a 1.b are the same. A similar analysis for predicted grades found effects of predicted grade (b=.213 (SE .009), beta=.201, t=22.94, $p<10^{-100}$) and private schooling (b= -.256 (SE .055), beta= -.041, t=-4.679, p=0.000003), but the addition of an interaction was not significant (t=0.680746, p=.455), again meaning that the slopes are similar in the two types of school in supplementary figure 1.b. The standard errors for the effects of private schooling suggest that the difference between the slope is similar for actual and predicted grades.

Supplementary table 6 contains a number of other interesting features. [Note that the main paper has some extended descriptive statistics and additional comments in the text].

Other outcome measures in relation to actual and predicted A-levels. There are four other outcome variables, two undergraduate and two postgraduate. For the undergraduate measures, PSA mark (supplementary figure 2) and SJT score (supplementary figure 3), both correlate more strongly with

[q] Actual and Predicted grades are scored on the basis of A*=12, A=10 etc so are in the range 6 to 36 for three best grades. b=.299 therefore means an increase of 0.3 deciles per step on the A-level grade score, and therefore a full A-level grade (e.g A*AA compared with AAA is 0.6 EPM deciles higher).

attained A-level grades than predicted A-levels (PSA: Z= 10.31, $p<10^{-23}$; SJT Z= 4.38, p=0.000012). The two postgraduate outcome measures, are based on smaller, but still substantial, numbers of doctors, MRCP(UK) Part 1 being taken by 910 doctors, and MRCS Part A by 440 doctors. Both outcomes have higher correlations with attained A-level grades than predicted grades, MRCP(UK) Part 1 correlating 0.421 with actual A-level grades (supplementary figure 4), and 0.283 with predicted grades (Z= 4.54, p=.000055), and MRCS Part A correlating 0.421 with actual grades (supplementary figure 5) compared with 0.358 with predicted grades (Z= 3.67, p=.000238). The five outcome measures therefore show the same broad pattern of results.

Correlations of outcome measures and the status of the SJT. The five outcome measures correlate well with each other (mean r = .420)[r], as might be expected given the academic backbone [29]. Noteworthy is the relatively low correlation of SJT with EPM (.319) and PSA (.346), compared with the correlation of EPM and PSA (.470). That pattern is repeated when postgraduate exams are included, the four non-SJT assessments showing a higher correlation (mean r=.499) than the correlations of the four non-SJT assessments with SJT (mean r = .322). Overall that suggests that SJT may be measuring a construct that is different in part from the other more academic assessments, and that will need investigating more closely in the future. It is also of interest when considering predicted grades that SJT correlates only slightly better with actual grades than predicted grades (.195 vs .160), compared with the other four outcomes (.297 vs .198; .306 vs .226; .421 vs .283; and .358 vs .181; mean r = .346 vs .222) raising the possibility that predicted grades may include some non-academic variance which then is predictive for SJT. That can be tested by regressing SJT on actual and predicted grades, when including predicted grades increases R from .195 to .206. The model including both grade types, shows an effect of actual grades (beta=.153, t=14.8, $p=10^{-49}$) and an effect of predicted grades (beta=.077, t=7.42, $p=1.2\times10^{-13}$), so that the beta effect of predicted grades is 50% of that for actual grades, compared with the earlier regression for deciles, where the beta of .052 for predicted grades is only 19% of the beta of .269 for attained grades.

The present SJT test is administered at the time of graduation. There is also a separate SJT administered as a part of the U(K)CAT tests, which was only introduced in 2014, and none of that cohort have outcome variables in the present data set. However it is of interest that, for the 4286 applicants in 2014 with U(K)CAT SJT, there is a correlation of .145 with Actual A-levels and .127 with predicted A-levels (Z=1.28, p=0.192). Overall it is possible that SJT tests are behaving differently to academic outcomes, despite moderately strong correlations of SJT with other academic outcomes. SJT tests are, "designed to assess for key attributes … including commitment to professionalism, coping with pressure, effective communication, patient focus, and working effectively as part of a team" [30 31].

Correlations of A-levels with GCSEs, U(K)CAT and BMAT. Without going into details, attained A-levels correlate more strongly with U(K)CAT and BMAT (r=.326 and .416) than do predicted A-levels (r=.272 and .326), suggesting that admissions tests are particularly assessing academic attainment. However GCSE grades show the reversed pattern and correlated *more strongly with predicted A-levels* (0.452) than with attained A-level grades (0.421), perhaps implying that teachers in part use GCSE grades to make predictions (as has been found in a previous study[18]).

Correlations of admissions tests with outcome measures. Neither of the two admissions tests, U(K)CAT and BMAT, has a strong prediction of EPM (r=.115 and .089 respectively), and both clearly

---

[r] Note that there are too few doctors who took both MRCP(UK) Part 1 and MRCS Part A to be able to calculate a correlation. Elsewhere we have looked at the relatively rare groups of doctors taking both MRCP(UK) and MRCGP, and shown high correlations between performance on the two assessments 28. Wakeford R, Denney ML, Ludka-Stempien K, et al. Cross-comparison of MRCGP & MRCP(UK) in a database linkage study of 2,284 candidates taking both examinations: Assessment of validity and differential performance by ethnicity. *BMC Medical Education* 2015;15(1 (doi:10.1186/s12909-014-0281-2)), making it likely that the same would also apply to MRCP(UK) Part 1 and MRCS Part A.

correlate less with EPM than does attained A-levels, r=.297, despite A-levels showing range restriction due to a ceiling effect at A*. PSA and SJT though show a somewhat different picture. PSA correlates more highly with BMAT (r=.321) than with U(K)CAT (r=.238), and the correlation with BMAT is higher than that with attained A-levels (r=.306). In contrast U(K)CAT and BMAT both correlate similarly with SJT (r=.243 and .249), and both correlations are higher than with attained A-levels (r=.195). BMAT and U(K)CAT both show correlations with the two postgraduate outcomes (0.200 and 0.378 for MRCP(UK) Part 1 and 0.181 and 0.319 for MRCS Part A, but both are lower than the correlations with A-levels (0.421 and 0.358). Taken overall, BMAT has somewhat higher correlations with the five outcome measures (mean r = .269) than does U(K)CAT (mean r = .195) but both correlate less with outcomes than do attained A-levels (mean r=.315). U(K)CAT correlates at a similar level to predicted A-levels (mean r=.209) but BMAT at a somewhat higher level.

McManus IC, *et al. BMJ Open* 2021; 11:e047354. doi: 10.1136/bmjopen-2020-047354

4. Supplementary Tables 1, 2, 3, 4, 5 & 6 and Supplementary Figures 1, 2, 3, 4 & 5

Supplementary Table 1: Comparison of predicted and forecasted grades in 2009 and 2012.

Estimated grades			Max grade	Over-predicted	Accurate	Under-predicted	Population	Source
**Predicted**	**October**	**2009**	**A**	**42%**	**52%**	**7%**	**UCAS**	Everett and Papageorgiou (2011)
**Forecasted**	**May**	**2009**	**A**	**33%**	**55%**	**12%**	**OCR**	Gill and Rushton (2011)
Forecasted-Predicted				*-9%*	*3%*	*5%*		
**Predicted**	**October**	**2012**	**A***	**68%**	**20%**	**12%**	**UCAS**	UCAS (2017)
**Forecasted**	**May**	**2012**	**A***	**39%**	**48%**	**13%**	**OCR**	Gill and Chang (2013)
Forecasted-Predicted				*-30%*	*29%*	*1%*		

Supplementary Table 2: Comparison of predicted and attained A-level grades in medical school applicants, 2010-2018

a) Counts of number of cases

		Attained Alevel grades						
		E	D	C	B	A	A*	*Total*
Predicted Alevel grades	E	**200**	35	10	5	0	0	*255 (0%)*
	D	235	**610**	155	35	10	0	*1045 (0%)*
	C	635	1220	**2110**	505	95	5	*4570 (2%)*
	B	635	2095	4755	**7355**	1695	175	*16715 (7%)*
	A	430	1925	8785	35640	**61950**	12655	*121390 (51%)*
	A*	50	135	635	6025	42815	**43395**	*93060 (39%)*
	Total	*2185*	*6020*	*16450*	*49570*	*106570*	*56235*	*237030*
		(1%)	*(3%)*	*(7%)*	*(21%)*	*(45%)*	*(24%)*	

b) Percentages within predicted grades

		Attained Alevel grades						
		E	D	C	B	A	A*	*Total*
Predicted Alevel grades	E	**79%**	14%	..	..	..	..	*100%*
	D	23%	**58%**	15%	3%	..	..	*100%*
	C	14%	27%	**46%**	11%	2%	..	*100%*
	B	4%	13%	28%	**44%**	10%	1%	*100%*
	A	0%	2%	7%	29%	**51%**	10%	*100%*
	A*	0%	0%	1%	7%	46%	**47%**	*100%*
	Total	*1%*	*3%*	*7%*	*21%*	*45%*	*24%*	*100%*

b) Percentages within predicted grades

		Attained Alevel grades						
		E	D	C	B	A	A*	*Total*
Predicted Alevel grades	E	**9%**	1%	..	..	..	..	*0%*
	D	11%	**10%**	1%	0%	..	..	*0%*
	C	29%	20%	**13%**	1%	0%	..	*2%*
	B	29%	35%	29%	**15%**	2%	0%	*7%*
	A	20%	32%	53%	72%	**58%**	23%	*51%*
	A*	2%	2%	4%	12%	40%	**77%**	*39%*
	Total	*100%*	*100%*	*100%*	*100%*	*100%*	*100%*	*100%*

Supplementary Table 3: Comparison of predicted and forecasted A-level grades in medical school applicants, 2010-2018

Subject	N	Mean Predicted	Mean Actual	Actual minus Predicted	r (Pearson)
Chemistry	62815	10.35	9.37	-0.98	0.623
Biology	61190	10.59	9.78	-0.82	0.632
Maths & Stats	54635	10.79	9.77	-1.02	0.600
Physics & Engineering	13870	10.67	9.52	-1.15	0.635
General Studies & Critical Thinking	6785	9.66	7.70	-1.96	0.534
Modern Languages	6720	10.59	9.74	-0.85	0.571
Psychology	6190	10.19	9.12	-1.07	0.631
Geography	4015	10.84	9.95	-0.89	0.538
History	3850	10.48	9.49	-0.99	0.546
English Literature & Language	3815	10.32	9.52	-0.80	0.681
Further Maths	2950	11.07	9.62	-0.80	0.681
Economics & Business Studies	2765	10.36	9.47	-0.89	0.577
Religious Studies	1890	10.45	9.40	-1.05	0.626
Art & Design	1035	10.60	10.03	-0.57	0.681
Latin & Classical Studies	675	10.74	9.65	-1.09	0.576
Music	640	10.49	9.51	-0.97	0.567
Sociology	525	9.51	8.49	-1.02	0.679
Computer Studies & ICT	475	9.89	8.82	-1.06	0.704
Physical Education	470	10.61	9.81	-0.80	0.610
Government & Politics	380	10.07	9.16	-0.91	0.656
Theatre Studies & Drama	260	10.14	9.02	-1.11	0.624
Science -- Misc & General	260	8.30	7.24	-1.06	0.821
Law	190	9.42	8.55	-0.87	0.766
Philosophy	155	10.37	9.06	-1.32	0.639
Classical Greek	115	10.90	9.98	-0.92	0.463
Media Studies	75	8.03	7.25	-0.78	0.798

Supplementary Table 4: Comparison of predicted and attained EPQ grades in medical school applicants, 2010-2018

a) EPQ: Counts of number of cases

		Attained EPQ grade						
		E	D	C	B	A	A*	*Total*
	E	**5**	0	0	0	0	0	*5 (0%)*
Predicted	D	0	**15**	0	0	0	0	*20 (0%)*
EPQ	C	10	10	**120**	15	5	0	*160 (2%)*
grade	B	15	40	90	**355**	100	30	*625 (7%)*
	A	40	135	405	920	**1970**	1150	*4620 (49%)*
	A*	15	35	125	375	940	**2420**	*3915 (42%)*
	Total	*85*	*240*	*740*	*1670*	*3010*	*3605*	*9345*
		(1%)	*(3%)*	*(8%)*	*(18%)*	*(32%)*	*(39%)*	

b) EPQ: Percentages within predicted grades

		Attained EPQ grade						
		E	D	C	B	A	A*	*Total*
	E	**..**	..	..	..	..	..	*...*
Predicted	D	..	**..**	..	..	..	..	*...*
EPQ	C	..	..	**46%**	..	..	..	*100%*
grade	B	..	13%	28%	**44%**	10%	1%	*100%*
	A	0%	2%	7%	29%	**51%**	10%	*100%*
	A*	..	0%	1%	7%	46%	**47%**	*100%*
	Total	*1%*	*3%*	*7%*	*21%*	*45%*	*24%*	*100%*

Supplementary Table 5: Comparison of predicted and forecasted SQA Highers and SQA Advanced Highers in medical school applicants, 2010-2018

a) SQA Advanced Highers: Counts of number of cases

		Attained SQA Highers							
		D7	C6	C5	B4	B3	A2	A1	*Total*
	D	**90**	0	0	0	0	0	0	95 (0.6%)
Predicted	C	20	**210**	**220**	15	10	10	0	485 (3.3%)
SQA	B	95	140	190	**455**	**490**	305	30	1700 (11.6%)
Highers	A	255	495	905	1405	2010	**5335**	**1955**	12360 (84.4%)
	Total	**465**	**845**	**1320**	**1875**	**2510**	**5645**	**1985**	14640 (100%)
	Total	*3.2%*	*5.8%*	*9.0%*	*12.8%*	*17.2%*	*38.6%*	*13.6%*	

b) SQA Advanced Highers: Percentages within predicted grades

		Attained SQA Highers							
		D7	C6	C5	B4	B3	A2	A1	*Total*
	D	**97%**	..	..	..	..	..	..	100%
Predicted	C	..	**43%**	**45%**	..	..	..	..	100%
SQA	B	6%	8%	11%	**27%**	**29%**	18%	2%	100%
Highers	A	2%	4%	7%	11%	16%	**43%**	**16%**	100%
	Total	*3.2%*	*5.8%*	*9.0%*	*12.8%*	*17.2%*	*38.6%*	*13.6%*	

Supplementary Table 6: Correlation matrix of selection measures, undergraduate outcome measures, and postgraduate outcome measures (separated by grey lines for clarity). Cells indicate Pearson correlation and N. (NB presented as figure 3 in the main paper).

			**Selection measures applicants**				**Undergraduate outcome measures**			**Postgraduate outcome measures**	
		GCSE grades	Predicted Alevels	Alevel grades	UKCAT	BMAT	EPM	SJT	PSA	MRCP(UK) Part 1	MRCS Part A
	GCSE grades	**1**	**0.452**	**0.421**	**0.265**	**0.223**	**0.180**	**0.190**	**0.201**	**0.212**	**0.173**
			22150	22150	22145	4935	12230	12185	12265	890	430
	Predicted A-level grades	**0.452**	**1**	**0.585**	**0.272**	**0.326**	**0.198**	**0.160**	**0.226**	**0.283**	**0.181**
**Selection measures**		22150		22955	22520	5225	12560	12515	12600	910	440
**in all applicants**	Attained A-level grades	**0.421**	**0.585**	**1**	**0.326**	**0.416**	**0.297**	**0.195**	**0.306**	**0.421**	**0.358**
		22150	22955		22520	5225	12560	12515	12600	910	440
	UKCAT total	**0.265**	**0.272**	**0.326**	**1**	**0.483**	**0.115**	**0.243**	**0.238**	**0.200**	**0.181**
		22145	22520	22520		5080	12385	12340	12420	900	435
	BMAT sections 1 and 2	**0.223**	**0.326**	**0.416**	**0.483**	**1**	**0.089**	**0.239**	**0.321**	**0.378**	**0.319**
		4935	5225	5225	5080		4850	4840	4875	450	240
	UKFPO EPM decile	**0.180**	**0.198**	**0.297**	**0.115**	**0.089**	**1**	**0.319**	**0.470**	**0.509**	**0.535**
**Undergraduate**		12230	12560	12560	12385	4850		12515	12505	905	440
**outcome measures**	UKFPO SJT score	**0.190**	**0.160**	**0.195**	**0.243**	**0.239**	**0.319**	**1**	**0.346**	**0.351**	**0.274**
		12185	12515	12515	12340	4840	12515		12475	905	435
	PSA score	**0.201**	**0.226**	**0.306**	**0.238**	**0.321**	**0.470**	**0.346**	**1**	**0.500**	**0.483**
		12265	12600	12600	12420	4875	12505	12475		910	440
	MRCP(UK) Part 1	**0.212**	**0.283**	**0.421**	**0.200**	**0.378**	**0.509**	**0.351**	**0.500**	**1**	**…**
**Postgraduate**		890	910	910	900	450	905	905	910		10
**outcome measures**	MRCS Part A	**0.173**	**0.181**	**0.358**	**0.181**	**0.319**	**0.535**	**0.274**	**0.483**	**…**	**1**
		430	440	440	435	240	440	435	440	10	

Supplementary figure 1: Mean EPM *revDeciles* (95% CI) in relation to actual A-level grades (green) and predicted A-level grades (red), state sector schooling shown in darker colours and private sector schooling in paler colours.

a.)

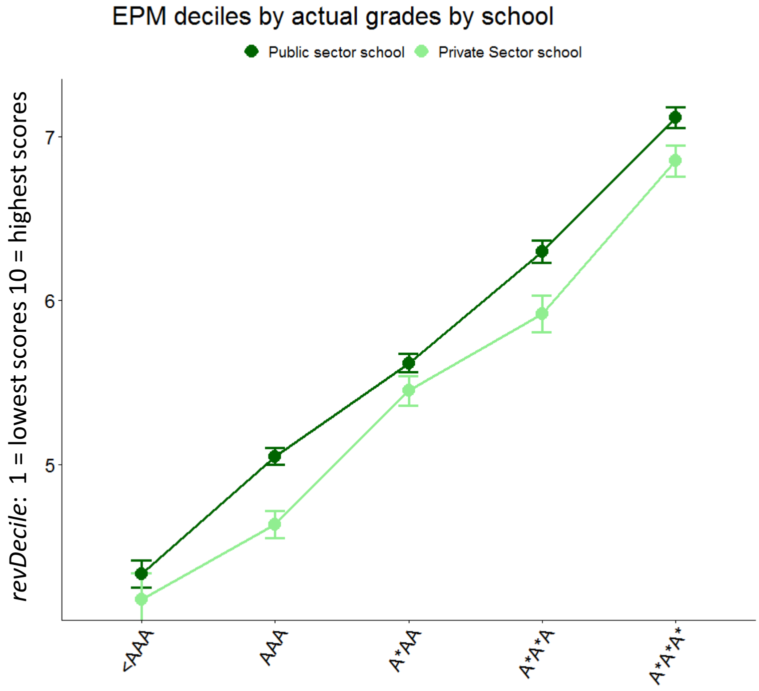

b.)

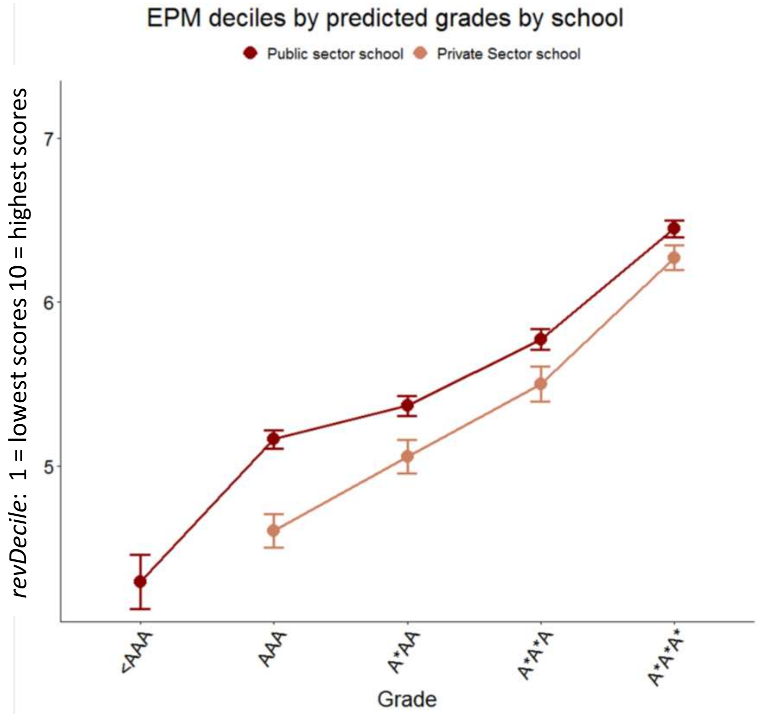

Supplementary figure 2: Mean PSA mark in relation to actual A-level grades (green) and predicted A-level grades (red)

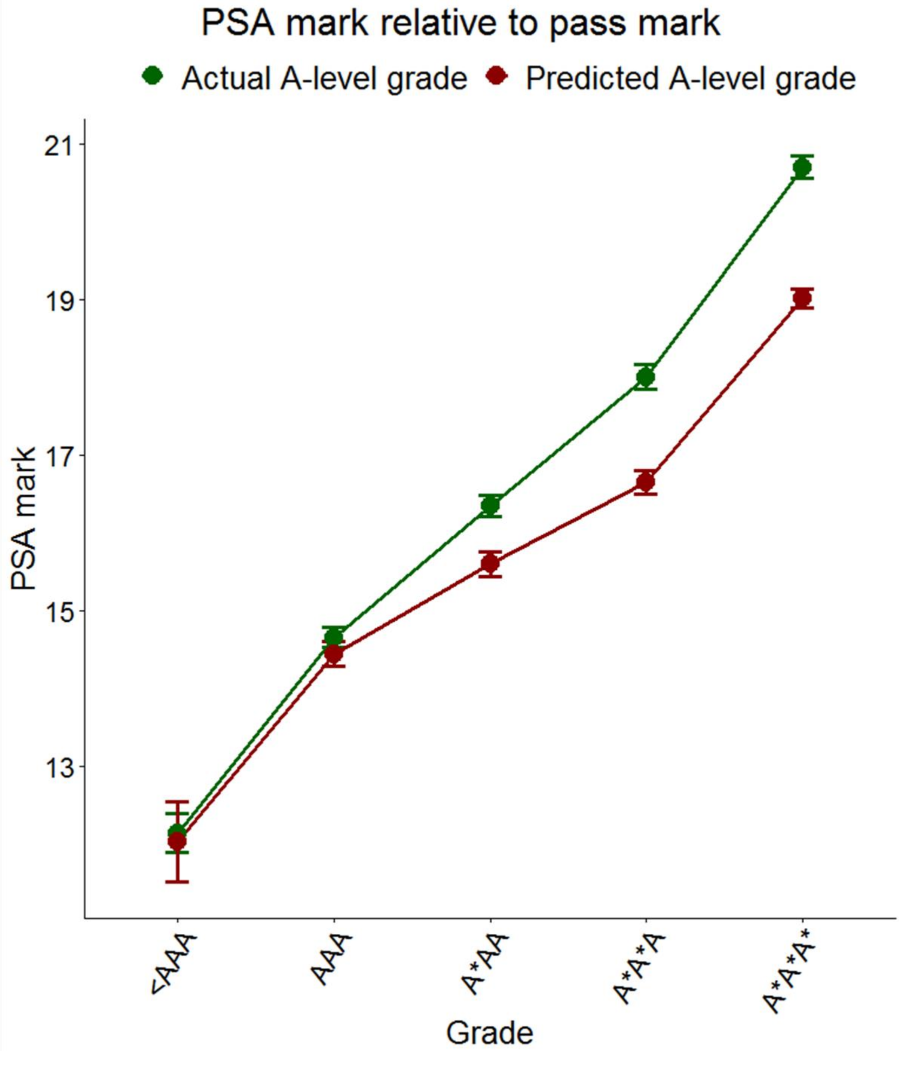

Supplementary figure 3: Mean SJT mark in relation to actual A-level grades (green) and predicted A-level grades (red)

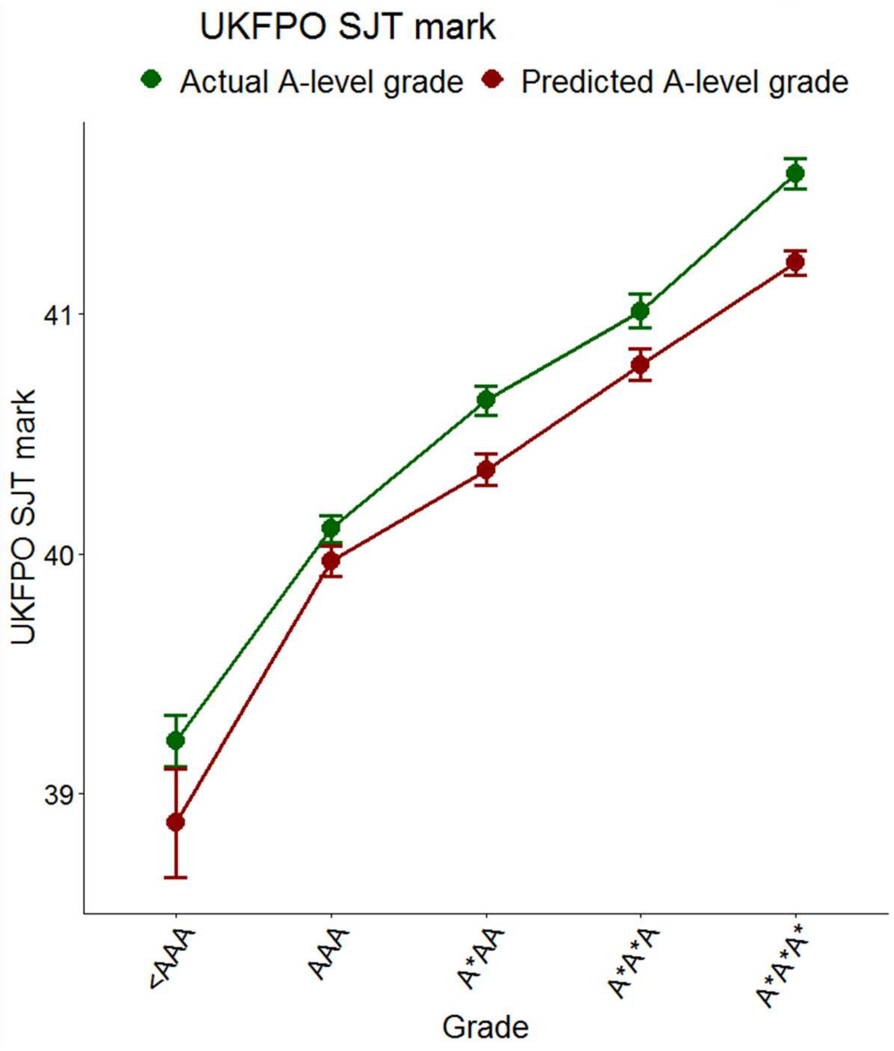

Supplementary figure 4: Mean MRCP(UK) Part 1 mark in relation to actual A-level grades (green) and predicted A-level grades (red)

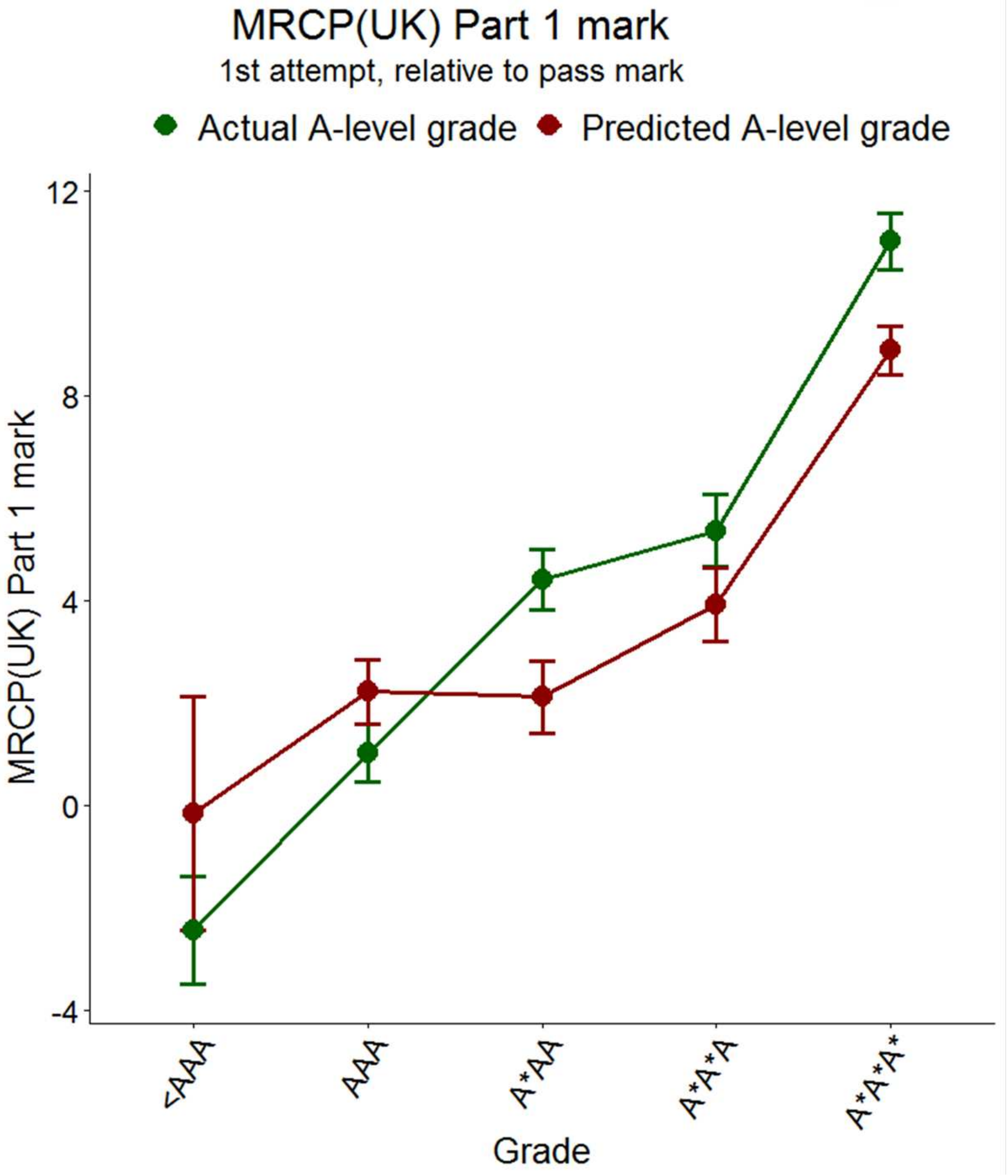

Supplementary figure 5: Mean MRCS Part A mark in relation to actual A-level grades (green) and predicted A-level grades (red)

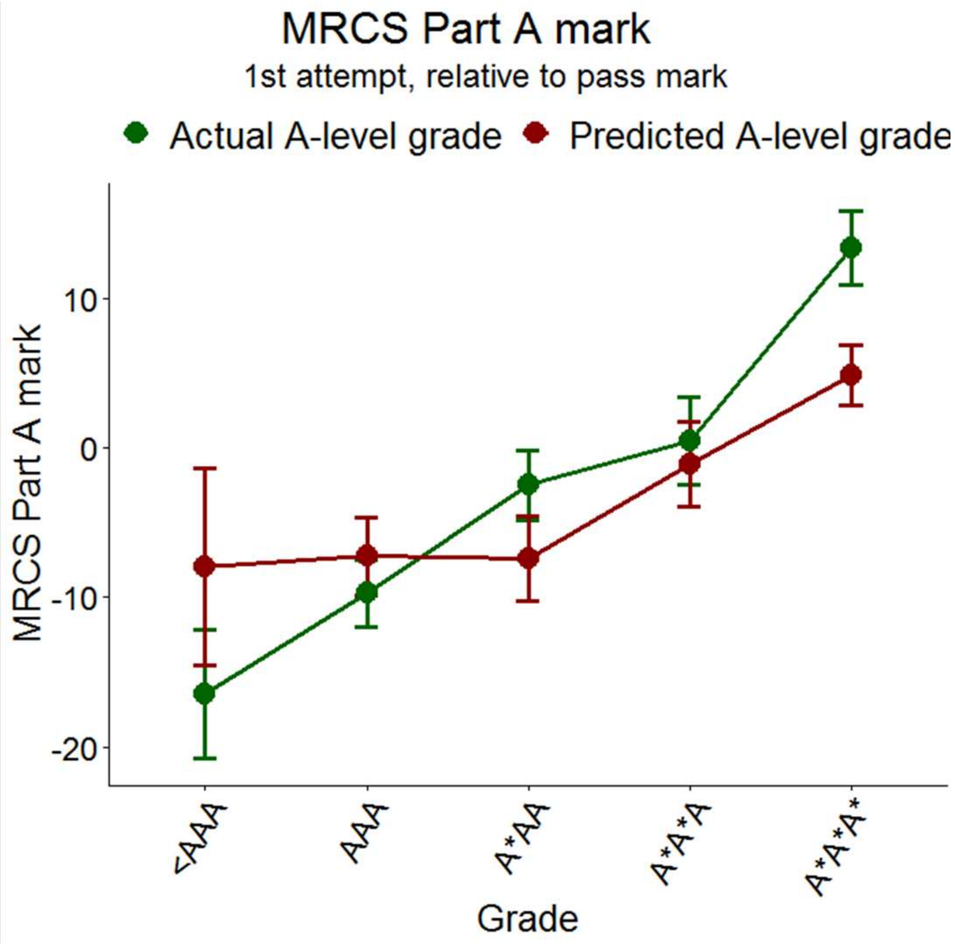

5. *Appendix*: Are independent (private sector) schools more accurate in their A-level predictions?

A recurrent suggestion in the literature is that schools in the private sector (Independent Schools) are more accurate in their A-level predictions than those in the state sector. That suggestion raises many issues, not least concerned with social equity, but a key one to be resolved is whether the differences mainly are secondary to differences in overall attainment level, and as a result may be artefacts due to the ordinal nature of A-level grades and to A* being the ceiling for A-level grades, and hence is a level beyond which candidates cannot reach. This appendix looks in detail at that question. The description is lengthy, technical, and partly didactic, and therefore has not been included in the main text or the main supplementary text. The conclusion is actually relatively simple: *Independent schools are not actually more accurate in their predictions, but they look that way because of having higher attained grades.*

The data. Appendix table 1 shows, in a similar format to those in the main paper, the A-level grades in P89 for applicants from state schools (defined as Comprehensives, Academies, Sixth Form Colleges, Tertiary Colleges and Technical Colleges) and private schools (defined as Independent Schools and Grammar Schools), with results restricted to first A-level attempts, duplicates removed, and where both A-level grades and predicted grades were available. Results are at the subject level, and therefore contain multiple subjects from individual applicants.

A simple glance at Appendix table 1 suggests that indeed Private Sector schools are more accurate, 53.3% of predictions being accurate compared to 45.5% of predictions from State Sector schools. Private schools also show a lower rate of over-prediction (40.5% compared with 48.0%), but not of under-prediction (6.2% vs 6.4%). These differences need however to be put into the context of higher overall attainment in private schools, where 29% of grades were A* compared with 20% in state schools, a finding that reflects most private schools being selective and therefore inevitably taking higher ability entrants. Since attained A* grades are more frequent in private schools, it is not surprising that predicted A* grades are also more frequent in private schools, 48% vs 35%. The question therefore is whether the differences in accuracy are secondary to differences in overall performance. That question is best answered using *polychoric correlations*, which need description.

Polychoric and tetrachoric correlations. Polychoric and tetrachoric correlations are used frequently in psychometrics when dealing with binary and ordinal data. The need for them is shown by a simple 2x2 association table of the sort often tested using a chi-square test. Consider Appendix figure 1, which is a simple association table for characteristics P and Q in 100 individuals. 80% of cases have P present but only 50% of cases have Q present, meaning that the *marginal proportions* are not the same (80% vs 50%). A chi-square test is highly significant (chi-squared = 25, 1 df, p=0.0000006) meaning that there is an association between P and Q. But what is the size of that association? Often in this situation a Pearson or Spearman correlation is calculated, and these give r_p=0.5 and r_s=0.5, which suggests a moderately strong association.

However there is a problem in using the Pearson correlation, as a careful look at the table shows because the number of cases in which P is absent but Q is present, in the top right-hand corner, is zero. In other words the association could not be any stronger, but the correlation is still only 0.5, whereas a perfect correlation is usually taken as being 1. The problem arises because the marginal proportions of P and Q are not the same, one being 0.5 and the other 0.8. If these two marginal proportions had been identical than all of the cases could have been on the diagonal and then the

Pearson correlation would indeed have been 1. So what does one do in the case where the marginal proportions are not the same? The answer is another correlation developed by Pearson, called the *tetrachoric* or *polychoric* correlation for 2x2 or for larger tables respectively.

The tetrachoric correlation assumes that the data actually come from a bivariate normal distribution with some underlying correlation, and asks if that distribution were divided horizontally and vertically, what the correlation would have to be to create the contingency table that has been found. The lower part of Appendix figure 1 shows that diagrammatically[s], the four quadrants containing the proportions of data in the contingency table. The calculation is easily carried out in the *R* function *polychor()* in the *polycor* library, and for the table in Appendix figure 1 it gives the answer that r_t=0.994, which effectively is r_t=1. The tetrachoric correlation therefore corresponds to our intuitive sense of what the correlation should be. The underlying bivariate normal distribution is assumed to have means of zero and standard deviations of one. *polychor()* then tells us that the thresholds for cutting the distribution need to be at 0.842 for P and 0 for Q. The threshold for Q at zero tells us that the cutting point is 0 standard deviations from the mean, and therefore 50% of cases are above the threshold and 50% below. The threshold for P is 0.842 standard deviations below the mean, and hence 20% of cases are below the threshold and 80% of cases above it. The marginal proportions of P and Q are then replicated.

For a 2x2 table it is always possible to fit the tetrachoric correlation and the marginal proportions exactly. If the table is larger, giving a polychoric correlation, the marginal proportions and the cell frequencies cannot always be fitted exactly as the normal distribution may not be entirely appropriate, and in that case maximum likelihood estimates of the correlation and thresholds are found. The polychoric calculation for an *m x n* table also provides a set of *(m-1)* and *(n-1)* thresholds for each of the variables, and it is possible to see if step sizes between the levels are equal. Polychoric correlations therefore are used for data where both measures are *ordinal* and for which it seems reasonable to assume an underlying latent distribution which is normal.

Polychoric correlations for A-level grades. A-level grades are certainly at least ordinal in nature, but it is not clear that they are *equal interval,* the step from, say, D to C not necessarily being the same size as the step from B to A. Polychoric calculations allow the direct estimation of the step sizes between grades. If step sizes are not equal then many conventional statistics are not optimal. Equal interval scales are measures such as length, where the increments are identical in size (so the difference between, say, 2 cms and 3 cms is the same length as the difference between 10 cms and 11 cms). A-levels are often scored on a simple basis of allocating points, such as A*=12, A=10, B=8, C=6, D=4 and E=2 (and indeed we have done this elsewhere here), but that can sometimes be misleading in situations such as calculating correlations between actual and predicted grades, partly because marginal proportions are not the same, and partly because the data are *censored*, grades above A* not being possible, however capable is a candidate, and hence over-prediction is not possible for estimated grades of A*. In the case of a high ability group such as applicants to medical school the latter is problematic as state and private schools predict an A* grade for 35% and 48% of exams. To put it another way, were a grade of A** available then many examinees might have merited it [32], albeit probably more at private than state schools. There is also potentially a problem of computing total A-level scores (so that, say, AAA with 30 points is regarded as equivalent to A*AB or A*A*C, which may not be exactly the case, although the approximation is probably good enough for most purposes).

[s] The correlation is actually drawn at 0.9 to make things pedagogically clearer, as a correlation of 1 is effectively a straight line.

Fitting polychoric correlations to A-level grades from state and private schools. The key question at present is whether private schools are more accurate in their predictions (53.3%) than state schools (45.5%) – see Appendix table 1. Accuracy can be considered in two ways, as the presence of systematic error (technically, 'bias'), equivalent to rates of A* etc being different in two groups, and random error, in terms of the correlation or lack of correlation between two sets of scores. Although the overall accuracy of private schools is *higher* than state schools, the correlation of predicted and actual grades is *lower* in private schools, with Pearson correlations of 0.635 in state schools and 0.552 in private schools (Appendix table 1), with a similar pattern for Spearman correlations. That suggests a potential problem in interpreting the data. Calculating the polychoric correlations suggests a very different picture, since the polychoric correlations in state schools (r_t= 0.717) and private schools (r_t= 0.678) are far more similar, particularly in comparison with the differences between the Pearson (or Spearman) correlations.

Interpreting the polychoric correlations is helped by a diagram. Appendix figure 2.a may look complex, but it summarises a lot of information about state sector applicants. The axes are on a normal distribution for the underlying latent scale, and so the units are standard deviations, from -4 to +4 SDs. Note these are not SDs for the raw data, but for the latent distribution. The polychoric correlation for the state sector is 0.717, and that is shown by the blue ellipse which is plotted to cover 99.9% of the data, which is reasonable given the large sample sizes. The dashed blue and yellow line on the diagonal is the line of equality for attained grades on the horizontal axis and predicted grades on the vertical axis. The vertical and horizontal lines show the thresholds separating the various A-level grades for attained and predicted grades. Appendix table 2 summarises the various thresholds and their intervals for state and private schools. As an example, for attained grades, the threshold separating A from A* (Appendix table 2, row 4, column A:A*) is 0.83, and so the vertical line in Appendix figure 2.a separating A from A* is at 0.83. Similarly the horizontal line for predicted grades separating A from A* is at 0.39 (row 2 in Appendix table 2). The intersection of these two lines is shown by a large red circle, which is *below* the blue-yellow dashed line, which indicates that the threshold for attained grades is higher than the threshold for predicted grades, so that it is easier to be predicted an A* than to attain an A*. The other vertical and horizontal lines show the thresholds between B and A (B:A), C and B (C:B), D and C (D:C) and E and D (E:D). As for A*:A, all of the intersections, shown as red dots, are below the dashed blue-yellow line of equality, showing that predicted grades are always more generous than attained grades. Row 6 of Appendix table 2 shows that on average the threshold for attained grades is 0.73 SDs lower than for predicted grades. The coloured boxes in Appendix figure 2.a are equivalent to the coloured boxes in appendix table 1, with grey indicating accuracy, green and blue indicating under-estimation, and red and yellow over-estimation. More of the figure is red or yellow than is blue or green, indicating the overall over-estimation by predicted grades. It is also clear from the figure that the differences between the thresholds are not equal. The width of D, from E:D to D:C, is smaller than the width of A (from B:A to A:A*), these values being shown in row 10 of Appendix table 2 for predicted grades and row 12 for attained grades. The widths of E and A* cannot be calculated as they stop either at minus infinity or plus infinity. It is clear that the scale is not equal interval, with less change being required to move from D to C than from B to A. Statistical analyses should take care therefore in assuming that the usual A* to E scale of grades is equal interval, and can be averaged.

The key question for this appendix is the extent to which state and private sector predictions are different. Appendix figure 2.b shows an equivalent plot to Appendix figure 2.a but for private sector A-levels. At a glance it is not easy to see any obvious difference, but it is important to remember that the latent scales for both graphs each have a mean of zero and SD of one. However looking carefully shows that the threshold for attained grades at A* is at 0.55 for private sector students compared

with 0.83 for state sector students (see rows 4 and 5 of table Appendix table 2). The threshold is lower for private sector students and hence more of these students will attain an A*, as is the case in Appendix table 2. All of the thresholds for the private sector students are actually moved to the left compared with state sector students (and compare the sizes of the A*A* boxes and the EE boxes in the two figures. Appendix figure 3 summarises the thresholds more clearly for attained and predicted grades in state and private sector schools. All thresholds are shown on the same horizontal scale. Attained grades for private schools are to the right of predicted grades, shown by the thin blue diagonal lines (meaning an attained A* is harder to get than a predicted A*), and the same pattern is seen for state schools, and shown by the thin diagonal red lines. Private school attained grades are also to the left of state school attained grades, shown by a thin purple line (with thresholds lower for private school students meaning that they get more A* grades). Similarly, private school predicted grades are also to the left of state school predicted grades, also shown by a thin purple line. A key feature of Appendix figure 3 is that the blue diagonal lines are parallel, the red diagonal lines are parallel and the purple diagonal lines are nearly parallel, meaning that the relationships of grade boundaries are the same in private and state schools, and for attained and predicted grades, but are merely slid along relative to one another. The state and private schools are therefore handling predicted grades in a way that is similar, and they are similar related in each case to attained grades.

The widths of the boxes in Appendix figure 2 are therefore very similar in state and private sector students, and are shown in rows 9 to 16 of Appendix table 2, particularly in rows 10 and 11, which compare predicted grades in state and private schools, and rows 12 and 13 which compare attained grades in private schools. The main difference between the two types of school is shown in the mean columns of rows 1 and 2 and rows 4 and 5, their mean differences being shown in the final column. Overall the state schools have thresholds which for predicted grades are on average are 0.47 SDs higher and for attained grades are 0.42 grades higher than for private sector schools (meaning that higher grades are harder to attain). These values are very similar and suggest that predictions in the two types of school are being carried out in a similar way, but the overall ability of private school students is higher, and that is reflected in the attained and predicted ways to a similar extent.

The private school students are therefore about 0.44 SDs higher on the latent scale than the state school students. As a result it is possible to plot state and private schools on the same graph (Appendix figure 4), with the only difference being that the private schools are further along the diagonal towards the top right corner. That difference accounts for all of the differences in the private and state school students, with all other differences in Appendix table 1 being artefacts of the artificial ceiling of the range at A*. To put it another way, were attained grades to be the same in state and private schools then the accuracy and the degree of over-estimation would be the same in the two types of schools.

In conclusion, conventional statistics comparing attained and predicted grades at A-level are inherently misleading, and suggest differences between groups which are probably not present, meaning that great care must be taken in interpretation.

McManus IC, *et al. BMJ Open* 2021; 11:e047354. doi: 10.1136/bmjopen-2020-047354

6. Appendix: Appendix Tables 1 & 2 and Appendix Figures 1, 2, 3 & 4.

Appendix table 1. Predicted vs Attained A-level grades in applicants from a) State Sector schools (non-Private schools) and b) Independent (Private sector) schools.

a) State Sector: Counts of number of cases

		Attained Alevel grades						
		E	D	C	B	A	A*	*Total*
	E	**140**	30	5	5	0	0	*180 (0%)*
Predicted	D	210	**420**	125	20	5	0	*780 (1%)*
Alevel	C	535	1050	**1545**	400	55	5	*3600 (2%)*
grades	B	500	1735	3910	**5190**	1090	115	*12540 (9%)*
	A	270	1330	6250	24310	**36915**	7425	*76495 (53%)*
	A*	25	75	395	3950	24725	**21410**	*50580 (35%)*
	Total	*1680*	*4645*	*12230*	*33870*	*62785*	*28960*	*144175*
		(1%)	*(3%)*	*(8%)*	*(23%)*	*(44%)*	*(20%)*	

	Prediction:			Correlations:		
Under	Accurate	Over		Pearson	Spearman	Polychoric
6.4%	**45.5%**	48.0%		0.635	0.590	0.717

a) Private Sector: Counts of number of cases

		Attained Alevel grades						
		E	D	C	B	A	A*	*Total*
	E	**15**	0	0	0	0	0	*15 (0%)*
Predicted	D	15	**55**	15	5	0	0	*85 (0%)*
Alevel	C	50	85	**200**	40	10	0	*385 (1%)*
grades	B	60	185	430	**1085**	335	30	*2130 (3%)*
	A	65	300	1650	8785	**19500**	3935	*34235 (49%)*
	A*	5	20	115	1420	15270	**16635**	*33455 (48%)*
	Total	*205*	*640*	*2405*	*11340*	*35115*	*20600*	*70305*
		(0%)	*(1%)*	*(3%)*	*(16%)*	*(50%)*	*(29%)*	

Under-	Prediction:			Correlations:		
estimate	Accurate	Over		Pearson	Spearman	Polychoric
6.2%	**53.3%**	40.5%		0.552	0.523	0.678

Appendix table 2. Thresholds, and intervals between thresholds, for the grades for applicants at State and Private schools. Values in bold show mean values across rows and down columns.

1	**Ordinal Thresholds**		E:D		D:C		C:B		B:A		A:A*		Mean	State minus Private
2	Predicted	State	-3.11		-2.55		-1.89		-1.18		0.39		**-1.67**	0.47
3		Private	-3.51		-3.00		-2.47		-1.78		0.06		**-2.14**	
4	Attained	State	-2.31		-1.74		-1.13		-0.33		0.83		**-0.94**	0.42
5		Private	-2.57		-2.25		-1.68		-0.81		0.55		**-1.35**	
6	Predicted-Attained	State	-0.80		-0.81		-0.76		-0.85		-0.44		**-0.73**	0.02
7		Private	-0.76		-0.75		-0.79		-0.97		-0.48		**-0.75**	
8			**-2.18**		**-1.85**		**-1.45**		**-0.99**		**0.15**		**-1.26**	
9	**Threshold intervals**			D:C - E:D		C:B - D:C		B:A - C:B		A:A* - B:A				
10	Predicted	State		-0.57		-0.66		-0.71		-1.57			**-0.87**	0.02
11		Private		-0.51		-0.54		-0.69		-1.84			**-0.89**	
12	Attained	State		-0.57		-0.61		-0.80		-1.17			**-0.79**	-0.01
13		Private		-0.32		-0.58		-0.87		-1.36			**-0.78**	
14	Predicted-Attained	State		0.01		-0.05		0.09		-0.41			**-0.09**	-0.02
15		Private		-0.01		0.04		0.18		-0.48			**-0.07**	
16				**-0.33**		**-0.40**		**-0.47**		**-1.14**			**-0.58**	

Appendix figure 1. Demonstration of how a conventional Pearson or Spearman correlation between binary variables P and Q cannot achieve a correlation of one when marginal proportions of P and Q differ. However the tetrachoric correlation is one, within calculation and rounding errors, being estimated from underlying latent correlation shown in the diagram, with thresholds at -0.842 and 0 for P and Q.

	Q absent	Q present	P totals
P absent	**0**	**20**	20
P present	**50**	**30**	80
Qtotals	50	50	100
Correlatio	Pearson	Spearman	Tetrachoric
	0.5	0.5	0.994
Threshold	P	Q	
	-0.842	0	

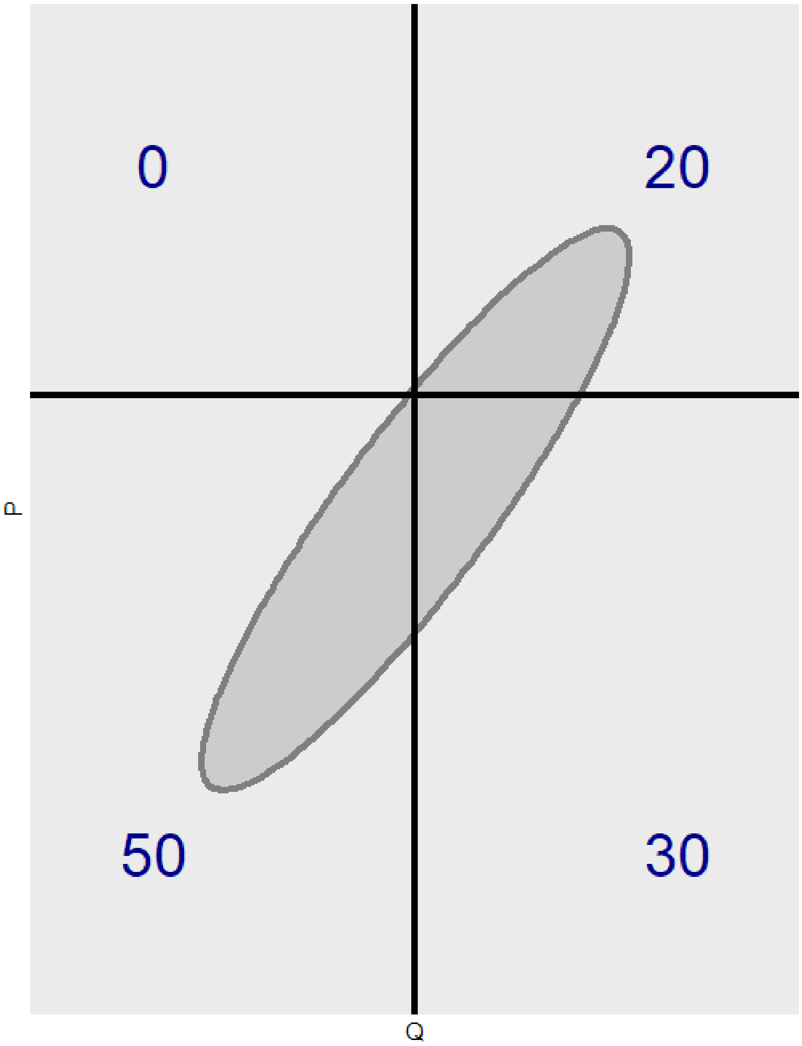

Appendix figure 2. Latent bivariate normal distribution for the relationship between attained A-level grades (horizontal) and predicted A-level grades (vertical). The correlation is represented by the blue ellipse. The dashed blue and yellow line is the line of equality of actual and attained grades. The vertical and horizontal black lines show the thresholds for the grades, shown as E, D, C, B, A and A*. The solid red dots and red line show where the thresholds for a grade intersect, with all below the main diagonal. Colours indicate over-prediction (yellow and pink) and under-prediction (green and blue).

2.a

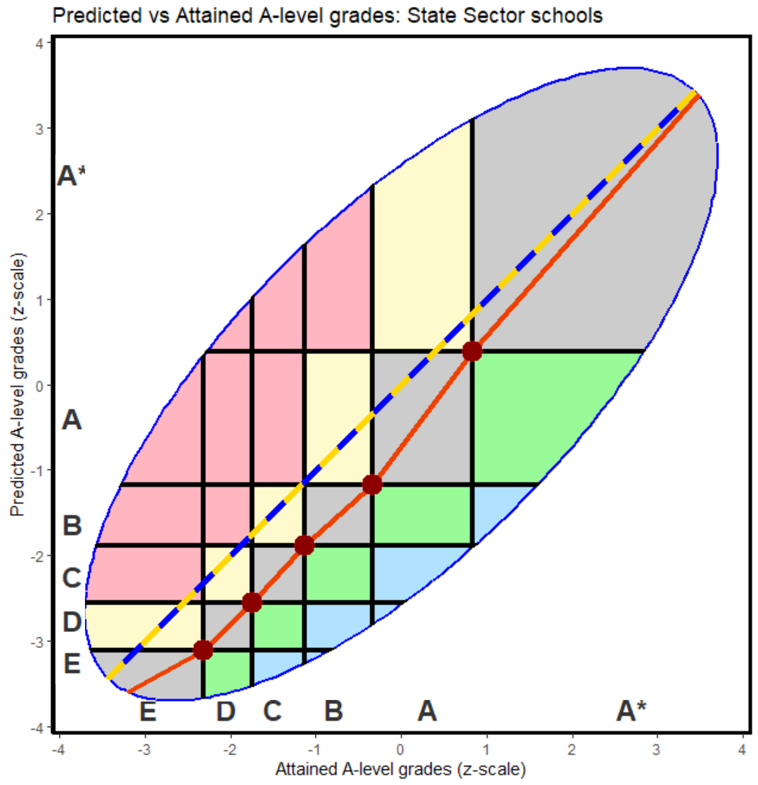

2.b

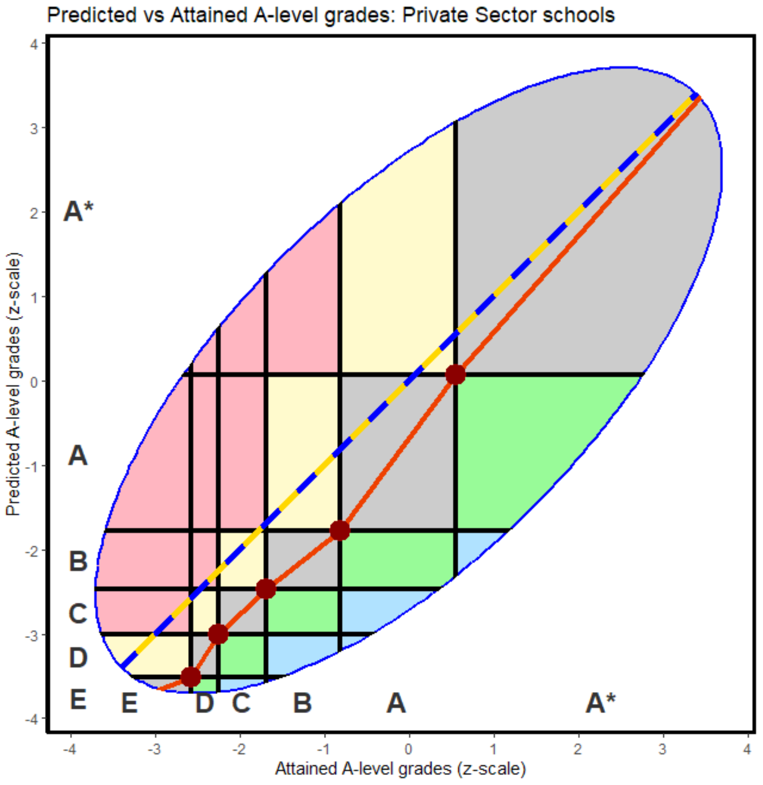

Appendix figure 3. Summary of polychoric thresholds shown on the horizontal axis, for private (blue) and state (red) schools, for attained grades (squares) and predicted grades (circles). Narrower diagonal lines show the links between attained and predicted grades for private (blue) and state (red) schools. Purple diagonal lines link equivalent points for private and state schools (e.g. attained with attained grades and predicted with predicted grades).

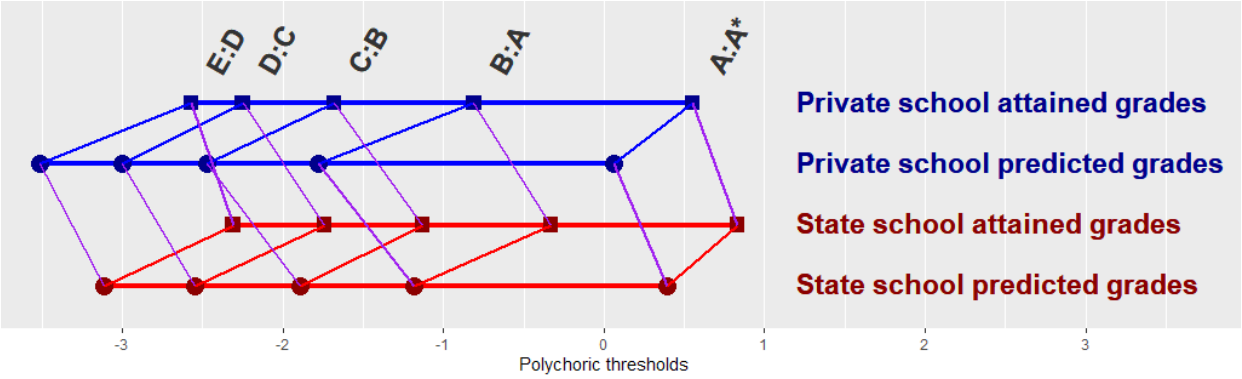

Appendix figure 4. See Appendix figure 2 for the majority of conventions. The fitted ellipses for state sector schools (red) and private sector schools (blue) are shown separately, with the same grade thresholds for both schools. The latent bivariate normal distributions for the two types of school differ entirely in their mean scores, that for private sector schools being shifted up and to the right (by the same amount). The school types therefore differ only in their mean ability levels.

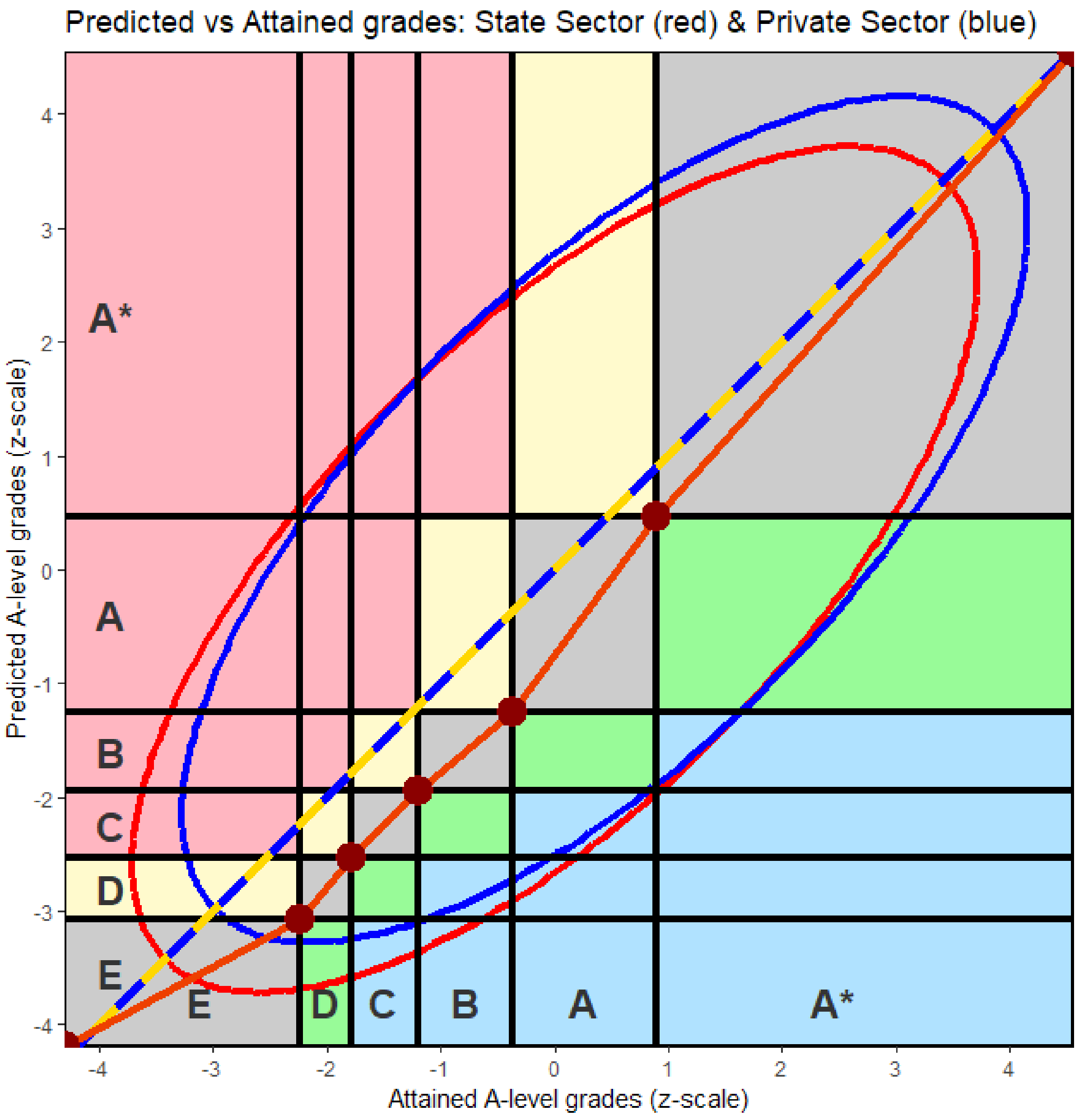

References

1. Petch JA. School estimates and examination results compared. Manchester: Joint Matriculation Board 1964.
2. Wilmut J. Experiences of summative teacher assessments in the UK. London: Qualifications and Curriculums Authority 2011.
3. Murphy RJL. Teachers' assessments and GCE results compared. *Educational Research* 1979;22(1):54-59.
4. Murphy RJL. O-level grades and teachers' estimates as predictors of the A-level results of UCCA applicants. *British Journal of Educational Psychology* 1981;51(1):1-9.
5. Everett N, Papageorgiou J. Investigating the Accuracy of Predicted A Level Grades as part of 2009 UCAS Admission Process. London: Department for Business, Innovation and Skills 2011.
6. Wyness G. Predicted grades: Accuracy and impact. A report of University and College Union. London: University and College Union (https://www.ucu.org.uk/media/8409/Predicted-grades-accuracy-and-impact-Dec-16/pdf/Predicted_grades_report_Dec2016.pdf) 2016.
7. Wyness G. Rules of the game: Disadvantaged students and the university admissions process. London: The Sutton Trust 2017.
8. Murphy R, Wyness G. Minority Report: the impact of predicted grades on university admissions of disadvantaged groups. London: **Centre for Education Policy and Equalising Opportunities (CEPEO) Working Paper No. 20-07, UCL Institute of Education** https://EconPapers.repec.org/RePEc:ucl:cepeow:20-07 2020.
9. UCAS. End of cycle report 2017: Qualifications and competition. Cheltenham: UCAS [https://www.ucas.com/data-and-analysis/ucas-undergraduate-releases/ucas-undergraduate-analysis-reports/2017-end-cycle-report] 2017.
10. Gill T, Rushton N. The accuracy of forecast grades for OCR Alevels: Statistics Report Series No 26. Cambridge: Cambridge Assessment [https://www.cambridgeassessment.org.uk/our-research/all-published-resources/statistical-reports/150215-the-accuracy-of-forecast%20-grades-for-ocr-a-levels-in-june-2012.pdf/] 2011.
11. Gill T, Chang Y. The accuracy of forecast grades for OCR A levels in June 2012: Statistics Report Series No.64. Cambridge: Cambridge Assessment Statistics Report Series No.64 2013.
12. Gill T, Benton T. The accuracy of forecast grades for OCR Alevels in June 2014: Statistics Report Series No 90. Cambridge: Cambridge Assessment [https://www.cambridgeassessment.org.uk/Images/241261-the-accuracy-of-forecast-grades-for-ocr-a-levels-in-june-2014.pdf] 2015.
13. UCAS. Factors associated with predicted and achieved A level attainment, August 2016. Cheltenham: UCAS: https://www.ucas.com/file/71796/download?token=D4uuSzur 2016.
14. McManus IC, Woolf K, Dacre J. The educational background and qualifications of UK medical students from ethnic minorities. *BMC Medical Education* 2008;8: 21 (http://www.biomedcentral.com/1472-6920/8/21)
15. Gill T. Methods used by teachers to predict final Alevel grades for their students. *Research Matters (UCLES)* 2019(28):33-42.
16. Lumb AB, Vail A. Applicants to medical school: the value of predicted school leaving grades. *Med Educ* 1997;31:307-11.
17. Richardson PH, Winder B, Briggs K, et al. Grade predictions for school-leaving examinations: do they predict anything? *Med Educ* 1998;32:294-97.
18. McManus IC, Richards P, Winder BC, et al. Medical school applicants from ethnic minorities: identifying if and when they are disadvantaged. *Brit Med J* 1995;310:496-500.
19. Boliver V. How fair is access to more prestigious universities? *British Journal of Sociology* 2013;64(2):344-64.

20. Woolf K, Harrison D, McManus IC. The attitudes, perceptions and experiences of medical school applicants following the closure of schools and cancellation of public examinations due to the COVID-19 pandemic in 2020. *medRxiv* 2020;submitted
21. Woolf K, Harrison D, McManus C. The attitudes, perceptions and experiences of medical school applicants following the closure of schools and cancellation of public examinations in 2020 due to the COVID-19 pandemic: a cross-sectional questionnaire study of UK medical applicants. *BMJ open* 2021;11(3):e044753.
22. McManus IC, Woolf K, Harrison D, et al. Calculated grades, predicted grades, forecasted grades and actual A-level grades: Reliability, correlations and predictive validity in medical school applicants, undergraduates, and postgraduates in a time of COVID-19. *medRxiv* 2020;doi: https://doi.org/10.1101/2020.06.02.20116830
23. Gill T, Rodeiro, C.V. Predictive validity of level 3 qualifications: Extended Project, Cambridge Pre-U, International Baccalaureate,BTEC Diploma. Cambridge: Cambridge Assessment: Cambridge Assessment Research Report 2014.
24. Thomson D. Moderating teaching judgments in 2020 [Blog post, 25th March 2020]. London: FFT Educational Lab: https://ffteducationdatalab.org.uk/2020/03/moderating-teacher-judgments-in-2020/ (accessed 16th April 2020) 2020.
25. McManus IC, Dewberry C, Nicholson S, et al. Construct-level predictive validity of educational attainment and intellectual aptitude tests in medical student selection: Meta-regression of six UK longitudinal studies. *BMC Medicine* 2013;11:243:doi:10.1186/741-7015-11-243.
26. Meng X-L, Rosenthal R, Rubin DB. Comparing correlated correlation coefficients. *Psychological Bulletin* 1992;111(1):172-75.
27. McManus IC, Dewberry C, Nicholson S, et al. The UKCAT-12 study: Educational attainment, aptitude test performance, demographic and socio-economic contextual factors as predictors of first year outcome in a collaborative study of twelve UK medical schools. *BMC Medicine* 2013;11 :244:doi:10.1186/741-7015-11-244.
28. Wakeford R, Denney ML, Ludka-Stempien K, et al. Cross-comparison of MRCGP & MRCP(UK) in a database linkage study of 2,284 candidates taking both examinations: Assessment of validity and differential performance by ethnicity. *BMC Medical Education* 2015;15(1 (doi:10.1186/s12909-014-0281-2))
29. McManus IC, Woolf K, Dacre J, et al. The academic backbone: Longitudinal continuities in educational achievement from secondary school and medical school to MRCP(UK) and the Specialist Register in UK medical students and doctors. *BMC Medicine* 2013;11:242:doi:10.1186/741-7015-11-242.
30. Patterson F, Zibarras L, Ashworth V. Situational judgement tests in medical education and training: Research, theory and practice: AMEE Guide No. 100. *Medical Teacher* 2016;38(1):3-17.
31. McManus IC, Harborne A, Smith D, et al. Exploring UK medical school differences: The *MedDifs* study of selection, teaching, student and F1 perceptions, postgraduate outcomes, and fitness to practise. *BMC Medicine* 2019;In press
32. McManus IC, Woolf K, Dacre JE. Even one star at A level could be "too little, too late" for medical student selection. *BMC Medical Education* 2008;8:16 (http://www.biomedcentral.com/1472-6920/8/16

1. Petch JA. School estimates and examination results compared. Manchester: Joint Matriculation Board 1964.
2. Wilmut J. Experiences of summative teacher assessments in the UK. London: Qualifications and Curriculums Authority 2011.
3. Murphy RJL. Teachers' assessments and GCE results compared. *Educational Research* 1979;22(1):54-59.

4. Murphy RJL. O-level grades and teachers' estimates as predictors of the A-level results of UCCA applicants. *British Journal of Educational Psychology* 1981;51(1):1-9.
5. Everett N, Papageorgiou J. Investigating the Accuracy of Predicted A Level Grades as part of 2009 UCAS Admission Process. London: Department for Business, Innovation and Skills 2011.
6. Wyness G. Predicted grades: Accuracy and impact. A report of University and College Union. London: University and College Union (https://www.ucu.org.uk/media/8409/Predicted-grades-accuracy-and-impact-Dec-16/pdf/Predicted_grades_report_Dec2016.pdf) 2016.
7. Wyness G. Rules of the game: Disadvantaged students and the university admissions process. London: The Sutton Trust 2017.
8. Murphy R, Wyness G. Minority Report: the impact of predicted grades on university admissions of disadvantaged groups. London: **Centre for Education Policy and Equalising Opportunities (CEPEO) Working Paper No. 20-07, UCL Institute of Education** https://EconPapers.repec.org/RePEc:ucl:cepeow:20-07 2020.
9. UCAS. End of cycle report 2017: Qualifications and competition. Cheltenham: UCAS [https://www.ucas.com/data-and-analysis/ucas-undergraduate-releases/ucas-undergraduate-analysis-reports/2017-end-cycle-report] 2017.
10. Gill T, Rushton N. The accuracy of forecast grades for OCR Alevels: Statistics Report Series No 26. Cambridge: Cambridge Assessment [https://www.cambridgeassessment.org.uk/our-research/all-published-resources/statistical-reports/150215-the-accuracy-of-forecast%20-grades-for-ocr-a-levels-in-june-2012.pdf/] 2011.
11. Gill T, Chang Y. The accuracy of forecast grades for OCR A levels in June 2012: Statistics Report Series No.64. Cambridge: Cambridge Assessment Statistics Report Series No.64 2013.
12. Gill T, Benton T. The accuracy of forecast grades for OCR Alevels in June 2014: Statistics Report Series No 90. Cambridge: Cambridge Assessment [https://www.cambridgeassessment.org.uk/Images/241261-the-accuracy-of-forecast-grades-for-ocr-a-levels-in-june-2014.pdf] 2015.
13. UCAS. Factors associated with predicted and achieved A level attainment, August 2016. Cheltenham: UCAS: https://www.ucas.com/file/71796/download?token=D4uuSzur 2016.
14. McManus IC, Woolf K, Dacre J. The educational background and qualifications of UK medical students from ethnic minorities. *BMC Medical Education* 2008;8: 21 (http://www.biomedcentral.com/1472-6920/8/21)
15. Gill T. Methods used by teachers to predict final Alevel grades for their students. *Research Matters (UCLES)* 2019(28):33-42.
16. Lumb AB, Vail A. Applicants to medical school: the value of predicted school leaving grades. *Med Educ* 1997;31:307-11.
17. Richardson PH, Winder B, Briggs K, et al. Grade predictions for school-leaving examinations: do they predict anything? *Med Educ* 1998;32:294-97.
18. McManus IC, Richards P, Winder BC, et al. Medical school applicants from ethnic minorities: identifying if and when they are disadvantaged. *Brit Med J* 1995;310:496-500.
19. Boliver V. How fair is access to more prestigious universities? *British Journal of Sociology* 2013;64(2):344-64.
20. Woolf K, Harrison D, McManus IC. The attitudes, perceptions and experiences of medical school applicants following the closure of schools and cancellation of public examinations due to the COVID-19 pandemic in 2020. *medRxiv* 2020;submitted
21. Woolf K, Harrison D, McManus C. The attitudes, perceptions and experiences of medical school applicants following the closure of schools and cancellation of public examinations in 2020 due to the COVID-19 pandemic: a cross-sectional questionnaire study of UK medical applicants. *BMJ open* 2021;11(3):e044753.
22. McManus IC, Woolf K, Harrison D, et al. Calculated grades, predicted grades, forecasted grades and actual A-level grades: Reliability, correlations and predictive validity in medical school

applicants, undergraduates, and postgraduates in a time of COVID-19. *medRxiv* 2020;doi: https://doi.org/10.1101/2020.06.02.20116830

23. Gill T, Rodeiro, C.V. Predictive validity of level 3 qualifications: Extended Project, Cambridge Pre-U, International Baccalaureate,BTEC Diploma. Cambridge: Cambridge Assessment: Cambridge Assessment Research Report 2014.
24. Thomson D. Moderating teaching judgments in 2020 [Blog post, 25th March 2020]. London: FFT Educational Lab: https://ffteducationdatalab.org.uk/2020/03/moderating-teacher-judgments-in-2020/ (accessed 16th April 2020) 2020.
25. McManus IC, Dewberry C, Nicholson S, et al. Construct-level predictive validity of educational attainment and intellectual aptitude tests in medical student selection: Meta-regression of six UK longitudinal studies. *BMC Medicine* 2013;11:243:doi:10.1186/741-7015-11-243.
26. Meng X-L, Rosenthal R, Rubin DB. Comparing correlated correlation coefficients. *Psychological Bulletin* 1992;111(1):172-75.
27. McManus IC, Dewberry C, Nicholson S, et al. The UKCAT-12 study: Educational attainment, aptitude test performance, demographic and socio-economic contextual factors as predictors of first year outcome in a collaborative study of twelve UK medical schools. *BMC Medicine* 2013;11 :244:doi:10.1186/741-7015-11-244.
28. Wakeford R, Denney ML, Ludka-Stempien K, et al. Cross-comparison of MRCGP & MRCP(UK) in a database linkage study of 2,284 candidates taking both examinations: Assessment of validity and differential performance by ethnicity. *BMC Medical Education* 2015;15(1 (doi:10.1186/s12909-014-0281-2))
29. McManus IC, Woolf K, Dacre J, et al. The academic backbone: Longitudinal continuities in educational achievement from secondary school and medical school to MRCP(UK) and the Specialist Register in UK medical students and doctors. *BMC Medicine* 2013;11:242:doi:10.1186/741-7015-11-242.
30. Patterson F, Zibarras L, Ashworth V. Situational judgement tests in medical education and training: Research, theory and practice: AMEE Guide No. 100. *Medical Teacher* 2016;38(1):3-17.
31. McManus IC, Harborne A, Smith D, et al. Exploring UK medical school differences: The *MedDifs* study of selection, teaching, student and F1 perceptions, postgraduate outcomes, and fitness to practise. *BMC Medicine* 2019;In press
32. McManus IC, Woolf K, Dacre JE. Even one star at A level could be 'too little, too late' for medical student selection. *BMC Medical Education* 2008;8:16 (http://www.biomedcentral.com/1472-6920/8/16)
