# Supplementary material for: Predictive validity of A-level grades and teacher-predicted grades in UK medical school applicants: a retrospective analysis of administrative data in a time of COVID-19
Source: BMJ Open. 2021 Dec 16;11(12):e047354. doi: 10.1136/bmjopen-2020-047354 (PMC8678544; doi:10.1136/bmjopen-2020-047354)
Supplement: Author's manuscript [file bmjopen-2020-047354.draft_revisions.pdf]

BMJ Open is committed to open peer review. As part of this commitment we make the peer review history of every article we publish publicly available.

When an article is published we post the peer reviewers' comments and the authors' responses online. We also post the versions of the paper that were used during peer review. These are the versions that the peer review comments apply to.

The versions of the paper that follow are the versions that were submitted during the peer review process. They are not the versions of record or the final published versions. They should not be cited or distributed as the published version of this manuscript.

BMJ Open is an open access journal and the full, final, typeset and author-corrected version of record of the manuscript is available on our site with no access controls, subscription charges or pay-per-view fees (http://bmjopen.bmj.com).

If you have any questions on BMJ Open's open peer review process please email info.bmjopen@bmj.com

BMJ Open

The predictive validity of A-level grades in medical school applicants in a time of COVID-19: A retrospective analysis of administrative data

Journal:	*BMJ Open*
Manuscript ID	bmjopen-2020-047354
Article Type:	Original research
Date Submitted by the Author:	26-Nov-2020

Complete List of Authors:	McManus, Ian; UCL Medical School, Research Department of Medical Education<br>Woolf, Katherine; UCL Medical School, Research Department for Medical Education<br>Harrison, David; UCL Medical School, Research Department for Medical Education<br>Tiffin, Paul; University of York, Department of Health Sciences; Hull York Medical School, Health Professions Education Unit<br>Paton, Lewis; University of York,<br>Cheung, Kevin; Cambridge Assessment<br>Smith, Daniel; General Medical Council

Keywords:	COVID-19, MEDICAL EDUCATION & TRAINING, EDUCATION & TRAINING (see Medical Education & Training)

SCHOLARONE™
Manuscripts

BMJ

I, the Submitting Author has the right to grant and does grant on behalf of all authors of the Work (as defined in the below author licence), an exclusive licence and/or a non-exclusive licence for contributions from authors who are: i) UK Crown employees; ii) where BMJ has agreed a CC-BY licence shall apply, and/or iii) in accordance with the terms applicable for US Federal Government officers or employees acting as part of their official duties; on a worldwide, perpetual, irrevocable, royalty-free basis to BMJ Publishing Group Ltd ("BMJ") its licensees and where the relevant Journal is co-owned by BMJ to the co-owners of the Journal, to publish the Work in this journal and any other BMJ products and to exploit all rights, as set out in our licence.

The Submitting Author accepts and understands that any supply made under these terms is made by BMJ to the Submitting Author unless you are acting as an employee on behalf of your employer or a postgraduate student of an affiliated institution which is paying any applicable article publishing charge ("APC") for Open Access articles. Where the Submitting Author wishes to make the Work available on an Open Access basis (and intends to pay the relevant APC), the terms of reuse of such Open Access shall be governed by a Creative Commons licence – details of these licences and which Creative Commons licence will apply to this Work are set out in our licence referred to above.

Other than as permitted in any relevant BMJ Author's Self Archiving Policies, I confirm this Work has not been accepted for publication elsewhere, is not being considered for publication elsewhere and does not duplicate material already published. I confirm all authors consent to publication of this Work and authorise the granting of this licence.

For peer review only

The predictive validity of A-level grades in medical school applicants in a time of COVID-19: A retrospective analysis of administrative data

I C McManus [a,b,e,*]

i.mcmanus@ucl.ac.uk ORCID 0000-0003-3510-4814

Katherine Woolf [a,c,e]

k.woolf@ucl.ac.uk ORCID 0000-0003-4915-0715

David Harrison [a,e]

david.harrison@ucl.ac.uk ORCID 0000-0002-6639-6752

Paul A Tiffin [b,d,f]

paul.tiffin@york.ac.uk ORCID 0000-0003-1770-5034

Lewis W Paton [b,f]

lewis.paton@york.ac.uk ORCID 0000-0002-3328-5634

Kevin Yet Fong Cheung [b,g]

Cheung.K@cambridgeenglish.org ORCID 0000-0002-9548-2932

Daniel T. Smith [a,b,h]

daniel.smith@gmc-uk.org ORCID 0000-0003-1215-5811

[a] UKMEDP089 project
[b] UKMEDP051 project
[c] UKMEDP089 Principal Investigator
[d] UKMEDP051 Principal Investigator
[e] Research Dept of Medical Education, University College London, London WC1E 6BT, UK
[f] Department of Health Sciences, University of York, Heslington, York, YO10 5DD, UK
[g] Cambridge Assessment, Shaftesbury Road, Cambridge CB2 8EA, UK
[h] General Medical Council, Regent's Place, 350 Euston Road, London NW1 3JN, UK
* Author for correspondence: i.mcmanus@ucl.ac.uk

For peer review only

Abstract

Objectives

Calculate the predictive validity and reliability of predicted A-level grades compared to attained A-level grades. Model potential consequences for UK medical schools of replacing public A-level examination grades with teacher-estimated calculated grades as a result of the COVID-19 pandemic.

Design

Longitudinal observational study using UK Medical Education Database data.

Setting

UK medical education and training.

Participants

Dataset 1: 81,202 medical school applicants in 2010 to 2018 with predicted and attained A-level grades. Dataset 2: 22,150 18-year old medical school applicants in 2010 to 2014 with predicted and attained A-level grades, of whom 12,600 had medical school assessment outcomes and 1,340 had postgraduate outcomes available.

Outcome measures

Correlations between predicted and attained A-level grades and undergraduate and postgraduate medical examination results. Estimated undergraduate medical school achievement.

Results

Dataset 1: 48.8% of predicted grades were accurate, grades were over-predicted in 44.7% of cases and under-predicted in 6.5% of cases. Dataset 2: Undergraduate and postgraduate outcomes were predicted significantly better by attained than by predicted A-level grades. The lower predictive validity of teacher-estimated grades suggests medical students selected in 2020 are likely to under-attain, with 13% more gaining the equivalent of the current lowest decile of performance and 16% fewer gaining the equivalent of the current top decile, with follow-on effects for postgraduate training.

Conclusions

The replacement of A-level examination grades with teacher-estimated calculated grades is likely to result in the medical students selected in 2020 having lower academic performance at medical school compared to previous years. Medical schools will probably also need to consider whether additional teaching is needed for entrants who are struggling, or might have missed out on important aspects of A-level teaching, with extra support being needed.

For peer review only

For peer review only - http://bmjopen.bmj.com/site/about/guidelines.xhtml

Strengths and limitations of this study

- First comparison of the predicted validity of predicted and attained A-level grades using performance in subsequent undergraduate and postgraduate assessments.
- Large sample size of UK medical applicants from 2010 to 2018 provides adequate statistical power.
- Analysis of complete population data mean the results are unlikely to be biased.
- This study is with medical applicants only, meaning generalisability to students on other courses is uncertain.

Background

> "… the … exam hall [is] a level playing field for all abilities, races and genders to get the grades they truly worked hard for and in true anonymity (as the examiners marking don't know you). [… Now we] are being given grades based on mere predictions." Yasmin Hussein, letter to *The Guardian*, March 29th 2020 [1].

> "[Let's] be honest, this year group will always be different…" Dave Thomson, blogpost on *FFT Educational Lab* [2]

> "One headmistress commented that 'entrance to university on teachers' estimates may be fraught with unimagined difficulties'. … If there is in the future considerable emphasis on school assessment, some work of calibration is imperatively called for." James Petch, December 1964[3].

UK schools, in response to the COVID-19 pandemic, closed on 20th March 2020, and Key Stage 5 [Level 3] public examinations such as A-levels and SQA assessments were cancelled for summer 2020. On April 3rd *Ofqual* (Office of Qualifications and Examinations Regulation) in England announced that A-level, GCSE and other exams under its purview in England would be replaced by *Calculated Grades,* at the core of which are teachers' estimates of the grades that their students would attain (called *Centre Assessment Grades, CAGs*), which would then be moderated by *Ofqual* using a computer algorithm which included the prior performance of the school attended by candidates – see the Calculated Grades subsection below for details. The Scottish Qualification Authority (SQA) and other national bodies also announced similar processes for their examinations. Inevitably the announcement of Calculated Grades resulted in confusion and uncertainty in examination candidates, particularly those needing A-levels or SQA Advanced Highers[a] to meet conditional offers for admission to university in autumn 2020. Universities also faced a major problem, having had A-levels taken away, which are, "the single most important bit of information [used in selection]" [4].

Some of the tensions implicit in Calculated Grades are well seen in the quotation above by Yasmin Hussein, a GCSE student in Birmingham, with its clear emphasis that a key strength of current examination systems such as GCSEs, A-levels and similar qualifications, is the *anonymity* and *externality* of the assessors, who know nothing of the students whose work they are marking. In contrast the replacement of actual grades attained in the exam hall with what Hussein describes as 'mere predictions' raises a host of questions, not the least being the possibility of bias when judgements are made by teachers[b].

---

[a] SQA Highers are taken the year before (rather like AS-levels used to be) and therefore they will be available for 2020 applicants. Advanced Highers will not be available and will be estimated.

[b] A number of students, designated as 'external' or 'private', do not have regular teachers who can make such assessments and Ofqual has announced that they will not be able to receive calculated grades. Cases may

For peer review only - http://bmjopen.bmj.com/site/about/guidelines.xhtml

Context of the current paper and the situation at the time of writing

Since the emergence of COVID-19 in Europe in early 2020, the situation has been constantly and rapidly changing. At the time of writing in November 2020, Europe is in the midst of a 'second wave' and England is in a second national lockdown. To help the reader situate the current paper in context, we explain briefly here what the exams situation was in the UK from April to August 2020, and provide more detailed in a postscript. The remainder of the paper should be read as it was published in pre-print in May 2020, when exams had been cancelled, the process for awarding Calculated Grades announced, but no grades yet released.

In Spring 2020, as universities entered into the final phases of the annual academic cycle of student selection, we considered in this paper the potential problems of using teacher-estimated grades such as the Calculated Grades proposed by Ofqual, rather than attained grades obtained in the usual way via examinations. We published the current paper in pre-print in May 2020 so that admissions tutors could see the paper within the admissions cycle. By August 2020 some immediate impacts were shown when the algorithms used by regulators resulted in many students, particularly those from historically poorly performing schools, having their expected results adjusted downwards. This forced the Scottish Government, followed by the English and Welsh Governments, to accept teacher-estimated Centre Assessment Grades (CAGs) without moderation by an algorithm or the Calculated Grade, whichever was the higher.

As predicted in this paper, this scrapping of the algorithm resulted in a very significant increase in grades compared to 2019 (see https://ffteducationdatalab.org.uk/2020/08/gcse-and-a-level-results-2020-how-grades-have-changed-in-every-subject/). One short-term impact of the increase in grades was to increase the number of students meeting university conditional offers. The longer-term impacts are still to be seen, but the current paper offers some clues, calculating the predictive validity of teacher-estimated predicted grades submitted to UCAS at application to university for applicants from 2010 to 2018 (i.e. before the pandemic) compared to attained grades, and modelling the potential effects on undergraduate and postgraduate performance in medical students and doctors of selecting medical students on teacher-estimated grades such as the Centre Assessment Grades submitted by schools to Ofqual during the pandemic in summer 2020.

Medical school admissions

This paper will mainly concentrate on medical school applications. Medical education has a range of useful educational measures, including admissions tests during selection, and outcomes at the end of undergraduate training, which are linked together through UKMED (United Kingdom Medical Education Database; https://www.ukmed.ac.uk/). UKMED provides a sophisticated platform for assessing predictive validity in multiple entry cohorts in undergraduate and postgraduate training [5]. The paper should also be read in parallel with a second study from some members of the present team which assesses attitudes and perceptions to calculated grades and other changes in selection of current medical school applicants in the UKMACS (UK Medical Applicants Cohort Study) [6].

Fundamental questions about selection in 2020 concern the likely nature of Calculated Grades, and the extent to which they will predict outcomes to the same extent as currently do *actual or attained grades*. The discussion will involve actual grades, and then four types of teacher-estimated grades: predicted grades (sent to UCAS at application to university), centre assessment grades (CAGs –

---

include students taking a gap year and retaking A-levels, or a student studying GCSE or A-level German at a *Samstagsschule* who for administrative convenience takes the exam at a local school where the subject is not actually taught. There no doubt are many other A-level candidates affected as well.

For peer review only - http://bmjopen.bmj.com/site/about/guidelines.xhtml

submitted by schools to Ofqual in 2020) calculated grades (CAGs adjusted using an algorithm) and forecasted A-level grades (submitted by teachers to exam boards pre-2015 as a quality check for real exam grades). These are summarised in a box below, together with final grades, which were the grades eventually accepted by UCAS and were the higher of the calculated grade or centre assessed grade.

Calculated grades

The status of calculated grades was made clear by *Ofqual* in April 2020:

> "The grades awarded to students will have equal status to the grades awarded in other years and should be treated in this way by universities, colleges and employers. On the results slips and certificates, grades will be reported in the same way as in previous years". [7], p.6.

The decisions of *Ofqual* are supported by Ministerial statement, and universities and other bodies have little choice therefore but to abide by them, although that does not mean that other factors may not need to be taken into account in some cases, as often occurs when applicants do not attain the grades in conditional offers.

None of the above means that calculated grades actually *will be* equivalent to conventional attained grades. Calculated grades will not actually *be* attained grades, they may well behave differently to attained grades, and in measurement terms they actually *are not* attained grades, even though in administrative and even in legal terms, by fiat, they have to be treated as equivalent[c]. From the perspective of educational research, although the key issue is the extent to which calculated grades actually will or can behave in an identical way to attained grades.

Box 1: A-level grades: Actual, predicted, centre assessment, calculated, final, forecasted, and teacher-estimated grades

Actual or attained grades. The grades awarded by examination boards/awarding organisations based on written and other assessments which are set and marked externally. Typically sat in *May and June of year 13*, with results announced in *mid-August*.

Predicted grades. Teacher estimates of the likely attained grades of candidates, provided to UCAS in the *first term of year 13*, and by *October 15th* for medical and some other applicants.

Centre assessment grades. Used in the production of Calculated grades (see below). Provided by examination centres (typically schools) between 1st and 12th of June 2020, consisting of teacher-estimated grades and candidate rankings within examination centres.

Calculated grades. The final grades to be provided for candidates by exam boards for Summer 2020 assessments, in the absence of attained grades. Based on centre assessment grades, with final calculated grades involving standardisation/adjustement by exam boards using an algorithm. Calculated grades, "will have equal status to the grades awarded in other years and should be treated in this way by universities, colleges and employers" (Ofqual). These grades were often referred to as the 'algorithm grades' and were abandoned by the UK governments in August 2020

Final grades. The grades used by UCAS in the 2020 admissions cycle – the higher of the teacher estimated grade or the centre assessment grade

Forecasted grades. Prior to 2015, teachers, in *May of Year 13*, provided to exam boards a forecast of the likely grades of candidates along with rankings. Forecasted grades therefore take place later in the academic cycle than predicted grades, close to the time examinations are actually sat.

Teacher-estimated grades. Generic term used in this paper to refer to grades estimated by teachers. Includes predicted grades, centre assessment grades, calculated grades, and forecasted grades.

For peer review only - http://bmjopen.bmj.com/site/about/guidelines.xhtml

In April 2020 Ofqual issued guidance on how *calculated grades* would be provided for candidates for whom examinations have been cancelled. Essentially, teachers will be required, for individual candidates taking individual subjects within a *candidate assessment centre (*usually a school), to estimate *grades for* candidates, and then to *rank order* candidates within grades, to produce *centre assessment grades*. A statistical standardisation process will then be carried out centrally using a computer algorithm. Ranking is needed because standardisation, "will need more granular information than the grade alone" ([7] p.7}, presumably to break ties at grade boundaries which occur because of standardisation. Standardisation, to produce *calculated grades*, will use an algorithm that takes into account the typical distribution of results from that centre for that subject in the three previous years, along with aggregated centre data on SATS and previous exam attainment as in GCSEs[d]. This approach is consistent with Ofqual's approach to standard-setting. Following Cresswell [8], Ofqual has argued that during times of change in assessments, and perhaps more generally, there should be a shift away from "comparable performance" (i.e. criterion-referencing), and that there is an "ethical imperative" to use "comparable outcomes" (i.e. norm-referencing) to minimise advantages and disadvantages to the first cohort taking a new assessment, as perhaps also for later cohorts as teachers improve at teaching new assessments [9].

Ofqual says that centre assessment grades, the core of calculated grades, "are not the same as … predicted grades provided to UCAS in support of university applications" [10], (p.7). Predicted grades in particular are provided by schools in October of year 13 and centre assessment grades in May/June of year 13, seven months later, when Ofqual says that teachers should also consider classwork, bookwork, assignments, mock exams and previous examinations such as AS-levels (taken only by a minority of candidates now), but should *not* include GCSE results, or any student work carried out after 20th March. Whether centre assessment grades, or calculated grades – centre assessment grades moderated by the algorithm - will be fundamentally different from predicted grades is ultimately an empirical question, which should be answerable when UCAS data for 2020 are available for medical school applicants in UKMED. In the meantime, it seems a reasonable assumption, except for a small proportion of candidates who have improved dramatically from October 2019 to March 2020, that centre assessment grades and hence calculated grades will probably correlate highly with earlier predicted grades. Predicted grades, which have been collected for decades, should therefore act as a reasonable proxy in research terms for centre assessment grades and therefore calculated grades.

The rationale for using A-level grades in selection

Stepping back slightly it is worth revisiting the reasons that A-levels exist and why universities use them in selection. A-levels assess at least three things: subject knowledge, intellectual ability, and study habits such as conscientiousness [11]. Knowledge and understanding of, say, chemistry is probably necessary for high level study of medical science and medicine, to which it provides an underpinning, and experience suggests that students without such knowledge may have problems. A-levels also provide evidence for a student's intellectual ability and capability of extended study at a high level. A-levels are regarded as a 'gold standard' qualification because of the rigour and objectivity of their setting and marking. Their measurement is therefore *reliable*, and the presumption is that they are also *valid*, in the many senses of that word [12-14], and as a result are *unbiased*. A crucial assumption is of *predictive validity*, that future outcomes at or after university are higher or better in those who have higher or better A-levels, as found both in predicting degree classes in general [15-17] and medical school performance in particular [18,19]. At the other

[d] It was this standardisation processed that Governments reversed in August 2020 after the protests against calculated grades.

For peer review only - http://bmjopen.bmj.com/site/about/guidelines.xhtml

extreme, A-levels could be compared conceptually with, say, a mere assertion by a friend or colleague that, “Oh yes, they know lots of chemistry”. That is likely neither to be reliable, valid nor unbiased, and hence is a base metal compared with the gold standard of A-levels. The empirical question therefore is where on the continuum from gold to base, lie calculated grades or predicted grades.

The issue of predictive validity has been little discussed in relation to calculated grades, but in a recent *TES* (*Times Educational Supplement*) survey of teachers, there were comments that, “predictions and staff assessments would never have the same validity as an exam”, so that, “Predictions, past assessment data and mock data is not sufficient, and will never beat the real thing in terms of accuracy." [20]. The current changes in university selection inevitably mean that difficult policy decisions need to be made by universities and medical schools. Even in the absence of direct, high-quality, evidence, policy-makers still have an obligation to make decisions, and, therefore it is argued, must take theory, related evidence, and so on, into account [21]. This paper will provide both a review of other evidence, and also new results on the related issue of predicted grades, which it will be argued are likely to behave in a way that is similar to calculated grades.

Review of literature on predicted and forecasted grades

Predicted grades in university selection

A notable feature of UK universities is that selection mostly takes place before A-levels or equivalent qualifications have been sat, so offers are largely conditional on later attained grades. As a result, UCAS application forms, since their inception in 1964, have included *predicted grades*, estimates by teachers of the A-level grades a student is likely to achieve. Admissions tutors also use other information in making conditional offers. A majority of applicants in England, applying in year 13 for university entry at age 18 will have taken GCSEs at age 16 in year 11, a few still take AS-levels in year 12, some students submit an EPQ (Extended Project Qualification), and UCAS forms also contain candidate statements and school references. Medical school applicants mostly also take admissions tests such as U(K)CAT or BMAT at the beginning of year 13, and many will take part in interviews or MMIs (multiple mini-interviews) (see https://www.medschools.ac.uk/studying-medicine/making-an-application/entry-requirements) .

Predicted grades have always been controversial. A House of Commons Briefing Paper in 2019 noted that the UK was unusual among developed countries in using predicted grades[e], and said that,

> “The use of predicted grades for university admissions has been questioned for a long time. Many critics argue that predicted grades should not be used for university entry because they are not sufficiently accurate and it has been suggested that disadvantaged students in particular lose out under this system.” [22] p.4

Others have suggested that as well as being “biased”, “predicting A-level grades is clearly an imprecise science” [23] (p.418). There have been repeated suggestions over the years, none as yet successful, that predicted grades should be replaced with a PQA (Post-Qualification Applications) system. As Nick Hillman puts it,

> “The oddity of our system is not so much that people apply before receiving their results; the oddity is that huge weight is put on predicted grades, which are notoriously unreliable. … PQA could tackle this…”[f].

[e] https://www.bbc.co.uk/news/education-44525719
[f] https://www.hepi.ac.uk/2019/08/14/pqa-just-what-does-it-mean/

For peer review only - http://bmjopen.bmj.com/site/about/guidelines.xhtml

The system of predicted grades is indeed odd, but also odd is the sparsity of academic research into predicted grades. The most important question that seems almost never to have been asked, and certainly not answered, is the fundamental one of whether it is predicted grades or actual grades which are better at predicting outcomes. Petch[3] considers that predicted and actual grades may be fundamentally different, perhaps being "complementary and not contradictory" (p.29), one being about scholarly attitude and the other about examination prowess, primarily because "the school knows the candidate as a pupil, knowledge not available to the examiners". For Petch, either a zero correlation or a perfect correlation between predicted and actual grades would be problematic, the latter perhaps implying that actual grades might be seen as redundant (p.6).

The advent of Ofqual's calculated grades, which are in effect predicted grades carried out in a slightly different way, means there is now an urgent need to know how effective predicted grades are likely to be as a substitute for attained A-level grades. Are they in fact 'notoriously unreliable', being 'mere predictions', or do they have equivalent predictive validity as attained grades?

The research literature on predicted grades

As part of the Supplementary Information to this paper we have included a discursive overview of research studies on predicted grades. Here we will merely provide a brief set of comments.

Most studies look at predictions at the level of individual exam subjects, which are graded from E to either A or, from 2010 onwards, A*. The most informative data show all combinations of predicted grades against attained grades, and Figure 1 gives an example for medical school applicants. Many commentators, though, look only at over-predictions ('optimistic') and under-predictions ('pessimistic'). Figure 2 summarises data from five studies of university applicants. Accurate predictions occur in 52% of cases when A is the maximum grade and 17% when A* is the maximum grade (and of course with more categories accuracy is likely to be lower). Grades are mostly over-predicted, in 42% of cases pre-2010 and 73% post-2010, with under-prediction rarer at 7% of cases pre-2010 and 10% post-2010. A number of studies have reported that under-prediction is more common in lower socio-economic groups, non-White applicants, and applicants from state school or further education [24-26]. A statistical issue means such differences are not easy to interpret, as a student predicted A* cannot be under-estimated, and therefore under-estimation will inevitably be more frequent in groups with lower overall levels of attainment.

Some studies also consider grade-point predictions, the sum of grade scores for the three best attaining subjects, scored A*=12, A=10, B=8, etc[g].. In particular a large study by UCAS [27] showed that applicants missing their predictions (i.e. they were over-predicted) tended to have lower predicted grades, lower GCSE attainment, were more likely to have taken physics, chemistry, biology and psychology, and were from disadvantaged areas. To some extent the same statistical problems of interpretation apply as with analysis at the level of individual exam subjects. For a number of years UCAS calculated grade-point predictions, and they are included in the P51 data described later.

What are predicted grades and how are they made?

UCAS says that "A predicted grade is the grade of qualification an applicant's school or college believes they're likely to achieve in positive circumstances."[h] Later though, the document says predicted grades should be, "**in the best interests of applicants** – fulfilment and success at college or

---

[g] In some studies a scoring of A*=6, A=5, B=4 is used. The 12,10,8... scoring was introduced so that AS levels, weighted at half an A-level, could be scored as A=5, B=4 etc (there being no A* grade at AS-level). For most purposes A*=12, A=10 … is equivalent in all respects to A*=6, A=5, etc, apart from a scaling factor.
[h] https://www.ucas.com/advisers/managing-applications/predicted-grades-what-you-need-know [Accessed 13th April 2020].

For peer review only - http://bmjopen.bmj.com/site/about/guidelines.xhtml

university is the end goal ", and "**aspirational but achievable** – stretching predicted grades are motivational for students, unattainable predicted grades are not" (all emphases in original). Predicted grades should be professional judgements and be data-driven, including the use of, "past Level 2 and Level 3 performance, and/or internal examinations to inform …predictions".

Few empirical studies have asked how teachers estimate grades, with little major progress since 1964 when Petch said, "Little seems to be known about measures taken by schools to standardize evaluations of pupils" [3] (p.7). Two important exceptions are the studies of Child and Wilson[i] in 2015 and Gill [28] in May 2018, with only the latter published, Gill sending questionnaires to selected OCR Board exam centres concerning Chemistry, English Literature and Psychology exams. Teachers said the most important information used in predicting grades was performance in mock exams, observations of quality of work and commitment, oral presentation, the opinion of other teachers in the same subject, other subjects, and the head of department. Some teachers raised concerns about the lack of high stakes for mock exams which meant that students did not treat them seriously. AS-level grades were an important aid in making predictions. There were also concerns about the loss of AS-levels to help in prediction, as also mentioned elsewhere [29], and that is relevant to 2020 where most candidates will not have taken AS-levels.

Predicted grades in other Key Stage 5 qualifications than A-levels

Almost all studies on predicted grades have considered A-levels, with a few occasional exceptions looking at GCSEs. We know of no studies on the Extended Project Qualification (EPQ) in England, of Scottish Highers and Advanced Highers, or any other qualifications. The Supplementary Information to this report includes data on both EPQ and SQA examinations.

Forecasted grades

Until 2015, teachers in the May of Year 13 provided awarding organisations with *forecasted grade*, and those forecasts in part contributed to quality control of grades by the boards. Since forecasted grades are produced five to seven months after predicted grades, and closer to the exam date, they might be expected to be more accurate than predicted grades, being based on better and more recent information. Forecasted grades are important as they are more similar than predicted grades to the proposed calculated grades in the way they are calculated, and it is noted that "they may differ somewhat from the predicted grades sent to UCAS as part of the university application process" [30]. Three formal analyses are available, for candidates in 2009 [31], 2012 [32] and 2014 [30], and four other studies from 1940 [33], 1963 [3], 1977 [35] and 2018 [28] are available, with one post-2000 study before A* grades were introduced and three after (Figure 2). Petch [33] also provides a very early description of forecasted grades, looking at teachers' predictions of pass or fail in School Certificate examinations in 1940, which also show clear over-prediction.

Forecasted A-level grades are similar in accuracy to predicted grades pre-2010 (42% vs 52%) but are more accurate post-2010 (47% vs 17%), in part due to a large drop in accuracy of later predicted grades. Despite there being *no aspirational or motivational reasons for over-prediction of forecasted grades*, particularly in the 1977 and 2018 studies, over-prediction nevertheless remains as frequent as in predicted grades (pre-2010: 39%; post-2010: 37%) and remains more common than under-prediction (pre-2010: 20%; post-2010 16%). Overall it is possible that calculated grades may be somewhat more accurate than predicted grades, if forecasted grades are taken as a model for calculated grades. Two set of forecasted grades are also available for GCSEs [35,36], and they show

---

[i] Child, S., & Wilson, F. (2015). An investigation of A level teachers' methods when estimating student grades. Cambridge Assessment internal report. Cambridge, UK: Cambridge Assessment

For peer review only - http://bmjopen.bmj.com/site/about/guidelines.xhtml

similar proportion of over and under-prediction as do results for A-levels. Over-prediction seems to be a feature of all predictions by teachers.

The three non-official studies of forecasted grades also asked teachers to rank-order candidates, a procedure which will also be included in calculated grades. The 1963 data[3] found a median correlation of rankings and exam marks within schools of 0.78, the 1977 data[35] a correlation of 0.66 [35], and the recent 2018 data [28] a correlation of about .82. The three estimates, mean r = 0.75, are somewhat higher than a meta-analytic estimate of .63 (SE =.03) for teachers' ability to predict academic achievement [36].

The Gill study [28] is also of interest as one teacher commented on the difficulty of providing rankings with 260 students sitting one exam, and the author commented that, "it was easier for smaller centres to make predictions because they know individual students better" (p.42), with it also being the case that responses to the questionnaire were more likely to come from smaller centres. The 1963 study of Petch[3] , as well as commenting on "considerable divergencies … in the methods by which estimates were produced" (p.27), as in the variable emphasis put on mock exams, also adds that, "some of the comments from schools suggested that at times there may be a moral ingredient lurking about some of the estimates"(p.28).

The studies of forecasted grades suggest that there is some possibility that calculated grades may be somewhat more accurate than predicted grades, but they also indicate the problems shown by teachers in trying to rank candidates. It also remains possible that examining boards have far more extensive and unpublished data on forecasted grades that they intend to use in assessing the likely effectiveness of calculated grades.

Applicants to medical school

So far, this paper has been entirely about university applicants across all subjects and the entire range of A-level grades. Only a handful of studies have looked at predicted grades in medical school applicants.

Lumb and Vail pointed out the importance of predicted grades since they determine in large part how shortlisting takes place [38]. In a study of 1995 applicants they found 52% of predictions were accurate, 41% were over-estimated and 7% under-estimated [38], values very similar to those reported in university selection in general (Figure 2).

A study by one of the present team used path modelling to assess the causal inter-relationships of GCSE grades, predicted grades, receipt of an offer, attained A-level grades, and acceptance at medical school [39]. Predicted grades were related to GCSE grades (beta=0.89), and attained A-level grades were predicted by both GCSE grades (beta=0.44) and predicted A-level grades (beta=0.74). The study supports claims that teachers may well be using GCSE grades in part to provide predicted grades, which is perhaps not unreasonable given the clear correlation.

Richardson et al [40] in a very important and seemingly unique study looked at the relative predictive validity of predicted as compared with attained A-level grades. Using a composite outcome of pre-clinical performance they found a minimal correlation with predicted grades (r=.024) compared with a correlation of 0.318 (p<.001) with attained A-level grades. To our knowledge this is the only study of any sort assessing the predictive validity of predicted vs attained A-level grades.

The present study

Although calculated grades are novel and untested in their details, predicted grades have been around for half a century, and there is also a literature on forecasted grades. This paper will try to answer several empirical questions about predicted grades, for which data are now available in

For peer review only - http://bmjopen.bmj.com/site/about/guidelines.xhtml

UKMED. Predicted grades will then be used, *faute de mieux*, to make inferences about the likely consequence of using calculated grades.

Empirical questions to be addressed.

The relationship between predicted and attained grades in medical school applicants

Few previous studies have looked in detail at this high-performing group of students. We will also provide brief results on Scottish Highers and Advanced Highers, and the EPQ (Extended Project Qualification), neither of which has been discussed anywhere else to our knowledge.

The predictive validity of predicted grades in comparison with attained grades

A fundamental question concerning calculated grades is whether teacher estimated grades are better or worse at predicting outcomes than are actual A-level grades. The relationship between predicted grades and actual grades cannot answer that question. Instead what matters is how well predicted and actual grades predict subsequent outcomes at the end of undergraduate. The only relatively small study on this of which we are aware in medical students [40] found that only actual grades had predictive validity.

Method

The method provided here is brief. A fuller description can be found in the Supplementary Information. Overall the project is **UKMEDP112**, approved by the UKMED Research Group in May 2020, with data coming from two separate but related UKMED projects, both of which include predicted grades.

Project **UKMEDP089**, “The UK Medical Applicant Cohort Study: Applications and Outcomes Study”, approved Dec 7th, 2018, with Dr Katherine Woolf as principal investigator, is an ongoing analysis of medical student selection as a part of UKMACS (UK Medical Applicant Cohort Study[j]). The data upload of 21st Jan 2020 included detailed information from UCAS and HESA on applicants for medicine from 2007 to 2018.

Project **UKMEDP051**, “A comparison of the properties of BMAT, GAMSAT and UKCAT”, approved Sept 25th, 2017, with Dr Paul Tiffin as principal investigator, is an ongoing analysis of the predictive validity of admissions tests and other selection methods such as A-levels and GCSEs in relation to undergraduate and postgraduate attainment. UCAS data are included, although at the time of the present analysis, 16th April 2020, this had not yet included the detailed subject level information available in UKMEDP089[k]. Outcome data for the P51 dataset are extensive, and in particular undergraduate progression data are included, such as UKFPO EPM and SJT, and PSA (Prescribing Safety Assessment).

Data from HESA are required to be reported using their rounding and suppression criteria (https://www.hesa.ac.uk/about/regulation/data-protection/rounding-and-suppression-anonymise-statistics) and those criteria have been used for all UKMED data. In particular the presence of a zero or a zero percentage may not always mean that there are no individuals in a cell of a table, and all integers are rounded to the nearest 5.

---

[j] https://ukmacs.wordpress.com/

[k] An upload for P51 dated 20th April 2020 is now available but pre-processing is required before the data can be analysed properly.

For peer review only - http://bmjopen.bmj.com/site/about/guidelines.xhtml

Results

As with the Method section, a fuller description of the results can be found in the Supplementary Information.

The relationships between predicted and actual grades in medical school applicants.

Predicted and actual A-level grades for individual A-level examinations

Figure 1 shows the relationship between predicted and attained A-level grades for 237,030 examinations from 2010 to 2018 (i.e. including A* grades). 39.3% of predicted grades are A* compared with 23.7% of attained grades. Figure 1.a shows predicted grades in relation to attained grades, with bold font for accurate predictions, green and blue shading for under-prediction and orange and red shading for over-prediction. Overall 48.8% of predicted grades are accurate, which is higher than for university applications in general (see Figure 2), reflecting the high proportion of A and A* grades (69%). Over-prediction occurred in 44.7% of cases, and under-prediction in 6.5% of cases. Figure 1.b show the data as percentages. About a half of A* predictions result in an attained A grade, and over a third of predicted A grades result in grade B or lower. Despite over-prediction by about half an A-level grade, predicted and attained grades have a Pearson correlation of r = 0.63.

Differences between A-level subjects

There is little in the literature on the extent to which different A-level subjects may differ in the accuracy of their predictions, perhaps with different degrees of bias or correlation. Detailed results are presented in the Supplementary Information. Overall, Biology, Chemistry, Maths and Physics are very similar in terms of over-prediction and correlation with actual grades. However General Studies is particularly over-estimated compared with other subjects.

Extended Project Qualification (EPQ) and SQA Advanced Highers

The Supplementary Information contains information on these qualifications. SQA Advanced Highers, as well as the EPQ, show similar proportions of over-estimation as other qualifications (see Figure 2).

Reliability of predicted and attained A-level grades

Considering the best three A-level grades, the reliability of an overall score can be calculated from the correlations of the individual subjects. For 66,006 candidates with at least three paired predicted and actual grades, Cronbach's alpha was 0.827 for actual grades and 0.786 for predicted grades, with a highly significant difference. The difference may in part reflect the higher proportion of A* grades in predicted than actual grades, but may also reflect greater measurement precision in the marking of actual A-levels.

How reliable are attained A-level grades?

Attained A-level grades, like any behavioural measurement are not perfectly reliable, in the sense that if a candidate took a parallel test containing equivalent but different items it is highly unlikely that they would get exactly the same mark as on the first attempt. They may, for instance, have been lucky (or unlucky) at their first attempt, having questions which they happened to have studied

For peer review only

or revised more (or revised less), and so on. Reliability is a technical subject[l] with many different approaches [41,42]. For continuous measures of raw scores, the reliability can be expressed as a coefficient such as alpha (and in one A-level maths test in 2011, alpha for the full test was about 0.97 [43], although it is suggested that value is unusually high). Boards though do not report raw scores, but instead award grades on a scale such as A* to E. The 'classification accuracy' of grades is harder to estimate, and is greater with fewer grade points, wider grade intervals, and a wide spread of candidate ability [43]. There seem to be few published estimates of classification accuracy for A-levels (although they do exist for GCSEs and AS-levels [43]).

Estimating classification accuracy for the present high-attaining group of medical school applicants is not easy. A fundamental limit for any applicants is that predicted grades cannot possibly predict actual grades better than attained grades predict themselves (the reliability or classification accuracy). However, from considering the correlation of the three best predicted and actual grades it is unlikely that such a limit has currently been reached. The correlation of actual with predicted grades is .585 (see figure 3), and the alpha reliabilities of .827 for actual grades and .786 for predicted grades (see above). The disattenuated correlation between predicted and actual grades is therefore .585/(√(.827x.786) = 0.726, which is substantially less than one, with predicted grades accounting for only about a half of the true variance present in actual grades. If the disattenuated correlation were close to one then it could be argued that predicted grades were doing as well as they could possibly do given that attained grades are not perfectly reliable, but that is clearly far from the case.

True scores and actual scores

From a theoretical, psychometric, point of view it could be argued that it is neither actual nor predicted grades which need to be estimated for applicants, but their 'true ability scores', or the 'latent scores', to use the technical expressions, of which predicted and actual grades are but imperfect estimates. In an ideal world that would be the case, and a well-constructed exam tries to get as close as possible to true scores. However, it is not possible to know true scores (and if it were the boards would provide selectors with those scores). Selection itself does not work on true scores but on the actual grades that are written down, by teachers for predicted grades, and as grades on exam result certificates by boards. They are the currency in which transactions are conducted during selection, so that a predicted grade of less than a certain level means a candidate will not get a conditional offer, and likewise too low an actual grade means a candidate holding a conditional offer will be rejected. For that reason it is not strictly the correlation of predicted and actual grades which matters, the two measures being treated as symmetric, but the forward prediction of actual grades from predicted grades, i.e. the actual grades conditional on the predicted grades (as shown in figure 1b).

Predictive validity of predicted and attained A-level grades in medical students.

Predictive validity in UKMEDP051

The version of the P51 data used here consists entirely of applicants applying to medical schools, but there is also follow-up into undergraduate and postgraduate training. Predicted A-level grades were available only for the UCAS application cycles of 2010 to 2014, and consisted of a single score in the range 4 to 36 points, based on the three highest predictions scored as A*=12, A=10, etc.. The modal

[l] See https://www.gov.uk/government/publications/reliability-of-assessment-compendium for a range of important papers commissioned and published by Ofqual.

For peer review only - http://bmjopen.bmj.com/site/about/guidelines.xhtml

score for 38,965 applicants was 30 (equivalent to AAA; mean=31.17; SD= 3.58; Median = 32; 5th, 25th, 75th and 95th percentiles= 26, 30, 34 and 36). For simplicity the study was restricted to applicants aged 18 in the year of application, who had both predicted and attained A-levels, which also ensured the sample contained only first applications for non-graduate courses, from candidates who had not taken pre-2010 A-levels. Overall, 22,955 applicants were studied. Other selection measures included were GCSEs (mean grade for best eight grades), as well as U(K)CAT and BMAT scores, based on the most recent attempt which for most of these cases was also the first attempt. For simplicity we used the total of the four sub-scores of U(K)CAT, and the total of Section 1 and 2 scores for BMAT.

Follow-up is complicated as application cohorts enter medical school in different years, and spread out in time through medical school. For this UKMED data extract, applicants in 2010 entered the medical register from 2015-18, 2011 applicants in 2016-8, 2012 applicants in 2017-18 and 2013 applicants in 2018. Applicants in 2014 would only qualify in 2019 or after, and the UKMED dataset did not include 2019 data. Some 2010 to 2013 applicants will also qualify after 2018. Again for simplicity, undergraduate outcome measures were restricted to the deciles of the UKFPO's Educational Performance Measure (EPM), the raw score of the UKFPO's Situational Judgement Test (SJT), and the score relative to the pass mark of the Prescribing Safety Assessment (PSA), all at first attempt. Relatively few doctors, mostly from the earlier cohorts, had progressed through to postgraduate assessments, but sufficient numbers for analysis were present for MRCP(UK) Part 1 and MRCS Part A, scores being analysed at the first attempt.

EPM, is a complicated measure summarising academic progression through medical school, with individual schools deciding what measures to include [44], and expressed as deciles *within* each school and graduating cohort year. EPM is here used as the main undergraduate outcome measure. Deciles are confusing, as UKFPO scores them in the reverse of the usual order, the first decile being highest performance and the tenth the lowest[m] (see Figure 5). Here for ease of interpretation we reverse the scoring in what we call *revDecile*, so that higher *revDeciles* indicate higher performance. It should also be remembered that deciles are not an equal interval scale.

Correlations between the measures are summarised in Figure 3. Large differences in Ns reflect some measures being used in applicants during *selection*, and others being outcome measures that are only present in *entrants*, as well as the smaller numbers of doctors who had progressed to postgraduate assessments. The distinction is emphasised by dividing the correlation matrix into three separate parts. Correlations of selection and outcome measures necessarily show *range restriction* because candidates have been selected on the basis of the selection measures, and likewise doctors taking postgraduate examinations may be self-selected for earlier examination performance.

Figure 3 contains much of interest (see Supplementary Information), but the most important question for present purposes is the extent to which Predicted and Attained A-level grades (shown in pink and green in Figure 3) differ in their prediction of the five outcome measures, remembering that undergraduate outcomes are typically five or six years after selection, and postgraduate outcomes are seven or eight years after selection.

Attained A-levels predict EPM with r=0.297 compared with a correlation of only 0.198 for predicted grades. N is large for these correlations and hence the difference, using a test for correlated correlations [45] is highly significant (Z=12.6, $p<10^{-33}$). These raw correlations are inevitably range restricted, and the construct level predictive validity accounting for range restriction and

---

[m] https://foundationprogramme.nhs.uk/wp-content/uploads/sites/2/2019/11/UKFP-2020-EPM-Framework-Final-1.pdf,

For peer review only - http://bmjopen.bmj.com/site/about/guidelines.xhtml

measurement error is likely to be much higher [46]. Multiple regression (see Supplementary Information) suggests that predicted grades may have a small amount of predictive variance which is not shared with attained A-levels. Figure 4 shows mean EPM *revDecile* scores in relation to actual and predicted A-levels. The slope of the line is clearly less for predicted A-levels, showing a less good prediction. It is also clear that attained grades predict well, A*A*A* entrants scoring an average of two deciles higher at the end of the course than those with AAA grades, each extra grade raising average performance by about two-thirds of a decile. In contrast the slope is less for predicted grades, being slightly less than half a decile per predicted A-level grade.

The broad pattern of results is similar for the other undergraduate outcomes, SJT and PSA, and is available in the Supplementary Information.

The two postgraduate outcome measures, MRCP(UK) Part 1 (Membership of the Royal Colleges of Physicians (UK) examination) Part 1, and MRCS (Membership of the Royal College of Surgeons) Part A , although both based on smaller numbers of doctors, are still significant, actual grades correlating more highly with MRCP(UK) Part 1 (r=.421) than do predicted grades (r=.283; Z= 4.54, p=.000055). Likewise, actual grades correlate more highly with MRCS Part A (r=.421) than do predicted grades (r=.358; Z= 3.67, p=.000238).

There are suggestions that predicted grades may not be equivalent in candidates from state schools and private schools, grades being predicted more accurately in independent schools [24,25]. That is looked at in the Supplementary Information, and while there is clear evidence, as found before in the UKCAT-12 study [47], that private school entrants underperform relative to expectations based on their A-levels, there is no evidence that predicted grades behave differently in candidates from private schools.

A practical question relevant to calculated grades concerns the extent to which, in the absence of attained A-level grades, other selection measures such as GCSEs, U(K)CAT and BMAT can replace the predictive variance of attained A-level grades. That will be considered for EPM where the sample sizes are large. Attained grades alone give R = 0.297, and predicted grades alone give R=.198, accounting for less than half as much outcome variance. Adding GCSEs to a regression model including just predicted grades increases R to .225, and also including U(K)CAT and BMAT increases R to .231, which is though substantially less than the .297 for attained A-levels alone. In the absence of attained A-level grades, prediction is therefore improved by including GCSEs and U(K)CAT or BMAT, although the prediction still falls short of that for actual A-levels alone.

Modelling the effect of only predicted grades being available for selection

In the context of the 2020 pandemic, an important question is the extent to which future outcomes may change as a result of selection being in terms of calculated grades. Calculated grades themselves cannot be known, but predicted grades are probably a reasonable surrogate for them in the first instance. A modelling exercise was therefore carried out whereby the numbers of students in the various EPM revDeciles were tabulated in relation to predicted grades at five grade levels, 36 pts ≡ A*A*A*, 34 pts ≡ A*A*A, 32 pts ≡ A*AA, 30 pts ≡ AAA and ≤ 28 pts ≡ ≤ AAB, the probability of each decile found for each predicted A-level band. Assuming that selection results in the usual numbers of entrants with grades of A*A*A*, A*A*A, etc, but based on calculated grades rather than actual grades, the expected numbers of students in the various EPM deciles can be found. Figure 5 shows deciles as standard UKFPO deciles (1 = highest), UKFPO scores (43 = highest), and *RevDeciles*, (10 = highest). The blue column shows the actual proportions in the deciles based on attained A-level grades. Note that for various reasons there are not exactly equal proportions in the ten

For peer review only - http://bmjopen.bmj.com/site/about/guidelines.xhtml

deciles[n]. Based on selection on attained A-level grades there are 7.2% of students in the lowest performing decile, compared with an expected proportion of 8.1% for selection on predicted grades, an increase of 0.9% percentage points, which is a relative increase of 13.0% in the proportion of the lowest decile, with an odds ratio of 1.141 of attaining the lowest decile. For the highest scoring decile, the proportion decreases from 10.1% with actual A-level grades to 8.8% if predicted A-level grades are used, an absolute decrease of 1.4%, and a relative decrease of 13.4% of top deciles, with an odds ratio of 0.853.

Of course the above calculations are based on the assumption that the 'deciles' for calculated grades are expressed at the same standard as currently. Were the outcomes to be re-standardised so that all deciles were equally represented then of course at finals no noticeable difference would be present[o]. However the academic backbone would still be present, and overall poorer performance on statistically equated postgraduate exams would be expected.

Discussion

The present data make clear that only slightly under half of predicted grades are accurate, with 45% being higher than attained grades, and only 17% being lower. The data also show that attained grades are far better predictors of medical school performance than are predicted grades, which account for only about a third as much outcome variance as attained grades. Attained grades are also more reliable than predicted grades.

Validation is the bottom line of all educational assessment, and in the present case it is validation against assessment five to eight years down the line from the original A-levels, in both undergraduate and postgraduate assessments. That is strong support for what we have called 'the academic backbone', prior attainment providing the underpinning for later attainment, and hence performance at assessments at all stages of training for GCSEs through to medical degrees (and probably on to postgraduate assessments) tend to be correlated [19]. The findings contradict suggestions that holistic judgments by teachers of predicted grades are better predictors of outcomes since teachers may know their students better than examiners. The immense efforts by exam boards and large numbers of trained markers to refine educational measurements is therefore gratifying and reassuring. Careful measurement does matter.

A remaining question is whether there is still some variance in predicted and actual grades which is complementary. A tiny amount, is the answer, predicted grades increasing R by about 0.05 when actual grades are already taken into account. What that variance might be is unclear, and it is possible that it is what Petch called 'scholarly attitude'. At present though it is worth remembering that *examination* grades at A-level are primarily predicting further *examination* grades at the end of medical school, although EPM scores do include some measures of course work, and practical and clinical skills. If other outcome measures, perhaps to do with communication, caring or other non-cognitive skills were available then predicted grades might show a greater predictive value.

The present data inevitably have some limitations, but they are few. There is little likelihood of bias since complete population samples have been considered, and there is good statistical power with large sample sizes. Inevitably not all outcomes can be considered, mainly because the cohorts

---

[n] In part this reflects the fact that some students, particularly weak ones, are given an EPM score, but then fail finals.

[o] This is based on deciles being calculated in a way that are equated to levels used in the present calculation. Of course if calculated strictly as deciles then of necessity 10% will still remain in the top decile, etc.. That difficulty of deciles will in large part be removed when the 2020 entrants graduate as UKMLA should be on stream by then.

For peer review only - http://bmjopen.bmj.com/site/about/guidelines.xhtml

analysed have not yet progressed sufficiently through postgraduate training. However those who are included show effects which are highly significant statistically.

Our questions about predicted grades have been asked in the practical context of the cancellation of A-level assessments and their replacement by calculated grades, as a result of the COVID-19 pandemic. It seems reasonable to assume, given the literature on predicted grades, and particularly on forecasted grades, that calculated grades will probably have similar predictive ability to predicted grades, but perhaps be a little more effective due to occurring later in the academic cycle. Such a conclusion would be on firmer ground if exam boards had analysed the predictive validity of the data they had collected on forecasted grades, particularly in comparison with predicted and actual grades. Such data may exist, and if so then they need to be seen. In their absence, the present data may be the best available guesstimates of the likely predictive validity of calculated rather than actual grades.

A further consideration is more general and asks what the broader effects of the COVID-19 pandemic may be on education. Students at all levels of education have had teaching and learning disrupted, often extensively, and that is also true of all stages of medical education. The current cohort of applicants will not be assessed formally at A-level. As well as meaning that they may only have calculated grades, which are likely to be less accurate, they also will have missed out on several other things. Year 13 students may have missed normal schooling from, say, mid-March until mid-May, when they should have taken public examinations, perhaps 30 to 40 school days. Year 12 students, from whom 2021 medical school entrants will be drawn, may miss school from mid-March until mid-July, totalling perhaps 80 school days. Burgess and Sievertsen [48], using data from two studies [50,51], estimate that 60 lost school days results in a reduction in performance of about 6% of a standard deviation, which they say is, “non-trivial”. These effects are likely to differ also by socio-economic background, particularly in effectiveness of home schooling. Applicants not taking A-levels will also suffer from the loss of the enhanced learning that occurs when learners are tested – the ‘testing effect’ – for which a meta-analysis found an effect size of 0.50 [51], which is also non-trivial. Taken overall, 2020 entrants to medical school, and perhaps those in 2021 as well, may perform less well as a result of missing out both on education and on its assessment.

What then are the specific implications of our findings for the use of calculated grades, for medical education and the selection of medical students? Whether our findings will generalise to other university courses is far from clear, not least because very few have the sophisticated undergraduate and postgraduate outcome measures found in medical education, which inevitably will make effects harder to demonstrate, although in principle the effects should still be there.

For universities and medical schools.

Medical schools are skilled in understanding the various data they hold on applicants, and how results ought to look given prior attainment, and they recognise that some sets of results might be skewed, sometimes because of small group sizes. They also recognise that predicted grades often err on the side of generosity. No doubt they will be looking at calculated grades to check whether they are compatible with other data. Nevertheless, calculated grades are largely uncharted territory even for experienced university admissions officers, with many potential uncertainties, particularly in relation to predicting future medical school performance.

A useful thought-experiment is to imagine what would happen were a computer-error accidentally to shuffle all A-level grades, but with the same numbers of A*, A, B grades, etc still being awarded for individual subjects, but without any relation to the grades that candidates should have attained. Administratively, selection would proceed as normal. However instead of medical schools admitting high-achieving entrants with A* and A grades, entrants would have what we might call nominal ‘A*’

For peer review only - http://bmjopen.bmj.com/site/about/guidelines.xhtml

and 'A' grades, but in reality would have had true grades anywhere from A* to E, with a mode at about B. Since A-levels undoubtedly predict university outcomes, many entrants with lower true grades would therefore probably struggle with course content and exams. For medical schools using criterion-referencing, more students than usual would fail exams. If norm-referencing were used, the usual proportion of students would pass but pass marks would be lower, students would know less at graduation, and the knowledge deficit would then become clear when taking statistically equated postgraduate examinations such as MRCP(UK).

Attained grades within candidates in different A-level subjects normally tend to correlate, so that the reliability of a total points score can be calculated (see above). Grades allocated at random would not however correlate within candidates. Were a medical school to select on total A-level points (say, with a threshold equivalent to AAA) there would still be applicants with that number of points. However because random allocation results in zero correlations of grades within candidates, the variance of total points would be less and candidates with high point scores would be in shorter supply.

Although only a thought-experiment, calculated grades will probably have a partial similarity to randomly allocated grades (and the reliability of predicted grades is indeed less than that of attained grades, because correlations are lower) in that measurement error will have increased. Different subjects will correlate less than usual, and there may be a shortage of qualified candidates, and nominal grades will correlate less with outcomes than usual, regression to the mean making outcomes somewhat poorer than usual. The predictions therefore are qualitatively similar to the pattern of those with random allocation, but less extreme.

A practical question is whether medical schools can do anything to ameliorate possible problems with calculated grades. For those holding conditional offers, a strong argument says there is a contractual agreement between a university and a candidate holding a conditional offer based on certain A-level grades (although a counter-argument might be that the contract implicitly said that payment would be in actual, gold-standard, attained grades, and not in the potentially baser currency of promissory notes based on calculated grades). If indeed there were to be too many candidates meeting their offers, then the precise nature of the contract could become crucial. For candidates not meeting the grades stated in conditional offers, so that there are unfilled places, there is no obvious contractual agreement, and medical schools may wish then to take into account GCSEs, U(K)CAT and BMAT scores, and perhaps other measures such as EPQ and MMI, although the latter two have not yet been validated as predictors of outcome. The use of some sorts of information, such as personal statements and work experience, may however favour more advantaged students [4], although medical schools may wish also to take educational disadvantage into account to widen participation and because applicants who do relatively better than their peers at poorly-performing schools tend to achieve higher grades in medical school than those with equivalent grades from high-performing schools [52]. Ultimately, universities will need to be, "flexible and responsive in their admissions processes" [53].

Whatever medical schools do choose to do (or not to do) the one certainty is that outcomes for the 2020 entry cohort will be followed with interest through their careers, and UKMED will be a major tool in doing that.[p]

[p] In the event, offers were honoured and the cap on medical school numbers was lifted to accommodate the extra students who met their conditional offers when the calculated grades algorithm was scrapped in August 2020.

For peer review only - http://bmjopen.bmj.com/site/about/guidelines.xhtml

For teachers

Centre assessment grades and rankings may well be problematic for teachers (and recent research suggests that rankings are hard to make for large numbers of candidates [28], and that different teachers use different sorts of information in making their judgements). Since the intention of calculated grades is that they will emulate the actual grades that students would have attained had they taken the exams, then a key emphasis probably has to be on what Petch called "anticipated examination prowess" rather than "scholarly attitude". How easy it is to do that though is very unclear (and a way to help may be to ask teachers to make both assessments on students, so that differences become clearer). Many teachers will already have provided predicted grades for UCAS applications earlier in the academic year, and whether it is entirely possible to forget those earlier estimates seems unlikely, particularly when much but not all of the information will be the same as earlier. The possibility of parental challenge when centre assessment grades differ from earlier predicted grades may also make the two sets of grades more similar. Cognitive dissonance may additionally make it particularly difficult to forget earlier predictions, although it might be worth remembering the saying often attributed to John Maynard Keynes: "When events change, I change my mind. What do you do?"[q]. Finally, from a research point of view it would be nice to think that information will be collected about how teachers and schools in practice are producing their centre assessment grades, although it seems possible that may not occur due to fears of legal challenge.

For medical school applicants

Cancellation of A-levels inevitably has produced uncertainty and anxiety for university applicants. Calculated grades have introduced what is to some extent an entirely unknown factor, although on balance it is likely that calculated grades will probably relate closely to predicted grades.

If calculated grades are similar to predicted grades, and given that much selection has in fact already taken place during shortlisting largely, but not entirely, on the basis of predicted grades (and other information such as admissions tests and interviews), then selection this year will mainly be on predicted grades. A particular problem normally is applicants for whom there is under-prediction of grades, as calculated grades are likely also to be under-predicted, but without any actual grades to assess whether that is the case. For those whose grades in normal circumstance are under-predicted, being predicted, say, BBB, but achieving A*AA, there is usually a safety net for a second chance at a place, formally through Clearing or sometimes through other more informal processes such as in medical schools which maintain waiting lists. Quite how, beyond random chance, calculated grades might differ substantially from predicted grades, is unclear. That also means it is difficult to see how Clearing and similar processes will work in 2020. Under-prediction is a particular risk in cases where teachers do not know, or in some cases do not like, their students, perhaps because of attitude, personal characteristics, or other factors, the externality and objectivity of examinations traditionally solving the problem. Petch, once again, put it well, describing,

> "instances, where, in the examination room, candidates have convinced the examiners that they are capable of more than their schools said that they were … Paradoxical as it will seem, examiners are not always on the side of authority; an able rebel can find his wider scope within the so-called cramping confines of an examination." [3](p.29).

There is a clear echo here of the quote by Yasmin Hussein with which this paper began. Hussein's concerns are not alone, and the UKMACS study in April 2020 found concerns about fairness were particularly present in medical school applicants from non-selective schools, from Black Asian and

[q] The phrase was probably said in 1970 by Paul Samuelson, a Nobel-prize winning economist, although in 1978 he himself attributed it to Keynes. https://quoteinvestigator.com/2011/07/22/keynes-change-mind/

For peer review only - http://bmjopen.bmj.com/site/about/guidelines.xhtml

Minority Ethnic (BAME) applicants, from female applicants, and from those living in more deprived areas [6].

The question of whether predicted grades are more accurate in private sector schools than public sector, state schools is complex and is discussed in an appendix to the Supplementary Information. There are subtle ceiling effects and non-linearities, but overall the best conclusion seems to be that private sector predictions may seem to be more accurate but that mainly reflects the higher actual grades of students at selective schools – if a student is predicted an A* they cannot over-achieve and private school pupils tend to be less likely to have low grades (not least because they have been selected to have higher than average ability levels) meaning that teachers predicting high grades are more likely to be correct. That conclusion is also important as it means that although there is an argument that calculated grades may predict outcomes better than predicted grades because they will have been standardised, predicted grades in effect are already standardised in so far as teachers at selective schools provide higher predicted grades because those candidates in actually do gain higher attained A-level grades. Standardisation by previous school performance is unlikely to change that conclusion.

Conclusions

The events of 2020 as a result of the COVID-19 pandemic are extra-ordinary, and unprecedented situations have occurred, of which the cancellations of GCSE and A-level exam cancellations are but one example. The current study should not be seen as criticism of the response of *Ofqual* to that situation; given the circumstances in which it found itself, with examinations cancelled (when the Chair of Ofqual Roger Taylor had recommended socially-distanced or delayed exams), Ofqual's solution to the problems has many obvious virtues.

For most university applicants there already existed predicted grades from the previous autumn when UCAS applications were submitted, but they would have been on average half a grade or so too high, being aspirational as much as realistic, and also for medical students would have been made by October 2019, whereas calculated grades would be based on teacher predictions in May 2020, albeit with several months of courses missing since March 2020.

Raw predicted grades would have wrecked much university planning as numbers of acceptances would inevitably have been far too high. There was also a risk that predicted grades could have been systematically higher from some schools than others – the ones with a tendency to call all of their geese swans -- and that probably applies also to the centre assessment grades sent to examination boards and mostly eventually accepted without central standardisation in August 2020.

This paper has provided evidence that the grades awarded to medical applicants in summer 2020 will probably not predict future outcomes with the same effectiveness as actual, attained grades, and that is a problem that universities and medical schools and postgraduate deaneries will have to work with, probably for many years as the 2020 cohort works through the system, some eventually being part of the first cohort to take the new national Medical Licensing Examination in 2024/2025 (https://www.gmc-uk.org/education/medical-licensing-assessment/uk-students-guide-to-the-mla). It seems likely therefore, as Thomson has said, "… this year group will always be different…"[2].

For peer review only - http://bmjopen.bmj.com/site/about/guidelines.xhtml

Postscript 25th November 2020

Introduction

The research for the present paper was carried out in April and May 2020, in parallel with the study of attitudes and responses of medical school applicants to the cancellation of A-level examinations and their replacement by 'Calculated Grades'[r]. These Calculated Grades consisted of teacher-estimated Centre Assessment Grades (CAGs) which were submitted to *Ofqual* and moderated using an algorithm. Calculated grades were released on 13th August, but then fairly rapidly the algorithm was abandoned and grades reverted to the unmoderated, unadjusted, teacher-estimated CAGs (or the Calculated Grade, if higher). This process, and the fallout from it, is described below.

Key findings from this paper and the accompanying applicant attitudes paper were presented to UK medical admissions tutors at a meeting of the MSC-SA (Medical Schools Council Selection Alliance) on 6th May 2020, and drafts of the two papers distributed. Both papers were finalized in May 2020 and uploaded to *medRxiv* on June 5th 2020. Little further happened over the next few weeks, and the present paper is therefore a statement of how we understood the situation at that time, with a few amendments for clarity.

In July and August 2020 however, things moved rapidly, with dramatic changes taking place. It would have been extremely confusing and probably misleading to have tried to incorporate those changes into the text of the main paper. Instead we hope this postscript will give readers a sense of what happened, to what extent events were correctly or incorrectly predicted by us, what impact the present paper may have had, and what may be the implications for the future.

The story is best told chronologically and we mostly use reports from newspapers, and refer interested readers to a brief summary on Wikipedia[s] and a journalistic review on the BBC website[t].

The awarding of A-level grades in 2020: the story from March to November 2020

As described in our main paper, on 20th March 2020 public examinations in the UK including A-levels were cancelled. On 3rd April *Ofqual* announced that exam grades in England would be replaced with Calculated Grades. Calculated Grades were to consist of Centre Assessment Grades (also called Centre Assessed Grades or CAGs), estimated by teachers that centres (mostly schools and colleges) would submit to *Ofqual*. *Ofqual* would moderate these CAGs using an algorithm – the details of which had not yet been published but, it was stated, would be based on the prior performance of pupils within schools. The Scottish Qualification Authority (SQA), Qualifications Wales, and the Northern Irish Council for Curriculum, Examination and Assessment (CCEA) also announced that they would use a broadly similar approach to that of *Ofqual*.

Schools (centres) had to return their teacher-estimated CAGs to *Ofqual* in June 2020. On June 16th a report in The Times said that "Teachers have marked too generously in allocating GCSE and A-level grades this year, research suggests" (The Times, 16th June), the article being based on a report from

---

[r] Woolf, K., Harrison, D., McManus, I.C. "The attitudes, perceptions and experiences of medical school applicants following the closure of schools and cancellation of public examinations in 2020 due to the COVID-19 pandemic", *medRxiv*, https://doi.org/10.1101/2020.06.02.20116855

[s] https://en.wikipedia.org/wiki/2020_UK_GCSE_and_A-Level_grading_controversy

[t] Coughlan,S. "Coronavirus: The story of the big U-turn of the summer", https://www.bbc.co.uk/news/education-54103612

For peer review only - http://bmjopen.bmj.com/site/about/guidelines.xhtml

FFT Education Datalab, which actually had only asked about GCSEs, and had no data on A-levels[u] . In July a Guardian article reported a statement from *Ofqual* that “a substantial number of students would receive at least one adjusted grade – usually downwards – as a result of a standardisation process” although they “sought to allay fears that certain groups of pupils, … could be disadvantaged by calculated grades. *Ofqual* said their analysis had found no evidence of widening of gaps in attainment”.(*The Guardian (G),* 21st July).

SQA results in Scotland are announced a week before those in England, so the SQA results announced on August 5th 2020 gave a preview of what was to come the following week in the rest of the UK. The Scottish results were immediately controversial when it emerged that the moderation of teacher-estimated grades (CAGs) by an algorithm had resulted in a quarter of grades being adjusted downwards. The Scottish Education Secretary, John Swinney, said that without those adjustments the pass rates would be up on the previous year by 14% for Highers and 13.4% for Advanced Highers. He added that, “… these robust processes mean we have upheld standards… All exam systems rely on an essential process known as moderation to uphold standards. This ensures an A grade is the same in every part of the country, making the system fair for everyone, and across all years.” *(G*, 4th August).

Teachers, students, parents and the media were unhappy with the moderation. A *Daily Telegraph* editorial entitled “Exam moderation is a gross injustice” attacked statistical modelling in general, and the SQA process in particular, which “gives poorer marks to children living in deprived areas … [without] recognition of individuals who buck the general trend” (*Daily Telegraph (DT)*, 5th Aug), and asked “Is the same fiasco about to be inflicted on A-level students in England and Wales?”. By 11th August, students in Scotland were protesting on the streets, Nicola Sturgeon, the First Minister, was apologising for the exam results debacle, the Scottish government was facing a vote of no confidence (*G*, 11th Aug). Expectations in the media of problems with A-levels were also growing. On 12th August it was announced in Scotland that the teacher-estimated CAGs downgraded by the SQA algorithm during moderation “would be reinstated” (*G*, 12th Aug).

In an attempt to prevent problems with A-levels, on August 12th 2020 the English education secretary, Gavin Williamson announced “a triple lock” for A-level students, whereby students could accept their Calculated Grade results, use the results of mock exams (practice exams which students take in schools), or use the results of real exams due to take place in Autumn 2020 after the start of the university academic year (*The Times*, (T), 12th August; *T*, 13th August). Protests were immediate as mock exams vary immensely, and many schools had been encouraged to cancel mock exams as a part of the Covid lockdown in March 2020.

A-level Calculated Grades (i.e. the teacher-estimated CAGs adjusted by the algorithm during moderation) were announced on 13th August 2020, and UCAS announced which students had obtained places at their chosen university based on these Calculated Grades. An immediate problem arose: following the Scottish government’s reversal students in Scotland now had unadjusted SQA grades, which were higher on average and gave them an advantage over applicants with A-level Calculated Grades, which had been adjusted (*G,* 13th Aug). University admission processes were also becoming embroiled in confusion, and although universities had, “reassured ministers that they will ‘soften’ the grades they normally require” (*T*, 13th August), by the next day universities were accused of being inflexible (*G*, 14th Aug).

[u] https://ffteducationdatalab.org.uk/2020/06/gcse-results-2020-a-look-at-the-grades-proposed-by-schools/

For peer review only - http://bmjopen.bmj.com/site/about/guidelines.xhtml

It soon became apparent that schools in the private, fee-paying sector, had probably benefited from the algorithm, primarily because statistical predictions were less accurate for the small class sizes more likely to be found in private schools, and in those cases the teacher-estimated CAGs had been allowed to stand unadjusted. Although the Prime Minister Boris Johnson defended the system saying, “Let’s be in no doubt about it: the exam results that we’ve got today are robust, they’re good, they’re dependable for employers” (*G*, 14th August), many backbench MPs were in revolt, having been deluged with complaints from constituents (*T*, 14th August). The next day Gavin Williamson said, “No U-turn. No change” (*T*, 15th August), and although he did agree to waive fees for appeals against Calculated Grades, he insisted that the grades themselves would not change in order to avoid the grade inflation that had occurred in Scotland (*T*, 15th August).

Meanwhile the *Ofqual* algorithm, published in a document over 300 pages in length, was being dissected carefully, and when one headteacher anonymously shared their school’s results, the problems became particularly apparent[v]. In the previous three years at their school, 12.5% of pupils had achieved A* and none had got a U; however the algorithm meant only 3.7% of students (equivalent to just one student) received an A* - much below the historic 12.5%. The algorithm also resulted in one student being awarded a U, despite no students at that school having received a U previously. The weekend newspapers attacked the government, “which deserved a U grade for this debacle” (Sunday Times (ST), 16th August). GCSE results, due on Aug 20th, were also on the horizon, with similar problems predicted (*T*, 15th Aug, p.14; *Observer (O)*, 16th August). Students in England demonstrated outside the Department for Education in London. *Ofqual* also announced guidance on the role of mock exams in appeals only to withdraw it a few hours later (*T*, 17th August).

On August 18th the government scrapped *Ofqual’s* algorithm and reverted to unadjusted teacher-estimated CAGs (*G*, 18th August). *The Times*, notionally a supporter of the Conservative Party, simply called its main editorial, “Another Fine Mess” (*T*, 18th August). The chairman and chief executive of *Ofqual* were criticised for having little experience of education (*T*, 19th August), and the Chief Executive eventually resigned on August 25th.

Several other problems now emerged, not the least being that universities would not know the (now unadjusted CAG) grades for several days. Once universities did receive these grades, they found that they did not have enough places to honour all the offers they had made students months earlier. This was because universities typically make more offers than they have places, knowing that a significant number of students will not meet those offers when they achieve lower exam grades than the Predicted Grades estimated by their teachers and submitted to UCAS when they apply to university. But now with the teacher-estimated CAGs replacing exam grades, and the resulting increase in the percentage of A and A* grades from 28% to 38%, many more students than expected did in fact meet their university offers (*T*, 18th August). This caused problems for some students whose Calculated Grades (the grades adjusted by the algorithm) had been too low for their first choice university and so had accepted offers from their second choice, but now they found their unadjusted teacher-estimated CAGs enabled them to meet their first choice offer they inevitably wanted to go there. Other students seemed to have been left in limbo, needing to delay entry until the next year, with a potential knock-on effect for students taking A-levels in 2021 (*T*, 19th August). Some groups of A-level students had clearly fallen through the net and were in limbo, such as who were home-schooled or private A-level students, having no teachers to estimate their grades (*G*, 21st August).

---

[v] Hern,Alex. (2020) “Do the maths: analysis shows why England’s grading system is both imprecise and unfair”, *Guardian*, 15th August, 2020, p.13; the analyses are based on Thomson, Dave, “A-Level results 2020: How have grades been calculated?”, 13th August 2020, https://ffteducationdatalab.org.uk/2020/08/a-level-results-2020-how-have-grades-been-calculated/.

For peer review only - http://bmjopen.bmj.com/site/about/guidelines.xhtml

Regarding medical schools specifically, *The Guardian* reported that the Norwich Medical School at the University of East Anglia had 185 places and a possible overshoot of 50, a 27% increase, emphasising that with medical school numbers more strictly limited than other university places and costing £50,000 a year this would have clear financial implications (*G*, 19th August). A news story based in part on the current research as published in pre-print on *medRxiv,* suggested that more medical students may be liable to drop out, a co-chairman of the Medical Schools Council suggesting that "we are going to have, on average, students with lower grades than in previous years" (*DT*, 20th August). A similar concern was also raised by headteachers in relation to GCSE grades, which it had now been announced would also be based on unadjusted teacher estimates, with fears that students, "could end up on unsuitable courses post-16 which could set them up for failure" (*G*, 21st August).

The cap on university student numbers was released on 17th August, the medical school cap following on 20th August, resulting in a bulge of new undergraduates. There was also a bulge in admissions to sixth form colleges as a result of unadjusted teacher-estimated GCSE grades being higher than the expected exam grades (*G*, 21st August). There are financial implications for educational institutions but until UCAS releases its entry statistics for October 2020, the size of the impacts will be unclear.

The controversies rumbled on into September 2020 as the House of Commons Select Committee on Education heard evidence from *Ofqual* and other bodies. *Ofqual* put out a lengthy statement on 2nd September in evidence to the Committee[w]. It set out the history according to the regulator, stating that in March its advice to the Secretary of State had been that, "the best option in terms of valid qualifications would be to hold exams in a socially distanced manner"; however, "The decision to use a system of statistical standardised teacher assessments was taken by the Secretary of State and issued as a direction to *Ofqual*". In reviewing the failure of the system, the conclusion was reached that, "a 'better' algorithm would not have made the outcomes significantly more acceptable. The inherent limitations of the data and the nature of the process were what made it unacceptable…. *To try to deliver comparable qualification results in the absence of students having taken any assessments (examinations) proved to be an impossible task*" (our emphasis). Cambridge Assessment's submission to Select Committee provides a detailed timeline of collaborative efforts to inform decision making.[x] With the model running and results being calculated, from late July through August, Cambridge Assessment worked with Ofqual and DfE to understand possible unfairness in the outcomes, and to put in place adequate remedy. No doubt the post-mortem will continue for a long while.

In autumn 2020, the needs of the next year began to be considered. One group, including several university Vice-Chancellors, argued that A-levels should be cancelled once more and replaced by teacher-estimated grades (*T*, 2nd October). Others argued more radically that teacher-estimated grades should permanently replace A-level examinations. On 12th October 2020 the Secretary of State announced that A-level exams would go ahead in England in summer 2021 with some minor changes, including being three weeks later than usual, and with results announced a few days later than typical[y]. Contingency measures would be in place for possible disruption, but were yet to be described, although a leaked newspaper report suggested that they might include more formal mock exams as a back-up when following earlier leaked reports (*G*, 10th October). Perhaps the most

[w] https://www.gov.uk/government/news/written-statement-from-chair-of-ofqual-to-the-education-select-committee, "Written statement from Chair of Ofqual to the Education Select Committee", 2nd Sept 2020.
[x] https://committees.parliament.uk/writtenevidence/11358/default/, "Written evidence submitted by Cambridge Assessment", 2nd Sept 2020.
[y] https://www.gov.uk/government/news/students-to-be-given-more-time-to-prepare-for-2021-exams, "Students to be given more time to prepare for 2021 exams", 12th October 2020.

For peer review only - http://bmjopen.bmj.com/site/about/guidelines.xhtml

interesting comment by Williamson was that, “Exams are the fairest way of judging a student’s performance …”, with its tacit acceptance that teacher-estimated grades perhaps actually were not fair to many students.

To date other UK countries have taken different approaches. On 7th October the Scottish Government announced the cancellation of National 5 exams in 2021 although Higher and Advanced Higher examinations would be taken (BBC, 7th October). A month later on 10th November the Welsh Government announced that GCSE and A-level examinations in Wales would be cancelled, with grades being based on classroom assessments instead (BBC, 10th November). On the same day Education Minister Peter Weir announced that GCSE and A-levels examinations would be taken in Northern Ireland (BBC, 10th November). In November 2020 the Secretary of State announced his intention to consider post-qualification university admissions (http://researchbriefings.files.parliament.uk/documents/CBP-8538/CBP-8538.pdf).

This postscript has been relatively brief, given the complexity of the events, but may help those new to the issues to navigate through the major changes that occurred. In research terms, in medical education, higher education more generally, and in secondary education, there seems little doubt that researchers will be following in detail the outcomes for the cohorts affected by the dramatic changes which resulted in a giant, unplanned, experiment, where notional grades awarded were probably different in many cases to what students would have been awarded in normal circumstances.

We began our main paper by quoting a letter to a newspaper at the beginning of lockdown by a student taking GCSEs, and so it is probably appropriate to finish with a letter to a different newspaper by an A-level student, written at the height of the A-levels crisis, which raises many subtle, important and mostly neglected questions, one which researchers will need to grapple with:

> “Ofqual’s grading system appears to be lacking in advocates. Blinded by rhetoric about what protesters call a ‘classist’ algorithm, key facts have been overlooked. It is very clear that teachers are shockingly bad at predicting grades; using teacher predictions there will be a 12% inflation in higher grades compared with last year. While some centres predicted accurately, some centres predicted only the highest grades for their students. This U-turn from the government entails a huge injustice for the pupils who had fair and accurate predictions, as well as for those taking exams next year. In the zero-sum game of university applications, the results of these pupils make them appear weaker than they are. Irresponsible teachers who over-predicted their pupils’ results ought to be ashamed that they too have thereby ‘dashed the dreams’ of many young people across the country. That it is less obvious does not make it any less true.” (Letter to *The Times*, 19th August 2020, by Seb Bird, A-level student, Bristol).

Acknowledgements

We are grateful to Paul Garrud, Gill Wyness, Paul Newton, Colin Melville and Christian Woodward for their comments on earlier versions of this manuscript, and to Jon Dowell, Peter Tang, Rachel Greatrix and other members of the UKMED Research Group and Advisory Board for their assistance in fast-tracking the paper, and for their comments on it. We also thank Tim Gill for providing us with an unpublished manuscript.

Contributors

DTS prepared the data extracts, provided details on date sources and variable definitions where required and commented on manuscript drafts. ICM originated the idea for the study, and discussed

For peer review only - http://bmjopen.bmj.com/site/about/guidelines.xhtml

it with other authors throughout the project. ICM wrote the first draft of the manuscript, and KW, DH, PAT, LP, KYFC and DTS have read, reviewed and commented on earlier drafts and contributed ideas, as well as approving the final draft.

Funding

KW is a National Institute for Health Research (NIHR) Career Development Fellow (NIHR CDF-2017-10-008) and is principal investigator for the UKMACS and UKMEDP089 projects supported by the NIHR funding.

DH is funded by NIHR grant CDF-2017-10-008 to KW.

PAT's research time is supported by an NIHR Career Development Fellowship (CDF 2015-08-11), and PAT is also principal investigator for the UKMEDP051 project.

LWP is partly supported by NIHR grant CDF 2015-08-11 to PAT, and a portion of his research time is funded by the UCAT board.

ICM, KYFC and DTS have received no specific funding for this project.

Disclaimers

KW and DH state that this publication presents independent research funded by the National Institute for Health Research (NIHR). The views expressed are those of the authors and not necessarily those of the NHS, the NIHR or the Department of Health and Social Care.

PAT states this research is supported by an NIHR Career Development Fellowship (CDF 2015-08-11). This paper presents independent research partly funded by the National Institute for Health Research. The views expressed are those of the authors and not necessarily those of the NHS, the NIHR or the Department of Health and Social Care.

KYFC is employed as the Head of Marking and Results at Cambridge Assessment English. The views expressed are those of the authors and do not represent the views of Cambridge Assessment.

DTS is employed by the GMC as a data analyst working on the UKMED project. The views expressed here are his views and not the views of the GMC.

Data sources. UK Medical Education Database (UKMED) UKMEDP051 data extract generated on 13th May 2019. UKMEDP089 extract generated 21st January 2020. UKMEDP112 project, using UKMEDP051 and UKMEDP089 data, approved for publication on 29th May 2020. We are grateful to UKMED for the use of these data. However, UKMED bears no responsibility for their analysis or interpretation.

UKMEDP051 data includes information derived from that collected by the Higher Education Statistics Agency Limited (HESA) and provided to the GMC (HESA Data). Source: HESA Student Record 2002/2003 to 2014/2015. Copyright Higher Education Statistics Agency Limited. The Higher Education Statistics Agency Limited makes no warranty as to the accuracy of the HESA Data, cannot accept responsibility for any inferences or conclusions derived by third parties from data or other information supplied by it.

UKMEDP051 and UKMEDP089 include Universities and Colleges Admissions Service (UCAS) data provided to the GMC (UCAS data). Source: UCAS (application cycles 2007 to 2018). Copyright Universities and Colleges Admissions Service (UCAS). UCAS makes no warranty as to the accuracy of the UCAS Data and cannot accept responsibility for any inferences or conclusions derived by third parties from data or other information supplied by it.

For peer review only - http://bmjopen.bmj.com/site/about/guidelines.xhtml
For peer review only

All data from HESA are required to be reported using their rounding and suppression criteria (https://www.hesa.ac.uk/about/regulation/data-protection/rounding-and-suppression-anonymise-statistics) and we have applied those criteria to all UKMED-based tables and values reported here.

Competing interests

ICM is a member of the UKMED Research Group and the UKMED Advisory Board, and is also on the UKMACS advisory group.

PAT is a member of the UKMED Research Group. PAT has previously received research funding from the ESRC, the EPSRC, the Department of Health for England, the UCAT Board, and the GMC. In addition, PAT has previously performed consultancy work on behalf of his employing University for the UCAT Board and Work Psychology Group and has received travel and subsistence expenses for attendance at the UCAT Research Group.

KYFC is a member of the UKMED Research Group, and is an employee of Cambridge Assessment - a group of exam boards that owns and administers the BioMedical Admissions Test (BMAT); UK GCSEs and A-levels; and International GCSEs and A-levels.

DTS is a member of the UKMED Research Group and the UKMED Advisory Board and is employed by the GMC as a data analyst working on the UKMED project.

KW, DH and LP declare no competing interests.

Authorship

ICM conceived the idea for the study, conducted the statistical analysis and wrote the first draft of the paper. All authors contributed to subsequent drafts and approved the final version.

Ethical approval

Queen Mary Research Ethics Committee, University of London, agreed on 11 November 2015 that there was no need for ethical review of UK Medical Education Database research studies. https://www.ukmed.ac.uk/documents/UKMED_research_projects_ethics_exemption.pdf

All data from HESA are required to be reported using their rounding and suppression criteria (https://www.hesa.ac.uk/about/regulation/data-protection/rounding-and-suppression-anonymise-statistics) and we have applied those criteria to all UKMED-based tables and values reported here.

Provenance and peer review

Not commissioned; reviewed by the UKMED Research Subgroup and Advisory board, and to submitted to an externally peer reviewed journal. This paper was fast-tracked through the UKMED governance processes as with other COVID-19 related research projects elsewhere.

Patient and public involvement.

No patient involvement.

Data sharing statement

Researchers wishing to re-analyse the data used for this study can apply for access to the same datasets via UKMED (www.ukmed.ac.uk).

For peer review only - http://bmjopen.bmj.com/site/about/guidelines.xhtml

References

1. Hussein Y: **Cancellation of GCSE is unfair to some students.** *The Guardian* 2020, https://www.theguardian.com/world/2020/mar/29/cancellation-of-gcse-exams-unfair-to-some-students.

2. Thomson D: *Moderating teaching judgments in 2020 [Blog post, 25th March 2020]*. London: FFT Educational Lab: https://ffteducationdatalab.org.uk/2020/03/moderating-teacher-judgments-in-2020/ (accessed 16th April 2020); 2020.

3. Petch JA: *School estimates and examination results compared*. Manchester: Joint Matriculation Board; 1964.

4. McKie A: **Scapped exams may spark UK admissions 'scramble'.** *Times Higher Education* 2020, **26th March 2020:** 9.

5. Dowell J, Cleland J, Fitzpatrick S, McManus IC, Nicholson S, Oppé T *et al.*: **The UK medical education database (UKMED): What is it? Why and how might you use it?** *BMC Medical Education* 2018, **18 (DOI 10.1186/s12909-017-1115-9):** 1-8.

6. Woolf K, Harrison D, McManus IC: **The attitudes, perceptions and experiences of medical school applicants following the closure of schools and cancellation of public examinations due to the COVID-19 pandemic in 2020 .** *medRxiv* 2020, **submitted**.

7. Ofqual: *Summer 2020 grades for GCSE, AS and A level, Extended Project Qualification and Advanced Extension Award in maths: Guidance for teachers, students, parents and carers*. Coventry: Ofqual: Ofqual/20/6607/2 https://assets.publishing.service.gov.uk/government/uploads/system/uploads/attachment_data/file/877842/Summer_2020_grades_for_GCSE_AS_A_level_EPQ_AEA_in_maths_-_guidance_for_teachers_students_parents.pdf (accessed 3rd April 2020); 2020.

8. Cresswell M: *Heaps, prototypes and ethics: The consequence of using judgments of student performance to set examination standards in a time of change*. London: Institute of Education; 2003.

9. Ofqual: *Setting GCSE, AS and A Level Grade Standards in Summer 2014 and 2015*. London: https://www.gov.uk/government/publications/setting-gcse-and-a-level-grade-standards-in-summer-2014-and-2015 [accessed 18th April 2020]; 2020.

10. Ofqual: *Summer 2020 grades for GCSE, AS and A level, Extended Project Qualification and Advanced Extension Award in maths: Information for Heads of Centre, Heads of Department and teachers on the submission of Centre assessment grades*. Coventry: Ofqual: Ofqual/20/6607/1 https://assets.publishing.service.gov.uk/government/uploads/system/uploads/attachment_data/file/877930/Summer_2020_grades_for_GCSE_AS_A_level_EPQ_AEA_in_maths_-_guidance_for_heads_of_centres.pdf (accessed 3rd April 2020); 2020.

11. McManus IC, Powis DA, Wakeford R, Ferguson E, James D, Richards P: **Intellectual aptitude tests and A levels for selecting UK school leaver entrants for medical school.** *Brit Med J* 2005, **331:** 555-559.

12. Downing SM: **Validity: On the meaningful interpretation of assessment data.** *Med Educ* 2003, **37:** 830-837.

For peer review only - http://bmjopen.bmj.com/site/about/guidelines.xhtml

13. American Educational Research Association, American Psychological Association, National Council on Measurement in Education: *Standards for educational and psychological testing*. Washington, DC: American Educational Research Association; 2014.

14. Kane MT: **Validating the interpretations and uses of test scores.** *JEM* 2014, **50:** 1-73.

15. Bekhradnia B, Thompson J: *Who does best at University?* London: Higher Education Funding Council England (http://webarchive.nationalarchives.gov.uk/20081202000732/http://hefce.ac.uk/Learning/whodoes/); 2002.

16. Higher Education Funding Council for England [HEFCE]: *Differencecs in degree outcomes: The effect of subject and student characteristics. Issues Paper 2015/21*. Bristol: HEFCE; https://webarchive.nationalarchives.gov.uk/20180319114800/http://www.hefce.ac.uk/pubs/Year/2015/201521/ 2015.

17. Higher Education Funding Council for England [HEFCE]: *Differencecs in student outcomes: The effect of student characteristics. Data Analysis March 2018/05*. Bristol: HEFCE; 2018.

18. McManus IC, Smithers E, Partridge P, Keeling A, Fleming PR: **A levels and intelligence as predictors of medical careers in UK doctors: 20 year prospective study.** *Brit Med J* 2003, **327:** 139-142.

19. McManus IC, Woolf K, Dacre J, Paice E, Dewberry C: **The academic backbone: Longitudinal continuities in educational achievement from secondary school and medical school to MRCP(UK) and the Specialist Register in UK medical students and doctors.** *BMC Medicine* 2013, **11:242:** doi:10.1186/1741-7015-11-242.

20. Lough C: **GCSEs: Only 39% teachers think 2020 grades fair for all: Plan for teacher-assessed GCSE and A-level grades prompts concerns about potential teacher bias, TES survey of 19,000 finds.** *TES (Times Educational Supplement)* 2020, **13th May 2020:** https://www.tes.com/news/coronavirus-gcses-only-39-teachers-think-2020-grades-fair-all.

21. Lilford R: **Policy makers should use evidence, but what should they do in an evidence vacuum>.** *ARC West Midlands News Blog [NIHR Applied Research Collaboration, West Midlands]* 2020, **2:** 1-2 (https://arcwm.files.wordpress.com/2020/04/arc-wm-newsblog-2020-04-24.pdf).

22. Hubbles S, Bolton P: *The review of university admissions [Briefing Paper Number 8538, 10 April 2019]*. London: House of Commoons [https://researchbriefings.files.parliament.uk/documents/CBP-8538/CBP-8538.pdf]; 2019.

23. Snell M, Thorpe A, Hoskins S, Chevalier A: **Teachers' Perceptions and A-Level Performance: Is There Any Evidence of Systematic Bias?** *ORE* 2008, **34:** 403-423.

24. Everett N, Papageorgiou J: *Investigating the Accuracy of Predicted A Level Grades as part of 2009 UCAS Admission Process*. London: Department for Business, Innovation and Skills; 2011.

25. Wyness G: *Predicted grades: Accuracy and impact. A report of University and College Union*. London: University and College Union (https://www.ucu.org.uk/media/8409/Predicted-grades-accuracy-and-impact-Dec-16/pdf/Predicted_grades_report_Dec2016.pdf); 2016.

For peer review only

For peer review only - http://bmjopen.bmj.com/site/about/guidelines.xhtml

26. UCAS: *End of cycle report 2017: Qualifications and competition*. Cheltenham: UCAS [https://www.ucas.com/data-and-analysis/ucas-undergraduate-releases/ucas-undergraduate-analysis-reports/2017-end-cycle-report]; 2017.

27. UCAS: *Factors associated with predicted and achieved A level attainment, August 2016*. Cheltenham: UCAS: https://www.ucas.com/file/71796/download?token=D4uuSzur; 2016.

28. Gill T: **Methods used by teachers to predict final Alevel grades for their students.** *Research Matters (UCLES)* 2019, 33-42.

29. Walland E, Darlington E: *Insights on trends in AS Levels, the EPQ and Core Maths: summary report*. Cambridge: 35859 /id}. Cambridge Assessment: https://www.cambridgeassessment.org.uk/.../527125-insights-on-trends-in-as- levels-the-epq-and-core-maths-summary-report.pdf; 2019.

30. Gill T, Benton T: *The accuracy of forecast grades for OCR Alevels in June 2014: Statistics Report Series No 90*. Cambridge: Cambridge Assessment [https://www.cambridgeassessment.org.uk/Images/241261-the-accuracy-of-forecast-grades-for-ocr-a-levels-in-june-2014.pdf]; 2015.

31. Gill T, Rushton N: *The accuracy of forecast grades for OCR Alevels: Statistics Report Series No 26*. Cambridge: Cambridge Assessment [https://www.cambridgeassessment.org.uk/our-research/all-published-resources/statistical-reports/150215-the-accuracy-of-forecast%20-grades-for-ocr-a-levels-in-june-2012.pdf/]; 2011.

32. Gill T, Chang Y: *The accuracy of forecast grades for OCR A levels in June 2012*. Cambridge: Cambridge Assessment Statistics Report Series No.64; 2013.

33. Petch JA: *Fifty years of examining: The Joint Matriculation Board, 1903-1953*. 1953.

34. Murphy RJL: **Teachers' assessments and GCE results compared.** *Educational Research* 1979, **22:** 54-59.

35. Gill T, Chang Y: *The accuracy of forecast grades for OCR Alevels in June 2013: Statistics Report Series No 89*. Cambridge: Cambridge Assessment [https://www.cambridgeassessment.org.uk/Images/241260-the-accuracy-of-forecast-grades-for-ocr-gcses-in-june-2013.pdf]; 2015.

36. Gill T, Benton T: *The accuracy of forecast grades for OCR GCSEs in June 2014: Statistics Report Series No 91*. Cambridge: Cambridge Assessment [https://www.cambridgeassessment.org.uk/Images/241265-the-accuracy-of-forecast-grades-for-ocr-gcses-in-june-2014.pdf]; 2015.

37. Südkamp A, Kaiser J, Möller J: **Accuracy of Teachers' Judgments of Students' Academic Achievement: A Meta-Analysis.** *Journal of Educational Research* 2012, **104:** 743-762.

38. Lumb AB, Vail A: **Applicants to medical school: the value of predicted school leaving grades.** *Med Educ* 1997, **31:** 307-311.

39. McManus IC, Richards P, Winder BC, Sproston KA, Styles V: **Medical school applicants from ethnic minorities: identifying if and when they are disadvantaged.** *Brit Med J* 1995, **310:** 496-500.

For peer review only - http://bmjopen.bmj.com/site/about/guidelines.xhtml

40. Richardson PH, Winder B, Briggs K, Tydeman C: **Grade predictions for school-leaving examinations: do they predict anything?** *Med Educ* 1998, **32:** 294-297.

41. Wilmut J, Wood R, Murphy R: *A review of research into the reliability of examinations: A discussion paper prepared for the School Curriculum and Assessment Authority*. Nottingham: School of Education [available at www.gov.uk/systems/uploads]; 1996.

42. Bramley T, Dhawan V: *Estimates of Reliability of Qualifications*. Cambridge: Cambridge Assessment:[https://assets.publishing.service.gov.uk/government/uploads/system/uploads/attachment_data/file/578868/2011-03-16-estimates-of-reliability-of-qualifications.pdf]; 2010.

43. Wheadon C, Stockford I: *Classification accuracy and consistency in GCSE and A level examinations offered by rthe Assessment and Qualifications Alliance (AQA) November 2008 to June 2009*. Office of Qualifications and Examinations Regulation: Coventry (http://www.ofqual.gov.uk/files/reliability/11-03-16-AQA-Classification-Accuracy-and-Consistency-in-GCSE-and-A-levels.pdf); 2011.

44. Curtis S, Smith D: **A comparison of undergraduate outcomes for students from gateway courses and standard entry medicine courses.** *BMC Medical Education* 2020, **20:** https://doi.org/10.1186/s12909-019-1918-y.

45. Meng X-L, Rosenthal R, Rubin DB: **Comparing correlated correlation coefficients.** *Psychol Bull* 1992, **111:** 172-175.

46. McManus IC, Dewberry C, Nicholson S, Dowell JS, Woolf K, Potts HWW: **Construct-level predictive validity of educational attainment and intellectual aptitude tests in medical student selection: Meta-regression of six UK longitudinal studies.** *BMC Medicine* 2013, **11:243:** doi:10.1186/1741-7015-11-243.

47. McManus IC, Dewberry C, Nicholson S, Dowell J: **The UKCAT-12 study: Educational attainment, aptitude test performance, demographic and socio-economic contextual factors as predictors of first year outcome in a collaborative study of twelve UK medical schools.** *BMC Medicine* 2013, **11 :244:** doi:10.1186/1741-7015-11-244.

48. Burgess S, Sievertsen HH: *Schools, skills, and learning: The impact of COVID-19 on education*. https://voxeu.org/article/impact-COVID-19-education (1st April 2020; accessed 31st May 2020): 2020.

49. Carlsson M, Dahl GB, Öckert B, oth D: **The effect of schooling on cognitive skills.** *Review of Economics and Statistics* 2015, **97:** 533-547.

50. Lavy V: **Do differncecs in schools' instrution time explain international achievemnt gaps? Evidence from developed and developing countries.** *The Economic Journal* 2015, **125:** 397-424.

51. Rowland CA: **The effect of testing versus restudy on retention: A meta-analytic review of the testing effect.** *Psychol Bull* 2014, **140:** 1432-1463.

52. Mwandigha LM, Tiffin PA, Paton LW, *et al* What is the effect of secondary (high) schooling on subsequent medical school performance? A national, UK-based, cohort study *BMJ Open* 2018;**8:**e020291. doi: 10.1136/bmjopen-2017-020291

For peer review only - http://bmjopen.bmj.com/site/about/guidelines.xhtml

53. Russell Group, GuildHE, Million Plus, University Alliance: *Joint statement on university admissions*. London: https://russellgroup.ac.uk/news/joint-statement-on-university-admissions/ (accessed 23rd April 2020); 2020.

For peer review only

For peer review only - http://bmjopen.bmj.com/site/about/guidelines.xhtml

Figure captions

Figure 1: Predicted vs attained A-level grades for individual subjects in applicants to UK medical schools. Accurate predictions are in bold; yellow – over-estimates by 1 grade; orange – over-estimates by 2+ grades; green – under-estimates by 1 grade; blue – under-estimates by 2+ grades. a) Counts; b) attained grades as percentages within predicted grades.

Figure 2: Over-estimates, under-estimate and accurate predicted grades in various studies. Black font: predicted grades; red font: forecasted grades; yellow background: pre-2000; blue background: pre-2010; bold, underlined: averaged results post-2000.

Figure 3: Correlation matrix of selection measures, undergraduate outcome measures, and postgraduate outcome measures (separated by grey lines for clarity). Cells indicate Pearson correlation and N.

Figure 4: Mean Educational Performance Measure (EPM) deciles (revDeciles; 95% CI) in relation to actual A-level grades (green) and predicted A-level grades (red)

Figure 5: Predicted decile outcomes if selection were on Predicted A-level grades (blue) rather than actual A-level grades (orange).

For peer review only

a) Counts of number of cases

		Attained Alevel grades						
		E	D	C	B	A	A*	*Total*
Predicted Alevel grades (points)	E (2 pts)	**200**	35	10	5	0	0	*255 (0%)*
	D (4 pts)	235	**610**	155	35	10	0	*1045 (0%)*
	C (6 pts)	635	1220	**2110**	505	95	5	*4570 (2%)*
	B (8 pts)	635	2095	4755	**7355**	1695	175	*16715 (7%)*
	A (10 pts)	430	1925	8785	35640	**61950**	12655	*121390 (51%)*
	A* (12 pts)	50	135	635	6025	42815	**43395**	*93060 (39%)*
	Total	*2185*	*6020*	*16450*	*49570*	*106570*	*56235*	*237030*
		(1%)	*(3%)*	*(7%)*	*(21%)*	*(45%)*	*(24%)*	

b) Percentages within predicted grades

		Attained Alevel grades						
		E	D	C	B	A	A*	*Total*
Predicted Alevel grades (points)	E (2 pts)	**79%**	14%	..	..	..	..	*100%*
	D (4 pts)	23%	**58%**	15%	3%	..	..	*100%*
	C (6 pts)	14%	27%	**46%**	11%	2%	..	*100%*
	B (8 pts)	4%	13%	28%	**44%**	10%	1%	*100%*
	A (10 pts)	0%	2%	7%	29%	**51%**	10%	*100%*
	A* (12 pts)	0%	0%	1%	7%	46%	**47%**	*100%*
	Total	*1%*	*3%*	*7%*	*21%*	*45%*	*24%*	*100%*

Figure 1: Predicted vs attained A-level grades for individual subjects in applicants to UK medical schools. Accurate predictions are in bold; yellow – over-estimates by 1 grade; orange – over-estimates by 2+ grades; green – under-estimates by 1 grade; blue – under-estimates by 2+ grades. a) Counts; b) attained grades as percentages within predicted grades.

108x60mm (300 x 300 DPI)

Study	Context	Year	A-level range	Over-estimated "Pessimistic"	Accurate	Under-estimated "Optimistic"
**University applicants overall: A-levels, etc**						
Everett & Papageorgiou (2011) [24]	Predicted Grades	2009	A-E	7%	52%	42%
UCAS (27]	Predicted Grades	2012	A*-E	12%	20%	68%
Wyness (2016) [25]	Predicted Grades	2013-15	A*-E	9%	16%	75%
UCAS (27]	Predicted Grades	2016	A*-E	9%	16%	74%
UCAS (27]	Predicted Grades	2017	A*-E	10%	16%	73%
Petch (1953) [32]	Forecasted Grades	1940	School Cert Pass/Fail	2%	89%	9%
Petch (1964) [4]	Non-official forecasted Grades	1963	A+B/C+D/E/O/F	18%	43%	39%
Murphy (1979) [33]	Non-official forecasted Grades	1977	A-E	29%	27%	44%
Gill & Rushton (2011) [30]	Forecasted Grades	2009	A-E	12%	55%	33%
Gill & Chang (2013) [31]	Forecasted Grades	2012	A*-E	13%	48%	39%
Gill & Benton (2015) [29]	Forecasted Grades	2014	A*-E	14%	43%	43%
Gill (2019) [28]	Non-official forecasted Grades	2018	A*-E	20%	45%	35%
	**Mean Predicted Grades**	Pre-2010	A-E	7%	52%	42%
	**Mean Forecasted Grades**	Pre-2010	A-E	20%	42%	39%
	**Mean Predicted Grades**	Post-2010	A*-E	**19%**	**17%**	**73%**
	**Mean Forecasted Grades**	Post-2010	A*-E	**15%**	**46%**	**39%**
**Medical school applicants and students: A-levels and other qualifications**						
Students: Lumb & Vail [37]	Predicted Grades	1995	A-E	7%	52%	41%
Applicants: A-levels (this study)	Predicted Grades	2010-18	A*-E	7%	49%	45%
Applicants: EPQ (this study)	Predicted Grades	2010-18	A*-E	14%	52%	34%
Applicants: SQA Adv Highers (this study)	Predicted Grades	2010-18	A*-E	3%	60%	38%
	**Mean Predicted Grades (Medics)**	2010-18	A*-E	**8%**	**53%**	**39%**
**GCSE grades: All candidates**						
Gill & Chang (2015) [35]	Forecasted GCSE Grades	2013	A*-G,U	12%	47%	41%
Gill & Benton (2015) [34]	Forecasted GCSE Grades	2014	A*-G,U	14%	44%	42%
	**Mean Forecasted GCSE Grades**			**13%**	**45%**	**42%**

Figure 2: Over-estimates, under-estimate and accurate predicted grades in various studies. Black font: predicted grades; red font: forecasted grades; yellow background: pre-2000; blue background: pre-2010; bold, underlined: averaged results post-2000.

108x60mm (300 x 300 DPI)

			**Selection measures applicants**				**Undergraduate outcome measures**			**Postgraduate outcome measures**	
		GCSE grades	Predicted Alevels	Alevel grades	UKCAT	BMAT	EPM	SJT	PSA	MRCP(UK) Part 1	MRCS Part A
	GCSE grades	**1**	**0.452**	**0.421**	**0.265**	**0.223**	**0.180**	**0.190**	**0.201**	**0.212**	**0.173**
			22150	22150	22145	4935	12230	12185	12265	890	430
	Predicted A-level grades	**0.452**	**1**	**0.585**	**0.272**	**0.326**	**0.198**	**0.160**	**0.226**	**0.283**	**0.181**
**Selection measures**		22150		22955	22520	5225	12560	12515	12600	910	440
**in all applicants**	Attained A-level grades	**0.421**	**0.585**	**1**	**0.326**	**0.416**	**0.297**	**0.195**	**0.306**	**0.421**	**0.358**
		22150	22955		22520	5225	12560	12515	12600	910	440
	UKCAT total	**0.265**	**0.272**	**0.326**	**1**	**0.483**	**0.115**	**0.243**	**0.238**	**0.200**	**0.181**
		22145	22520	22520		5080	12385	12340	12420	900	435
	BMAT sections 1 and 2	**0.223**	**0.326**	**0.416**	**0.483**	**1**	**0.089**	**0.239**	**0.321**	**0.378**	**0.319**
		4935	5225	5225	5080		4850	4840	4875	450	240
	UKFPO EPM decile	**0.180**	**0.198**	**0.297**	**0.115**	**0.089**	**1**	**0.319**	**0.470**	**0.509**	**0.535**
**Undergraduate**		12230	12560	12560	12385	4850		12515	12505	905	440
**outcome measures**	UKFPO SJT score	**0.190**	**0.160**	**0.195**	**0.243**	**0.239**	**0.319**	**1**	**0.346**	**0.351**	**0.274**
		12185	12515	12515	12340	4840	12515		12475	905	435
	PSA score	**0.201**	**0.226**	**0.306**	**0.238**	**0.321**	**0.470**	**0.346**	**1**	**0.500**	**0.483**
		12265	12600	12600	12420	4875	12505	12475		910	440
	MRCP(UK) Part 1	**0.212**	**0.283**	**0.421**	**0.200**	**0.378**	**0.509**	**0.351**	**0.500**	**1**	**...**
**Postgraduate**		890	910	910	900	450	905	905	910		10
**outcome measures**	MRCS Part A	**0.173**	**0.181**	**0.358**	**0.181**	**0.319**	**0.535**	**0.274**	**0.483**	**...**	**1**
		430	440	440	435	240	440	435	440	10	

Figure 3: Correlation matrix of selection measures, undergraduate outcome measures, and postgraduate outcome measures (separated by grey lines for clarity). Cells indicate Pearson correlation and N.

108x60mm (300 x 300 DPI)

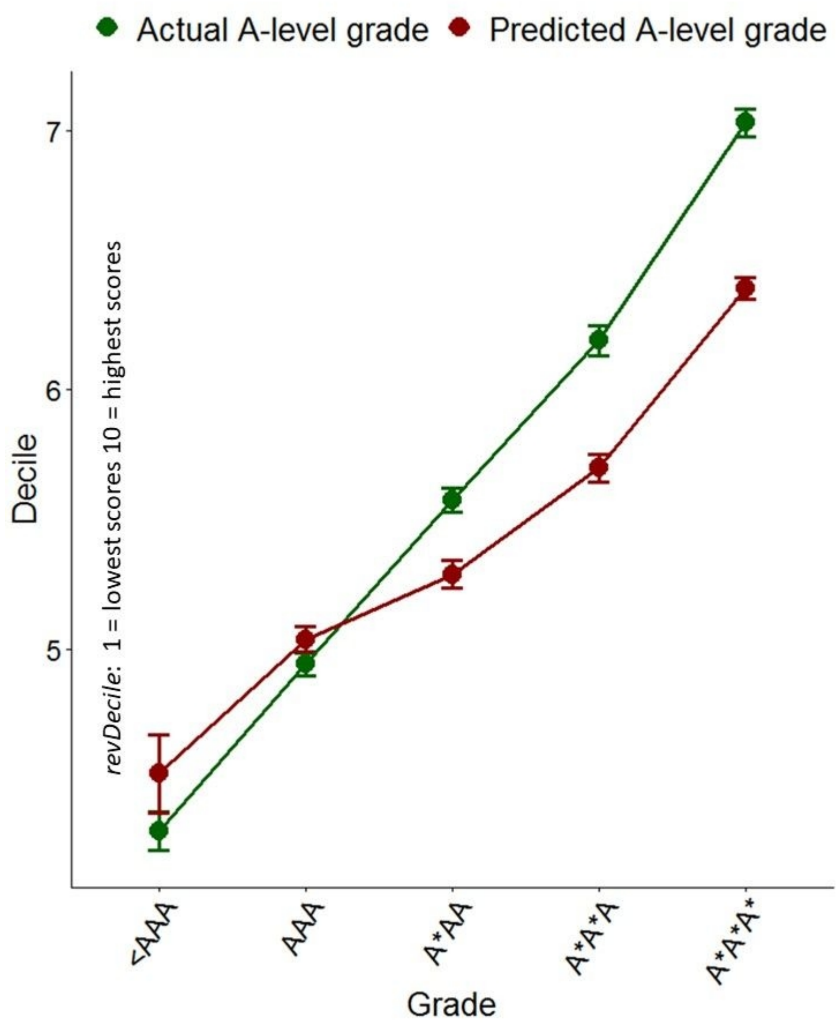

Figure 4: Mean Educational Performance Measure (EPM) deciles (revDeciles; 95% CI) in relation to actual A-level grades (green) and predicted A-level grades (red)

60x88mm (300 x 300 DPI)

		UKFPO		Selection grades:			Absolute	Relative
	Decile	score	*RevDecile*	Attained	Predicted	Odds Ratio	difference	increase
Worst	10	34	1	7.2%	8.1%	1.141	0.9%	13.0%
	9	35	2	9.4%	10.6%	1.135	1.1%	12.0%
	8	36	3	10.1%	11.1%	1.107	1.0%	9.5%
	7	37	4	10.7%	11.2%	1.052	0.5%	4.6%
	6	38	5	10.7%	10.8%	1.003	0.0%	0.3%
	5	39	6	10.6%	10.4%	0.978	-0.2%	-2.0%
	4	40	7	10.7%	10.4%	0.970	-0.3%	-2.7%
	3	41	8	10.3%	9.7%	0.935	-0.6%	-5.8%
	2	42	9	10.2%	9.1%	0.882	-1.1%	-10.7%
Best	1	43	10	10.1%	8.8%	0.853	-1.4%	-13.4%

Figure 5: Predicted decile outcomes if selection were on Predicted A-level grades (blue) rather than actual A-level grades (orange).

108x60mm (300 x 300 DPI)

Calculated grades, predicted grades, forecasted grades and actual A-level grades: Reliability, correlations and predictive validity in medical school applicants, and undergraduates

Supplementary information

- Supplementary literature review
- Supplementary Methods
- Supplementary Results
- *Appendix*: Are independent (private sector) schools more accurate in their A-level predictions?

For peer review only

Supplementary literature review

Overview of literature on predicted, forecasted and attained A-level grades. The majority of studies reported here are also discussed in the main paper, in much more abbreviated form, but here are described more discursively.

University applications in general

Petch in 1964 [1] did what Wilmut has described as "one of the earliest and most celebrated studies of teacher estimates of examination result" [2] (p.60), describing how Petch found, "grade agreement in about 43% of cases, but the examination grade was higher than the teacher estimate in 18% of cases, but lower in 39% of cases, sometimes heavily so".

Two other early studies were by Murphy in the first of which in 1979 he compared actual and predicted grades both for A-levels and, unusually, for O-levels (the predecessor of GCSEs) [3], including two-way tables of predicted vs actual grades. Of 291 results the predicted grades were accurate in 27% of cases, over-predictions in 44% and under-prediction in 29% of cases. Teachers were also asked to provide a rank order of students, and overall these correlated 0.6 with rank order in the examination, although individual teachers showed a range of correlations from just less than zero through to more than 0.9. Murphy's 1981 study drew on application forms submitted to UCCA (now UCAS) by 15,109 candidates, of which "a large number included teachers' pre-examination estimates of A-level grades" (with predicted grades being A, A/B, B, B/C, C etc). Results were broken down by exam board and also by subject. The overall correlation of predicted and actual grades was 0.66, with Physics, Chemistry and French showing the highest correlations. The study also looked at A-level – O-level correlations [4]. Although described as predicted grades, these data are actually best described as being *forecasted grades*.

More recent studies have mostly been concerned with the relationship of attained A-level grades and the predicted A-level grades entered on UCAS application forms by teachers. UCAS changed the way it collected such data in 2009, so that for UK-domiciled applicants subject-level predicted grades

For peer review only - http://bmjopen.bmj.com/site/about/guidelines.xhtml

were available, rather than as earlier when predicted grades were only available as total point scores [5]. For various reasons, not all A-levels have predicted grades. Most analyses are for candidates across all ability levels. Note that A* grades were only introduced in 2010.

In a study of 2009 applicants [5], overall *accuracy* at the subject level for A-levels for 219,744 A-levels was 52%, with predicted and attained grade being the same. In 42% of cases predicted grades were over-estimates, and in only 7% were they under-estimates. A grades tended to be predicted more accurately but that in part reflects that A grades cannot be under-predicted (or E grades over-predicted).

Female candidates showed a slight tendency for grades to be more accurately predicted (52.3% vs 51.1% in males). Socio-economic group showed strong relationships to accuracy, with 58% accurate predictions in the Higher Managerial group and 43% in the Routine group, but that in part reflects different actual A-level achievement (58% of Managerial candidates receiving an A grade compared to 33% of Routine candidates). The Higher Managerial group had the greatest over-prediction and the Routine group the highest under-prediction. Considering ethnicity, 53% of White applicants had accurate predictions compared with 47% of Asian ethnicity, and 39% of those of Black ethnicity. Centre (school) was related to accuracy, with 64% accuracy in Independent schools, 47% in state schools, and 40% in those in Further or Higher education. The authors note that multivariate analyses are probably needed to tease apart the relationships between the various correlates of accuracy. Other analyses looked at disability, region, and nation within the UK. Number of choices also related to accuracy, applicants making four choices being more accurate than those making five choices, but it was suggested that was because of the majority of the former being higher attainers applying to Medicine, Dentistry or Veterinary Medicine. The paper concluded that it is difficult to separate out the various factors involved in accuracy, not least because of the ceiling and floor effects for high and low attainers [5].

Wyness [6] analysed aggregated data provided by UCAS for the applicants from 2013-15, and hence A* grades were included in the analysis. Overall only 16.1% of grades were accurately predicted, a much lower figure than the earlier study using 2009 data [5], perhaps because of the inclusion of the new A* grades. 8.54% of grades were under-predicted, while 75.4% of grades were over-predicted. As with the 2009 data, there was a clear relationship between over-prediction and attained grade, although it is noted that there are strong ceiling effects at work. As with the 2009 study, independent schools provided the most accurate predictions. Applicants from disadvantaged backgrounds showed moderate to severe over-prediction. Asian and Black applicants were also more likely to be severely over-predicted. There were no differences between male and female applicants. The report is particularly interesting as it looks at prediction in high ability students, defined as AAB or above. The difference between the most and least disadvantaged in this group is much smaller, with 44.0% overpredicted in the most disadvantaged and 47.4% in the least disadvantaged. There was some evidence that under-predicted applicants tended to show under-matching (i.e. entering less competitive universities than their actual grades might predict). Further analyse and discussion of these data are provided elsewhere [7,8].

UCAS in 2017 provided some limited data on over-prediction and under-prediction of A-levels since the introduction of A* grades, with data for 2012, 2016 and 2017 [9]. Overall 19.5%, 16.3% and 16.0% of predictions were accurate, with over-prediction in 68.4%, 74.3% and 73.3% of cases, and under-prediction in 11.8%, 9.0% and 10.4% (figures from EoC17_Figure7_9_database.csv[1]). UCAS commented that, the gap between achieved and predicted A-level grades, “continues to widen” (p.23), although a comparison of 2016 and 207 results concluded that there was little effect effect due to the reforms in A-levels that took place in 2017.

[1] https://www.ucas.com/file/140426/download?token=tUxAGXtt

For peer review only - http://bmjopen.bmj.com/site/about/guidelines.xhtml

Not all studies have used the *predicted grades* provided to UCAS for use by universities in selection, which for medical school applicants would have been by mid-October). Until 2015 teachers were also asked, by the end of the following May, just before A-levels were sat, to provide *forecasted grades* to Awarding Organisations, and those grades then contributed in part to decisions on grading. Forecasted grades are clearly of particular interest given proposals for calculated grades to be based on estimates of performance by schools during May. Three analyses are available, for candidates taking A-levels in 2009 [10], 2012 [11] and 2014 [12] which are before and after A* grades were introduced. A primary interest must be the comparison of these forecasted grades with the more usually studied predicted grades, described earlier for 2009 [5] and 2012 [9]. Note that the studies of forecasted grades are only for OCR (Oxford, Cambridge and Royal Society of Arts Examination Board) and hence include all A-level candidates, whereas the studies of predicted grades are for university applicants. Supplementary table 1 compares the two sets of predictions. In 2009 there is little difference between predicted and forecasted grades in accuracy, with a small diminution of over-predictions. The picture three years later, in 2012 after A* grades have been introduced, is rather different. Forecasted grades have an accuracy of 48% compared with only 20% for predicted grades. Taken overall it is difficult to reconcile the two studies which are only three years apart. Based on the 2009 data it would seem that predictions in May are no more accurate than those in October, whereas the 2012 data suggest that May predictions are much more accurate than October predictions. Having said that, even in May 2012, slightly less than a half of forecasted grades are accurate, with the same grade as in October.

It should be noted, as pointed out earlier, that the early studies by Murphy should probably be regarded as being of forecasted and not predicted grades.

Grade point predictions. The analyses described so far have been at the level of A-level subjects. Students mostly take three or sometimes more A-levels, and universities usually look at the three best grades attained. Scoring grades as A*=12, A=10, B=8, C=6, D=4 and E=2 then a candidate passing three A-levels will score between 6 and 36 points for their three best grades[2]. Two studies [10,11] have pointed out the difficulty of using totalled points. As an example, a candidate predicted AAA will be predicted 30 points but may attain grades AAA or grades A*A*D; both are equally accurate in point terms but not in grade terms. Total predicted points are important in that UCAS for a number of years only provided total predicted points for the best three A-levels, without subjects or individual grades being specified[3].

UCAS in 2016 reviewed predicted and actual A-level grade points in applicants from 2010 to 2015 [13] considering the best three grades attained. Achieved grades were one or two grades in total lower for attained than predicted grades. About a half of applicants in 2015 missed predicted total grades by two or more grades (e.g. ABB rather than AAA), a proportion that had increased by a third since 2010. Simple analyses in particular showed that missing predicted grades was associated with having *lower* predicted grades overall (as in the earlier analyses at the subject level). Multivariate analyses i.e. taking other factors into account, found missing predicted grades was associated with having *higher* predicted grades, lower GCSE attainment, taking biology, chemistry and maths, having Asian, Black, Mixed and Other ethnicity, coming from disadvantaged areas, being female, and having '[pre-A-level]unconditional offers'. Of particular interest is the relationship to GCSE grades, which have a strong relationship to A-level attainment [14] which is clearly seen in the UCAS data (see their figures 5 and 6).

---

[2] Some studies, including my own earlier ones, score A*=6, B=5, etc.. Such schemes became less popular with the advent of AS-grades, which were scored as half of an A-level, and hence it made sense to double the points available for a full A-level so that totals remained integer. With the near disappearance now of AS-levels that rationale makes less sense.

[3] Earlier studies, such my 1991 cohort, had to extract predicted grades from UCAS references, and hence they are often embedded in free text, making it difficult to match them up with specific A-level subjects.

For peer review only - http://bmjopen.bmj.com/site/about/guidelines.xhtml

What are predicted grades and how are they made?

UCAS, in its document, "Predicted grades – what you need to know"[4] says that "A predicted grade is the grade of qualification an applicant's school or college believes they're likely to achieve in positive circumstances." Later the document says predicted grades should be, "**in the best interests of applicants** – fulfilment and success at college or university is the end goal ", and "**aspirational but achievable** – stretching predicted grades are motivational for students, unattainable predicted grades are not" (all emphases in original). It also says that grades should be "determined by professional judgement" and be data-driven, including "past Level 2 and Level 3 performance, and/or internal examinations to inform your predictions".

Gill [15] has described the relatively sparse literature on how teachers estimate grades. Gill's own study followed the methodology of Child and Wilson [16] although that study is not in the public domain. Gill sent questionnaires in May to selected OCR exam centres concerning Chemistry, English Literature and Psychology, and as well as estimating grades teachers were also asked to rank within grades, the method currently being adopted by Ofqual for calculated grades[5]. Teachers also indicated the evidence they had used for each decision. The response rate was extremely low (2.8%). About 45% of forecasted grades were accurate (which is similar to the 48% in supplementary table 1). Detailed A-level raw marks were also available and could be correlated with rankings, giving correlations of .87, .76 and .83 for the three subjects. Those correlations are high, and certainly are higher than a meta-analytic estimate of the effect size for teachers predicting academic achievement in pupils of 0.63 (SE=.03), although there was substantial heterogeneity. They are also higher than Murphy's 1979 estimate of 0.66 for the correlation of rankings and exam marks [3]. The most important information said by teachers to be used when predicting grades was performance in mock exams, and observations of quality of work and commitment, with oral presentation also important. Amongst other topics written in, the most important was the opinion of other teachers both in the same subject and other subjects, including the head of department. Other teachers raised concerns about the lack of high stakes for mock exams which meant that students did not treat them seriously. There were also concerns about the loss of AS-levels to help in prediction.

Other examinations. We know of no studies that have looked at accuracy of prediction of Scottish Highers or Advanced Highers, of the EPQ (Extended Project Question) used in England, or of other examinations carried out in the UK.

Applications to medical school

Relatively few studies have looked at predicted grades in medical school applicants, although those studies do show a tendency to ask rather more stretching questions, perhaps because of the different interests of the researchers, and the specificity of the course and its outcomes.

Lumb and Vail pointed out that predicted grades are particularly important in the shortlisting phase of medical student selection [17]. They studied 1661 applications in 1995 to a single medical school who had estimated grades for 5053 A-levels, 52% of predictions being accurate, 41% were over-estimated and 7% under-estimated [17]. The authors presented an ROC curve (but not the area under the curve), and concluded that, "… selectors for medical schools can have some confidence in

[4] https://www.ucas.com/advisers/managing-applications/predicted-grades-what-you-need-know [Accessed 13th April 2020].

[5] One teacher refused to take part because of the difficulty of ranking 260 students sitting one exam. Another teacher commented, "it was easier for smaller centres to make predictions because they know individual students better" (p.42). The paper in fact comments that, "Responses to the questionnaire were more likely to come from smaller centres. … [T]he maximum centre size amongst the sample data was only 40 for Chemistry (compared with 423 amongst all centres), 26 for English Literature (compared with 180) and 32 for psychology (compared with 378)."

For peer review only - http://bmjopen.bmj.com/site/about/guidelines.xhtml

the accuracy of predictions and we should therefore continue to use them … [for] selecting the doctors of the future.” (p.311).

Richardson et al, studied 721 entrants from 1991 to 1994 to a single medical school [18]. Unusually they looked at predictive validity, assessing how well predicted and actual A-level grades related to a composite outcome on the pre-clinical course. Predicted and actual A-level grades showed a minimal correlation (r=0.024), but selection would have imposed range restriction. Pre-clinical exam performance correlated 0.318 (p<.001) with attained A-level grades, but only 0.041 (NS) with predicted A-level grades. This is a rare study in which predictive validity was assessed and it implied that selection should be on actual grades rather than predicted grades, concluding in contra-distinction to Lumb and Vail that, “medical school admissions panels would be well advised to take the predicted grade with a sizeable pinch of salt” (p.296).

A third study, by one of the present team, took a different approach, using path modelling to assess the causal inter-relationships between GCSE grades, predicted A-level grades, receipt of an offer, actual A-level grades, and acceptance at medical school in an original sample size of 6901 applicants to five English medical schools [19]. A-level estimates were predicted by GCSE grades (beta=0.89), with attained A-level grades predicted by both GCSE grades (beta=0.44) and predicted A-level grades (beta=0.74). A substantive question of interest was whether the paths in the model differed between White and non-White candidates, with it being shown that none of the relationships described showed ethnic differences (although non-white candidates were significantly less likely than White candidates to receive an offer based on predicted A-level grades). Although the study reported no follow-up into the medical course, this dataset is analysed further below to assess predictive validity for postgraduate examination performance.

A comment on issues in studying predicted A-level grades.

Although predicted A-level grades have been an integral part of university application and selection in the UK for four decades, obtaining data on them is less than easy. Early studies, including my own, as well as those of other medical researchers, simply resorted to having researchers transcribe grades from paper UCCA and UCAS application forms, although often that was not easy in earlier forms as the predictions were often embedded in the free text of the Referee’s Statement. Until 2009 UCAS only recorded the summed score of the best three A-levels, so that study of specific subjects was not possible. Even now obtaining UCAS data on predicted grades is less than easy, and Boliver in 2013 comments, “It would have been desirable to include predicted A-level grades… . Unfortunately UCAS are unable to provide this information in microdata form because of uncertainty about its validity in the case of applicants whose application is not linked to a school or college … (personal communication from UCAS).” [20]. Similarly Wyness in 2016 in her study of three years of UCAS data comments that, “The data are aggregate (for reasons of privacy)” [6], which means of course that proper analyses at the level of individual participants are not possible. There is an irony here in that of course all universities have access to predicted grades provided by UCAS as a part of the admissions process, but subsequently obtaining those data for research is often very difficult. The data for the present study are the result of an important collaboration between UKMED and UCAS, with UCAS providing detailed information on applicants to UK medical schools for inclusion in the database, which is hosted in a safe haven to ensure strict controls on access; we are very grateful to UCAS for that collaboration without which the present study would not be possible.

Supplementary methods

Data for the present study comes from two separate primary sources:

For peer review only - http://bmjopen.bmj.com/site/about/guidelines.xhtml

"P89". UKMED project UKMEDP089, "The UK Medical Applicant Cohort Study: Applications and Outcomes Study", approved Dec 7th, 2018, with Dr Katherine Woolf as principal investigator, is an ongoing analysis as a part of UKMACS (UK Medical Applicant Cohort Study). Data are primarily concerned with the process of selection. In particular in the upload of 21st Jan 2020[6] there is detailed information from UCAS on all applicants to medical schools from 2007 to 2018, including all attained Key Stage 5 (Level 3) qualifications (e.g. A-levels and SQA) as well as teacher predicted grades for individual Key stage 5 qualifications.

"P51". UKMED project UKMEDP051, "A comparison of the properties of BMAT, GAMSAT and UKCAT", approved Sept 25th, 2017, with Dr Paul Tiffin as principal investigator, is an ongoing analysis of the predictive validity of admissions tests and other selection methods such as A-levels and GCSEs in relation to undergraduate and postgraduate attainment. A major feature of the study is the inclusion of data from UCAS, although in the13th May 2019 data upload, which was used here[7], UCAS predicted grades were only available as a composite, 18-point score, for application years 2010 to 2014. A new upload of the data in late April 2020 will provide more detailed information, but that will require quite extensive coding, etc., making it similar to the qualifications data for applicants in P89. For the present data upload, predicted A-level grades are in the old UCAS format consisting of a single number from 6 to 18 (i.e. 3 Es to 3 A*s using A*=6 coding). Outcome data for the P51 dataset are more extensive, and in particular include data for end of undergraduate training, including the UKFPO EPM measures, the UKFPO SJT as well as PSA (Prescribing Safety Assessment). Some data are available for later postgraduate examinations, but numbers inevitably are small for cohorts entering medical school in 2011 onwards.

A-level grade scoring. In both P89 and P51, A-level grades are expressed numerically on a standard scale of A*=12 points, A=10, B=8, C=6, D=4 and E=2, or have been rescaled to that score.

Rounding and suppression criteria. All data from HESA are required to be reported using their rounding and suppression criteria (https://www.hesa.ac.uk/about/regulation/data-protection/rounding-and-suppression-anonymise-statistics) and although not all data in the current study use HESA measures we have nevertheless applied the HESA criteria to all UKMED-based tables and values reported in this study. It should be noted in particular that the presence of a zero or a zero percentage may not always mean that there are no individuals in a cell of a table. All Ns are rounded to the nearest 5 which should easily flag up that rounding has been applied, all counts ending in 0 or 5. Percentages are only reported when the number of participants is greater than 22.5.

Supplementary Results

Predicted and actual grades for Key Stage 5 qualifications.

Predicted and actual grades for individual A-levels. Supplementary table 2 shows the relationship between predicted and attained A-level grades for 237,030 individual examinations from 2010 to 2018. Supplementary table 2.a shows frequencies in the various combinations, with bold values in grey boxes on the diagonal indicating accurate prediction of grades, green and blue indicating under-prediction by 1 or 2 grades, and orange and red indicating over-prediction by 1 or 2 grades. Overall 48.8% of predicted grades are accurate. Under-prediction occurs by one grade in 35.7% of cases, and by two or more grades in 9.0% of cases. Over-prediction is by one grade for 6.3% of A-levels, and 0.1% by two or more grades. It should be remembered that since the median grade for actual A-

[6] OUTPUT_UCAS_QUALS_DEC_20200121_1.TXT and OUTPUT_UCAS_QUALS_DEC_20200121_2.TXT (both dated 21/1/2020), and OUTPUT_UCAS_QUALS_VER.TXT (dated 5/12/2019).
[7] UKCAT51_APP_ALL_DATA_13052019_FILE1.SAV and UKCAT51_APP_ALL_DATA_13052019_FILE2.SAV (both dated 13/5/2019).

For peer review only - http://bmjopen.bmj.com/site/about/guidelines.xhtml

level grades is A, then over-prediction in such cases can only be by a maximum of one grade, since A* is the highest grade.

Supplementary tables 2.b and 2.c show the data of supplementary table 2.a as percentages. As has been pointed out [5] percentages within predicted grades and percentages within actual grades have different interpretations and uses. Both are presented here, but from the perspective of admissions tutors perhaps the most useful are those in supplementary table 2.b of percentages within predicted grades in relation to actual grades, as they show the likelihood that a predicted grade will actually manifest as particular actual grades. About a half of A* predictions actually gain an A grade, and over a third of predicted A grades result in a grade B or lower.

Allocating points on the basis of A*=12, A=10, B=8, C=6, D=4 and E=2, predicted grades show systematic *bias*, the mean prediction of 10.53 points being systematically higher than the mean actual grade of 9.55 points, the difference of 0.98 points being about half of an A-level grade, and can be seen in the greater numbers in red and orange cells in supplementary table 2.a (over-prediction, 45%) than in the blue and green cells (under-prediction, 6%).

Despite the bias, predicted grades overall show a reasonable *correlation* with actual grades, with a Pearson r_p of 0.624 and a Spearman correlation r_s of 0.581. Both predicted and actual grades are skewed because of censorship, values above A* not being possible. A tetrachoric or polychoric correlation fits an underlying latent normal distribution into account, accepting that row and column totals may not be equally spaced, being ordinary in nature [21]. Using the *polychor()* function in *R* the polychoric correlation, r_t is somewhat higher at 0.716 (SE 0.002), and is probably the best estimate of the true extent of correlation.

Differences between A-level subjects. A-levels in different subjects may show differences in their degrees of bias or correlation. Subjects were divided into 26 broad groups (see supplementary table 3), with the Modern Languages group including 21 languages.

Supplementary table 3 shows the mean predicted points, the mean actual points, actual minus predicted points, and the Pearson correlation of predicted and actual points. Subjects are sorted by the number of examination entries, and values are colour coded on a green-yellow-red scale, green indicating higher predicted and actual grades, a smaller difference between predicted and actual grades (i.e. less bias), and higher correlation of predicted and actual grades.

Considering the four major subjects of chemistry, biology, maths and physics, differences between actual and predicted grades are very similar (-1.15 to -0.98) indicating a bias of about 1 point (i.e. half of a grade) and very similar correlations of 0.600 to 0.635. Amongst other subjects there is inevitably greater variation in those subjects taken less frequently. Of particular interest, given that some medical schools use it for selection, is General Studies, which has the largest difference of predicted and actual grades of -1.96 points, equivalent to a whole grade. The smallest bias is for art and design subjects at -.57 points, perhaps indicating the role of an incourse portfolio in these subjects giving teachers a better sense of how students are performing. Correlations of predicted and actual grades are mostly very similar, although the lower correlations are for general studies, modern languages, geography, history, economics, music and classics, and, as mentioned, for general studies.

Total predicted and actual points, correlations between grades and reliability of measures

Reliability of actual and predicted A-levels. The reliability of total points from the three best actual and predicted A-levels was calculated by randomly sampling a pair of grades from the best three and finding the correlation. Cronbach's alpha for the three totalled grades could then be calculated from the standard formula, Alpha = 3.r/(1+2.r) where r is the mean correlation, and is equivalent to a single randomly sampled correlation between a pair of grades since any pair should give similar results. Analysis was restricted to the 66,006 candidates who had at least three paired predicted and actual grades. For actual grades r=0.615 (SE .003) giving alpha=0.827, while for predicted grades

For peer review only - http://bmjopen.bmj.com/site/about/guidelines.xhtml

r=0.550 (SE = .004) and hence alpha =0.786. Given the standard errors, the correlation between grades is clearly substantially lower for predicted than actual grades, and the same must be true of alpha. Interpreting the difference is not entirely straightforward, since on the one hand more predicted grades are at A*, meaning that there should be fewer non-identical grades, but range restriction might also result in a lower correlation. In terms of mechanism, teachers may collaborate in producing predicted grades [15], and such non-independence would increase correlations and increase alpha. However teachers may also spend less time making judgements than do A-level examiners, and hence there should be lower correlations. On balance it seems that the most likely conclusion is that estimated grades are somewhat less reliable than actual grades, but there is clearly a need for more complex modelling of the reliability of actual and estimated grades.

Predicted and actual grades for Extended Project Qualification (EPQ). The English EPQ has become popular qualification for medical school applicants, being taken by 18616 applicants over the years 2018 to 2018, about 2100 applicants a year (perhaps 10% of all applicants). There is evidence that it has predictive validity for degree outcomes [21]. At present it is not known if it predicts outcomes in application or at medical school. Supplementary table 4 shows the relationship between actual and predicted grades. Grades are over-estimated in 33.7% of cases, under-estimated in 14.0% and accurate in 52.3% of cases, the mean score difference, the bias, being 0.805, which is a little under half a grade. Pearson's correlation is r_p=.459, Spearman's correlation is r_s=.457, whereas the polychoric correlation is somewhat higher at r_t=.569.

Predicted and actual grades for SQA Advanced Highers. SQA Advanced Highers, as with SQA Highers, are scored both as simple literals (A, B, C D) and as a more extended scoring (A1, A2, B3, B4, C5, C6, D7), although predicted grades are only in terms of literals. Supplementary tables 5.a and 5.b show, that A grades are more frequent in predicted than in attained grades. Using literals, 59.8% of predictions are accurate, 37.7% are over-estimated, and 2.6% are under-estimated, and for literal grades the bias was 0.976 points, equivalent to half a grade. Correlations of predicted grades with literal attained grades were r_p=.407 and r_s=.357, whereas with extended grades were r_p=.409 and r_s=.355. Polychoric correlations were r_t=.575 for literal grades and r_t=.587 for extended grades, again showing the similarity across the two grading schemes.

Summary. Taking all the exam types together, A-Levels, EPQ and SQA Advanced Highers, it is generally clear that predicted grades are usually about a half-grade higher than actual grades. Where grades are not accurate there are about four times as many grades over-estimated as under-estimated.

Predictive validity of predicted and attained A-level grades.

A key question throughout discussions of calculated grades is whether grades estimated by teachers are better or worse at predicting outcomes than are actual A-level grades. That question is answered not in terms of how well predicted grades relate to actual A-level grades, but by assessing how well predicted and actual grades predict subsequent outcomes during undergraduate and postgraduate training. It should also be said that it is not entirely self-evident that teachers' grades will be less good, and in the context of GCSEs rather than A-levels, Thomson said, "It is possible, in theory at least, that teacher judgements may be more reliable than exam grades, particularly in those subjects where exam reliability is lower" [22], with "more reliable" being somewhat ambiguous and perhaps also meaning more valid as well as more reliable in the narrow statistical sense. Questions about predictive validity can be answered by the P51 dataset.

Predictive validity in P51. The P51 UKMED data includes only applicants applying for medical schools. Predicted A-level grades were available only for the UCAS application cycles of 2010 to 2014, and consisted of a single score in the range 2 to 18 points, based on the three highest predictions scored as A*=6, A=5, etc.. The modal score for 38964 applicants was 15 (equivalent to AAA; mean=15.88; SD= 1.79; Median = 16; 5th, 25th, 75th and 95th percentiles= 13, 15, 17 and 18). Some older applicants had only pre-A* A-levels, and it was also desirable to restrict the analysis to

For peer review only - http://bmjopen.bmj.com/site/about/guidelines.xhtml

standard applicants in their first year of application, and so only those aged 18 in the UCAS year were included. For multiple reasons not all applicants had both predicted grades and attained A-level grades, and analysis was restricted to the 22954 applicants with both predicted and attained grades. Other selection measures which were included in the analysis are GCSEs (mean grade for best eight grades), as well as U(K)CAT and BMAT scores, which are based on the most recent attempt which in most of the present cases is also the first attempt. For simplicity we used the total of the four sub-scores for U(K)CAT, and for BMAT the total of the Section 1 and 2 scores. No GAMSAT scores were available for this age-group.

Outcome measures are complicated as different application cohorts enter medical school and graduate at different times, and lags within the system mean that not all outcome measures are available. In this UKMED data extract, applicants to UCAS in 2010 entered the medical register from 2015-18, 2011 applicants in 2016-8, 2012 applicants in 2017-18 and 2013 applicants in 2018. Applicants for 2014 would only have qualified in 2019 but the UKMED dataset did not yet include that years, and some earlier entrants would also be expected to qualify after 2018. For simplicity, outcome measures were restricted to the deciles of the UKFPO's Educational Performance Measure (EPM), the raw score of the UKFPO's Situational Judgement Test (SJT), and the score relative to the pass mark of the Prescribing Safety Assessment (PSA), all at first attempt, as these are the main outcomes from undergraduate training. Insufficient numbers of doctors had progressed further in postgraduate training to make analysis meaningful in this data extract.

Supplementary table 6 (presented also in the main paper) summarises the correlation matrix of the various measures. It is important to note that the large differences in Ns are primarily because some measures are present in applicants and used during *selection*, and others are undergraduate outcome measures from medical school, which of necessity are only present in *entrants*, and some are postgraduate outcome measures, only present in *graduates*, not all cohorts yet having reached that stage. The three parts of the correlation matrix are separated to clarify the distinction. Correlations of selection and outcome measures necessarily show range restriction because candidates have been selected on the basis of these measures, and in the case of graduates, selected and self-selected, so that they are less variable than would be the case in an unrestricted population of applicants. The most important question for these data is the extent to which Predicted and Attained A-level grades (shown in pink and green in Supplementary table 6) differ in how much they predict the three outcome measures, which typically are taken five or six years later.

Prediction of Educational Performance Measure (EPM). EPM is probably the most important outcome measure since it integrates educational performance across assessments for all but the final year of the undergraduate course[8]. Note that deciles are confusing, as UKFPO scores them in the reverse of the usual order, the first decile being highest performance and the tenth the lowest. Here for ease of interpretation we reverse the scoring in what we call *revDecile*, so that higher *revDeciles* indicate higher performance. EPM is a summary of outcome across assessments within a medical school, expressed as deciles of achievement within each school. EPM is predicted r=0.297 by attained A-level grades but only r=0.198 by predicted grades. Although in absolute terms those correlations may seem small it must be remembered that they are range restricted, and the construct level predictive validity, taking into account range restriction and measurement error is likely to be much higher [23]. N is large for these correlations and hence the differences are highly significant using Meng and Rosenthal's test for correlated correlations [24], Z = 12.6, with $p<10^{-33}$. Although predicted grades predict less well than attained grades, they may predict differently, and hence contribute something over and above attained grades in predicting outcome? Entering predicted grades after attained grades in a multiple regression shows a highly significant but small additional prediction of predicted grades (beta=.052, compared with beta=.269 for attained grades).

[8] https://foundationprogramme.nhs.uk/wp-content/uploads/sites/2/2019/11/UKFP-2020-EPM-Framework-Final-1.pdf

For peer review only - http://bmjopen.bmj.com/site/about/guidelines.xhtml

Attained grades are therefore substantially better at predicting undergraduate outcome, but predicted grades may have a small amount of variance which is not shared with attained A-levels.

Can other measures replaced attained A-level grades for predicting EPM? In the absence of attained grades, to what extent can other selection measures such as GCSE grades, U(K)CAT and BMAT replace the predictive variance in attained A-level grades? Regressing EPM on just predicted grades gives multiple R = .198, compared with an R of 0.297 when regressed on just actual grades. Adding GCSEs to Predicted grades increases R to .225, while also including U(K)CAT and BMAT increases R to .231, although that is still far short of the .297 from A-levels alone. Interestingly if Actual Grades are now added in to the equation as well, R increases to .308, which is higher than the R for just actual grades. Exploration suggests that the effect is due to the additional effect of GCSEs grades compared with just having attained A-level grades in the model (R=.306; Beta(attained grades)=.268, beta(GCSES)=.077). Overall therefore if only Predicted Grades are available, an improved prediction is obtain by including GCSEs and U(K)CAT/BMAT, although the model still falls short of that of actual A-levels in terms of prediction.

Private and State Sector schooling and EPM. The UKCAT-12 study [25] found that medical students educated in the private sector performed less well at medical school than those educated in the state sector with equivalent A-level grades. It is important to replicate that finding in the present data, and to explore the extent to which there are effects related to predicted as opposed to attained grades. Overall 6149 (26.8%) of students were educated in the private sector, compared with 16805 (73.2%) in the state sector. Supplementary figure 1 plots *revDecile* in relation to attained and predicted grades, separately by private and state education. Visually it is immediately clear that there is an overall main effect of schooling, the lines for private sector schools (pale green and pale red) being below those for state schools. Note that the point for private schools with predicted grades <AAA is missing, as N was very small, because of few private schools predicting grades below AAA. Considering just attained grades, regression showed effects of both A-level grade (b=.299 (SE .008)[9], beta=.301, t=35.24, $p<10^{-100}$) and private schooling (b= -.292 (SE=.053), beta= -.047, t=-5.478, $p=4\times10^{-8}$), but the addition of an interaction was not significant (t=0.746, p=.455) meaning that the slopes in supplementary figure 1.a 1.b are the same. A similar analysis for predicted grades found effects of predicted grade (b=.213 (SE .009), beta=.201, t=22.94, $p<10^{-100}$) and private schooling (b= -.256 (SE .055), beta= -.041, t=-4.679, p=0.000003), but the addition of an interaction was not significant (t=0.680746, p=.455), again meaning that the slopes are similar in the two types of school in supplementary figure 1.b. The standard errors for the effects of private schooling suggest that the difference between the slope is similar for actual and predicted grades.

Supplementary table 6 contains a number of other interesting features.

Other outcome measures in relation to actual and predicted A-levels. There are four other outcome variables, two undergraduate and two postgraduate. For the undergraduate measures, PSA mark (supplementary figure 2) and SJT score (supplementary figure 3), both correlate more strongly with attained A-level grades than predicted A-levels (PSA: Z= 10.31, $p<10^{-23}$; SJT Z= 4.38, p=0.000012). The two postgraduate outcome measures, are based on smaller, but still substantial, numbers of doctors, MRCP(UK) Part 1 being taken by 910 doctors, and MRCS Part A by 440 doctors. Both outcomes have higher correlations with attained A-level grades than predicted grades, MRCP(UK) Part 1 correlating 0.421 with actual A-level grades (supplementary figure 4), and 0.283 with predicted grades (Z= 4.54, p=.000055), and MRCS Part A correlating 0.421 with actual grades (supplementary figure 5) compared with 0.358 with predicted grades (Z= 3.67, p=.000238). The five outcome measures therefore show the same broad pattern of results.

---

[9] Actual and Predicted grades are scored on the basis of A*=12, A=10 etc so are in the range 6 to 36 for three best grades. b=.299 therefore means an increase of 0.3 deciles per step on the A-level grade score, and therefore a full A-level grade (e.g A*AA compared with AAA is 0.6 EPM deciles higher).

For peer review only - http://bmjopen.bmj.com/site/about/guidelines.xhtml

Correlations of outcome measures and the status of the SJT. The five outcome measures correlate well with each other (mean r = .420)[10], as might be expected given the academic backbone [27]. Noteworthy is the relatively low correlation of SJT with EPM (.319) and PSA (.346), compared with the correlation of EPM and PSA (.470). That pattern is repeated when postgraduate exams are included, the four non-SJT assessments showing a higher correlation (mean r=.499) than the correlations of the four non-SJT assessments with SJT (mean r = .322). Overall that suggests that SJT may be measuring a construct that is different in part from the other more academic assessments, and that will need investigating more closely in the future. It is also of interest when considering predicted grades that SJT correlates only slightly better with actual grades than predicted grades (.195 vs .160), compared with the other four outcomes (.297 vs .198; .306 vs .226; .421 vs .283; and .358 vs .181; mean r = .346 vs .222) raising the possibility that predicted grades may include some non-academic variance which then is predictive for SJT. That can be tested by regressing SJT on actual and predicted grades, when including predicted grades increases R from .195 to .206. The model including both grade types, shows an effect of actual grades (beta=.153, t=14.8, $p=10^{-49}$) and an effect of predicted grades (beta=.077, t=7.42, $p=1.2 \times 10^{-13}$), so that the beta effect of predicted grades is 50% of that for actual grades, compared with the earlier regression for deciles, where the beta of .052 for predicted grades is only 19% of the beta of .269 for attained grades.

The present SJT test is administered at the time of graduation. There is also a separate SJT administered as a part of the U(K)CAT tests, which was only introduced in 2014, and none of that cohort have outcome variables in the present data set. However it is of interest that, for the 4286 applicants in 2014 with U(K)CAT SJT, there is a correlation of .145 with Actual A-levels and .127 with predicted A-levels (Z=1.28, p=0.192). Overall it is possible that SJT tests are behaving differently to academic outcomes, despite moderately strong correlations of SJT with other academic outcomes. SJT tests are, "designed to assess for key attributes … including commitment to professionalism, coping with pressure, effective communication, patient focus, and working effectively as part of a team" [28] [29].

Correlations of A-levels with GCSEs, U(K)CAT and BMAT. Without going into details, attained A-levels correlate more strongly with U(K)CAT and BMAT (r=.326 and .416) than do predicted A-levels (r=.272 and .326), suggesting that admissions tests are particularly assessing academic attainment. However GCSE grades show the reversed pattern and correlated *more strongly with predicted A-levels* (0.452) than with attained A-level grades (0.421), perhaps implying that teachers in part use GCSE grades to make predictions (as has been found in a previous study[19]).

Correlations of admissions tests with outcome measures. Neither of the two admissions tests, U(K)CAT and BMAT, has a strong prediction of EPM (r=.115 and .089 respectively), and both clearly correlate less with EPM than does attained A-levels, r=.297, despite A-levels showing range restriction due to a ceiling effect at A*. PSA and SJT though show a somewhat different picture. PSA correlates more highly with BMAT (r=.321) than with U(K)CAT (r=.238), and the correlation with BMAT is higher than that with attained A-levels (r=.306). In contrast U(K)CAT and BMAT both correlate similarly with SJT (r=.243 and .249), and both correlations are higher than with attained A-levels (r=.195). BMAT and U(K)CAT both show correlations with the two postgraduate outcomes (0.200 and 0.378 for MRCP(UK) Part 1 and 0.181 and 0.319 for MRCS Part A, but both are lower than the correlations with A-levels (0.421 and 0.358). Taken overall, BMAT has somewhat higher correlations with the five outcome measures (mean r = .269) than does U(K)CAT (mean r = .195) but both correlate less with outcomes than do attained A-levels (mean r=.315). U(K)CAT correlates at a similar level to predicted A-levels (mean r=.209) but BMAT at a somewhat higher level.

---

[10] Note that there are too few doctors who took both MRCP(UK) Part 1 and MRCS Part A to be able to calculate a correlation. Elsewhere we have looked at the relatively rare groups of doctors taking both MRCP(UK) and MRCGP, and shown high correlations between performance on the two assessments [26], making it likely that the same would also apply to MRCP(UK) Part 1 and MRCS Part A.

For peer review only - http://bmjopen.bmj.com/site/about/guidelines.xhtml

Reference List

1. Petch JA: *School estimates and examination results compared*. Manchester: Joint Matriculation Board; 1964.

2. Wilmut J: *Experiences of summative teacher assessments in the UK*. London: Qualifications and Curriculums Authority; 2011.

3. Murphy RJL: **Teachers' assessments and GCE results compared.** *Educational Research* 1979, **22:** 54-59.

4. Murphy RJL: **O-level grades and teachers' estimates as predictors of the A-level results of UCCA applicants.** *BJEP* 1981, **51:** 1-9.

5. Everett N, Papageorgiou J: *Investigating the Accuracy of Predicted A Level Grades as part of 2009 UCAS Admission Process*. London: Department for Business, Innovation and Skills; 2011.

6. Wyness G: *Predicted grades: Accuracy and impact. A report of University and College Union*. London: University and College Union (https://www.ucu.org.uk/media/8409/Predicted-grades-accuracy-and-impact-Dec-16/pdf/Predicted_grades_report_Dec2016.pdf); 2016.

7. Wyness G: *Rules of the game: Disadvantaged students and the university admissions process*. London: The Sutton Trust; 2017.

8. Murphy R, Wyness G: *Minority Report: the impact of predicted grades on university admissions of disadvantaged groups*. London: **Centre for Education Policy and Equalising Opportunities (CEPEO) Working Paper No. 20-07, UCL Institute of Education** https://EconPapers.repec.org/RePEc:ucl:cepeow:20-07; 2020.

9. UCAS: *End of cycle report 2017: Qualifications and competition*. Cheltenham: UCAS [https://www.ucas.com/data-and-analysis/ucas-undergraduate-releases/ucas-undergraduate-analysis-reports/2017-end-cycle-report]; 2017.

10. Gill T, Rushton N: *The accuracy of forecast grades for OCR Alevels: Statistics Report Series No 26*. Cambridge: Cambridge Assessment [https://www.cambridgeassessment.org.uk/our-research/all-published-resources/statistical-reports/150215-the-accuracy-of-forecast%20-grades-for-ocr-a-levels-in-june-2012.pdf/]; 2011.

11. Gill T, Chang Y: *The accuracy of forecast grades for OCR A levels in June 2012*. Cambridge: Cambridge Assessment Statistics Report Series No.64; 2013.

12. Gill T, Benton T: *The accuracy of forecast grades for OCR Alevels in June 2014: Statistics Report Series No 90*. Cambridge: Cambridge Assessment [https://www.cambridgeassessment.org.uk/Images/241261-the-accuracy-of-forecast-grades-for-ocr-a-levels-in-june-2014.pdf]; 2015.

13. UCAS: *Factors associated with predicted and achieved A level attainment, August 2016*. Cheltenham: UCAS: https://www.ucas.com/file/71796/download?token=D4uuSzur; 2016.

For peer review only

For peer review only - http://bmjopen.bmj.com/site/about/guidelines.xhtml

14. McManus IC, Woolf K, Dacre J: **The educational background and qualifications of UK medical students from ethnic minorities.** *BMC Medical Education* 2008, **8: 21 (http://www.biomedcentral.com/1472-6920/8/21)**.

15. Gill T: **Methods used by teachers to predict final Alevel grades for their students.** *Research Matters (UCLES)* 2019, 33-42.

16. Child S, Wilson F: *An investigation of A level teachers' methods when estimating student grades*. Cambridge: Cambridge Assessment (Unpublished document, October 2015); 2015.

17. Lumb AB, Vail A: **Applicants to medical school: the value of predicted school leaving grades.** *Med Educ* 1997, **31:** 307-311.

18. Richardson PH, Winder B, Briggs K, Tydeman C: **Grade predictions for school-leaving examinations: do they predict anything?** *Med Educ* 1998, **32:** 294-297.

19. McManus IC, Richards P, Winder BC, Sproston KA, Styles V: **Medical school applicants from ethnic minorities: identifying if and when they are disadvantaged.** *Brit Med J* 1995, **310:** 496-500.

20. Boliver V: **How fair is access to more prestigious universities?** *British Journal of Sociology* 2013, **64:** 344-364.

21. Gill TRCV: *Predictive validity of level 3 qualifications: Extended Project, Cambridge Pre-U, International Baccalaureate,BTEC Diploma*. Cambridge: Cambridge Assessment: Cambridge Assessment Research Report; 2014.

22. Thomson D: *Moderating teaching judgments in 2020 [Blog post, 25th March 2020]*. London: FFT Educational Lab: https://ffteducationdatalab.org.uk/2020/03/moderating-teacher-judgments-in-2020/ (accessed 16th April 2020); 2020.

23. McManus IC, Dewberry C, Nicholson S, Dowell JS, Woolf K, Potts HWW: **Construct-level predictive validity of educational attainment and intellectual aptitude tests in medical student selection: Meta-regression of six UK longitudinal studies.** *BMC Medicine* 2013, **11:243:** doi:10.1186/1741-7015-11-243.

24. Meng X-L, Rosenthal R, Rubin DB: **Comparing correlated correlation coefficients.** *Psychol Bull* 1992, **111:** 172-175.

25. McManus IC, Dewberry C, Nicholson S, Dowell J: **The UKCAT-12 study: Educational attainment, aptitude test performance, demographic and socio-economic contextual factors as predictors of first year outcome in a collaborative study of twelve UK medical schools.** *BMC Medicine* 2013, **11 :244:** doi:10.1186/1741-7015-11-244.

26. Wakeford R, Denney ML, Ludka-Stempien K, Dacre J, McManus IC: **Cross-comparison of MRCGP & MRCP(UK) in a database linkage study of 2,284 candidates taking both examinations: Assessment of validity and differential performance by ethnicity.** *BMC Medical Education* 2015, **15**.

27. McManus IC, Woolf K, Dacre J, Paice E, Dewberry C: **The academic backbone: Longitudinal continuities in educational achievement from secondary school and medical school to MRCP(UK) and the Specialist Register in UK medical students and doctors.** *BMC Medicine* 2013, **11:242:** doi:10.1186/1741-7015-11-242.

For peer review only - http://bmjopen.bmj.com/site/about/guidelines.xhtml

28. Patterson F, Zibarras L, Ashworth V: **Situational judgement tests in medical education and training: Research, theory and practice: AMEE Guide No. 100.** *Med Teach* 2016, **38:** 3-17.

29. McManus IC, Harborne A, Smith D, Horsfall HL, Devine O: **Exploring UK medical school differences: The *MedDifs* study of selection, teaching, student and F1 perceptions, postgraduate outcomes, and fitness to practise .** *BMC Medicine* 2019, **In press**.

For peer review only

Supplementary Table 1: Comparison of predicted and forecasted grades in 2009 and 2012.

Estimated grades			Max grade	Over-predicted	Accurate	Under-predicted	Population	Source
**Predicted**	**October**	**2009**	**A**	**42%**	**52%**	**7%**	**UCAS**	Everett and Papageorgiou (2011)
**Forecasted**	**May**	**2009**	**A**	**33%**	**55%**	**12%**	**OCR**	Gill and Rushton (2011)
Forecasted-Predicted				*-9%*	*3%*	*5%*		
**Predicted**	**October**	**2012**	**A***	**68%**	**20%**	**12%**	**UCAS**	UCAS (2017)
**Forecasted**	**May**	**2012**	**A***	**39%**	**48%**	**13%**	**OCR**	Gill and Chang (2013)
Forecasted-Predicted				*-30%*	*29%*	*1%*		

Supplementary Table 2: Comparison of predicted and attained A-level grades in medical school applicants, 2010-2018

a) Counts of number of cases

		Attained Alevel grades						
		E	D	C	B	A	A*	*Total*
Predicted Alevel grades	E	**200**	35	10	5	0	0	*255 (0%)*
	D	235	**610**	155	35	10	0	*1045 (0%)*
	C	635	1220	**2110**	505	95	5	*4570 (2%)*
	B	635	2095	4755	**7355**	1695	175	*16715 (7%)*
	A	430	1925	8785	35640	**61950**	12655	*121390 (51%)*
	A*	50	135	635	6025	42815	**43395**	*93060 (39%)*
	Total	*2185*	*6020*	*16450*	*49570*	*106570*	*56235*	*237030*
		(1%)	*(3%)*	*(7%)*	*(21%)*	*(45%)*	*(24%)*	

b) Percentages within predicted grades

		Attained Alevel grades						
		E	D	C	B	A	A*	*Total*
Predicted Alevel grades	E	**79%**	14%	..	..	..	..	*100%*
	D	23%	**58%**	15%	3%	..	..	*100%*
	C	14%	27%	**46%**	11%	2%	..	*100%*
	B	4%	13%	28%	**44%**	10%	1%	*100%*
	A	0%	2%	7%	29%	**51%**	10%	*100%*
	A*	0%	0%	1%	7%	46%	**47%**	*100%*
	Total	*1%*	*3%*	*7%*	*21%*	*45%*	*24%*	*100%*

b) Percentages within predicted grades

		Attained Alevel grades						
		E	D	C	B	A	A*	*Total*
Predicted Alevel grades	E	**9%**	1%	..	..	..	..	*0%*
	D	11%	**10%**	1%	0%	..	..	*0%*
	C	29%	20%	**13%**	1%	0%	..	*2%*
	B	29%	35%	29%	**15%**	2%	0%	*7%*
	A	20%	32%	53%	72%	**58%**	23%	*51%*
	A*	2%	2%	4%	12%	40%	**77%**	*39%*
	Total	*100%*	*100%*	*100%*	*100%*	*100%*	*100%*	*100%*

Supplementary Table 3: Comparison of predicted and forecasted A-level grades in medical school applicants, 2010-2018

Subject	N	Mean Predicted	Mean Actual	Actual minus Predicted	r (Pearson)
Chemistry	62815	10.35	9.37	-0.98	0.623
Biology	61190	10.59	9.78	-0.82	0.632
Maths & Stats	54635	10.79	9.77	-1.02	0.600
Physics & Engineering	13870	10.67	9.52	-1.15	0.635
General Studies & Critical Thinking	6785	9.66	7.70	-1.96	0.534
Modern Languages	6720	10.59	9.74	-0.85	0.571
Psychology	6190	10.19	9.12	-1.07	0.631
Geography	4015	10.84	9.95	-0.89	0.538
History	3850	10.48	9.49	-0.99	0.546
English Literature & Language	3815	10.32	9.52	-0.80	0.681
Further Maths	2950	11.07	9.62	-0.80	0.681
Economics & Business Studies	2765	10.36	9.47	-0.89	0.577
Religious Studies	1890	10.45	9.40	-1.05	0.626
Art & Design	1035	10.60	10.03	-0.57	0.681
Latin & Classical Studies	675	10.74	9.65	-1.09	0.576
Music	640	10.49	9.51	-0.97	0.567
Sociology	525	9.51	8.49	-1.02	0.679
Computer Studies & ICT	475	9.89	8.82	-1.06	0.704
Physical Education	470	10.61	9.81	-0.80	0.610
Government & Politics	380	10.07	9.16	-0.91	0.656
Theatre Studies & Drama	260	10.14	9.02	-1.11	0.624
Science -- Misc & General	260	8.30	7.24	-1.06	0.821
Law	190	9.42	8.55	-0.87	0.766
Philosophy	155	10.37	9.06	-1.32	0.639
Classical Greek	115	10.90	9.98	-0.92	0.463
Media Studies	75	8.03	7.25	-0.78	0.798

Supplementary Table 4: Comparison of predicted and attained EPQ grades in medical school applicants, 2010-2018

a) EPQ: Counts of number of cases

		Attained EPQ grade						
		E	D	C	B	A	A*	*Total*
	E	**5**	0	0	0	0	0	*5 (0%)*
Predicted	D	0	**15**	0	0	0	0	*20 (0%)*
EPQ	C	10	10	**120**	15	5	0	*160 (2%)*
grade	B	15	40	90	**355**	100	30	*625 (7%)*
	A	40	135	405	920	**1970**	1150	*4620 (49%)*
	A*	15	35	125	375	940	**2420**	*3915 (42%)*
	Total	*85*	*240*	*740*	*1670*	*3010*	*3605*	*9345*
		(1%)	*(3%)*	*(8%)*	*(18%)*	*(32%)*	*(39%)*	

b) EPQ: Percentages within predicted grades

		Attained EPQ grade						
		E	D	C	B	A	A*	*Total*
	E	**..**	..	..	..	..	..	*...*
Predicted	D	..	**..**	..	..	..	..	*...*
EPQ	C	..	..	**46%**	..	..	..	*100%*
grade	B	..	13%	28%	**44%**	10%	1%	*100%*
	A	0%	2%	7%	29%	**51%**	10%	*100%*
	A*	..	0%	1%	7%	46%	**47%**	*100%*
	Total	*1%*	*3%*	*7%*	*21%*	*45%*	*24%*	*100%*

Supplementary Table 5: Comparison of predicted and forecasted SQA Highers and SQA Advanced Highers in medical school applicants, 2010-2018

a) SQA Advanced Highers: Counts of number of cases

		Attained SQA Highers							
		D7	C6	C5	B4	B3	A2	A1	*Total*
	D	**90**	0	0	0	0	0	0	95 (0.6%)
Predicted	C	20	**210**	**220**	15	10	10	0	485 (3.3%)
SQA	B	95	140	190	**455**	**490**	305	30	1700 (11.6%)
Highers	A	255	495	905	1405	2010	**5335**	**1955**	12360 (84.4%)
	Total	**465**	**845**	**1320**	**1875**	**2510**	**5645**	**1985**	14640 (100%)
	Total	*3.2%*	*5.8%*	*9.0%*	*12.8%*	*17.2%*	*38.6%*	*13.6%*	

b) SQA Advanced Highers: Percentages within predicted grades

		Attained SQA Highers							
		D7	C6	C5	B4	B3	A2	A1	*Total*
	D	**97%**	..	..	..	..	..	..	100%
Predicted	C	..	**43%**	**45%**	..	..	..	..	100%
SQA	B	6%	8%	11%	**27%**	**29%**	18%	2%	100%
Highers	A	2%	4%	7%	11%	16%	**43%**	**16%**	100%
	Total	*3.2%*	*5.8%*	*9.0%*	*12.8%*	*17.2%*	*38.6%*	*13.6%*	

Supplementary Table 6: Correlation matrix of selection measures, undergraduate outcome measures, and postgraduate outcome measures (separated by grey lines for clarity). Cells indicate Pearson correlation and N. (NB presented as figure 3 in the main paper).

		Selection measures applicants					Undergraduate outcome measures			Postgraduate outcome measures	
		GCSE grades	Predicted Alevels	Alevel grades	UKCAT	BMAT	EPM	SJT	PSA	MRCP(UK) Part 1	MRCS Part A
	GCSE grades	**1**	**0.452**	**0.421**	**0.265**	**0.223**	**0.180**	**0.190**	**0.201**	**0.212**	**0.173**
			22150	22150	22145	4935	12230	12185	12265	890	430
	Predicted A-level grades	**0.452**	**1**	**0.585**	**0.272**	**0.326**	**0.198**	**0.160**	**0.226**	**0.283**	**0.181**
**Selection measures**		22150		22955	22520	5225	12560	12515	12600	910	440
**in all applicants**	Attained A-level grades	**0.421**	**0.585**	**1**	**0.326**	**0.416**	**0.297**	**0.195**	**0.306**	**0.421**	**0.358**
		22150	22955		22520	5225	12560	12515	12600	910	440
	UKCAT total	**0.265**	**0.272**	**0.326**	**1**	**0.483**	**0.115**	**0.243**	**0.238**	**0.200**	**0.181**
		22145	22520	22520		5080	12385	12340	12420	900	435
	BMAT sections 1 and 2	**0.223**	**0.326**	**0.416**	**0.483**	**1**	**0.089**	**0.239**	**0.321**	**0.378**	**0.319**
		4935	5225	5225	5080		4850	4840	4875	450	240
	UKFPO EPM decile	**0.180**	**0.198**	**0.297**	**0.115**	**0.089**	**1**	**0.319**	**0.470**	**0.509**	**0.535**
**Undergraduate**		12230	12560	12560	12385	4850		12515	12505	905	440
**outcome measures**	UKFPO SJT score	**0.190**	**0.160**	**0.195**	**0.243**	**0.239**	**0.319**	**1**	**0.346**	**0.351**	**0.274**
		12185	12515	12515	12340	4840	12515		12475	905	435
	PSA score	**0.201**	**0.226**	**0.306**	**0.238**	**0.321**	**0.470**	**0.346**	**1**	**0.500**	**0.483**
		12265	12600	12600	12420	4875	12505	12475		910	440
	MRCP(UK) Part 1	**0.212**	**0.283**	**0.421**	**0.200**	**0.378**	**0.509**	**0.351**	**0.500**	**1**	**…**
**Postgraduate**		890	910	910	900	450	905	905	910		10
**outcome measures**	MRCS Part A	**0.173**	**0.181**	**0.358**	**0.181**	**0.319**	**0.535**	**0.274**	**0.483**	**…**	**1**
		430	440	440	435	240	440	435	440	10	

Supplementary figure 1: Mean EPM *revDeciles* (95% CI) in relation to actual A-level grades (green) and predicted A-level grades (red), state sector schooling shown in darker colours and private sector schooling in paler colours.

a.)

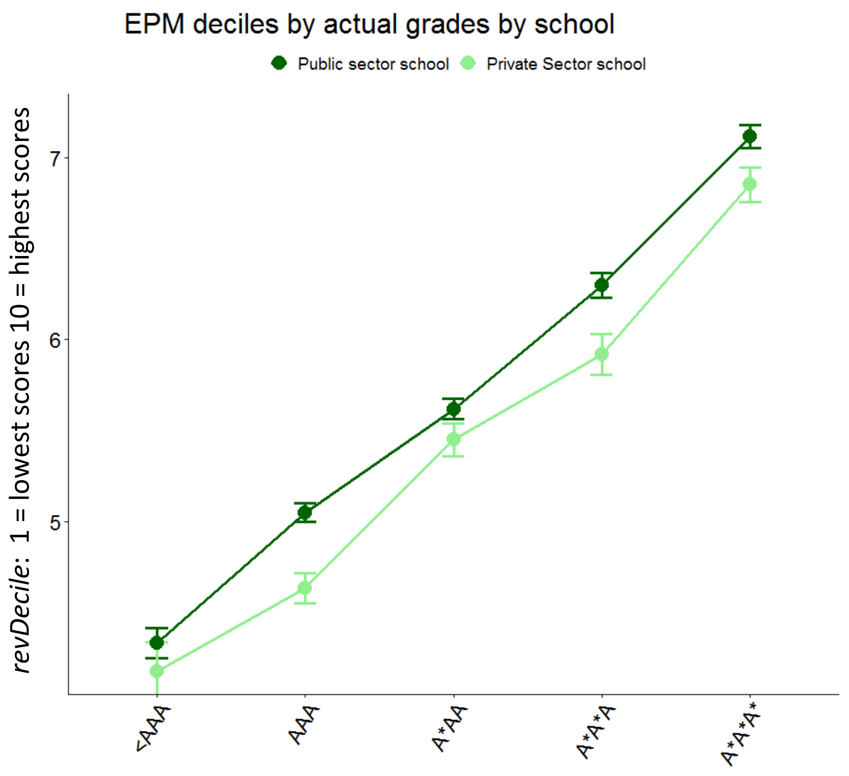

b.)

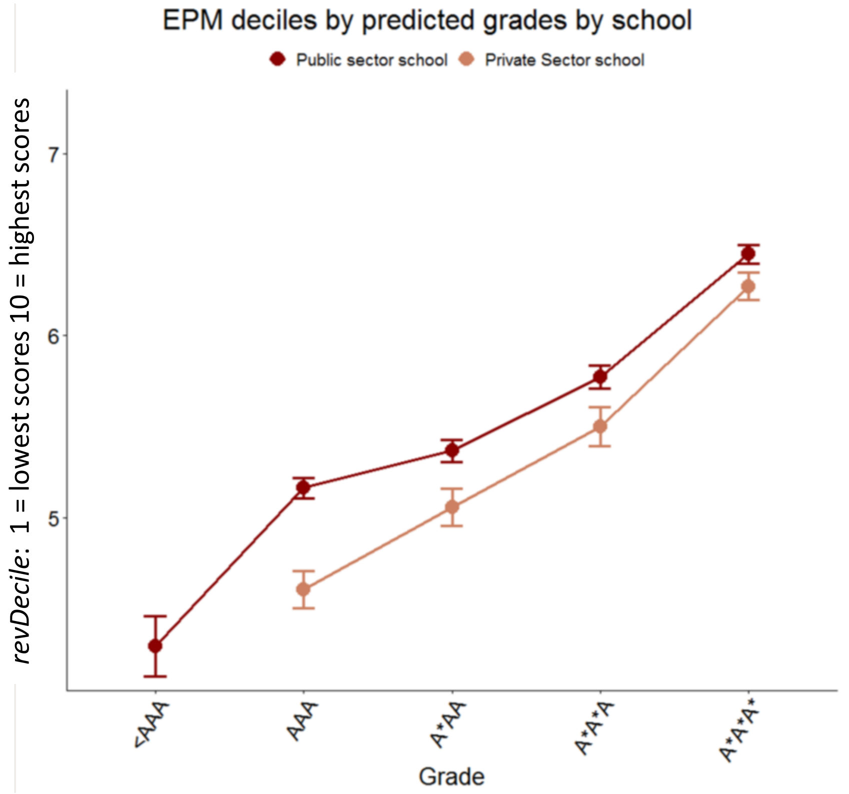

Supplementary figure 2: Mean PSA mark in relation to actual A-level grades (green) and predicted A-level grades (red)

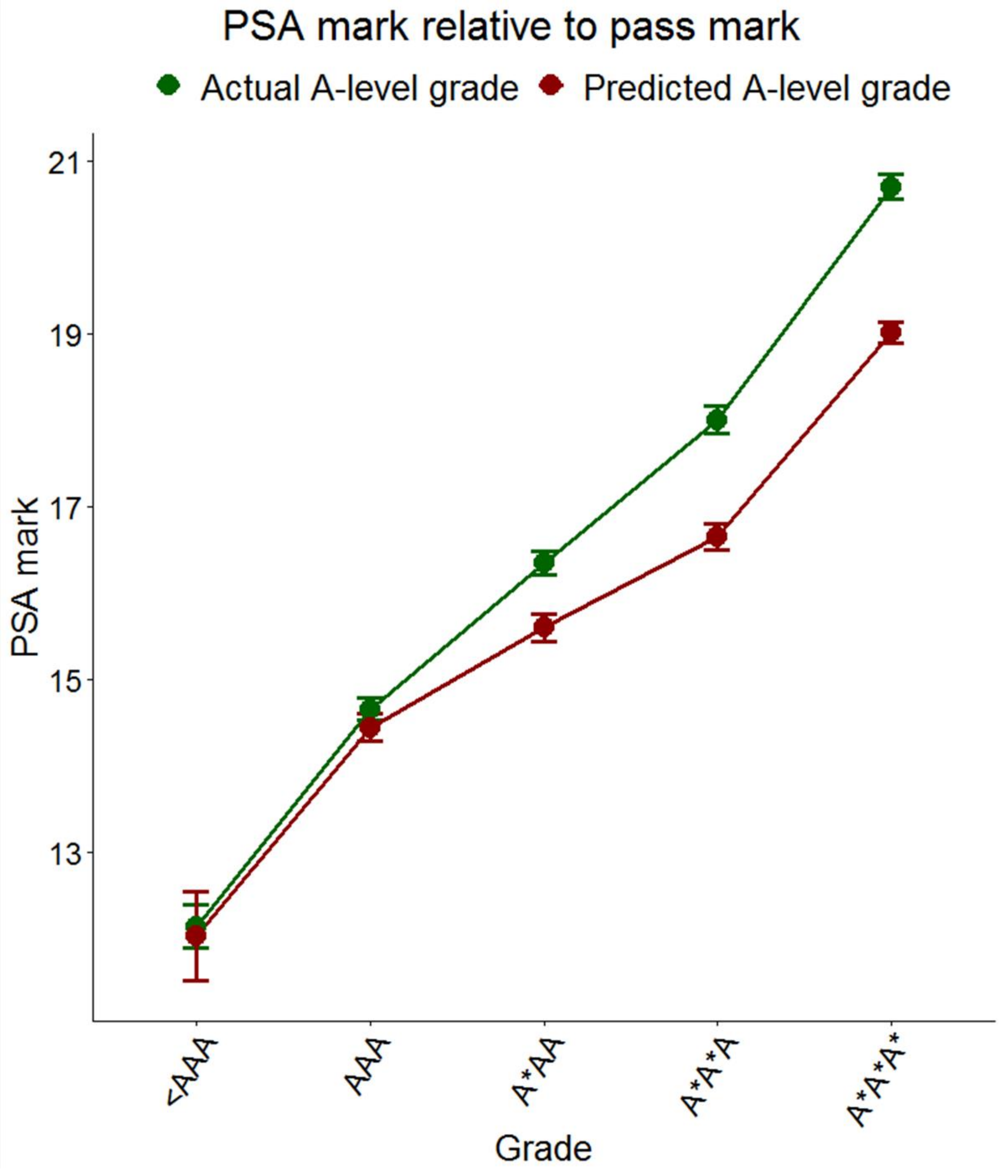

Supplementary figure 3: Mean SJT mark in relation to actual A-level grades (green) and predicted A-level grades (red)

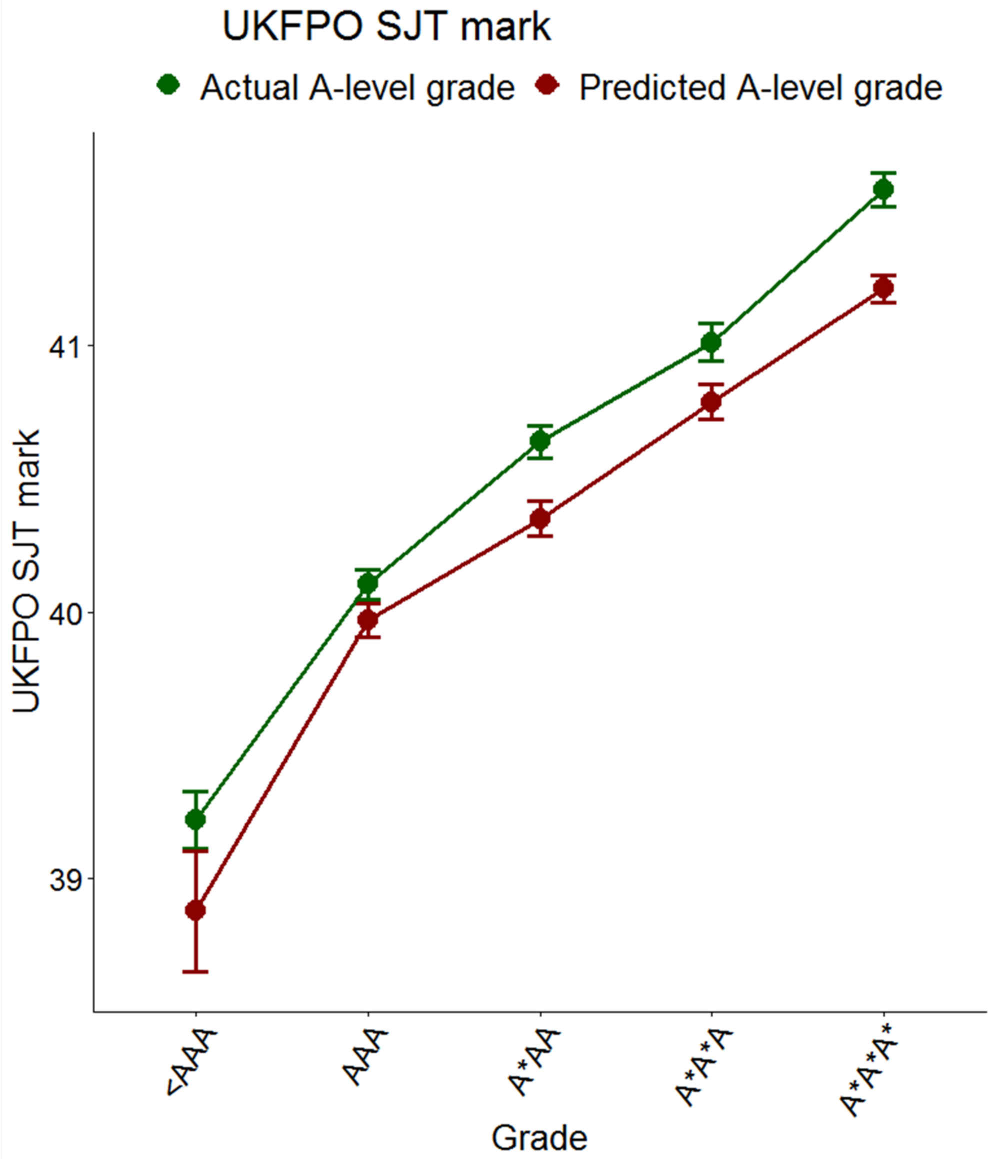

Supplementary figure 4: Mean MRCP(UK) Part 1 mark in relation to actual A-level grades (green) and predicted A-level grades (red)

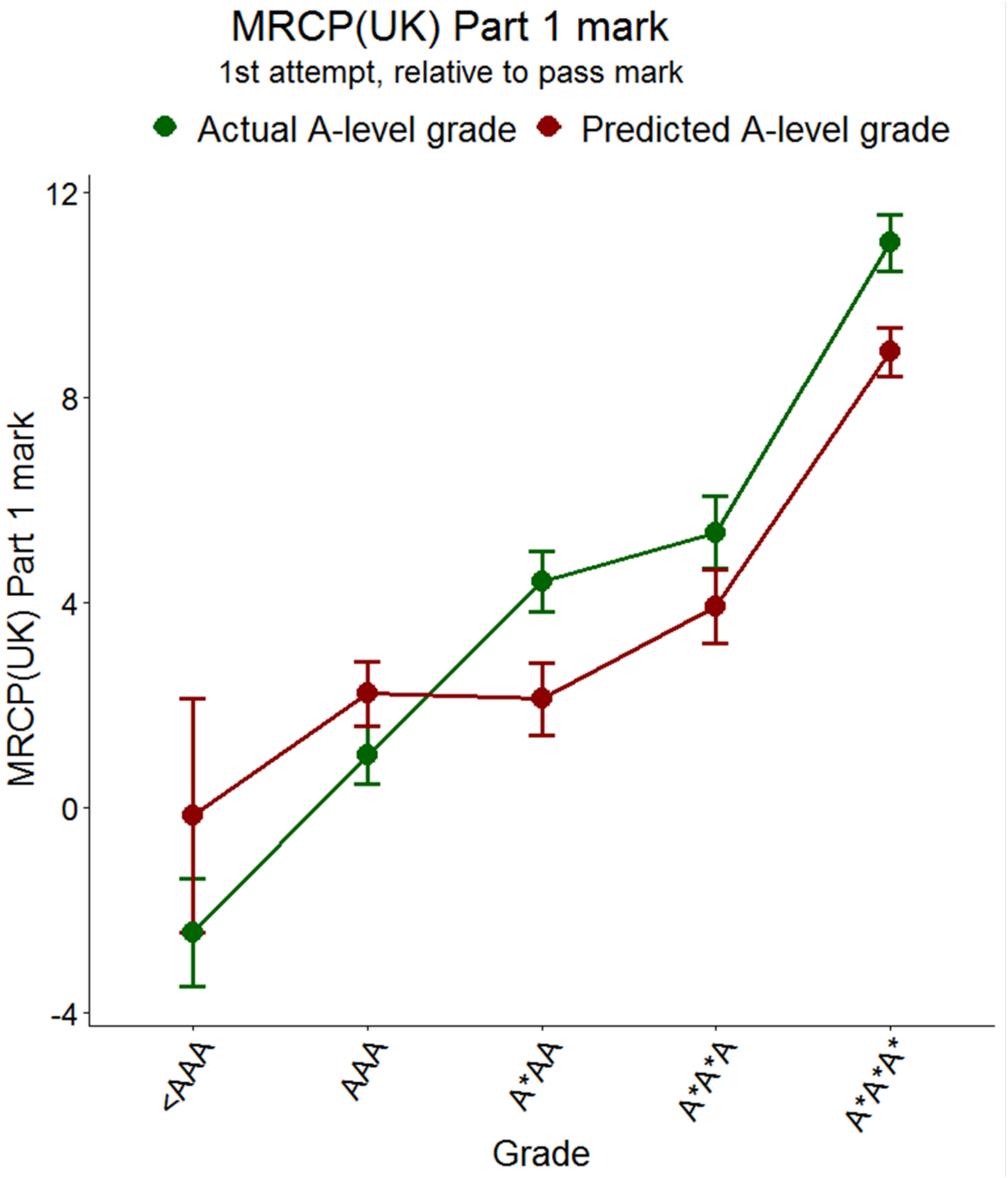

Supplementary figure 5: Mean MRCS Part A mark in relation to actual A-level grades (green) and predicted A-level grades (red)

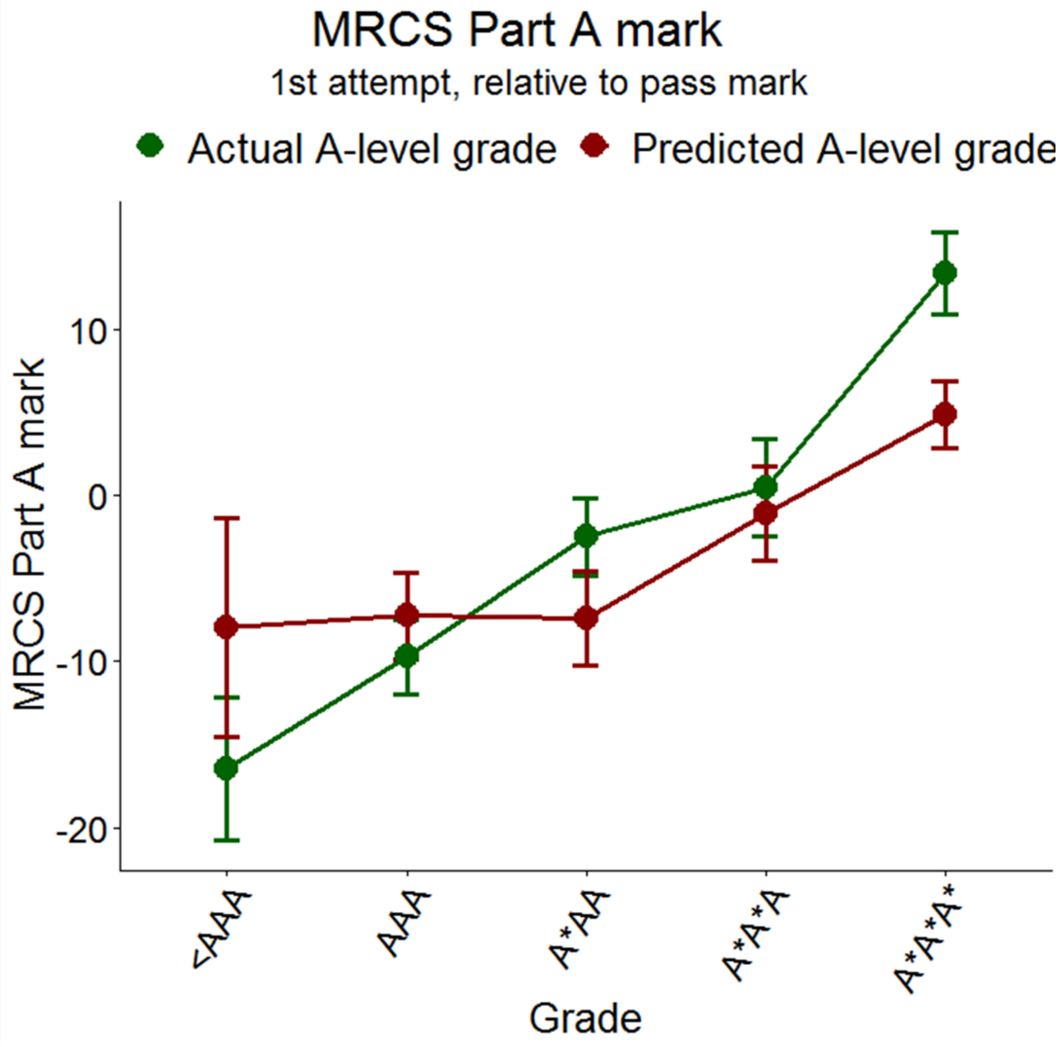

Appendix:

Are independent (private sector) schools more accurate in their A-level predictions?

A recurrent suggestion in the literature is that schools in the private sector (Independent Schools) are more accurate in their A-level predictions than those in the state sector. That suggestion raises many issues, not least concerned with social equity, but a key one to be resolved is whether the differences mainly are secondary to differences in overall attainment level, and as a result may be artefacts due to the ordinal nature of A-level grades and to A* being the ceiling for A-level grades, and hence is a level beyond which candidates cannot reach. This appendix looks in detail at that question. The description is lengthy, technical, and partly didactic, and therefore has not been included in the main text or the main supplementary text. The conclusion is actually relatively simple: *Independent schools are not actually more accurate in their predictions, but they look that way because of having higher attained grades.*

The data. Appendix table 1 shows, in a similar format to those in the main paper, the A-level grades in P89 for applicants from state schools (defined as Comprehensives, Academies, Sixth Form Colleges, Tertiary Colleges and Technical Colleges) and private schools (defined as Independent Schools and Grammar Schools), with results restricted to first A-level attempts, duplicates removed, and where both A-level grades and predicted grades were available. Results are at the subject level, and therefore contain multiple subjects from individual applicants.

A simple glance at Appendix table 1 suggests that indeed Private Sector schools are more accurate, 53.3% of predictions being accurate compared to 45.5% of predictions from State Sector schools. Private schools also show a lower rate of over-prediction (40.5% compared with 48.0%), but not of under-prediction (6.2% vs 6.4%). These differences need however to be put into the context of higher overall attainment in private schools, where 29% of grades were A* compared with 20% in state schools, a finding that reflects most private schools being selective and therefore inevitably taking higher ability entrants. Since attained A* grades are more frequent in private schools, it is not surprising that predicted A* grades are also more frequent in private schools, 48% vs 35%. The question therefore is whether the differences in accuracy are secondary to differences in overall performance. That question is best answered using *polychoric correlations*, which need description.

Polychoric and tetrachoric correlations. Polychoric and tetrachoric correlations are used frequently in psychometrics when dealing with binary and ordinal data. The need for them is shown by a simple 2x2 association table of the sort often tested using a chi-square test. Consider Appendix figure 1, which is a simple association table for characteristics P and Q in 100 individuals. 80% of cases have P present but only 50% of cases have Q present, meaning that the *marginal proportions* are not the same (80% vs 50%). A chi-square test is highly significant (chi-squared = 25, 1 df, p=0.0000006) meaning that there is an association between P and Q. But what is the size of that association? Often in this situation a Pearson or Spearman correlation is calculated, and these give r_p=0.5 and r_s=0.5, which suggests a moderately strong association.

However there is a problem in using the Pearson correlation, as a careful look at the table shows because the number of cases in which P is absent but Q is present, in the top right-hand corner, is zero. In other words the association could not be any stronger, but the correlation is still only 0.5, whereas a perfect correlation is usually taken as being 1. The problem arises because the marginal

For peer review only - http://bmjopen.bmj.com/site/about/guidelines.xhtml

proportions of P and Q are not the same, one being 0.5 and the other 0.8. If these two marginal proportions had been identical than all of the cases could have been on the diagonal and then the Pearson correlation would indeed have been 1. So what does one do in the case where the marginal proportions are not the same? The answer is another correlation developed by Pearson, called the *tetrachoric* or *polychoric* correlation for 2x2 or for larger tables respectively.

The tetrachoric correlation assumes that the data actually come from a bivariate normal distribution with some underlying correlation, and asks if that distribution were divided horizontally and vertically, what the correlation would have to be to create the contingency table that has been found. The lower part of Appendix figure 1 shows that diagrammatically[1], the four quadrants containing the proportions of data in the contingency table. The calculation is easily carried out in the *R* function *polychor()* in the *polycor* library, and for the table in Appendix figure 1 it gives the answer that r_t=0.994, which effectively is r_t=1. The tetrachoric correlation therefore corresponds to our intuitive sense of what the correlation should be. The underlying bivariate normal distribution is assumed to have means of zero and standard deviations of one. *polychor()* then tells us that the thresholds for cutting the distribution need to be at 0.842 for P and 0 for Q. The threshold for Q at zero tells us that the cutting point is 0 standard deviations from the mean, and therefore 50% of cases are above the threshold and 50% below. The threshold for P is 0.842 standard deviations below the mean, and hence 20% of cases are below the threshold and 80% of cases above it. The marginal proportions of P and Q are then replicated.

For a 2x2 table it is always possible to fit the tetrachoric correlation and the marginal proportions exactly. If the table is larger, giving a polychoric correlation, the marginal proportions and the cell frequencies cannot always be fitted exactly as the normal distribution may not be entirely appropriate, and in that case maximum likelihood estimates of the correlation and thresholds are found. The polychoric calculation for an *m x n* table also provides a set of *(m-1)* and *(n-1)* thresholds for each of the variables, and it is possible to see if step sizes between the levels are equal. Polychoric correlations therefore are used for data where both measures are *ordinal* and for which it seems reasonable to assume an underlying latent distribution which is normal.

Polychoric correlations for A-level grades. A-level grades are certainly at least ordinal in nature, but it is not clear that they are *equal interval,* the step from, say, D to C not necessarily being the same size as the step from B to A. Polychoric calculations allow the direct estimation of the step sizes between grades. If step sizes are not equal then many conventional statistics are not optimal. Equal interval scales are measures such as length, where the increments are identical in size (so the difference between, say, 2 cms and 3 cms is the same length as the difference between 10 cms and 11 cms). A-levels are often scored on a simple basis of allocating points, such as A*=12, A=10, B=8, C=6, D=4 and E=2 (and indeed we have done this elsewhere here), but that can sometimes be misleading in situations such as calculating correlations between actual and predicted grades, partly because marginal proportions are not the same, and partly because the data are *censored*, grades above A* not being possible, however capable is a candidate, and hence over-prediction is not possible for estimated grades of A*. In the case of a high ability group such as applicants to medical school the latter is problematic as state and private schools predict an A* grade for 35% and 48% of exams. To put it another way, were a grade of A** available then many examinees might have merited it [1], albeit probably more at private than state schools. There is also potentially a problem of computing total A-level scores (so that, say, AAA with 30 points is regarded as equivalent to A*AB or A*A*C,

[1] The correlation is actually drawn at 0.9 to make things pedagogically clearer, as a correlation of 1 is effectively a straight line.

which may not be exactly the case, although the approximation is probably good enough for most purposes).

Fitting polychoric correlations to A-level grades from state and private schools. The key question at present is whether private schools are more accurate in their predictions (53.3%) than state schools (45.5%) – see Appendix table 1. Accuracy can be considered in two ways, as the presence of systematic error (technically, 'bias'), equivalent to rates of A* etc being different in two groups, and random error, in terms of the correlation or lack of correlation between two sets of scores. Although the overall accuracy of private schools is *higher* than state schools, the correlation of predicted and actual grades is *lower* in private schools, with Pearson correlations of 0.635 in state schools and 0.552 in private schools (Appendix table 1), with a similar pattern for Spearman correlations. That suggests a potential problem in interpreting the data. Calculating the polychoric correlations suggests a very different picture, since the polychoric correlations in state schools (r_t= 0.717) and private schools (r_t= 0.678) are far more similar, particularly in comparison with the differences between the Pearson (or Spearman) correlations.

Interpreting the polychoric correlations is helped by a diagram. Appendix figure 2.a may look complex, but it summarises a lot of information about state sector applicants. The axes are on a normal distribution for the underlying latent scale, and so the units are standard deviations, from -4 to +4 SDs. Note these are not SDs for the raw data, but for the latent distribution. The polychoric correlation for the state sector is 0.717, and that is shown by the blue ellipse which is plotted to cover 99.9% of the data, which is reasonable given the large sample sizes. The dashed blue and yellow line on the diagonal is the line of equality for attained grades on the horizontal axis and predicted grades on the vertical axis. The vertical and horizontal lines show the thresholds separating the various A-level grades for attained and predicted grades. Appendix table 2 summarises the various thresholds and their intervals for state and private schools. As an example, for attained grades, the threshold separating A from A* (Appendix table 2, row 4, column A:A*) is 0.83, and so the vertical line in Appendix figure 2.a separating A from A* is at 0.83. Similarly the horizontal line for predicted grades separating A from A* is at 0.39 (row 2 in Appendix table 2). The intersection of these two lines is shown by a large red circle, which is *below* the blue-yellow dashed line, which indicates that the threshold for attained grades is higher than the threshold for predicted grades, so that it is easier to be predicted an A* than to attain an A*. The other vertical and horizontal lines show the thresholds between B and A (B:A), C and B (C:B), D and C (D:C) and E and D (E:D). As for A*:A, all of the intersections, shown as red dots, are below the dashed blue-yellow line of equality, showing that predicted grades are always more generous than attained grades. Row 6 of Appendix table 2 shows that on average the threshold for attained grades is 0.73 SDs lower than for predicted grades. The coloured boxes in Appendix figure 2.a are equivalent to the coloured boxes in appendix table 1, with grey indicating accuracy, green and blue indicating under-estimation, and red and yellow over-estimation. More of the figure is red or yellow than is blue or green, indicating the overall over-estimation by predicted grades. It is also clear from the figure that the differences between the thresholds are not equal. The width of D, from E:D to D:C, is smaller than the width of A (from B:A to A:A*), these values being shown in row 10 of Appendix table 2 for predicted grades and row 12 for attained grades. The widths of E and A* cannot be calculated as they stop either at minus infinity or plus infinity. It is clear that the scale is not equal interval, with less change being required to move from D to C than from B to A. Statistical analyses should take care therefore in assuming that the usual A* to E scale of grades is equal interval, and can be averaged.

The key question for this appendix is the extent to which state and private sector predictions are different. Appendix figure 2.b shows an equivalent plot to Appendix figure 2.a but for private sector

For peer review only - http://bmjopen.bmj.com/site/about/guidelines.xhtml

A-levels. At a glance it is not easy to see any obvious difference, but it is important to remember that the latent scales for both graphs each have a mean of zero and SD of one. However looking carefully shows that the threshold for attained grades at A* is at 0.55 for private sector students compared with 0.83 for state sector students (see rows 4 and 5 of table Appendix table 2). The threshold is lower for private sector students and hence more of these students will attain an A*, as is the case in Appendix table 2. All of the thresholds for the private sector students are actually moved to the left compared with state sector students (and compare the sizes of the A*A* boxes and the EE boxes in the two figures. Appendix figure 3 summarises the thresholds more clearly for attained and predicted grades in state and private sector schools. All thresholds are shown on the same horizontal scale. Attained grades for private schools are to the right of predicted grades, shown by the thin blue diagonal lines (meaning an attained A* is harder to get than a predicted A*), and the same pattern is seen for state schools, and shown by the thin diagonal red lines. Private school attained grades are also to the left of state school attained grades, shown by a thin purple line (with thresholds lower for private school students meaning that they get more A* grades). Similarly, private school predicted grades are also to the left of state school predicted grades, also shown by a thin purple line. A key feature of Appendix figure 3 is that the blue diagonal lines are parallel, the red diagonal lines are parallel and the purple diagonal lines are nearly parallel, meaning that the relationships of grade boundaries are the same in private and state schools, and for attained and predicted grades, but are merely slid along relative to one another. The state and private schools are therefore handling predicted grades in a way that is similar, and they are similar related in each case to attained grades.

The widths of the boxes in Appendix figure 2 are therefore very similar in state and private sector students, and are shown in rows 9 to 16 of Appendix table 2, particularly in rows 10 and 11, which compare predicted grades in state and private schools, and rows 12 and 13 which compare attained grades in private schools. The main difference between the two types of school is shown in the mean columns of rows 1 and 2 and rows 4 and 5, their mean differences being shown in the final column. Overall the state schools have thresholds which for predicted grades are on average are 0.47 SDs higher and for attained grades are 0.42 grades higher than for private sector schools (meaning that higher grades are harder to attain). These values are very similar and suggest that predictions in the two types of school are being carried out in a similar way, but the overall ability of private school students is higher, and that is reflected in the attained and predicted ways to a similar extent.

The private schools students are therefore about 0.44 SDs higher on the latent scale than the state school students. As a result it is possible to plot state and private schools on the same graph (Appendix figure 4), with the only difference being that the private schools are further along the diagonal towards the top right corner. That difference accounts for all of the differences in the private and state school students, with all other differences in Appendix table 1 being artefacts of the artificial ceiling of the range at A*. To put it another way, were attained grades to be the same in state and private schools then the accuracy and the degree of over-estimation would be the same in the two types of schools.

Conventional statistics comparing attained and predicted grades at A-level are inherently misleading, and suggest differences between groups which are probably not present, meaning that great care must be taken in interpretation.

1. McManus IC, Woolf K, Dacre JE: **Even one star at A level could be "too little, too late" for medical student selection.** *BMC Medical Education* 2008, **8:16** **(http://www.biomedcentral.com/1472-6920/8/16)**.

For peer review only - http://bmjopen.bmj.com/site/about/guidelines.xhtml

Appendix table 1. Predicted vs Attained A-level grades in applicants from a) State Sector schools (non-Private schools) and b) Independent (Private sector) schools.

a) State Sector: Counts of number of cases

		Attained Alevel grades						
		E	D	C	B	A	A*	*Total*
	E	**140**	30	5	5	0	0	*180 (0%)*
Predicted	D	210	**420**	125	20	5	0	*780 (1%)*
Alevel	C	535	1050	**1545**	400	55	5	*3600 (2%)*
grades	B	500	1735	3910	**5190**	1090	115	*12540 (9%)*
	A	270	1330	6250	24310	**36915**	7425	*76495 (53%)*
	A*	25	75	395	3950	24725	**21410**	*50580 (35%)*
	Total	*1680*	*4645*	*12230*	*33870*	*62785*	*28960*	*144175*
		(1%)	*(3%)*	*(8%)*	*(23%)*	*(44%)*	*(20%)*	

	Prediction:			Correlations:		
Under	Accurate	Over		Pearson	Spearman	Polychoric
6.4%	**45.5%**	48.0%		0.635	0.590	0.717

a) Private Sector: Counts of number of cases

		Attained Alevel grades						
		E	D	C	B	A	A*	*Total*
	E	**15**	0	0	0	0	0	*15 (0%)*
Predicted	D	15	**55**	15	5	0	0	*85 (0%)*
Alevel	C	50	85	**200**	40	10	0	*385 (1%)*
grades	B	60	185	430	**1085**	335	30	*2130 (3%)*
	A	65	300	1650	8785	**19500**	3935	*34235 (49%)*
	A*	5	20	115	1420	15270	**16635**	*33455 (48%)*
	Total	*205*	*640*	*2405*	*11340*	*35115*	*20600*	*70305*
		(0%)	*(1%)*	*(3%)*	*(16%)*	*(50%)*	*(29%)*	

Under-	Prediction:			Correlations:		
estimate	Accurate	Over		Pearson	Spearman	Polychoric
6.2%	**53.3%**	40.5%		0.552	0.523	0.678

Appendix table 2. Thresholds, and intervals between thresholds, for the grades for applicants at State and Private schools. Values in bold show mean values across rows and down columns.

1	**Ordinal Thresholds**		E:D		D:C		C:B		B:A		A:A*		Mean	State minus Private
2	Predicted	State	-3.11		-2.55		-1.89		-1.18		0.39		**-1.67**	0.47
3		Private	-3.51		-3.00		-2.47		-1.78		0.06		**-2.14**	
4	Attained	State	-2.31		-1.74		-1.13		-0.33		0.83		**-0.94**	0.42
5		Private	-2.57		-2.25		-1.68		-0.81		0.55		**-1.35**	
6	Predicted-Attained	State	-0.80		-0.81		-0.76		-0.85		-0.44		**-0.73**	0.02
7		Private	-0.76		-0.75		-0.79		-0.97		-0.48		**-0.75**	
8			**-2.18**		**-1.85**		**-1.45**		**-0.99**		**0.15**		**-1.26**	
9	**Threshold intervals**			D:C - E:D		C:B - D:C		B:A - C:B		A:A* - B:A				
10	Predicted	State		-0.57		-0.66		-0.71		-1.57			**-0.87**	0.02
11		Private		-0.51		-0.54		-0.69		-1.84			**-0.89**	
12	Attained	State		-0.57		-0.61		-0.80		-1.17			**-0.79**	-0.01
13		Private		-0.32		-0.58		-0.87		-1.36			**-0.78**	
14	Predicted-Attained	State		0.01		-0.05		0.09		-0.41			**-0.09**	-0.02
15		Private		-0.01		0.04		0.18		-0.48			**-0.07**	
16				**-0.33**		**-0.40**		**-0.47**		**-1.14**			**-0.58**	

Appendix figure 1. Demonstration of how a conventional Pearson or Spearman correlation between binary variables P and Q cannot achieve a correlation of one when marginal proportions of P and Q differ. However the tetrachoric correlation is one, within calculation and rounding errors, being estimated from underlying latent correlation shown in the diagram, with thresholds at -0.842 and 0 for P and Q.

	Q absent	Q present	P totals
P absent	**0**	**20**	20
P present	**50**	**30**	80
Qtotals	50	50	100
Correlatio	Pearson	Spearman	Tetrachoric
	0.5	0.5	0.994
Threshold	P	Q	
	-0.842	0	

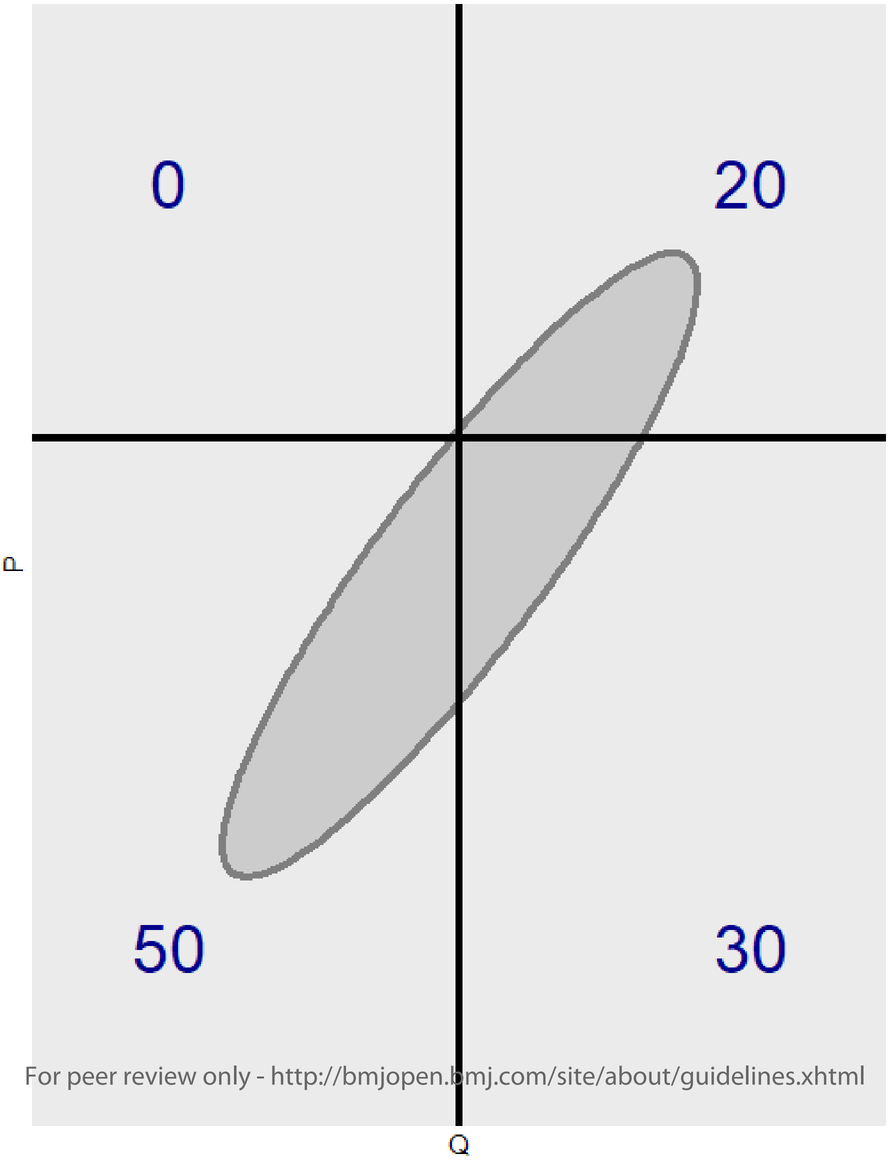

Appendix figure 2. Latent bivariate normal distribution for the relationship between attained A-level grades (horizontal) and predicted A-level grades (vertical). The correlation is represented by the blue ellipse. The dashed blue and yellow line is the line of equality of actual and attained grades. The vertical and horizontal black lines show the thresholds for the grades, shown as E, D, C, B, A and A*. The solid red dots and red line show where the thresholds for a grade intersect, with all below the main diagonal. Colours indicate over-prediction (yellow and pink) and under-prediction (green and blue).

2.a

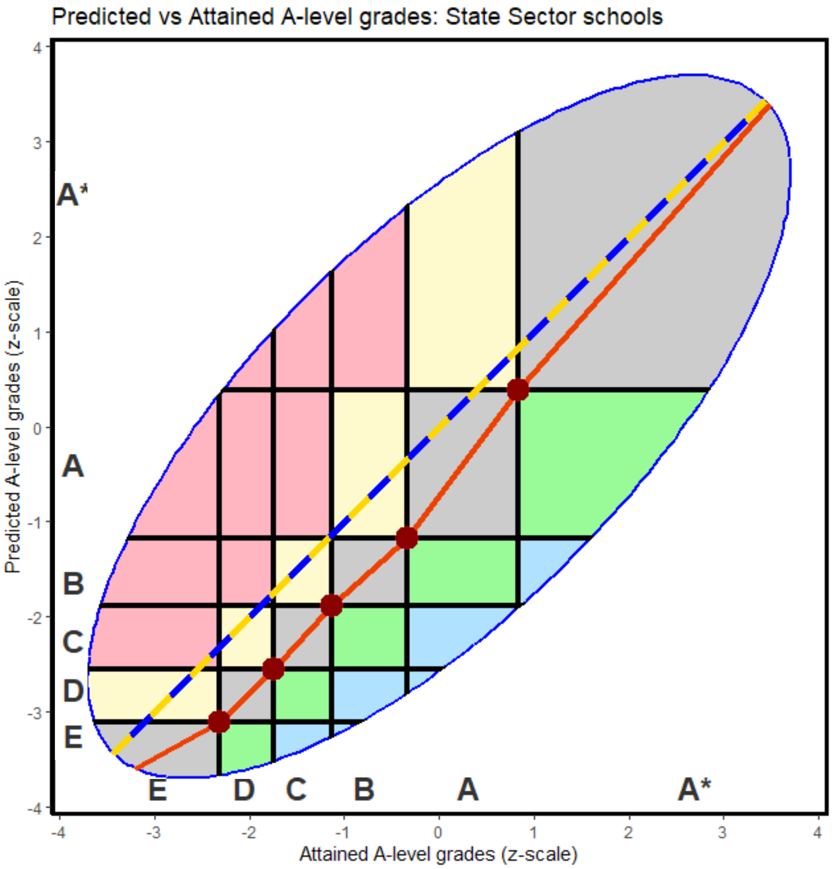

2.b

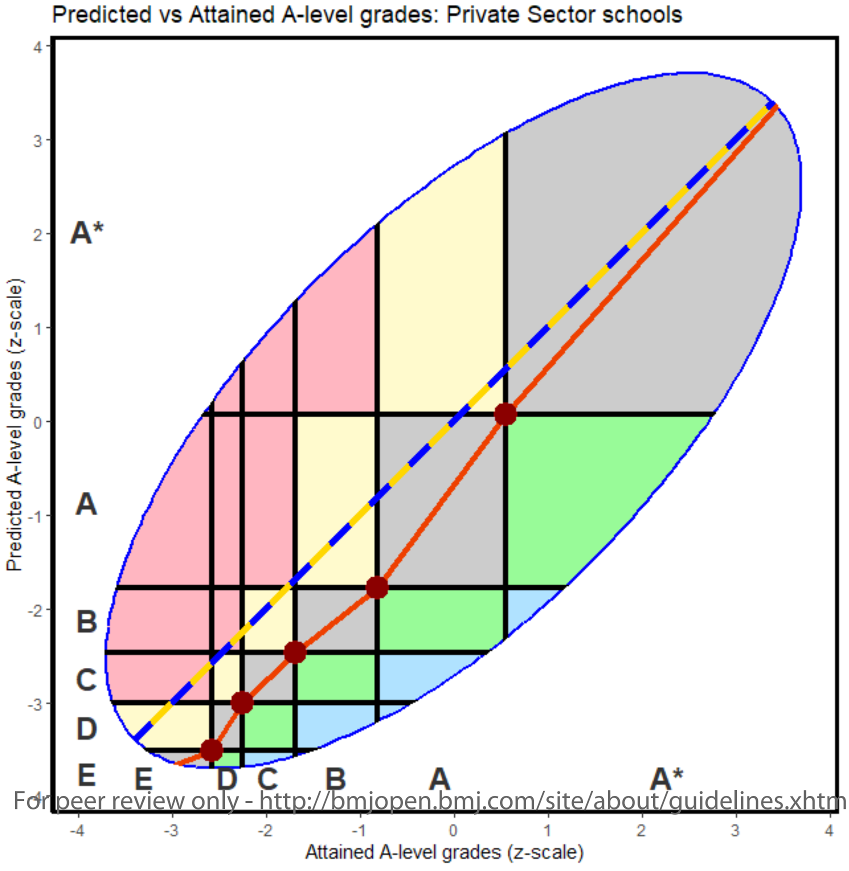

Appendix figure 3. Summary of polychoric thresholds shown on the horizontal axis, for private (blue) and state (red) schools, for attained grades (squares) and predicted grades (circles). Narrower diagonal lines show the links between attained and predicted grades for private (blue) and state (red) schools. Purple diagonal lines link equivalent points for private and state schools (e.g. attained with attained grades and predicted with predicted grades).

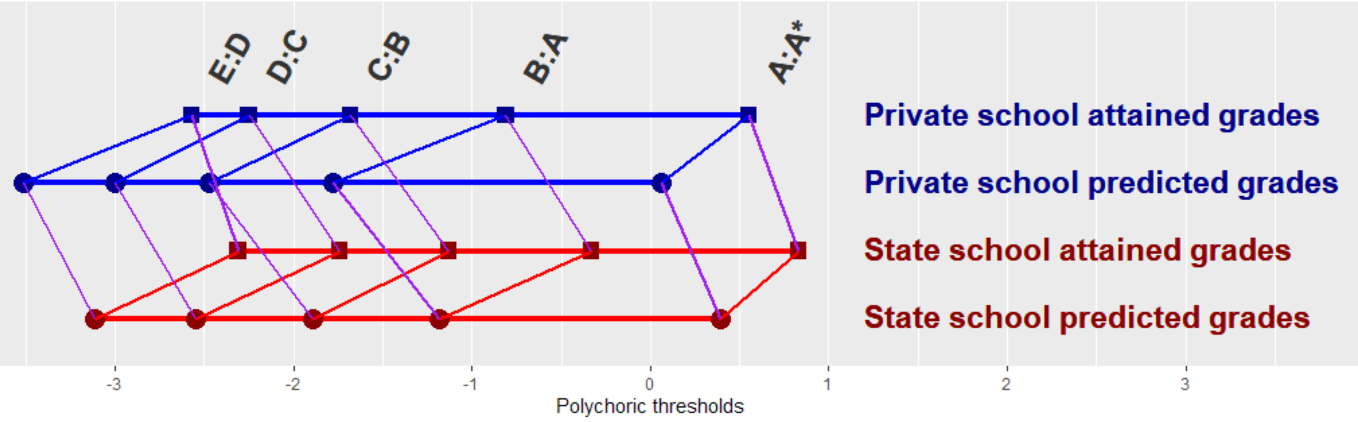

Appendix figure 4. See Appendix figure 2 for the majority of conventions. The fitted ellipses for state sector schools (red) and private sector schools (blue) are shown separately, with the same grade thresholds for both schools. The latent bivariate normal distributions for the two types of school differ entirely in their mean scores, that for private sector schools being shifted up and to the right (by the same amount). The school types therefore differ only in their mean ability levels.

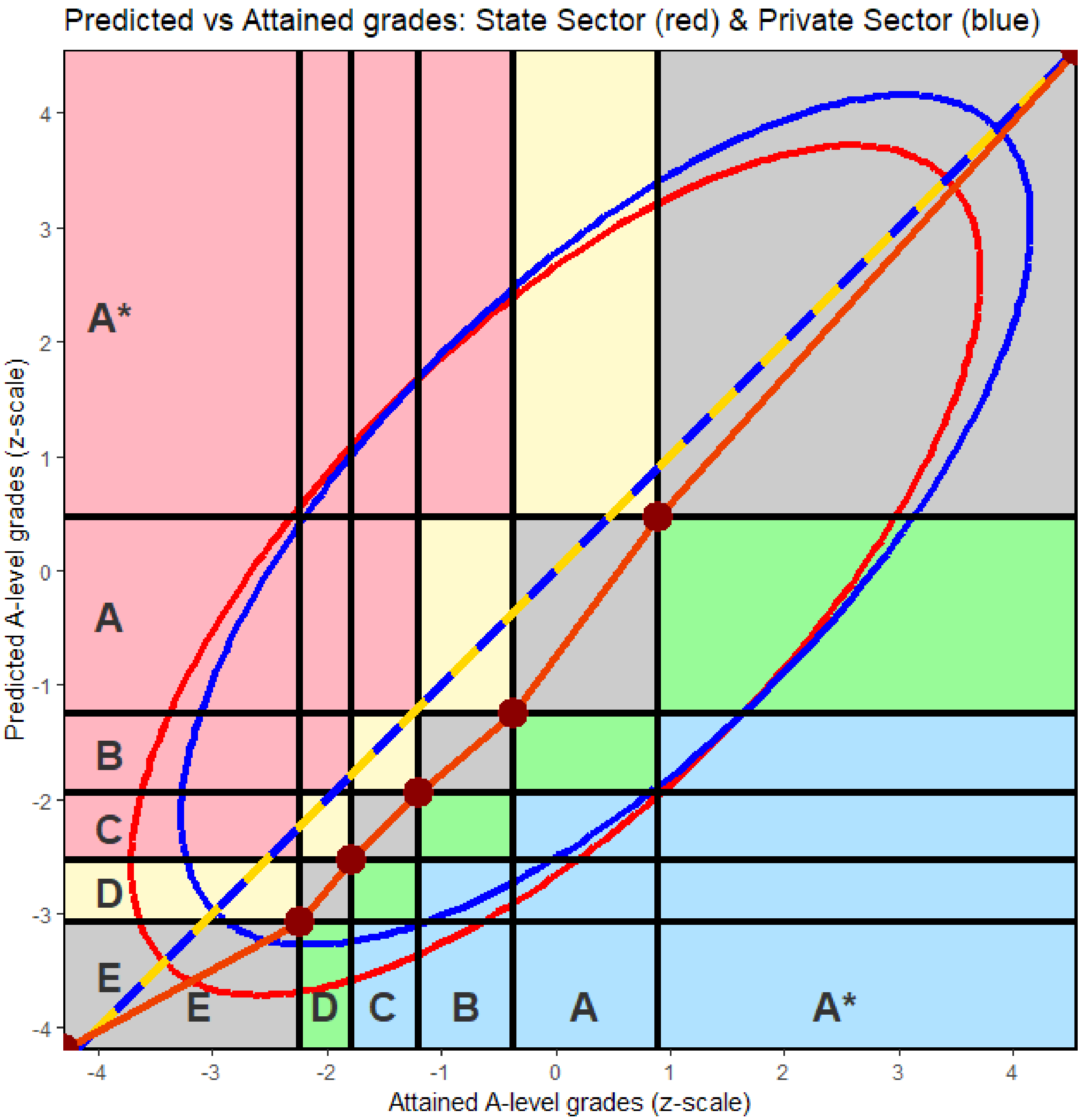

BMJ Open

The predictive validity of A-level grades and teacher-predicted grades in UK medical school applicants: A retrospective analysis of administrative data in a time of COVID-19

Journal:	*BMJ Open*
Manuscript ID	bmjopen-2020-047354.R1
Article Type:	Original research
Date Submitted by the Author:	06-Jul-2021

Complete List of Authors:	McManus, Ian; UCL Medical School, Research Department of Medical Education<br>Woolf, Katherine; UCL Medical School, Research Department for Medical Education<br>Harrison, David; UCL Medical School, Research Department for Medical Education<br>Tiffin, Paul; University of York, Department of Health Sciences; Hull York Medical School, Health Professions Education Unit<br>Paton, Lewis; University of York,<br>Cheung, Kevin; Cambridge Assessment<br>Smith, Daniel; General Medical Council

**Primary Subject Heading**:	Medical education and training
Secondary Subject Heading:	Health services research
Keywords:	COVID-19, MEDICAL EDUCATION & TRAINING, EDUCATION & TRAINING (see Medical Education & Training)

SCHOLARONE™
Manuscripts

I, the Submitting Author has the right to grant and does grant on behalf of all authors of the Work (as defined in the below author licence), an exclusive licence and/or a non-exclusive licence for contributions from authors who are: i) UK Crown employees; ii) where BMJ has agreed a CC-BY licence shall apply, and/or iii) in accordance with the terms applicable for US Federal Government officers or employees acting as part of their official duties; on a worldwide, perpetual, irrevocable, royalty-free basis to BMJ Publishing Group Ltd ("BMJ") its licensees and where the relevant Journal is co-owned by BMJ to the co-owners of the Journal, to publish the Work in this journal and any other BMJ products and to exploit all rights, as set out in our licence.

The Submitting Author accepts and understands that any supply made under these terms is made by BMJ to the Submitting Author unless you are acting as an employee on behalf of your employer or a postgraduate student of an affiliated institution which is paying any applicable article publishing charge ("APC") for Open Access articles. Where the Submitting Author wishes to make the Work available on an Open Access basis (and intends to pay the relevant APC), the terms of reuse of such Open Access shall be governed by a Creative Commons licence – details of these licences and which Creative Commons licence will apply to this Work are set out in our licence referred to above.

Other than as permitted in any relevant BMJ Author's Self Archiving Policies, I confirm this Work has not been accepted for publication elsewhere, is not being considered for publication elsewhere and does not duplicate material already published. I confirm all authors consent to publication of this Work and authorise the granting of this licence.

For peer review only

The predictive validity of A-level grades and teacher-predicted grades in UK medical school applicants: A retrospective analysis of administrative data in a time of COVID-19

I C McManus [a,b,e,*]
i.mcmanus@ucl.ac.uk ORCID 0000-0003-3510-4814
Katherine Woolf [a,c,e]
k.woolf@ucl.ac.uk ORCID 0000-0003-4915-0715
David Harrison [a,e]
david.harrison@ucl.ac.uk ORCID 0000-0002-6639-6752
Paul A Tiffin [b,d,f]
paul.tiffin@york.ac.uk ORCID 0000-0003-1770-5034
Lewis W Paton [b,f]
lewis.paton@york.ac.uk ORCID 0000-0002-3328-5634
Kevin Yet Fong Cheung [b,g]
Cheung.K@cambridgeenglish.org ORCID 0000-0002-9548-2932
Daniel T. Smith [a,b,h]
daniel.smith@gmc-uk.org ORCID 0000-0003-1215-5811

For peer review only

[a] UKMEDP089 project
[b] UKMEDP051 project
[c] UKMEDP089 Principal Investigator
[d] UKMEDP051 Principal Investigator
[e] Research Dept of Medical Education, University College London, London WC1E 6BT, UK
[f] Department of Health Sciences, University of York, Heslington, York, YO10 5DD, UK
[g] Cambridge Assessment, Shaftesbury Road, Cambridge CB2 8EA, UK
[h] General Medical Council, Regent's Place, 350 Euston Road, London NW1 3JN, UK
* Author for correspondence: i.mcmanus@ucl.ac.uk

For peer review only - http://bmjopen.bmj.com/site/about/guidelines.xhtml

Abstract

Objectives

To compare in UK medical students the predictive validity of attained A-level grades and teacher-predicted A-levels for undergraduate and postgraduate outcomes. Teacher-predicted A-level grades are a plausible proxy for the teacher-estimated grades that replaced UK examinations in 2020 as a result of the COVID-19 pandemic. The study also models the likely future consequences for UK medical schools of replacing public A-level examination grades with teacher-predicted grades.

Design

Longitudinal observational study using UK Medical Education Database (UKMED) data.

Setting

UK medical education and training.

Participants

Dataset 1: 81,202 medical school applicants in 2010 to 2018 with predicted and attained A-level grades. Dataset 2: 22,150 18-year old medical school applicants in 2010 to 2014 with predicted and attained A-level grades, of whom 12,600 had medical school assessment outcomes and 1,340 had postgraduate outcomes available.

Outcome measures

Undergraduate and postgraduate medical examination results in relation to attained and teacher-predicted A-level results.

Results

Dataset 1: Teacher-predicted grades were accurate for 48.8% of A-levels, over-predicted in 44.7% of cases and under-predicted in 6.5% of cases. Dataset 2: Undergraduate and postgraduate outcomes correlated significantly better with attained than with teacher-predicted A-level grades. Modelling suggests that using teacher-estimated grades instead of attained grades will mean that 2020 entrants are more likely to under-attain compared with previous years, 13% more gaining the equivalent of the lowest performance decile and 16% fewer reaching the equivalent of the current top decile, with knock-on effects for postgraduate training.

Conclusions

The replacement of attained A-level examination grades with teacher-estimated grades as a result of the COVID-19 pandemic may result in 2020 medical school entrants having somewhat lower academic performance compared to previous years. Medical schools may need to consider additional teaching for entrants who are struggling, or who might need extra support for missed aspects of A-level teaching.

For peer review only - http://bmjopen.bmj.com/site/about/guidelines.xhtml

For peer review only

For peer review only - http://bmjopen.bmj.com/site/about/guidelines.xhtml

Strengths and limitations of this study

- This is the first comparison of the predictive validity of teacher-predicted and attained A-level grades for performance in undergraduate and postgraduate assessments five to eight years later.
- The large sample size of all UK medical applicants from 2010 to 2018 provides adequate statistical power, and the complete population data means the results are unlikely to be biased.
- The teacher-predicted grades are those provided by schools as a part of university application, and probably form a good proxy for the "centre-assessment grades", introduced by Ofqual during the Covid crisis of 2020.
- This study is with medical school applicants only, so that generalisability to students on other university courses is uncertain; however the over-prediction of grades we find in medical school applicants is similar to that found elsewhere for university applicants in general.

For peer review only

For peer review only - http://bmjopen.bmj.com/site/about/guidelines.xhtml

Background

"… the … exam hall [is] a level playing field for all abilities, races and genders to get the grades they truly worked hard for and in true anonymity (as the examiners marking don't know you). [… Now we] are being given grades based on mere predictions." Yasmin Hussein, letter to *The Guardian*, March 29th 2020 [1].

"[Let's] be honest, this year group will always be different…" Dave Thomson, blogpost on *FFT Educational Lab* [2]

"One headmistress commented that 'entrance to university on teachers' estimates may be fraught with unimagined difficulties'. … If there is in the future considerable emphasis on school assessment, some work of calibration is imperatively called for." James Petch, December 1964[3].

UK schools closed on 20th March 2020 in response to the COVID-19 pandemic, and Key Stage 5 [Level 3] public examinations such as A-levels and SQA assessments were cancelled for summer 2020, and replaced by a complex system involving teacher assessments of the grades students would have achieved had they taken the examinations. A-levels and SQA assessments, like other national examinations in the UK, are normally set and marked anonymously by examination boards which are entirely separate from schools, and teachers usually play no part in this external assessment process. A-levels are good predictors of performance at university in general[4], and at medical schools specifically[5 6]. Within this context the present paper compares achieved A-level grades with teacher-predicted grades, and in particular considers their relative predictive validities for educational outcomes at UK medical schools. The analyses were originally described in May 2020 and published as a preprint [7] while events were still ongoing and outcomes were not known. The present paper maintains much of that structure, and while mostly looking forward from 2020, also in part looks back from the perspective of 2021, meaning that past, present and future tenses are intermingled.

On April 3rd 2020 *Ofqual* (Office of Qualifications and Examinations Regulation) in England announced that A-level, GCSE and other exams under its purview, would be replaced by *Calculated Grades,* at the core of which are teachers' estimates of the grades that their students would attain (called *Centre Assessment Grades, CAGs*), which would then be moderated by *Ofqual* using a computer algorithm which included the prior performance of the school attended by candidates – see the Calculated Grades subsection below for details. The Scottish Qualification Authority (SQA) and other national bodies also announced similar processes for their examinations. Inevitably the announcement of Calculated Grades resulted in confusion and uncertainty in examination candidates, particularly those needing A-levels or SQA Advanced Highers[a] to meet conditional offers for admission to university in autumn 2020. Universities also faced a major problem for student selection, having had A-levels taken away, which are, "the single most important bit of information [used in selection]" [8].

Some of the tensions implicit in Calculated Grades are well seen in the quotation above by Yasmin Hussein, a GCSE student in Birmingham, with its clear emphasis that a key strength of current

[a] SQA Highers are taken the year before (rather like AS-levels used to be) and therefore they will be available for 2020 applicants. Advanced Highers will not be available and will be estimated.

For peer review only - http://bmjopen.bmj.com/site/about/guidelines.xhtml

examination systems such as GCSEs, A-levels and similar qualifications, is their *anonymity* and *externality* with assessors who know nothing of the students whose work they are marking. In contrast the replacement of actual grades attained in the exam hall with what Hussein describes as 'mere predictions' raises a host of questions, not the least being the possibility of bias when judgements are made by teachers.

Context of the current paper and the situation at the time of writing

Since the appearance of COVID-19 in Europe in early 2020, the situation has been and still is rapidly changing. As mentioned earlier, this paper was originally written in May 2020, but was revised and submitted to the journal, essentially as the preprint but with some additions, in November 2020 when Europe was in the midst of a 'second wave' and England, Wales, Scotland and Northern Ireland, in a second national lockdown. The paper took almost six months to be reviewed, with revisions only being requested in May 2021 with the third UK national lockdown still not ended. To help the reader situate the current paper we explain briefly here what the exams situation was in the UK from April to August 2020, with more details provided in a postscript in Section 1 of the Supplementary Information.

University selection in the UK for admission in October 2020 began in the autumn, with medical school applicants submitting by October 15th to UCAS applications for four medical schools. Selection which may include interviews and other assessments is usually completed by the end of March with students being told of offers or rejections. Offers are usually conditional on A-levels and other qualifications to be taken in May, with results announced in August. In Spring 2020 as UK universities entered the final phases of the annual academic cycle of student selection, the present paper considered the potential problems of using teacher-estimated grades such as the Calculated Grades proposed by Ofqual, rather than attained grades obtained in the usual way via examinations. The pre-print of May 2020 was circulated primarily for information to medical school admissions tutors. By August 2020 some immediate effects on selection were shown when the algorithms used by regulators resulted in many students, particularly those from historically poorly performing schools, having their expected results adjusted downwards. This forced the Scottish Government, followed then by the English and Welsh Governments, to accept either teacher-estimated Centre Assessment Grades (CAGs) without moderation by an algorithm, or the Calculated Grade, whichever was the higher.

As expected in the pre-print, given that teacher-estimated grades were found to be higher than attained A-level grades, the scrapping of the algorithm resulted in a significant increase in grades compared to 2019[b], with an immediate impact on the numbers of students meeting university conditional offers. Longer-term impacts are still to be seen, with some likely to result from the lower predictive validity of teacher-estimated grades, and a likely increase in under-performing students in medical schools and postgraduate training.

Medical school admissions

This paper mainly concentrates on medical school applications. UK medical education has a range of useful educational measures, including admissions tests during selection, and outcomes at the end of undergraduate training, which are linked together through UKMED (United Kingdom Medical Education Database; https://www.ukmed.ac.uk/). UKMED provides a sophisticated platform for assessing predictive validity in multiple entry cohorts in undergraduate and postgraduate training [9]. The current paper should also be read in parallel with a second study from some members of the

[b] https://ffteducationdatalab.org.uk/2020/08/gcse-and-a-level-results-2020-how-grades-have-changed-in-every-subject/

For peer review only - http://bmjopen.bmj.com/site/about/guidelines.xhtml

present team which assesses attitudes and perceptions to calculated grades and other changes in selection of current medical school applicants in the UKMACS (UK Medical Applicants Cohort Study) [10 11].

Fundamental questions about selection in 2020 concerned the likely nature of Calculated Grades, and the extent to which they would predict outcomes to the same extent as currently did *actual or attained grades*. The discussion will involve actual grades, and then four types of teacher-estimated grades: predicted grades (sent to UCAS at application to university), centre assessment grades (CAGs – submitted by schools to *Ofqual* in 2020) calculated grades (CAGs adjusted using an algorithm) and forecasted A-level grades (submitted by teachers to exam boards pre-2015 as a quality check for real exam grades). These related but different assessments are summarised in Box 1 below, together with final grades, which were the grades eventually accepted by UCAS and were the higher of the calculated grade or centre assessed grade. It should be noted that we have tried to use 'teacher-predicted' grades only to refer to the grades included as a part of the normal UCAS process, whereas the term teacher-estimated grades is used in a more generic sense.

Calculated grades

The status of calculated grades was made clear by *Ofqual* in April 2020:

> "The grades awarded to students will have equal status to the grades awarded in other years and should be treated in this way by universities, colleges and employers. On the results slips and certificates, grades will be reported in the same way as in previous years". [12], p.6.

The decisions of *Ofqual* are supported by Ministerial statement, and universities and other bodies have little choice therefore but to abide by them, although that does not mean that other factors may not need to be taken into account in some cases, as often occurs when applicants do not attain the grades in conditional offers.

For peer review only - http://bmjopen.bmj.com/site/about/guidelines.xhtml

None of the above means that calculated grades actually *will be* equivalent to conventional attained grades. Calculated grades will not actually *be* attained grades, they may well behave differently to attained grades, and in measurement terms they actually *are not* attained grades, even though in administrative and even in legal terms, by fiat, they have to be treated as equivalent. From the perspective of educational research, the key issue is the extent to which calculated grades actually will or can behave in an identical way to attained grades.

Box 1: A-level grades: Actual, predicted, centre assessment, calculated, final, forecasted, and teacher-estimated grades

Actual or attained grades. The grades awarded by examination boards/awarding organisations based on written and other assessments which are set and marked externally. Typically sat in *May and June of year 13*, with results announced in *mid-August*.

Predicted grades. Teacher estimates of the likely attained grades of candidates, provided to UCAS in the *first term of year 13*, and by *October 15th* for medical and some other applicants.

Centre assessment grades. Used in the production of Calculated grades (see below). Provided by examination centres (typically schools) between 1st and 12th of June 2020, consisting of teacher-estimated grades and candidate rankings within examination centres.

Calculated grades. The final grades to be provided for candidates by exam boards for Summer 2020 assessments, in the absence of attained grades. Based on centre assessment grades, with final calculated grades involving standardisation/adjustment by exam boards using an algorithm. Calculated grades, "will have equal status to the grades awarded in other years and should be treated in this way by universities, colleges and employers" (Ofqual). These grades were often referred to as the 'algorithm grades' and were abandoned by the UK governments in August 2020

Final grades. The grades used by UCAS in the 2020 admissions cycle – the higher of the teacher estimated grade or the centre assessment grade

Forecasted grades. Prior to 2015, teachers, in *May of Year 13*, provided to exam boards a forecast of the likely grades of candidates along with rankings. Forecasted grades therefore take place later in the academic cycle than predicted grades, close to the time examinations are actually sat.

Teacher-estimated grades. Generic term used in this paper to refer to grades estimated by teachers. Includes predicted grades, centre assessment grades, calculated grades, and forecasted grades.

In April 2020 *Ofqual* issued guidance on how *calculated grades* would be provided for candidates for whom examinations have been cancelled. Essentially, teachers would be required, for individual candidates taking individual subjects within a *candidate assessment centre (*usually a school), to estimate *grades for* candidates, and then to *rank order* candidates within grades, to produce *centre assessment grades*. A statistical standardisation process would then be carried out centrally using a computer algorithm. Ranking is needed because standardisation, "will need more granular information than the grade alone" ([12] p.7}, presumably to break ties at grade boundaries which occur because of standardisation. Standardisation, to produce *calculated grades*, would use an algorithm that took into account the typical distribution of results from that centre for that subject in the three previous years, along with aggregated centre data on SATS and previous exam attainment as in GCSEs[c]. This approach is consistent with *Ofqual's* approach to standard-setting. Following Cresswell[13], *Ofqual* has argued that during times of change in assessments, and perhaps more

[c] It was this standardisation process that Governments reversed in August 2020 after the protests against calculated grades.

generally, there should be a shift away from “comparable performance” (i.e. criterion-referencing), and that there is an “ethical imperative” to use “comparable outcomes” (i.e. norm-referencing) to minimise advantages and disadvantages to the first cohort taking a new assessment, as perhaps also for later cohorts as teachers improve at teaching new assessments [14].

Ofqual said that centre assessment grades, the core of calculated grades, “are not the same as … predicted grades provided to UCAS in support of university applications” [15], (p.7). Predicted grades in particular are provided by schools in October of year 13 and centre assessment grades in May/June of year 13, seven months later, when Ofqual says that teachers should also consider classwork, bookwork, assignments, mock exams and previous examinations such as AS-levels (taken only by a minority of candidates now), but should *not* include GCSE results, or any student work carried out after 20th March. Whether centre assessment grades, or calculated grades – centre assessment grades moderated by the algorithm - will be fundamentally different from predicted grades is ultimately an empirical question, which should be answerable when UCAS data for 2020 are available for medical school applicants in UKMED. In the meantime, and *it is a core and a reasonable assumption*, that centre assessment grades and hence calculated grades will probably correlate highly with earlier predicted grades, except for a small proportion of candidates who have improved dramatically from October 2019 to March 2020. Predicted grades, which have been collected for decades, should therefore act as a reasonable proxy in research terms for centre assessment grades and therefore calculated grades, particularly in the absence of any other information.

The rationale for using A-level grades in selection

Stepping back slightly it is worth revisiting the reasons that A-levels exist and why universities use them in selection. A-levels assess at least three things: subject knowledge, intellectual ability, and study habits such as conscientiousness [16]. Knowledge and understanding of, say, chemistry is probably necessary for the high level study of medical science and medicine, to which it provides an underpinning, and experience suggests that students without such knowledge may have problems. A-levels also provide evidence for a student’s intellectual ability and capability of extended study at a high level. A-levels are regarded as a ‘gold standard’ qualification because of the rigour and objectivity of their setting and marking (see for example Ofqual’s ‘Reliability Programme’[17]). Their measurement is therefore *reliable*, and the presumption is that they are also *valid*, in some of the many senses of that word [18-20], and as a result are *unbiased*. A crucial assumption is of *predictive validity*, that future outcomes at or after university are higher or better in those who have higher or better A-levels, as found both in predicting degree classes in general [4 21 22] and medical school performance in particular [5 23]. There is also an assumption of *incremental validity*, A-levels being better predictors than other measures[6]. At the other extreme, A-levels could be compared conceptually with, say, a mere assertion by a friend or colleague that, “Oh yes, they know lots of chemistry”. That is likely neither to be reliable, valid nor unbiased, and hence is a base metal compared with the gold standard of A-levels. The empirical question therefore is where on the continuum from gold to base metals, lie calculated grades or teacher-predicted grades.

The issue of predictive validity has been little discussed in relation to calculated grades, but in a *TES* (*Times Educational Supplement*) survey of teachers, there were comments that, “predictions and staff assessments would never have the same validity as an exam”, so that, “Predictions, past assessment data and mock data is not sufficient, and will never beat the real thing in terms of accuracy" [24]. The changes in university selection inevitably meant that difficult policy decisions needed to be made by universities and medical schools. Even in the absence of direct, high-quality, evidence, policy-makers still have an obligation to make decisions, and, therefore it is argued, must take theory, related evidence, and so on, into account [25]. This paper provides both a review of other

For peer review only - http://bmjopen.bmj.com/site/about/guidelines.xhtml

evidence, and also results on the related issue of predicted grades, which it will be argued are likely to behave in a way that is similar to calculated grades.

Review of literature on predicted and forecasted grades

Predicted grades in university selection

A notable feature of UK universities is that selection mostly takes place before A-levels or equivalent qualifications have been sat, so offers are largely conditional on later attained grades. As a result, UCAS application forms, since their inception in 1964, have included *predicted grades*, estimates by teachers of the A-level grades a student is likely to achieve. Admissions tutors also use other information in making conditional offers. A majority of applicants in England, applying in year 13 for university entry at age 18 will have taken GCSEs at age 16 in year 11, a few still take AS-levels in year 12, some students submit an EPQ (Extended Project Qualification), and UCAS forms also contain candidate statements and school references. Medical school applicants mostly also take admissions tests such as U(K)CAT or BMAT at the beginning of year 13, and many will take part in interviews or MMIs (multiple mini-interviews)[d].

Predicted grades have always been controversial. A House of Commons Briefing Paper in 2019 noted that the UK was unusual among developed countries in using predicted grades[e], and said that,

> "The use of predicted grades for university admissions has been questioned for a long time. Many critics argue that predicted grades should not be used for university entry because they are not sufficiently accurate and it has been suggested that disadvantaged students in particular lose out under this system." [26] p.4

Others have suggested that as well as being "biased", "predicting A-level grades is clearly an imprecise science" [27] (p.418). There have been repeated suggestions over the years, none as yet successful, that predicted grades should be replaced with a PQA (Post-Qualification Applications) system. As Nick Hillman puts it,

> "The oddity of our system is not so much that people apply before receiving their results; the oddity is that huge weight is put on predicted grades, which are notoriously unreliable. … PQA could tackle this…"[f].

The system of predicted grades is indeed odd, but also odd is the sparsity of academic research into predicted grades. The most important question that seems almost never to have been asked, and certainly not answered, is the fundamental one of whether it is predicted grades or actual grades which are better at predicting outcomes. Petch[3], in his 1964 monograph which was one of the first serious discussions of the issues, considers that predicted and actual grades may be fundamentally different, perhaps being "complementary and not contradictory" (p.29), one being about scholarly attitude and the other about examination prowess, primarily because "the school knows the candidate as a pupil, knowledge not available to the examiners". For Petch, either a zero correlation or a perfect correlation between predicted and actual grades would be problematic, the latter perhaps implying that actual grades might be seen as redundant (p.6).

The advent of *Ofqual's* calculated grades, which are in effect predicted grades carried out by teachers in a slightly different way, means there was a serious need in 2020 to know how effective

---

[d] see https://www.medschools.ac.uk/studying-medicine/making-an-application/entry-requirements) .
[e] https://www.bbc.co.uk/news/education-44525719
[f] https://www.hepi.ac.uk/2019/08/14/pqa-just-what-does-it-mean/

For peer review only - http://bmjopen.bmj.com/site/about/guidelines.xhtml

predicted grades were likely to be as a substitute for attained A-level grades, and the same concern will apply in 2021, with *Ofqual* implementing a different model for teacher-estimated grades[g]. Are teacher-predicted grades in fact 'notoriously unreliable', being 'mere predictions', or do they have equivalent predictive validity as attained grades?

The research literature on predicted grades

As part of Section 1 of the Supplementary Information to this paper we have included a more detailed overview of research studies on predicted grades. Here we will merely provide a brief set of comments.

Most studies look at predictions at the level of individual exam subjects, which at A-level are graded from E to A or, from 2010 onwards, from E to A*. The most informative data show all combinations of predicted grades against attained grades, and Figure 1 gives an example for medical school applicants. Many commentators, though, look only at over-predictions ('optimistic') and under-predictions ('pessimistic'). Figure 2 summarises data from five studies of university applicants. Accurate predictions occur in 52% of cases when A is the maximum grade and 17% when A* is the maximum grade (and with more categories accuracy is likely to be lower). Grades are mostly over-predicted, in 42% of cases pre-2010 and 73% post-2010, with under-prediction rarer at 7% of cases pre-2010 and 10% post-2010. A number of studies have reported that under-prediction is more common in lower socio-economic groups, non-White applicants, and applicants from state school or further education [28-30]. A statistical issue means such differences are not easy to interpret, as a student predicted A* cannot be under-estimated, and therefore under-estimation will inevitably be more frequent in groups with lower overall levels of attainment. This issue is discussed and analysed at length in Section 5 of the Supplementary Information in relation to applicants from private-sector schools.

Some studies also consider grade-point predictions, the sum of grade scores for the three best attaining subjects, scored A*=12, A=10, B=8, etc[h].. In particular a large study by UCAS [31] showed that applicants 'missing their predictions' (i.e. they were over-predicted) tended to have lower predicted grades, lower GCSE attainment, were more likely to have taken physics, chemistry, biology and psychology, and were from disadvantaged areas. To some extent the same statistical problems of interpretation apply as with analysis at the level of individual exam subjects. For a number of years UCAS only provided grade-point predictions, and they are included in the P51 data analysed below.

What are predicted grades and how are they made?

UCAS says that "A predicted grade is the grade of qualification an applicant's school or college believes they're likely to achieve in positive circumstances."[i] Later though, the document says predicted grades should be, "**in the best interests of applicants** – fulfilment and success at college or university is the end goal ", and "**aspirational but achievable** – stretching predicted grades are motivational for students, unattainable predicted grades are not" (all emphases in original). Predicted grades should be professional judgements and be data-driven, including the use of, "past Level 2 and Level 3 performance, and/or internal examinations to inform …predictions".

---

[g] https://www.gov.uk/government/publications/awarding-qualifications-in-summer-2021/awarding-qualifications-in-summer-2021

[h] In some studies a scoring of A*=6, A=5, B=4 is used. The 12,10,8... scoring was introduced so that AS levels, weighted at half an A-level, could be scored as A=5, B=4 etc (there being no A* grade at AS-level). For most purposes A*=12, A=10 … is equivalent in all respects to A*=6, A=5, etc, apart from a scaling factor.

[i] https://www.ucas.com/advisers/managing-applications/predicted-grades-what-you-need-know [Accessed 13th April 2020].

For peer review only - http://bmjopen.bmj.com/site/about/guidelines.xhtml

Few empirical studies have asked how teachers estimate grades, with not much progress since 1964 when Petch said, "Little seems to be known about measures taken by schools to standardize evaluations of pupils" [3] (p.7). Two important exceptions are the studies of Child and Wilson[j] in 2015 and Gill [32] in May 2018, with only the latter published. Gill sent questionnaires to selected OCR (Oxford, Cambridge and Royal Society of Arts) Examination Board exam centres concerning Chemistry, English Literature and Psychology exams. Teachers said the most important information used in predicting grades was performance in mock exams, observations of quality of work and commitment, oral presentation, the opinion of other teachers in the same subject and in other subjects, and the head of department. Some teachers raised concerns about the lack of high stakes for mock exams which meant that some students did not treat them seriously. AS-level grades were an important aid in making predictions, and there were concerns about the loss of AS-levels to help in prediction, as also mentioned elsewhere [33], and that is relevant to 2020 where most candidates will not have taken AS-levels.

Studies considered so far almost entirely are concerned with teacher predictions of A-level grades, since they are important for university admissions. More generally, studies looking at a wider range of teacher estimates, often in younger children, find a tendency for over-estimation across a range of skills[34], with judgements often being systematically lower for marginalised learners[35]. A different position is taken in a genetically-informed study of twins, which suggests, in a forcefully worded conclusion, that, "Teachers can reliably and validly monitor students' progress, abilities and inclinations. … For these reasons, we suggest that teacher assessments could replace some, or all, high-stakes exams"[36]. The study however uses only correlations as measures of accuracy, and cannot assess over- or under-estimation. Also, teacher ratings were only available at ages 7,11 and 14, at the same time as standardised tests are carried out, but were not available for GCSEs at age 16, or for A-levels and University Entrance at age 18, and as such are not informative for the purposes of the present study.

Predicted grades in other Key Stage 5 qualifications than A-levels

Almost all studies on predicted grades have considered A-levels, with a few occasional exceptions looking at GCSEs. We know of no studies on the Extended Project Qualification (EPQ) in England, of Scottish Highers and Advanced Highers, or any other qualifications. Section 3 of the Supplementary Information includes data on both EPQ and SQA examinations.

Forecasted grades

Until 2015, teachers in the May of school year 13 provided awarding organisations with *forecasted grades*, and those forecasts in part contributed to quality control of grades by the boards. Since forecasted grades were produced five to seven months after predicted grades, and closer to the exam date, they might be expected to be more accurate than predicted grades, being based on better and more recent information. Forecasted grades are important as they are more similar than predicted grades to the proposed calculated grades in the way they are calculated, and it is noted that "they may differ somewhat from the predicted grades sent to UCAS as part of the university application process" [37]. Three formal analyses are available, for candidates in 2009 [38], 2012 [39] and 2014 [37], and four other studies from 1940 [40], 1963 [3], 1977 [41] and 2018 [32] are also available, with one post-2000 study before A* grades were introduced and three after (Figure 2). Petch [40] also provides a very early description of forecasted grades, looking at teachers' predictions of pass or fail in School Certificate examinations in 1940, which also show clear over-prediction.

[j] Child, S., & Wilson, F. (2015). An investigation of A level teachers' methods when estimating student grades. Cambridge Assessment internal report. Cambridge, UK: Cambridge Assessment

For peer review only - http://bmjopen.bmj.com/site/about/guidelines.xhtml

Forecasted A-level grades are similar in accuracy to predicted grades pre-2010 (42% vs 52%) but are more accurate post-2010 (47% vs 17%), in part due to a drop in accuracy of predicted grades when A* grades are available. Despite there being *no aspirational or motivational reasons for teachers to over-predict forecasted grades*, particularly in the 1977 and 2018 studies, over-prediction nevertheless remains as frequent as with predicted grades (pre-2010: 39%; post-2010: 37%) and remains more common than under-prediction (pre-2010: 20%; post-2010 16%). Overall it is perhaps possible that calculated grades may be somewhat more accurate than predicted grades, but forecasted grades appear broadly in their behaviour to predicted grades. Two sets of forecasted grades are available for GCSEs [42 43], and they show similar proportions of over and under-prediction as do results for A-levels. Over-prediction seems to be a feature of all predictions by teachers.

The three non-official studies of forecasted grades also asked teachers to rank-order candidates, a procedure which was included in calculated grades. The 1963 data[3] found a median correlation of rankings and exam marks within schools of 0.78, the 1977 data[41] a correlation of 0.66 [41], and the recent 2018 data [32] a correlation of about .82. The three estimates, mean r = 0.75, are somewhat higher than a meta-analytic estimate of .63 (SE =.03) for teachers' ability to predict academic achievement [44].

The Gill study [32] is also of interest as one teacher commented on the difficulty of providing rankings with 260 students sitting one exam, and the author noted that, "it was easier for smaller centres to make predictions because they know individual students better" (p.42), with it also being the case that responses to the questionnaire were more likely to come from smaller centres. The 1963 study of Petch[3] , as well as commenting on "considerable divergencies … in the methods by which estimates were produced" (p.27), as in the variable emphasis put on mock exams, also adds that, "some of the comments from schools suggested that at times there may be a moral ingredient lurking about some of the estimates"(p.28).

Overall it seems possible but unlikely that calculated grades might be more accurate than predicted grades, but they also make clear the problems shown by teachers in ranking and grading candidates. It also remains possible that examining boards have far more extensive and unpublished data on forecasted grades that they intend to use in assessing the likely effectiveness of calculated grades.

Applicants to medical school

So far, this review section has been entirely about university applicants across all subjects and the entire range of A-level grades. Only a handful of studies have looked at predicted grades in medical school applicants.

Lumb and Vail emphasised the importance of teacher-predicted grades since they determine in large part how shortlisting takes place [45]. In a study of 1995 applicants they found 52% of predictions were accurate, 41% were over-estimated and 7% under-estimated [45], values very similar to those reported in university selection in general (Figure 2).

A study by one of the present team used path modelling to assess the causal inter-relationships of GCSE grades, predicted grades, receipt of an offer, attained A-level grades, and acceptance at medical school [46]. Predicted grades were related to GCSE grades (beta=0.89), and attained A-level grades were predicted by both GCSE grades (beta=0.44) and predicted A-level grades (beta=0.74). The study supports claims that teachers may well be using GCSE grades in part to provide predicted grades, which is perhaps not unreasonable given the clear correlation.

Richardson et al [47] in an important and seemingly unique study looked at the relative predictive validity of predicted as compared with attained A-level grades. Using a composite outcome of pre-clinical performance there was a minimal correlation with predicted grades (r=.024) compared with

For peer review only - http://bmjopen.bmj.com/site/about/guidelines.xhtml

a correlation of 0.318 (p<.001) with attained A-level grades. To our knowledge this is the only study of any sort assessing the predictive validity of predicted vs attained A-level grades.

The present study

Although calculated grades are novel and untested in their details, predicted grades have been around for half a century, and there is also a small literature on forecasted grades. This paper will try to answer several empirical questions about predicted grades, for which data are now available in UKMED. Predicted grades will then be used, *faute de mieux*, to make inferences about the likely consequence of using calculated grades.

Empirical questions to be addressed.

The relationship between predicted and attained grades in medical school applicants

Few previous studies have looked in detail at this high-performing group of students. We will also provide brief results on Scottish Highers and Advanced Highers, and the EPQ (Extended Project Qualification), neither of which has been discussed elsewhere to our knowledge.

The predictive validity of predicted grades in comparison with attained grades

A fundamental question concerning calculated grades is whether teacher-predicted grades are better or worse at predicting outcomes than are actual A-level grades. The relationship between predicted grades and actual grades cannot itself answer that question. Instead what matters is the relative performance of predicted and actual grades in predicting subsequent outcomes at the end of undergraduate or postgraduate training. The only relatively small study on this of which we are aware in medical students [47] found that only actual grades had predictive validity.

Method

The method provided here is brief. A fuller description including a detailed table of measures can be found in Section 2 of the Supplementary Information. Overall the project is **UKMEDP112**, approved by the UKMED Research Group in May 2020, with data coming from two separate but related UKMED projects, both of which included predicted grades.

Project **UKMEDP089**, "The UK Medical Applicant Cohort Study: Applications and Outcomes Study", approved Dec 7th, 2018, with Dr Katherine Woolf as principal investigator, is an ongoing analysis of medical student selection as a part of UKMACS (UK Medical Applicant Cohort Study[k]). The data upload of 21st Jan 2020 included detailed information from UCAS and HESA on applicants for medicine from 2007 to 2018.

Project **UKMEDP051**, "A comparison of the properties of BMAT, GAMSAT and UKCAT", approved Sept 25th, 2017, with Dr Paul Tiffin as principal investigator, is an ongoing analysis of the predictive validity of admissions tests and other selection methods such as A-levels and GCSEs in relation to undergraduate and postgraduate attainment. The present analysis used the download files dated 13th May 2019[l]. UCAS data are included, although when the present analysis began the file had not yet included the detailed subject level information available in UKMEDP089[m]. Outcome data for the P51 dataset are extensive, and in particular undergraduate progression data are included, such as

[k] https://ukmacs.wordpress.com/
[l] UKCAT51_APP_ALL_DATA_13052019_FILE1.SAV and UKCAT51_APP_ALL_DATA_13052019_FILE2.SAV .
[m] An upload for P51 was made available on 20th April 2020 but was not included in the present analyses.

For peer review only - http://bmjopen.bmj.com/site/about/guidelines.xhtml

UKFPO EPM and SJT, and PSA (Prescribing Safety Assessment), as well as performance on some postgraduate examinations (MRCP Part1 and MRCS Part A).

Data from HESA and hence UKMED are required to be reported using their rounding and suppression criteria (https://www.hesa.ac.uk/about/regulation/data-protection/rounding-and-suppression-anonymise-statistics) and those criteria have been used for all UKMED data. In particular the presence of a zero or the absence of a percentage may not always mean that there are no individuals in a cell of a table, and all integers are rounded to the nearest 5.

Results

A fuller description of the results can be found in Section 3 of the Supplementary Information.

The relationships between predicted and actual grades in medical school applicants.

Predicted and actual A-level grades for individual A-level examinations

Figure 1 shows the relationship between predicted and attained A-level grades for 237,030 examinations from 2010 to 2018 (i.e. assessments including A* outcomes). 39.3% of predicted grades are A* compared with 23.7% of attained grades. Figure 1.a shows predicted grades in relation to attained grades, with bold font for accurate predictions, green and blue shading for under-prediction and orange and red shading for over-prediction. Overall 48.8% of predicted grades are accurate, which is higher than for university applications in general (see Figure 2), reflecting the high proportion of A and A* grades (69%). Over-prediction occurred in 44.7% of cases, and under-prediction in 6.5% of cases. Figure 1.b show the data as percentages. About a half of A* predictions result in an attained A grade, and over a third of predicted A grades result in grade B or lower. Predicted and attained grades have a Pearson correlation of r = 0.63.

Differences between A-level subjects

There is little in the literature on the extent to which different A-level subjects may differ in the accuracy of their predictions, perhaps with different degrees of bias or correlation. Detailed results are presented in Section 3 of the Supplementary Information. Overall, Biology, Chemistry, Maths and Physics are very similar in terms of over-prediction and correlation with actual grades. However General Studies is particularly over-estimated compared with other subjects.

Extended Project Qualification (EPQ) and SQA Advanced Highers

Section 3 of the Supplementary Information contains information on these qualifications. SQA Advanced Highers, as well as the EPQ, show similar proportions of over-estimation as other qualifications (see Figure 2).

Reliability of predicted and attained A-level grades

Considering the best three A-level grades, the reliability of an overall score can be calculated from the correlations of the individual subjects. For 66,006 candidates with at least three paired predicted and actual grades, Cronbach's alpha was 0.827 for actual grades and 0.786 for predicted grades, with a highly significant difference. The difference may in part reflect the higher proportion of A* grades in predicted than actual grades, and hence a greater ceiling effect, but may also reflect greater measurement precision in the marking of actual A-levels.

For peer review only - http://bmjopen.bmj.com/site/about/guidelines.xhtml

How reliable are attained A-level grades?

Attained A-level grades, like any behavioural measurement are not perfectly reliable, in the sense that if a candidate took a parallel test containing equivalent but different items it is highly unlikely that they would get exactly the same mark as on the first attempt. They may, for instance, have been lucky (or unlucky) at their first attempt, being asked questions on topics which they happened to have studied or revised more (or revised less), and so on. Reliability is a technical subject[n] with many different approaches [48 49]. For continuous measures of raw scores, the reliability can be expressed as a coefficient such as alpha (and in one A-level maths test in 2011, alpha for the full test was about 0.97 [50], although it is suggested that value is unusually high). Boards though do not report raw scores, but instead award grades on a scale such as A* to E. The 'classification accuracy' of grades is harder to estimate, and is greater with fewer grade points, wider grade intervals, and a wide spread of candidate ability [50]. There seem to be few published estimates of classification accuracy for A-levels (although they do exist for GCSEs and AS-levels [50]).

Estimating classification accuracy for the present high-attaining group of medical school applicants is not easy. A fundamental limit for any applicants is that predicted grades cannot possibly predict actual grades better than attained grades predict themselves (the reliability or classification accuracy). However, from considering the correlation of the three best predicted and actual grades it is unlikely that such a limit has currently been reached. The correlation of actual with predicted grades is .585, and the alpha reliabilities of .827 for actual grades and .786 for predicted grades (see above). The disattenuated correlation between predicted and actual grades is therefore .585/(√(.827x.786) = 0.726, which is substantially less than one, with predicted grades accounting for only about a half of the true variance present in actual grades. If the disattenuated correlation were close to one then it could be argued that predicted grades were doing as well as they could possibly do given that attained grades are not perfectly reliable, but that is clearly far from the case.

True scores and actual scores

From a theoretical, psychometric, point of view it could be argued that it is neither actual nor predicted grades which need to be estimated for applicants, but their 'true ability scores', or the 'latent scores', to use the technical expressions, of which predicted and actual grades are but imperfect estimates. In an ideal world that would be the case, and a well-constructed exam tries to get as close as possible to true scores. However, it is not possible to know true scores (and if it were the boards would provide selectors with those scores). Selection itself does not work on true scores but on the actual grades that are written down, by teachers for predicted grades, and as grades on exam result certificates by boards. They are the currency in which transactions are conducted during selection, so that a predicted grade of less than a certain level means a candidate will not get a conditional offer, and likewise too low an actual grade means a candidate holding a conditional offer will be rejected. For that reason it is not strictly the correlation of predicted and actual grades which matters, the two measures being treated as symmetric, but the forward prediction of actual grades from predicted grades, i.e. the actual grades conditional on the predicted grades (as shown in figure 1b).

[n] See https://www.gov.uk/government/publications/reliability-of-assessment-compendium for a range of important papers commissioned and published by Ofqual.

For peer review only - http://bmjopen.bmj.com/site/about/guidelines.xhtml

Predictive validity of predicted and attained A-level grades in medical students.

Predictive validity in UKMEDP051

The version of the P51 data used here consists entirely of applicants applying to medical schools, but there is also follow-up into undergraduate and postgraduate training. Predicted A-level grades were available only for the UCAS application cycles of 2010 to 2014 (i.e. applying for university entry in October 2009, for the academic year 2010/11, etc), and consisted of a single score in the range 4 to 36 points, based on the sum of the three highest predicted grades, scored as A*=12, A=10, etc. The modal score for 38,965 applicants was 30 (equivalent to AAA; mean=31.17; SD= 3.58; Median = 32; 5th, 25th, 75th and 95th percentiles= 26, 30, 34 and 36). For simplicity the study was restricted to applicants aged 18 in the year of application, who had both predicted and attained A-levels, which also ensured the sample contained only first applications for non-graduate courses, from candidates who had not taken pre-2010 A-levels, when A* grades were not available. Overall, 22,955 applicants were studied. Other selection measures included were GCSEs (mean grade for best eight grades), as well as U(K)CAT and BMAT scores, based on the most recent attempt which for cases was also the first attempt. For simplicity we used the total of the four sub-scores of U(K)CAT, and the total of Section 1 and 2 scores for BMAT.

Follow-up is complicated as application cohorts enter medical school in different years, and spread out in time through medical school and training. Figure 3 uses an Ibry chart [51-54] to show the educational progression of typical 18-year old medical school entrants, through to postgraduate qualifications. There are however many variants on this theme. The horizontal axis shows academic years (September to August) and training years (August to July), with career stages, key events and measures used on the vertical axis, with coloured boxes indicating typical students, although there are many variants on entry and progression. The blue boxes show typical students on a five-year course who entered medical school in October 2010 at the age of 18. They would have taken GCSEs in June 2008 in school year 11, in the 2007/8 academic year, and some would have taken AS-levels in June 2009. Applicants would have taken aptitude tests in school year 13, most taking either U(K)CAT or BMAT but some taking both tests. U(K)CAT would have been taken between July and September 2009, and BMAT in November 2009. UCAS applications are submitted in October, with teachers providing teacher-estimated grades. Note that U(K)CAT results are known before UCAS applications, but BMAT results are not known until after application. A-levels would have been taken in May-June 2010, with results known in August 2010, and successful applicants entering medical school in Oct 2010. Students on a five year course would start the 2nd medical school year in Oct 2011, the 3rd and 4th years in 2012 and 2013, and during their final year beginning in Oct 2014 they would take the SJT and PSA tests and be awarded an EPM score, with graduation in May 2015. The first of the two Foundation years starts in August 2015, and core or specialist training begins in August 2017. Medical students at some schools take an optional or a compulsory intercalated BSc (iBSc) between years 2 and 3. As a result they are then a year later in progressing to the later stages, and are shown by the green boxes in figure 3. Although years are broadly divided into Basic Medical Science and Clinical stages, some medical schools have courses which are far more integrated[55].

The above description is for 18-year olds entering the 2010 entry cohort. The present study included the 2010 to 2014 entry cohorts (shown by the solid black box in the lower left of figure 3). For simplicity the last of those cohorts is the only other one, the 2014 entrants having red boxes to show progression for a five year course, and orange for a six-year course including an iBSc. It should be re-emphasised that all career trajectories are idealized, and in reality students and doctors have many and varied training trajectories.

For peer review only - http://bmjopen.bmj.com/site/about/guidelines.xhtml

Data were available up until the 2018 academic year, and years after that are therefore shown greyed out in figure 3. Although all cohorts had data for EPM, SJT and PSA, the later entry cohorts are less likely to have postgraduate qualifications.

Undergraduate outcome measures were for simplicity restricted to the deciles of the UKFPO's Educational Performance Measure (EPM), the raw score of the UKFPO's Situational Judgement Test (SJT), and the score relative to the pass mark of the Prescribing Safety Assessment (PSA), all at first attempt. Relatively few doctors, mostly from the earlier cohorts, had progressed through to postgraduate assessments, but sufficient numbers for analysis were present for MRCP(UK) Part 1 and MRCS Part A, scores being analysed at the first attempt. It should be noted that while U(K)CAT, BMAT, PSA, SJT, and postgraduate assessments are *nationally standardised*, EPM deciles are *locally standardised* within medical schools.

EPM, is a complicated measure summarising academic progression through the first four years of medical school, with individual medical schools deciding what measures to include [56], and expressed as deciles *within* each school and graduating cohort year. EPM is here used as the main undergraduate outcome measure. EPM deciles are confusing, as UKFPO scores them in the reverse of the conventional order, the first decile being highest performance and the tenth the lowest[o]. Here for ease of interpretation we reverse the scoring in what we call *revDecile*, so that higher scores indicate higher performance. It should also be remembered that deciles are not an equal interval scale (figure 4)

Correlations between the measures are summarised in Figure 5. Large differences in Ns reflect some measures being used in applicants during *selection*, and others being outcome measures that are only present in *entrants*, as well as the smaller numbers of doctors who had progressed to postgraduate assessments. The distinction is emphasised by dividing the correlation matrix into three separate parts. Correlations of selection and outcome measures necessarily show *range restriction* because candidates have been selected on the basis of the selection measures, and likewise doctors taking postgraduate examinations may be self-selected for earlier examination performance.

Figure 5 contains much of interest (see also Section 3 of the Supplementary Information), but the most important question for present purposes is the extent to which Predicted and Attained A-level grades (shown in pink and green in Figure 5) differ in their prediction of the five outcome measures, remembering that undergraduate outcomes are typically five or six years after selection, and postgraduate outcomes are seven or eight years after selection.

Attained A-levels predict EPM with a simple Pearson correlation of r=0.297 compared with a correlation of only 0.198 for predicted grades (simple correlations, *r,* are shown in blue in figure 5). N is large for these correlations and hence the difference, using a test for correlated correlations [57] is highly significant (Z=12.6, $p<10^{-33}$). Multiple regression (see Section 3 of the Supplementary Information) suggests that predicted grades may have a small amount of predictive variance which is not shared with attained A-levels. Figure 4 shows mean EPM *revDecile* scores in relation to actual and predicted A-levels. The slope of the line is clearly less for predicted A-levels, showing a less good prediction. It is also clear that attained grades predict well, A*A*A* entrants scoring an average of two deciles higher at the end of the course than those with AAA grades, each extra grade raising average performance by about two-thirds of a decile. In contrast the slope is less for predicted grades, being slightly less than half a decile per predicted A-level grade. The broad pattern of results

[o] https://foundationprogramme.nhs.uk/wp-content/uploads/sites/2/2019/11/UKFP-2020-EPM-Framework-Final-1.pdf,

For peer review only - http://bmjopen.bmj.com/site/about/guidelines.xhtml

is similar for the other undergraduate outcomes, SJT and PSA, and is shown in section 3 of the Supplementary Information.

The two postgraduate outcome measures, MRCP(UK) Part 1 (Membership of the Royal Colleges of Physicians (UK) examination) Part 1, and MRCS (Membership of the Royal College of Surgeons) Part A , although both based on smaller, but still substantial, numbers of doctors, are still significant, actual grades correlating more highly with MRCP(UK) Part 1 (r=.421) than do predicted grades (r=.283; Z= 4.54, p=.000055). Likewise, actual grades correlate more highly with MRCS Part A (r=.421) than do predicted grades (r=.358; Z= 3.67, p=.000238).

The simple correlations (*r)* in figure 5 are inevitably range restricted as A-level grades and predicted A-level grades have themselves been used as a part of the selection process. Taking range restriction into account using the method of Hunter, Schmidt and Le[6 58] (see also [59]), uses u_X, the ratio of standard deviations in the predictors in the unrestricted and the restricted population, values below one indicating more range restriction. Figure 5 shows u_X [uX] at the bottom of the columns, and it can be seen that it is much lower for actual A-level grades than predicted A-level grades, suggesting that actual grades are more important in the selection process than are predicted grades. Construct-level predictive validity (CLPV)[6] can be calculated, taking reliability of measures into account, using .827 for attained A-levels and .785 for predicted A-levels (see earlier), with all other reliabilities set at 0.9 in the absence of better estimates. Note that the calculation, unlike that carried out previously [6], for simplicity does not take censorship/ceiling effects of A-levels into account, and a fuller analysis will be presented elsewhere. The CLPV, ρ_{TPa} [shown as rTPa in figure 5], given the greater range restriction, are relatively higher for actual Alevel grades than for predicted Alevel grades. CLPV for predicting EPM is 0.403 for actual A-level grades compared with 0.251 for predicted A-level grades. For predicting postgraduate qualifications, CLPV for MRCP(UK) Part 1 and MRCS Part A are .601 and .519 for attained A-level grades compared with .360 and .216 respectively for predicted A-level grades.

There are suggestions that predicted grades may not be equivalent in candidates from state schools and private schools, grades being predicted more accurately in independent schools [28 29]. That is looked at in Section 5 of the Supplementary Information, and while there is clear evidence, as found before in the UKCAT-12 study [60], that private school entrants underperform relative to expectations based on their A-levels, there is no evidence that predicted grades behave differently in candidates from private schools.

A practical question relevant to calculated grades concerns the extent to which, in the absence of attained A-level grades, other selection measures such as GCSEs, U(K)CAT and BMAT can replace the predictive variance of attained A-level grades. That will be considered for EPM where the sample sizes are large. Attained grades alone give r = 0.297, and predicted grades alone give r=.198, accounting for less than half as much outcome variance. Adding GCSEs to a regression model including just predicted grades increases multiple R to .225, and also including U(K)CAT and BMAT increases it to .231, which though is still substantially less than the .297 for attained A-levels alone. In the absence of attained A-level grades, prediction is improved by including GCSEs and U(K)CAT or BMAT, but the prediction still falls short of that for actual A-levels alone.

Modelling the effect of only predicted grades being available for selection

In the context of the 2020 pandemic, an important question is the extent to which future outcomes may change as a result of selection being in terms of calculated grades. Calculated grades themselves were not known at the time of the study, but predicted grades are probably a reasonable surrogate for them in the first instance. A modelling exercise was therefore carried out whereby the numbers of students in the various EPM *RevDeciles* were tabulated in relation to predicted grades at five grade levels, 36 pts ≡ A*A*A*, 34 pts ≡ A*A*A, 32 pts ≡ A*AA, 30 pts ≡ AAA

For peer review only - http://bmjopen.bmj.com/site/about/guidelines.xhtml

and ≤ 28 pts ≡ ≤ AAB, with the probability of each decile found for each predicted A-level band. Assuming that selection results in the usual numbers of entrants with grades of A*A*A*, A*A*A, etc, but based on calculated grades rather than actual grades, the expected numbers of students in the various EPM deciles can be found. Figure 6 shows deciles as standard UKFPO deciles (1 = highest), UKFPO scores (43 = highest), and *RevDeciles*, (10 = highest). The blue column shows the actual proportions in the deciles based on attained A-level grades. Note that for various reasons there are not exactly equal proportions in the ten deciles[p]. Based on selection on attained A-level grades there are 7.2% of students in the lowest performing decile, compared with an expected proportion of 8.1% for selection on predicted grades, an increase of 0.9% percentage points, which is a relative increase of 13.0% in the proportion of the lowest decile, with an odds ratio of 1.141 of attaining the lowest decile. For the highest scoring decile, the proportion decreases from 10.1% with actual A-level grades to 8.8% if predicted A-level grades are used, an absolute decrease of 1.4%, and a relative decrease of 13.4% of top deciles, with an odds ratio of 0.853.

Of course, the above calculations are based on the assumption that the 'deciles' for calculated grades are expressed at the same standard as currently. Were the outcomes to be re-standardised so that all deciles were equally represented then of course at finals no noticeable difference in performance would be present[q]. However the academic backbone would still be present, and overall poorer performance on statistically equated postgraduate exams[61] would be expected.

Discussion

The present data make clear that under a half of predicted grades are accurate, with 45% being higher than attained grades, and 17% being lower. The data also show that attained grades are far better predictors of medical school performance than are predicted grades, which account for only about a third as much outcome variance as attained grades. Attained grades are also more reliable than predicted grades.

Validation is the bottom line for all measures used during selection, and in the present case it is validation against assessment five to eight years down the line from the original A-levels, in both undergraduate and postgraduate assessments. That is strong support for what we have called 'the academic backbone', prior attainment providing the underpinning for later attainment, and hence there are correlations in performance at all stages of training from GCSEs through to medical degrees and on into postgraduate assessments [5].

Our findings contradict suggestions that holistic judgments by teachers of predicted grades are better predictors of outcomes since teachers may know their students better than examiners. The immense efforts by exam boards and large numbers of trained markers to refine educational measurements is therefore gratifying and reassuring. Careful measurement does matter.

An important question is whether there is some variance in predicted and actual grades which is complementary. We found that adding predicted grades to the model predicting outcomes improved the multiple correlation coefficient by only 0.05, accounting for only an additional 0.25% of variance)). This suggests that predicted grades may provide a very small amount of additional information in predicting outcomes. What that information might be is unclear, and it is possible

---

[p] In part this reflects the fact that some students, particularly weak ones, are given an EPM score, but then fail finals.

[q] This is based on deciles being calculated in a way that are equated to levels used in the present calculation. Of course if calculated strictly as deciles then of necessity 10% will still remain in the top decile, etc.. That difficulty of deciles will in large part be removed when the 2020 entrants graduate as UKMLA should be on stream by then.

For peer review only - http://bmjopen.bmj.com/site/about/guidelines.xhtml

that it is what Petch called 'scholarly attitude'. At present though it is worth remembering that *examination* grades at A-level are primarily predicting further *examination* grades at the end of medical school, although EPM scores do include formal assessments of course work, and practical and clinical skills. If other outcome measures, perhaps to do with communication, caring or other non-cognitive skills were available then predicted grades might show a greater predictive value.

The present data inevitably have some limitations. There is little likelihood of bias since complete population samples have been considered, and there is good statistical power with large sample sizes. Inevitably not all outcomes can be considered, mainly because the cohorts analysed have not yet progressed sufficiently through postgraduate training. However, those postgraduate outcomes which are included do show substantial effects which are highly significant statistically.

Our questions about predicted grades have been asked in the practical context of the cancellation of A-level assessments and their replacement by calculated grades, as a result of the COVID-19 pandemic. It seems reasonable to assume, given the literature on predicted grades, and particularly on forecasted grades, that calculated grades will probably have similar predictive ability to predicted grades, but perhaps be a little more effective due to occurring later in the academic cycle. Such a conclusion would be on firmer ground if exam boards had analysed the predictive validity of the data they had collected on forecasted grades, particularly in comparison with predicted and actual grades. Such data may exist, and if so then they need to be seen. In their absence, the present data may be the best available guesstimates of the likely predictive validity of calculated rather than actual grades.

A potential limitation of our study is that we do not include the calculated and final grades for students who applied for admission in 2020;however calculated and final grades for 2020 will be available in UKMED in 2021, and since that year group will also have the teacher-predicted grades submitted to UCAS, an immediate question of interest will be the extent of the correlation of the measures, and hence whether teacher-predicted grades are indeed a proxy for calculated grades. Having said that, it will not be possible to calculate the predictive validity of teacher-predicted and calculated grades for a number of years until the cohort progresses through undergraduate training. Medium- and long-term predictive validity inevitably take time to acquire, and practical decision-making sometimes has to be based on proxy, surrogate measures, with teacher-predicted grades at application to UCAS being a reasonable substitute. If it were the case that teacher-predicted grades for UCAS and teacher-estimated grades as a part of calculated grades were fundamentally discrepant then serious questions would be raised about one or other set of estimates. The same applies to the teacher-estimated grades being used as a substitute for A-levels in the summer of 2021, which will apply to the cohort applying for entry to medical school in 2021.

Under-prediction

Under-prediction is a particular risk in cases where teachers do not know their students well, or in some cases perhaps underestimate their ability because of attitude, personal characteristics, or other factors. There is some evidence that teacher-assessed grades relate more to student personality than do grades in national examinations[62 63], although effects were relatively weak. Any such biases are traditionally solved by the externality and objectivity of national examinations. Petch, once again, put it well, describing,

> "instances, where, in the examination room, candidates have convinced the examiners that they are capable of more than their schools said that they were … Paradoxical as it will seem, examiners are not always on the side of authority; an able rebel can find his wider scope within the so-called cramping confines of an examination." [3](p.29).

For peer review only - http://bmjopen.bmj.com/site/about/guidelines.xhtml

There is a clear echo here of the quote by Yasmin Hussein with which this paper began. Hussein's concerns are not alone, and the UKMACS study in April 2020 found concerns about fairness were particularly present in medical school applicants from non-selective schools, from Black Asian and Minority Ethnic (BAME) applicants, from female applicants, and from those living in more deprived areas [10].

Effects of loss of schooling.

A further consideration is more general and asks what the broader effects of the COVID-19 pandemic may be on medical education. Students at all levels of education have had teaching and learning disrupted, often extensively, and that is also true of all stages of medical education. The 2020 cohort of applicants/entrants will not have been assessed formally at A-level. As well as meaning that they may only have calculated grades, which are likely to be less accurate, they also will have missed out on significant amounts of teaching. UK students who should have taken A-level exams in 2020 missed around 30 to 40 school days; those in the year below from whom 2021 medical school entrants will be drawn, will have missed around 80 days. Burgess and Sievertsen [64], using data from two studies [65 66], estimate that 60 lost school days results in a reduction in performance of about 6% of a standard deviation, which they say is, "non-trivial" (and for comparison a rule of thumb is that students in school improve by about one third of a standard deviation in each school year[67]). These effects are likely to differ also by socio-economic background, particularly given variability in the effectiveness of home schooling. Applicants not taking A-levels will also suffer from the loss of the enhanced learning that occurs when learners are tested – the 'testing effect' – for which meta-analyses have found effect sizes of about 0.50 [68 69], which is also non-trivial. Taken overall, 2020 entrants to medical school, and perhaps those in 2021 as well, may – without additional support - perform less well in the future as a result of missing out both on education and on its proper assessment.

Conclusions

The events of 2020 as a result of the COVID-19 pandemic were extra-ordinary, and unprecedented situations occurred of which the cancellations of GCSE and A-level exam cancellations were but one example. The current study should not be seen as criticism of the response of *Ofqual* to that situation; given the circumstances in which it found itself, with examinations cancelled (when the Chair of Ofqual, Roger Taylor, had recommended socially-distanced or delayed exams), Ofqual's solution to the problems had many obvious virtues. We began this paper by quoting a letter to a newspaper in March 2020 at the beginning of lockdown by a student taking GCSEs, and so it is probably appropriate to finish with a letter to a different newspaper by an A-level student. Written at the height of the A-levels crisis, in August 2020, it raises many subtle, important and mostly neglected questions, ones which researchers will need to grapple with in the future:

> "*Ofqual's* grading system appears to be lacking in advocates. Blinded by rhetoric about what protesters call a 'classist' algorithm, key facts have been overlooked. It is very clear that teachers are shockingly bad at predicting grades; using teacher predictions there will be a 12% inflation in higher grades compared with last year. While some centres predicted accurately, some centres predicted only the highest grades for their students. This U-turn from the government entails a huge injustice for the pupils who had fair and accurate predictions, as well as for those taking exams next year. In the zero-sum game of university applications, the results of these pupils make them appear weaker than they are. Irresponsible teachers who over-predicted their pupils' results ought to be ashamed that they too have thereby 'dashed the dreams' of many young people across the country. That it

For peer review only - http://bmjopen.bmj.com/site/about/guidelines.xhtml

is less obvious does not make it any less true." (Letter to *The Times*, 19th August 2020, by Seb Bird, A-level student, Bristol)[70].

For most university applicants there already existed predicted grades from the previous autumn when UCAS applications were submitted, but they would have been on average half a grade or so too high, being aspirational as much as realistic, and also for medical students would have been made by October 2019, whereas calculated grades would be based on teacher predictions in May 2020, albeit with several months of courses missing since March 2020.

In May 2020 we wrote that raw teacher-predicted grades would have wrecked much university planning, particularly coming so late in the year, after offers had been made, as numbers of acceptances would inevitably have been far too high[7]. That in fact happened, and quotas for university entries had to be abandoned in August 2020, including for medicine, and that had knock-on effects into first year university courses, and probably beyond. There was also a risk that predicted grades could have been systematically higher from some schools than others – the ones with a tendency to call all of their "geese" "swans" -- and that probably applies also to the centre assessment grades sent to examination boards and mostly eventually accepted without central standardisation in August 2020. The consequences of that will not become apparent for a few years.

This paper has provided evidence that the grades awarded to medical applicants in summer 2020 will probably not predict future outcomes with the same effectiveness as actual, attained grades, and that is a problem that universities and medical schools and postgraduate deaneries will have to work with, probably for many years as the 2020 cohort works through the system. It seems likely therefore, as Thomson has said, "… this year group will always be different…"[2].

For peer review only

For peer review only - http://bmjopen.bmj.com/site/about/guidelines.xhtml

Figure captions

Figure 1: Predicted vs attained A-level grades for individual subjects in applicants to UK medical schools. Accurate predictions are in bold; yellow – over-estimates by 1 grade; orange – over-estimates by 2+ grades; green – under-estimates by 1 grade; blue – under-estimates by 2+ grades. a) Counts; b) attained grades as percentages within predicted grades.

Figure 2: Over-estimated, under-estimated and accurate predicted grades in various studies. Black font: predicted grades; red font: forecasted grades; yellow background: pre-2000; blue background: pre-2010; bold, underlined: averaged results post-2000.

Figure 3: An Ibry chart illustrating the progression of the 2010 to 2014 medical school entry cohorts through secondary schooling, application to medical school, undergraduate and post-graduate training, with the timing of key events shown. See text for further details.

Figure 4: Mean EPM *revDeciles* (95% CI) in relation to actual A-level grades (green) and predicted A-level grades (red)

Figure 5: Correlation matrix of selection measures, undergraduate outcome measures, and postgraduate outcome measures (separated by grey lines for clarity). Cells indicate simple Pearson correlations (*r*; in blue), construct-level predictive validity (*rTPa*; in red) and sample size (*N*; in black).

Figure 6: Predicted decile outcomes if selection were on Predicted A-level grades (blue) rather than actual A-level grades (orange).

For peer review only

For peer review only - http://bmjopen.bmj.com/site/about/guidelines.xhtml

Acknowledgements

We are grateful to Paul Garrud, Gill Wyness, Paul Newton, Colin Melville and Christian Woodward for their comments on earlier versions of this manuscript, and to Jon Dowell, Peter Tang, Rachel Greatrix and other members of the UKMED Research Group and Advisory Board for their assistance in fast-tracking the pre-print of this paper, and for their comments on it. We also thank Tim Gill for providing us with an unpublished manuscript.

Contributors

DTS prepared the data extracts, provided details on date sources and variable definitions where required and commented on manuscript drafts. ICM originated the idea for the study, and discussed it with other authors throughout the project. ICM wrote the first draft of the manuscript, and KW, DH, PAT, LP, KYFC and DTS have read, reviewed and commented on earlier drafts and contributed ideas, as well as approving the final draft, both of the preprint, and of the present paper.

Funding

KW is a National Institute for Health Research (NIHR) Career Development Fellow (NIHR CDF-2017-10-008) and is principal investigator for the UKMACS and UKMEDP089 projects supported by the NIHR funding.

DH is funded by NIHR grant CDF-2017-10-008 to KW.

PAT's research time is supported by an NIHR Career Development Fellowship (CDF 2015-08-11), and PAT is also principal investigator for the UKMEDP051 project.

LWP is partly supported by NIHR grant CDF 2015-08-11 to PAT, and a portion of his research time is funded by the UCAT board.

ICM, KYFC and DTS have received no specific funding for this project.

Disclaimers

KW and DH state that this publication presents independent research funded by the National Institute for Health Research (NIHR). The views expressed are those of the authors and not necessarily those of the NHS, the NIHR or the Department of Health and Social Care.

PAT states this research is supported by an NIHR Career Development Fellowship (CDF 2015-08-11). This paper presents independent research partly funded by the National Institute for Health Research. The views expressed are those of the authors and not necessarily those of the NHS, the NIHR or the Department of Health and Social Care.

KYFC is employed as the Head of Marking and Results at Cambridge Assessment English. The views expressed are those of the authors and do not represent the views of Cambridge Assessment.

DTS is employed by the GMC as a data analyst working on the UKMED project. The views expressed here are his views and not the views of the GMC.

Data sources. UK Medical Education Database (UKMED) UKMEDP051 data extract generated on 13th May 2019. UKMEDP089 extract generated 21st January 2020. UKMEDP112 project, using UKMEDP051 and UKMEDP089 data, approved for publication on 29th May 2020. We are grateful to UKMED for the use of these data. However, UKMED bears no responsibility for their analysis or interpretation.

For peer review only - http://bmjopen.bmj.com/site/about/guidelines.xhtml

UKMEDP051 data includes information derived from that collected by the Higher Education Statistics Agency Limited (HESA) and provided to the GMC (HESA Data). Source: HESA Student Record 2002/2003 to 2014/2015. Copyright Higher Education Statistics Agency Limited. The Higher Education Statistics Agency Limited makes no warranty as to the accuracy of the HESA Data, cannot accept responsibility for any inferences or conclusions derived by third parties from data or other information supplied by it.

UKMEDP051 and UKMEDP089 include Universities and Colleges Admissions Service (UCAS) data provided to the GMC (UCAS data). Source: UCAS (application cycles 2007 to 2018). Copyright Universities and Colleges Admissions Service (UCAS). UCAS makes no warranty as to the accuracy of the UCAS Data and cannot accept responsibility for any inferences or conclusions derived by third parties from data or other information supplied by it.

All data from HESA are required to be reported using their rounding and suppression criteria (https://www.hesa.ac.uk/about/regulation/data-protection/rounding-and-suppression-anonymise-statistics) and we have applied those criteria to all UKMED-based tables and values reported here.

For peer review only

Competing interests

ICM is a member of the UKMED Research Group and the UKMED Advisory Board, and is also on the UKMACS advisory group.

PAT is a member of the UKMED Research Group. PAT has previously received research funding from the ESRC, the EPSRC, the Department of Health for England, the UCAT Board, and the GMC. In addition, PAT has previously performed consultancy work on behalf of his employing University for the UCAT Board and Work Psychology Group and has received travel and subsistence expenses for attendance at the UCAT Research Group.

KYFC is a member of the UKMED Research Group, and is an employee of Cambridge Assessment - a group of exam boards that owns and administers the BioMedical Admissions Test (BMAT); UK GCSEs and A-levels; and International GCSEs and A-levels.

DTS is a member of the UKMED Research Group and the UKMED Advisory Board and is employed by the GMC as a data analyst working on the UKMED project.

KW, DH and LP declare no competing interests.

Authorship

ICM conceived the idea for the study, conducted the statistical analysis and wrote the first draft of the paper. All authors contributed to subsequent drafts and approved the final version.

Ethical approval

Queen Mary Research Ethics Committee, University of London, agreed on 11 November 2015 that there was no need for ethical review of UK Medical Education Database research studies. https://www.ukmed.ac.uk/documents/UKMED_research_projects_ethics_exemption.pdf

All data from HESA are required to be reported using their rounding and suppression criteria (https://www.hesa.ac.uk/about/regulation/data-protection/rounding-and-suppression-anonymise-statistics) and we have applied those criteria to all UKMED-based tables and values reported here.

Provenance and peer review

Not commissioned; reviewed by the UKMED Research Subgroup and Advisory board, and to submitted to an externally peer reviewed journal. This paper was fast-tracked through the UKMED governance processes as with other COVID-19 related research projects elsewhere.

Patient and public involvement.

No patient involvement.

Data sharing statement

Researchers wishing to re-analyse the data used for this study can apply for access to the same datasets via UKMED (www.ukmed.ac.uk).

Figure captions

Figure 1: Predicted vs attained A-level grades for individual subjects in applicants to UK medical schools. Accurate predictions are in bold; yellow – over-estimates by 1 grade; orange – over-estimates by 2+ grades; green – under-estimates by 1 grade; blue – under-estimates by 2+ grades. a) Counts; b) attained grades as percentages within predicted grades.

Figure 2: Over-estimates, under-estimate and accurate predicted grades in various studies. Black font: predicted grades; red font: forecasted grades; yellow background: pre-2000; blue background: pre-2010; bold, underlined: averaged results post-2000.

Figure 3: An Ibry chart illustrating the progression of eighteen-year old 2010 to 2014 medical school entry cohorts through secondary schooling, application to medical school, undergraduate and post-graduate training, with the timing of key events shown. See text for further details.

Figure 4: Correlation matrix of selection measures, undergraduate outcome measures, and postgraduate outcome measures (separated by grey lines for clarity). Cells indicate Pearson correlations (r) and N, as well as the construct-level predictive validity (rTPa). Range restriction is indicated by uX. See text for details.

Figure 5: Predicted decile outcomes if selection were on Predicted A-level grades (blue) rather than actual A-level grades (orange).

For peer review only

References

1. Hussein Y. Cancellation of GCSE is unfair to some students. *The Guardian* 2020(March 29th): https://www.theguardian.com/world/2020/mar/29/cancellation-of-gcse-exams-unfair-to-some-students.
2. Thomson D. Moderating teaching judgments in 2020 [Blog post, 25th March 2020]. London: FFT Educational Lab: https://ffteducationdatalab.org.uk/2020/03/moderating-teacher-judgments-in-2020/ (accessed 16th April 2020) 2020.
3. Petch JA. School estimates and examination results compared. Manchester: Joint Matriculation Board 1964.
4. Higher Education Funding Council for England [HEFCE]. Differences in student outcomes: The effect of student characteristics. Data Analysis March 2018/05,. Bristol: HEFCE 2018.
5. McManus IC, Woolf K, Dacre J, et al. The academic backbone: Longitudinal continuities in educational achievement from secondary school and medical school to MRCP(UK) and the Specialist Register in UK medical students and doctors. *BMC Medicine* 2013;11:242:doi:10.1186/741-7015-11-242.
6. McManus IC, Dewberry C, Nicholson S, et al. Construct-level predictive validity of educational attainment and intellectual aptitude tests in medical student selection: Meta-regression of six UK longitudinal studies. *BMC Medicine* 2013;11:243:doi:10.1186/741-7015-11-243.
7. McManus IC, Woolf K, Harrison D, et al. Calculated grades, predicted grades, forecasted grades and actual A-level grades: Reliability, correlations and predictive validity in medical school applicants, undergraduates, and postgraduates in a time of COVID-19. *medRxiv* 2020;doi: https://doi.org/10.1101/2020.06.02.20116830
8. McKie A. Scrapped exams may spark UK admissions 'scramble'. *Times Higher Education* 2020;26th March 2020:9-9.
9. Dowell J, Cleland J, Fitzpatrick S, et al. The UK medical education database (UKMED): What is it? Why and how might you use it? *BMC Medical Education* 2018;18 (DOI 10.1186/s12909-017-1115-9)(6):1-8.
10. Woolf K, Harrison D, McManus IC. The attitudes, perceptions and experiences of medical school applicants following the closure of schools and cancellation of public examinations due to the COVID-19 pandemic in 2020. *medRxiv* 2020;submitted
11. Woolf K, Harrison D, McManus C. The attitudes, perceptions and experiences of medical school applicants following the closure of schools and cancellation of public examinations in 2020 due to the COVID-19 pandemic: a cross-sectional questionnaire study of UK medical applicants. *BMJ open* 2021;11(3):e044753.
12. Ofqual. Summer 2020 grades for GCSE, AS and A level, Extended Project Qualification and Advanced Extension Award in maths: Guidance for teachers, students, parents and carers. Coventry: Ofqual: Ofqual/20/6607/2 https://assets.publishing.service.gov.uk/government/uploads/system/uploads/attachment_data/file/877842/Summer_2020_grades_for_GCSE_AS_A_level_EPQ_AEA_in_maths_-_guidance_for_teachers_students_parents.pdf (accessed 3rd April 2020) 2020.
13. Cresswell M. Heaps, prototypes and ethics: The consequence of using judgments of student performance to set examination standards in a time of change. London: Institute of Education 2003.
14. Ofqual. Setting GCSE, AS and A Level Grade Standards in Summer 2014 and 2015. London: https://www.gov.uk/government/publications/setting-gcse-and-a-level-grade-standards-in-summer-2014-and-2015 [accessed 18th April 2020] 2020.
15. Ofqual. Summer 2020 grades for GCSE, AS and A level, Extended Project Qualification and Advanced Extension Award in maths: Information for Heads of Centre, Heads of Department and teachers on the submission of Centre assessment grades. Coventry: Ofqual: Ofqual/20/6607/1 https://assets.publishing.service.gov.uk/government/uploads/system/uploads/attachment_data/file/877930/Summer_2020_grades_for_GCSE_AS_A_level_EPQ_AEA_in_maths_-_guidance_for_heads_of_centres.pdf (accessed 3rd April 2020) 2020.

For peer review only - http://bmjopen.bmj.com/site/about/guidelines.xhtml

16. McManus IC, Powis DA, Wakeford R, et al. Intellectual aptitude tests and A levels for selecting UK school leaver entrants for medical school. *BMJ* 2005;331:555-59.
17. Opposs D, He Q. The reliability programme: Final Report. Coventry: Office of Qualifications and Examinations Regulation (http://www.ofqual.gov.uk/files/reliability/11-03-16-Ofqual-The-Final-Report.pdf) 2011.
18. Downing SM. Validity: On the meaningful interpretation of assessment data. *ME* 2003;37:830-37.
19. Association AER, Association AP, Education NCoMi. Standards for educational and psychological testing. Washington, DC: American Educational Research Association 2014.
20. Kane MT. Validating the interpretations and uses of test scores. *Journal of Educational Measurement* 2014;50(1):1-73.
21. Bekhradnia B, Thompson J. Who does best at University? London: Higher Education Funding Council England (http://webarchive.nationalarchives.gov.uk/20081202000732/http://hefce.ac.uk/Learning/whodoes/) 2002.
22. Higher Education Funding Council for England [HEFCE]. Differences in degree outcomes: The effect of subject and student characteristics. Issues Paper 2015/21. Bristol: HEFCE 2015.
23. McManus IC, Smithers E, Partridge P, et al. A levels and intelligence as predictors of medical careers in UK doctors: 20 year prospective study. *BMJ* 2003;327:139-42.
24. Lough C. GCSEs: Only 39% teachers think 2020 grades fair for all: Plan for teacher-assessed GCSE and A-level grades prompts concerns about potential teacher bias, TES survey of 19,000 finds. *TES (Times Educational Supplement)* 2020;13th May 2020:https://www.tes.com/news/coronavirus-gcses-only-39-teachers-think-2020-grades-fair-all.
25. Lilford R. Policy makers should use evidence, but what should they do in an evidence vacuum? *ARC West Midlands News Blog [NIHR Applied Research Collaboration, West Midlands]* 2020;2(4 (April 24th, 2020)):1-2 (https://arcwm.files.wordpress.com/2020/04/arc-wm-newsblog-20-04-24.pdf).
26. Hubbles S, Bolton P. The review of university admissions [Briefing Paper Number 8538, 10 April 2019]. London: House of Commoons [https://researchbriefings.files.parliament.uk/documents/CBP-8538/CBP-8538.pdf] 2019.
27. Snell M, Thorpe A, Hoskins S, et al. Teachers' Perceptions and A-Level Performance: Is There Any Evidence of Systematic Bias? *Oxford Review of Education* 2008;34(4):403-23.
28. Everett N, Papageorgiou J. Investigating the Accuracy of Predicted A Level Grades as part of 2009 UCAS Admission Process. London: Department for Business, Innovation and Skills 2011.
29. Wyness G. Predicted grades: Accuracy and impact. A report of University and College Union. London: University and College Union (https://www.ucu.org.uk/media/8409/Predicted-grades-accuracy-and-impact-Dec-16/pdf/Predicted_grades_report_Dec2016.pdf) 2016.
30. UCAS. End of cycle report 2017: Qualifications and competition. Cheltenham: UCAS [https://www.ucas.com/data-and-analysis/ucas-undergraduate-releases/ucas-undergraduate-analysis-reports/2017-end-cycle-report] 2017.
31. UCAS. Factors associated with predicted and achieved A level attainment, August 2016. Cheltenham: UCAS: https://www.ucas.com/file/71796/download?token=D4uuSzur 2016.
32. Gill T. Methods used by teachers to predict final Alevel grades for their students. *Research Matters (UCLES)* 2019(28):33-42.
33. Walland E, Darlington E. Insights on trends in AS Levels, the EPQ and Core Maths: summary report. Cambridge: 35859 /id}. Cambridge Assessment: https://www.cambridgeassessment.org.uk/.../527125-insights-on-trends-in-as- levels-the-epq-and-core-maths-summary-report.pdf 2019.
34. Urhahne D, Wijnia L. A Review on the Accuracy of Teacher Judgments. *Educational Research Review* 2020:100374.
35. Meissel K, Meyer F, Yao ES, et al. Subjectivity of teacher judgments: Exploring student characteristics that influence teacher judgments of student ability. *Teaching and Teacher Education* 2017;65:48-60.

For peer review only - http://bmjopen.bmj.com/site/about/guidelines.xhtml

36. Rimfeld K, Malanchini M, Hannigan LJ, et al. Teacher assessments during compulsory education are as reliable, stable and heritable as standardized test scores. *Journal of Child Psychology and Psychiatry* 2019;60(12):1278-88.
37. Gill T, Benton T. The accuracy of forecast grades for OCR Alevels in June 2014: Statistics Report Series No 90. Cambridge: Cambridge Assessment [https://www.cambridgeassessment.org.uk/Images/241261-the-accuracy-of-forecast-grades-for-ocr-a-levels-in-june-2014.pdf] 2015.
38. Gill T, Rushton N. The accuracy of forecast grades for OCR Alevels: Statistics Report Series No 26. Cambridge: Cambridge Assessment [https://www.cambridgeassessment.org.uk/our-research/all-published-resources/statistical-reports/150215-the-accuracy-of-forecast%20-grades-for-ocr-a-levels-in-june-2012.pdf/] 2011.
39. Gill T, Chang Y. The accuracy of forecast grades for OCR A levels in June 2012: Statistics Report Series No.64. Cambridge: Cambridge Assessment Statistics Report Series No.64 2013.
40. Petch JA. Fifty years of examining: The Joint Matriculation Board, 1903-19531953.
41. Murphy RJL. Teachers' assessments and GCE results compared. *Educational Research* 1979;22(1):54-59.
42. Gill T, Chang Y. The accuracy of forecast grades for OCR GCSEs in June 2013: Statistics Report Series No 89. Cambridge: Cambridge Assessment [https://www.cambridgeassessment.org.uk/Images/241260-the-accuracy-of-forecast-grades-for-ocr-gcses-in-june-2013.pdf] 2015.
43. Gill T, Benton T. The accuracy of forecast grades for OCR GCSEs in June 2014: Statistics Report Series No 91. Cambridge: Cambridge Assessment [https://www.cambridgeassessment.org.uk/Images/241265-the-accuracy-of-forecast-grades-for-ocr-gcses-in-june-2014.pdf] 2015.
44. Südkamp A, Kaiser J, Möller J. Accuracy of Teachers' Judgments of Students' Academic Achievement: A Meta-Analysis. *Journal of Educational Research* 2012;104(3):743-62.
45. Lumb AB, Vail A. Applicants to medical school: the value of predicted school leaving grades. *Med Educ* 1997;31:307-11.
46. McManus IC, Richards P, Winder BC, et al. Medical school applicants from ethnic minorities: identifying if and when they are disadvantaged. *Brit Med J* 1995;310:496-500.
47. Richardson PH, Winder B, Briggs K, et al. Grade predictions for school-leaving examinations: do they predict anything? *Med Educ* 1998;32:294-97.
48. Wilmut J, Wood R, Murphy R. A review of research into the reliability of examinations: A discussion paper prepared for the School Curriculum and Assessment Authority. Nottingham: School of Education [available at www.gov.uk/systems/uploads] 1996.
49. Bramley T, Dhawan V. Estimates of Reliability of Qualifications. Cambridge: Cambridge Assessment:[https://assets.publishing.service.gov.uk/government/uploads/system/uploads/attachment_data/file/578868/2011-03-16-estimates-of-reliability-of-qualifications.pdf] 2010.
50. Wheadon C, Stockford I. Classification accuracy and consistency in GCSE and A level examinations offered by the Assessment and Qualifications Alliance (AQA) November 2008 to June 2009. Office of Qualifications and Examinations Regulation: Coventry (http://www.ofqual.gov.uk/files/reliability/11-03-16-AQA-Classification-Accuracy-and-Consistency-in-GCSE-and-A-levels.pdf) 2011.
51. Marey EJ. La Méthode graphique dans les sciences expérimentales et particuliérement en physiologie et en médecine. Paris1878.
52. Wainer H, Harik P, Neter J. Visual Revelations: Stigler's Law of Eponymy and Marey's Train Schedule: Did Serjev Do It Before Ibry, and What About Jules Petiet? *Chance* 2013;26(1):53-56.
53. Tufte ER. The visual display of quantitative information. Cheshire, CT: Graphics Press 2018.
54. Garrud P, McManus IC. Impact of accelerated, graduate-entry medicine courses: a comparison of profile, success, and specialty destination between graduate entrants to accelerated or standard medicine courses in UK. *BMC Medical Education* 2018;18: 250:https://doi.org/10.1186/s12909-018-1355-3.

For peer review only - http://bmjopen.bmj.com/site/about/guidelines.xhtml

55. Devine OP, Harborne AC, Horsfall HL, et al. The Analysis of Teaching of Medical Schools (AToMS) survey: an analysis of 47,258 timetabled teaching events in 25 UK medical schools relating to timing, duration, teaching formats, teaching content, and problem-based learning. *BMC Medicine* 2020;18(126):https://doi.org/10.1186/s12916-020-01571-4.
56. Curtis S, Smith D. A comparison of undergraduate outcomes for students from gateway courses and standard entry medicine courses. *BMC Medical Education* 2020;20(4):https://doi.org/10.1186/s12909-019-1918-y.
57. Meng X-L, Rosenthal R, Rubin DB. Comparing correlated correlation coefficients. *Psychological Bulletin* 1992;111(1):172-75.
58. Hunter JE, Schmidt FL, Le H. Implications of direct and indirect range restriction for meta-analysis methods and findings. *Journal of Applied Psychology* 2006;91(3):594-612.
59. Fife DA, Mendoza JL, Terry R. Revisiting Case IV: A reassessment of bias and standard errors of Case IV under range restriction. *British Journal of Mathematical and Statistical Psychology* 2013;66(3):521-42.
60. McManus IC, Dewberry C, Nicholson S, et al. The UKCAT-12 study: Educational attainment, aptitude test performance, demographic and socio-economic contextual factors as predictors of first year outcome in a collaborative study of twelve UK medical schools. *BMC Medicine* 2013;11 :244:doi:10.1186/741-7015-11-244.
61. McManus IC, Chis L, Fox R, et al. Implementing statistical equating for MRCP(UK) Parts 1 and 2. *BMC Medical Education* 2014;14(204):http://www.biomedcentral.com/1472-6920/14/204; doi:10.1186/472-6920-14-204.
62. Papageorgiou KA, Likhanov M, Costantini G, et al. Personality, Behavioral strengths and difficulties and performance of adolescents with high achievements in science, literature, art and sports. *Personality and Individual Differences* 2020;160:109917.
63. Zimmermann F, Schütte K, Taskinen P, et al. Reciprocal effects between adolescent externalizing problems and measures of achievement. *Journal of educational psychology* 2013;105(3):747.
64. Burgess S, Sievertsen HH. Schools, skills, and learning: The impact of COVID-19 on education. https://voxeu.org/article/impact-COVID-19-education (1st April 2020; accessed 31st May 2020)2020.
65. Carlsson M, Dahl GB, Öckert B, et al. The effect of schooling on cognitive skills. *Review of Economics and Statistics* 2015;97(3):533-47.
66. Lavy V. Do differences in schools' instruction time explain international achievement gaps? Evidence from developed and developing countries. *The Economic Journal* 2015;125(588):397-424.
67. Hanushek EA, Woessman L. The economic impacts of learning losses (OECD Education Working Paper). *Education Working Papers (OECD Publishing, Paris)* 2020;No. 225 doi: https://doi.org/10.1787/21908d74-e
68. Rowland CA. The effect of testing versus restudy on retention: A meta-analytic review of the testing effect. *Psychological Bulletin* 2014;140(6):1432-63.
69. Yang C, Luo L, Vadillo MA, et al. Testing (quizzing) boosts classroom learning: A systematic and meta-analytic review. *Psychological Bulletin* 2021
70. Bird S. A-levels fiasco. *The Times* 2020 August 19th.

For peer review only - http://bmjopen.bmj.com/site/about/guidelines.xhtml

a) Counts of number of cases

		Attained Alevel grades						
		E	D	C	B	A	A*	*Total*
Predicted Alevel grades (points)	E (2 pts)	**200**	35	10	5	0	0	*255 (0%)*
	D (4 pts)	235	**610**	155	35	10	0	*1045 (0%)*
	C (6 pts)	635	1220	**2110**	505	95	5	*4570 (2%)*
	B (8 pts)	635	2095	4755	**7355**	1695	175	*16715 (7%)*
	A (10 pts)	430	1925	8785	35640	**61950**	12655	*121390 (51%)*
	A* (12 pts)	50	135	635	6025	42815	**43395**	*93060 (39%)*
	Total	*2185*	*6020*	*16450*	*49570*	*106570*	*56235*	*237030*
		(1%)	*(3%)*	*(7%)*	*(21%)*	*(45%)*	*(24%)*	

b) Percentages within predicted grades

		Attained Alevel grades						
		E	D	C	B	A	A*	*Total*
Predicted Alevel grades (points)	E (2 pts)	**79%**	14%	..	..	..	..	*100%*
	D (4 pts)	23%	**58%**	15%	3%	..	..	*100%*
	C (6 pts)	14%	27%	**46%**	11%	2%	..	*100%*
	B (8 pts)	4%	13%	28%	**44%**	10%	1%	*100%*
	A (10 pts)	0%	2%	7%	29%	**51%**	10%	*100%*
	A* (12 pts)	0%	0%	1%	7%	46%	**47%**	*100%*
	Total	*1%*	*3%*	*7%*	*21%*	*45%*	*24%*	*100%*

For peer review only - http://bmjopen.bmj.com/site/about/guidelines.xhtml

Key:	Blue background: Pre-2010 results	Yellow background: Pre-2000 results				
	Red font: Forecasted grades	**Bold, underlined: Averaged results, post 2000**				
	Study	Context	Year	A-level range	Under-estimated "Pessimistic"	Accurate	Over-estimated "Optimistic"
**University applicants overall: A-levels, etc**							
	Everett & Papageorgiou (2011) [24]	Predicted Grades	2009	A-E	7%	52%	42%
	UCAS [27]	Predicted Grades	2012	A*-E	12%	20%	68%
	Wyness (2016) [25]	Predicted Grades	2013-15	A*-E	9%	16%	75%
	UCAS [27]	Predicted Grades	2016		9%	16%	74%
	UCAS [27]	Predicted Grades	2017		10%	16%	73%
	Petch (1953) [33]	Forecasted Grades	1940	School Cert Pass/Fail	2%	89%	9%
	Petch (1964) [4]	Non-official forecasted Grades	1963	A+B/C+D/E/O/F	18%	43%	39%
	Murphy (1979) [34]	Non-official forecasted Grades	1977	A-E	29%	27%	44%
	Gill and Rushton (2011) [31]	Forecasted Grades	2009	A-E	12%	55%	33%
	Gill and Chang (2013) [32]	Forecasted Grades	2012	A*-E	13%	48%	39%
	Gill and Benton (2015) [30]	Forecasted Grades	2014	A*-E	14%	43%	43%
	Gill (2019) [28]	Non-official forecasted Grades	2018	A*-E	20%	45%	35%
		**Mean Predicted Grades**	Pre-2010	A-E	**7%**	**52%**	**42%**
		**Mean Forecasted Grades**	Pre-2010	A-E	**20%**	**42%**	**39%**
		**Mean Predicted Grades**	Post-2010	A*-E	**10%**	**17%**	**73%**
		**Mean Forecasted Grades**	Post-2010	A*-E	**15%**	**46%**	**39%**
**Medical school applicants and students: Alevels and other qualifications**							
	Students: Lumb & Vail [38]	Predicted grades	1995	A-E	7%	52%	41%
	Applicants: Alevels (this study)	Predicted grades	2010-18	A*-E	7%	49%	45%
	Applicants: EPQ (this study)	Predicted grades	2010-19	A*-E	14%	52%	34%
	Applicants: SQA Adv. Highers (this study)	Predicted grades	2010-18	A-D	3%	60%	38%
		**Mean Predicted Grades (Medics)**			**8%**	**53%**	**39%**
**GCSE grades: All candidates**							
	Gill and Chang (2015) [36]	Forecasted GCSE Grades	2013	A*-G,U	12%	47%	41%
	Gill and Benton (2015) [35]	Forecasted GCSE Grades	2014	A*-G,U	14%	44%	42%
		**Mean Forecasted GCSE grades**			**13%**	**45%**	**42%**

For peer review only - http://bmjopen.bmj.com/site/about/guidelines.xhtml

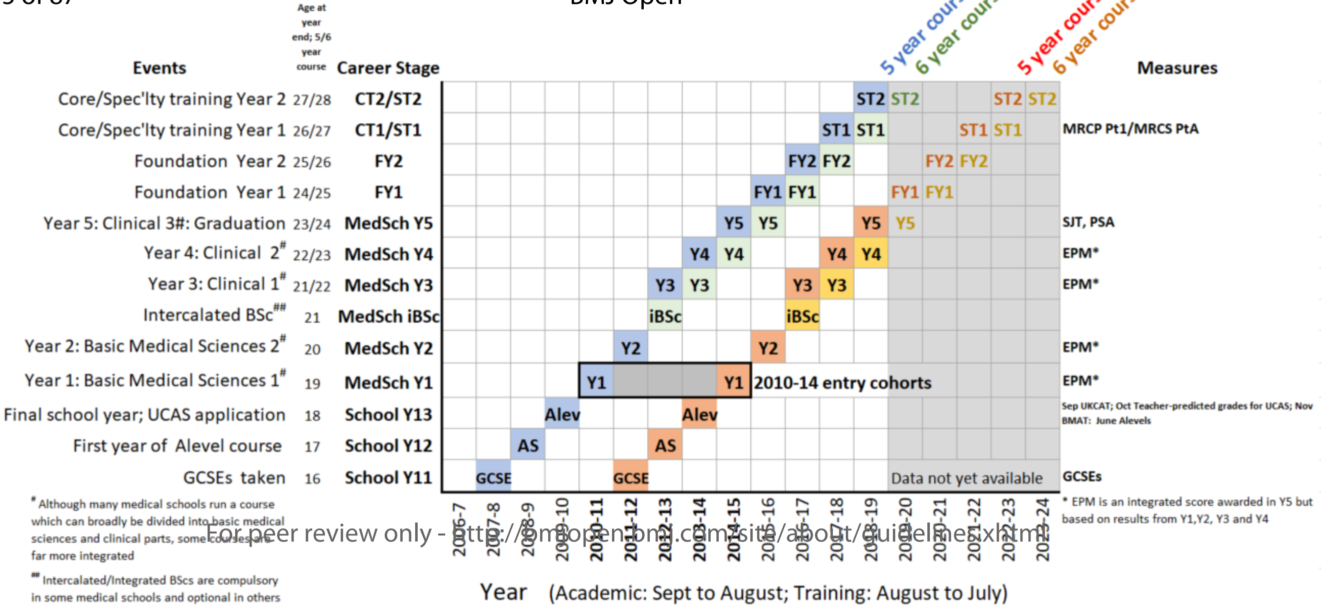

For peer review only - http://bmjopen.bmj.com/site/about/guidelines.xhtml

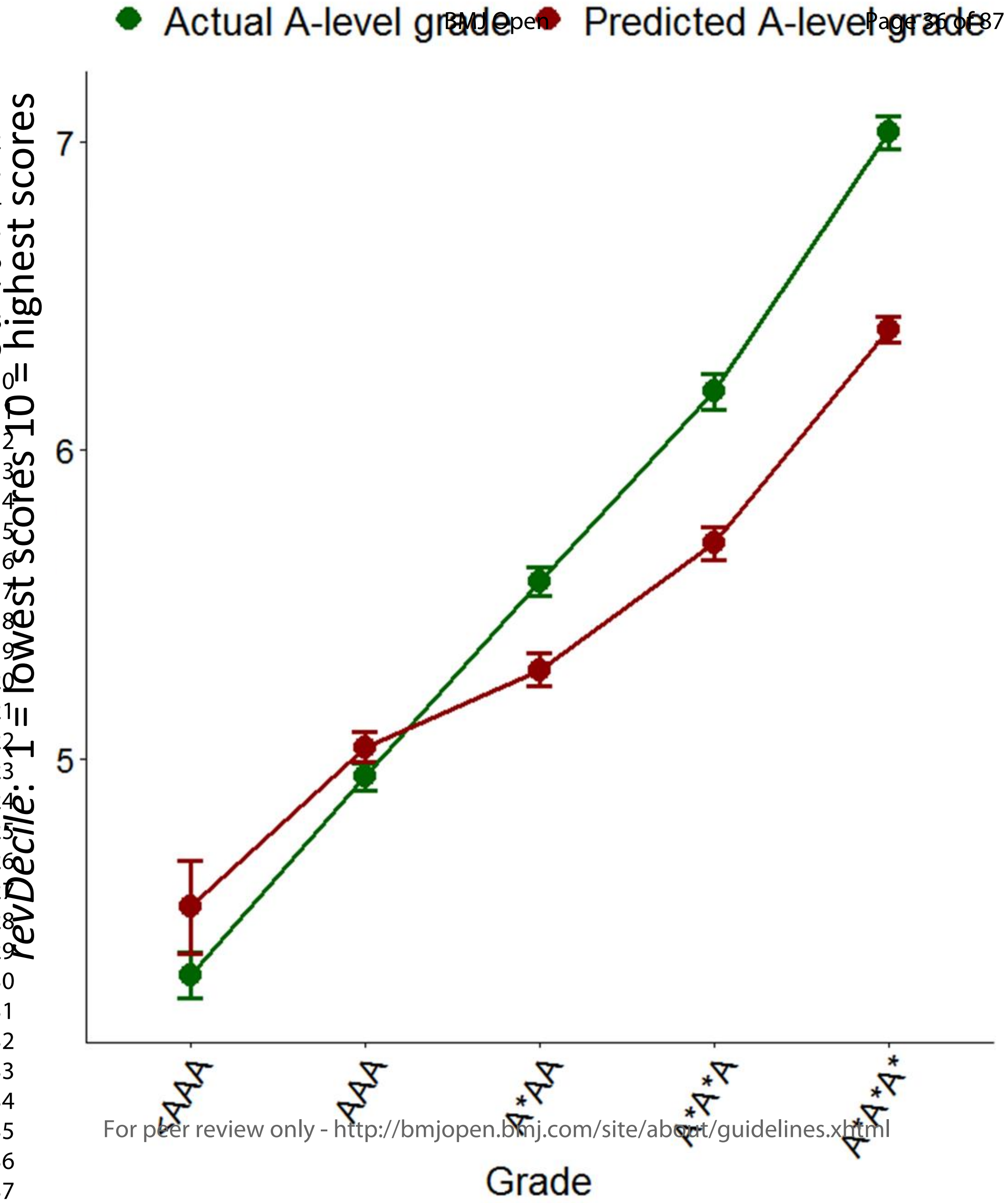
Actual A-level grade
Predicted A-level grade
revDecile: 1 = lowest scores 10 = highest scores
7
6
5
<AAA
AAA
A*AA
A*A*A
A*A*A*
Grade

			Selection measures applicants					Undergraduate outcome measures			Postgraduate outcome measures	
			GCSE grades	Predicted Alevels	Alevel grades	UKCAT	BMAT	EPM	SJT	PSA	MRCP(UK) Part 1	MRCS Part A
**Selection measures in all applicants**	**GCSE grades**	r	1	0.452	0.421	0.265	0.223	0.180	0.190	0.201	0.212	0.173
		N		22150	22150	22145	4935	12230	12185	12265	890	430
	**Predicted A-level grades**	r	0.452	1	0.585	0.272	0.326	0.198	0.160	0.226	0.283	0.181
		N	22150		22955	22520	5225	12560	12515	12600	910	440
	**Attained A-level grades**	r	0.421	0.585	1	0.326	0.416	0.297	0.195	0.306	0.421	0.358
		N	22150	22955		22520	5225	12560	12515	12600	910	440
	**UKCAT total**	r	0.265	0.272	0.326	1	0.483	0.115	0.243	0.238	0.200	0.181
		N	22145	22520	22520		5080	12385	12340	12420	900	435
	**BMAT sections 1 and 2**	r	0.223	0.326	0.416	0.483	1	0.089	0.239	0.321	0.378	0.319
		N	4935	5225	5225	5080		4850	4840	4875	450	240
**Undergraduate outcome measures**	**UKFPO EPM decile**	r	0.180	0.198	0.297	0.115	0.089	1	0.319	0.470	0.509	0.535
		rTPa	0.213	0.251	0.403	0.149	0.101	-	-	-	-	-
		N	12230	12560	12560	12385	4850		12515	12505	905	440
	**UKFPO SJT score**	r	0.190	0.160	0.195	0.243	0.239	0.319	1	0.346	0.351	0.274
		rTPa	0.223	0.203	0.267	0.310	0.267	-		-	-	-
		N	12185	12515	12515	12340	4840	12515		12475	905	435
	**PSA score**	r	0.201	0.226	0.306	0.238	0.321	0.470	0.346	1	0.500	0.483
		rTPa	0.236	0.287	0.415	0.305	0.360	-	-	-	-	-
		N	12265	12600	12600	12420	4875	12505	12475		910	440
**Postgraduate outcome measures**	**MRCP(UK) Part 1**	r	0.212	0.283	0.421	0.200	0.378	0.509	0.351	0.500	1	...
		rTPa	0.272	0.360	0.601	0.273	0.398	0.586	0.391	0.576	-	-
		N	890	910	910	900	450	905	905	910	-	10
	**MRCS Part A**	r	0.173	0.181	0.358	0.181	0.319	0.535	0.274	0.483	...	1
		rTPa	0.196	0.216	0.519	0.282	0.313	0.618	0.306	0.575	-	-
		N	430	440	440	435	240	440	435	440	10	-
**All outcome measures** (unweighted mean)	**Undergraduate (n=3)**	r	0.190	0.195	0.266	0.199	0.216	-	-	-	-	-
		rTPa	0.224	0.247	0.362	0.255	0.243	-	-	-	-	-
	**Postgraduate (n=2)**	r	0.193	0.232	0.390	0.191	0.349	0.522	0.313	0.492	-	-
		rTPa	0.234	0.288	0.560	0.278	0.356	0.602	0.349	0.576	-	-
	**Undergraduate and Postgraduate (n=5)**	r	0.191	0.210	0.315	0.195	0.269	-	-	-	-	-
		rTPa	0.228	0.263	0.441	0.264	0.288	-	-	-	-	-
**Range restriction (uX) = SD(entrants)/SD(applicants)**	**EPM, SJT & PSA**	uX	*0.955*	*0.958*	*0.888*	*0.890*	*0.994*	-	-	-	-	-
	**MRCP(UK) Pt1**	uX	*0.833*	*0.954*	*0.883*	*0.842*	*1.055*	*0.962*	*0.997*	*0.957*	-	-
	**MRCS Part A**	uX	*0.985*	*0.998*	*0.835*	*0.761*	*1.123*	*0.946*	*0.958*	*0.927*	-	-

For peer review only - http://bmjopen.bmj.com/site/about/guidelines.xhtml

		UKFPO		Selection grades:			Absolute	Relative
	Decile	score	*RevDecile*	Attained	Predicted	Odds Ratio	difference	increase
Worst	10	34	1	7.2%	8.1%	1.141	0.9%	13.0%
	9	35	2	9.4%	10.6%	1.135	1.1%	12.0%
	8	36	3	10.1%	11.1%	1.107	1.0%	9.5%
	7	37	4	10.7%	11.2%	1.052	0.5%	4.6%
	6	38	5	10.7%	10.8%	1.003	0.0%	0.3%
	5	39	6	10.6%	10.4%	0.978	-0.2%	-2.0%
	4	40	7	10.7%	10.4%	0.970	-0.3%	-2.7%
	3	41	8	10.3%	9.7%	0.935	-0.6%	-5.8%
	2	42	9	10.2%	9.1%	0.882	-1.1%	-10.7%
Best	1	43	10	10.1%	8.8%	0.853	-1.4%	-13.4%

For peer review only - http://bmjopen.bmj.com/site/about/guidelines.xhtml

The predictive validity of A-level grades and teacher-predicted grades in UK medical school applicants: A retrospective analysis of administrative data in a time of COVID

Supplementary information

Contents

1. Supplementary literature review, including a summary of events from March to November 2020. 2

2. Supplementary Methods including a table of measures 11

3. Supplementary Results including the Extended Project Qualification (EPQ) and SQA Advanced Highers. 13

4. Supplementary Tables 1, 2, 3, 4, 5 & 6 and Supplementary Figures 1, 2, 3, 4 & 5 20

5. *Appendix*: Are independent (private sector) schools more accurate in their A-level predictions?.. 32

6. Appendix: Appendix Tables 1 & 2 and Appendix Figures 1, 2, 3 & 4. 36

References 43

Note: The Supplementary Material contains extended versions of the Literature Review from the main paper, the Method section of the main paper, and the Results section of the main paper. In order to maintain the flow and continuity of the supplementary material, some of the material is duplicated from the main paper. In addition to the supplementary literature review, methods and results, there is also an appendix which looks in depth at the issue of whether independent (private sector) schools are more accurate in their A-level predictions.

For peer review only - http://bmjopen.bmj.com/site/about/guidelines.xhtml

1. Supplementary literature review, including a summary of events from March to November 2020.

Overview of literature on predicted, forecasted and attained A-level grades. The majority of studies reported here are also discussed in the main paper, in much more abbreviated form, but here are described more discursively.

University applications in general

Petch in 1964 [1] did what Wilmut has described as "one of the earliest and most celebrated studies of teacher estimates of examination result" [2] (p.60), describing how Petch found, "grade agreement in about 43% of cases, but the examination grade was higher than the teacher estimate in 18% of cases, but lower in 39% of cases, sometimes heavily so".

Two other early studies were by Murphy in the first of which in 1979 he compared actual and predicted grades both for A-levels and, unusually, for O-levels (the predecessor of GCSEs) [3], including two-way tables of predicted vs actual grades. Of 291 results the predicted grades were accurate in 27% of cases, over-predictions in 44% and under-prediction in 29% of cases. Teachers were also asked to provide a rank order of students, and overall these correlated 0.6 with rank order in the examination, although individual teachers showed a range of correlations from just less than zero through to more than 0.9. Murphy's 1981 study drew on application forms submitted to UCCA (now UCAS) by 15,109 candidates, of which "a large number included teachers' pre-examination estimates of A-level grades" (with predicted grades being A, A/B, B, B/C, C etc). Results were broken down by exam board and also by subject. The overall correlation of predicted and actual grades was 0.66, with Physics, Chemistry and French showing the highest correlations. The study also looked at A-level – O-level correlations [4]. Although described as predicted grades, these data are actually best described as being *forecasted grades*.

More recent studies have mostly been concerned with the relationship of attained A-level grades and the predicted A-level grades entered on UCAS application forms by teachers. UCAS changed the way it collected such data in 2009, so that for UK-domiciled applicants subject-level predicted grades were available, rather than as earlier when predicted grades were only available as total point scores [5]. For various reasons, not all A-levels have predicted grades. Most analyses are for candidates across all ability levels. Note that A* grades were only introduced in 2010.

In a study of 2009 applicants [5], overall *accuracy* at the subject level for A-levels for 219,744 A-levels was 52%, with predicted and attained grade being the same. In 42% of cases predicted grades were over-estimates, and in only 7% were they under-estimates. A grades tended to be predicted more accurately but that in part reflects that A grades cannot be under-predicted (or E grades over-predicted).

Female candidates showed a slight tendency for grades to be more accurately predicted (52.3% vs 51.1% in males). Socio-economic group showed strong relationships to accuracy, with 58% accurate predictions in the Higher Managerial group and 43% in the Routine group, but that in part reflects different actual A-level achievement (58% of Managerial candidates receiving an A grade compared to 33% of Routine candidates). The Higher Managerial group had the greatest over-prediction and the Routine group the highest under-prediction. Considering ethnicity, 53% of White applicants had accurate predictions compared with 47% of Asian ethnicity, and 39% of those of Black ethnicity. Centre (school) was related to accuracy, with 64% accuracy in Independent schools, 47% in state schools, and 40% in those in Further or Higher education. The authors note that multivariate analyses are probably needed to tease apart the relationships between the various correlates of accuracy. Other analyses looked at disability, region, and nation within the UK. Number of choices also related to accuracy, applicants making four choices being more accurate than those making five choices, but it was suggested that was because of the majority of the former being higher attainers applying to Medicine, Dentistry or Veterinary Medicine. The paper concluded that it is difficult to

For peer review only - http://bmjopen.bmj.com/site/about/guidelines.xhtml

separate out the various factors involved in accuracy, not least because of the ceiling and floor effects for high and low attainers [5].

Wyness [6] analysed aggregated data provided by UCAS for the applicants from 2013-15, and hence A* grades were included in the analysis. Overall only 16.1% of grades were accurately predicted, a much lower figure than the earlier study using 2009 data [5], perhaps because of the inclusion of the new A* grades. 8.54% of grades were under-predicted, while 75.4% of grades were over-predicted. As with the 2009 data, there was a clear relationship between over-prediction and attained grade, although it is noted that there are strong ceiling effects at work. As with the 2009 study, independent schools provided the most accurate predictions. Applicants from disadvantaged backgrounds showed moderate to severe over-prediction. Asian and Black applicants were also more likely to be severely over-predicted. There were no differences between male and female applicants. The report is particularly interesting as it looks at prediction in high ability students, defined as AAB or above. The difference between the most and least disadvantaged in this group is much smaller, with 44.0% overpredicted in the most disadvantaged and 47.4% in the least disadvantaged. There was some evidence that under-predicted applicants tended to show under-matching (i.e. entering less competitive universities than their actual grades might predict). Further analyse and discussion of these data are provided elsewhere [7 8].

UCAS in 2017 provided some limited data on over-prediction and under-prediction of A-levels since the introduction of A* grades, with data for 2012, 2016 and 2017 [9]. Overall 19.5%, 16.3% and 16.0% of predictions were accurate, with over-prediction in 68.4%, 74.3% and 73.3% of cases, and under-prediction in 11.8%, 9.0% and 10.4% (figures from EoC17_Figure7_9_database.csv[a]). UCAS commented that, the gap between achieved and predicted A-level grades, “continues to widen” (p.23), although a comparison of 2016 and 207 results concluded that there was little effect effect due to the reforms in A-levels that took place in 2017.

Not all studies have used the *predicted grades* provided to UCAS for use by universities in selection, which for medical school applicants would have been by mid-October). Until 2015 teachers were also asked, by the end of the following May, just before A-levels were sat, to provide *forecasted grades* to Awarding Organisations, and those grades then contributed in part to decisions on grading. Forecasted grades are clearly of particular interest given proposals for calculated grades to be based on estimates of performance by schools during May. Three analyses are available, for candidates taking A-levels in 2009 [10], 2012 [11] and 2014 [12] which are before and after A* grades were introduced. A primary interest must be the comparison of these forecasted grades with the more usually studied predicted grades, described earlier for 2009 [5] and 2012 [9]. Note that the studies of forecasted grades are only for OCR (Oxford, Cambridge and Royal Society of Arts Examination Board) and hence include all A-level candidates, whereas the studies of predicted grades are for university applicants. Supplementary table 1 compares the two sets of predictions. In 2009 there is little difference between predicted and forecasted grades in accuracy, with a small diminution of over-predictions. The picture three years later, in 2012 after A* grades have been introduced, is rather different. Forecasted grades have an accuracy of 48% compared with only 20% for predicted grades. Taken overall it is difficult to reconcile the two studies which are only three years apart. Based on the 2009 data it would seem that predictions in May are no more accurate than those in October, whereas the 2012 data suggest that May predictions are much more accurate than October predictions. Having said that, even in May 2012, slightly less than a half of forecasted grades are accurate, with the same grade as in October.

It should be noted, as pointed out earlier, that the early studies by Murphy should probably be regarded as being of forecasted and not predicted grades.

[a] https://www.ucas.com/file/140426/download?token=tUxAGXtt

For peer review only - http://bmjopen.bmj.com/site/about/guidelines.xhtml

Grade point predictions. The analyses described so far have been at the level of A-level subjects. Students mostly take three or sometimes more A-levels, and universities usually look at the three best grades attained. Scoring grades as A*=12, A=10, B=8, C=6, D=4 and E=2 then a candidate passing three A-levels will score between 6 and 36 points for their three best grades[b]. Two studies [10] [11] have pointed out the difficulty of using totalled points. As an example, a candidate predicted AAA will be predicted 30 points but may attain grades AAA or grades A*A*D; both are equally accurate in point terms but not in grade terms. Total predicted points are important in that UCAS for a number of years only provided total predicted points for the best three A-levels, without subjects or individual grades being specified[c].

UCAS in 2016 reviewed predicted and actual A-level grade points in applicants from 2010 to 2015 [13] considering the best three grades attained. Achieved grades were one or two grades in total lower for attained than predicted grades. About a half of applicants in 2015 missed predicted total grades by two or more grades (e.g. ABB rather than AAA), a proportion that had increased by a third since 2010. Simple analyses in particular showed that missing predicted grades was associated with having *lower* predicted grades overall (as in the earlier analyses at the subject level). Multivariate analyses i.e. taking other factors into account, found missing predicted grades was associated with having *higher* predicted grades, lower GCSE attainment, taking biology, chemistry and maths, having Asian, Black, Mixed and Other ethnicity, coming from disadvantaged areas, being female, and having '[pre-A-level]unconditional offers'. Of particular interest is the relationship to GCSE grades, which have a strong relationship to A-level attainment [14] which is clearly seen in the UCAS data (see their figures 5 and 6).

What are predicted grades and how are they made?

UCAS, in its document, "Predicted grades – what you need to know"[d] says that "A predicted grade is the grade of qualification an applicant's school or college believes they're likely to achieve in positive circumstances." Later the document says predicted grades should be, "**in the best interests of applicants** – fulfilment and success at college or university is the end goal ", and "**aspirational but achievable** – stretching predicted grades are motivational for students, unattainable predicted grades are not" (all emphases in original). It also says that grades should be "determined by professional judgement" and be data-driven, including "past Level 2 and Level 3 performance, and/or internal examinations to inform your predictions".

Gill [15] has described the relatively sparse literature on how teachers estimate grades. Gill's own study followed the methodology of Child and Wilson[e] although that study is not in the public domain. Gill sent questionnaires in May to selected OCR exam centres concerning Chemistry, English Literature and Psychology, and as well as estimating grades teachers were also asked to rank within grades, the method currently being adopted by Ofqual for calculated grades[f]. Teachers also

---

[b] Some studies, including my own earlier ones, score A*=6, B=5, etc.. Such schemes became less popular with the advent of AS-grades, which were scored as half of an A-level, and hence it made sense to double the points available for a full A-level so that totals remained integer. With the near disappearance now of AS-levels that rationale makes less sense.

[c] Earlier studies, such my 1991 cohort, had to extract predicted grades from UCAS references, and hence they are often embedded in free text, making it difficult to match them up with specific A-level subjects.

[d] https://www.ucas.com/advisers/managing-applications/predicted-grades-what-you-need-know [Accessed 13th April 2020].

[e] Child S, Wilson F. An investigation of A level teachers' methods when estimating student grades. Cambridge: Cambridge Assessment (Unpublished document, October 2015).

[f] One teacher refused to take part because of the difficulty of ranking 260 students sitting one exam. Another teacher commented, "it was easier for smaller centres to make predictions because they know individual students better" (p.42). The paper in fact comments that, "Responses to the questionnaire were more likely to come from smaller centres. … [T]he maximum centre size amongst the sample data was only 40 for Chemistry

For peer review only - http://bmjopen.bmj.com/site/about/guidelines.xhtml

indicated the evidence they had used for each decision. The response rate was extremely low (2.8%). About 45% of forecasted grades were accurate (which is similar to the 48% in supplementary table 1). Detailed A-level raw marks were also available and could be correlated with rankings, giving correlations of .87, .76 and .83 for the three subjects. Those correlations are high, and certainly are higher than a meta-analytic estimate of the effect size for teachers predicting academic achievement in pupils of 0.63 (SE=.03), although there was substantial heterogeneity. They are also higher than Murphy's 1979 estimate of 0.66 for the correlation of rankings and exam marks [3]. The most important information said by teachers to be used when predicting grades was performance in mock exams, and observations of quality of work and commitment, with oral presentation also important. Amongst other topics written in, the most important was the opinion of other teachers both in the same subject and other subjects, including the head of department. Other teachers raised concerns about the lack of high stakes for mock exams which meant that students did not treat them seriously. There were also concerns about the loss of AS-levels to help in prediction.

Other examinations. We know of no studies that have looked at accuracy of prediction of Scottish Highers or Advanced Highers, of the EPQ (Extended Project Question) used in England, or of other examinations carried out in the UK.

Applications to medical school

Relatively few studies have looked at predicted grades in medical school applicants, although those studies do show a tendency to ask rather more stretching questions, perhaps because of the different interests of the researchers, and the specificity of the course and its outcomes.

Lumb and Vail pointed out that predicted grades are particularly important in the shortlisting phase of medical student selection [16]. They studied 1661 applications in 1995 to a single medical school who had estimated grades for 5053 A-levels, 52% of predictions being accurate, 41% were over-estimated and 7% under-estimated [16]. The authors presented an ROC curve (but not the area under the curve), and concluded that, "… selectors for medical schools can have some confidence in the accuracy of predictions and we should therefore continue to use them … [for] selecting the doctors of the future." (p.311).

Richardson et al, studied 721 entrants from 1991 to 1994 to a single medical school [17]. Unusually they looked at predictive validity, assessing how well predicted and actual A-level grades related to a composite outcome on the pre-clinical course. Predicted and actual A-level grades showed a minimal correlation (r=0.024), but selection would have imposed range restriction. Pre-clinical exam performance correlated 0.318 (p<.001) with attained A-level grades, but only 0.041 (NS) with predicted A-level grades. This is a rare study in which predictive validity was assessed and it implied that selection should be on actual grades rather than predicted grades, concluding in contra-distinction to Lumb and Vail that, "medical school admissions panels would be well advised to take the predicted grade with a sizeable pinch of salt" (p.296).

A third study, by one of the present team, took a different approach, using path modelling to assess the causal inter-relationships between GCSE grades, predicted A-level grades, receipt of an offer, actual A-level grades, and acceptance at medical school in an original sample size of 6901 applicants to five English medical schools [18]. A-level estimates were predicted by GCSE grades (beta=0.89), with attained A-level grades predicted by both GCSE grades (beta=0.44) and predicted A-level grades (beta=0.74). A substantive question of interest was whether the paths in the model differed between White and non-White candidates, with it being shown that none of the relationships described showed ethnic differences (although non-white candidates were significantly less likely

(compared with 423 amongst all centres), 26 for English Literature (compared with 180) and 32 for psychology (compared with 378)."

For peer review only - http://bmjopen.bmj.com/site/about/guidelines.xhtml

than White candidates to receive an offer based on predicted A-level grades). Although the study reported no follow-up into the medical course, this dataset is analysed further below to assess predictive validity for postgraduate examination performance.

A comment on issues in studying predicted A-level grades.

Although predicted A-level grades have been an integral part of university application and selection in the UK for four decades, obtaining data on them is less than easy. Early studies, including my own, as well as those of other medical researchers, simply resorted to having researchers transcribe grades from paper UCCA and UCAS application forms, although often that was not easy in earlier forms as the predictions were often embedded in the free text of the Referee's Statement. Until 2009 UCAS only recorded the summed score of the best three A-levels, so that study of specific subjects was not possible. Even now obtaining UCAS data on predicted grades is less than easy, and Boliver in 2013 comments, "It would have been desirable to include predicted A-level grades… . Unfortunately UCAS are unable to provide this information in microdata form because of uncertainty about its validity in the case of applicants whose application is not linked to a school or college … (personal communication from UCAS)." [19]. Similarly Wyness in 2016 in her study of three years of UCAS data comments that, "The data are aggregate (for reasons of privacy)" [6], which means of course that proper analyses at the level of individual participants are not possible. There is an irony here in that of course all universities have access to predicted grades provided by UCAS as a part of the admissions process, but subsequently obtaining those data for research is often very difficult. The data for the present study are the result of an important collaboration between UKMED and UCAS, with UCAS providing detailed information on applicants to UK medical schools for inclusion in the database, which is hosted in a safe haven to ensure strict controls on access; we are very grateful to UCAS for that collaboration without which the present study would not be possible.

A summary of events surrounding the cancellation of Alevels from March to November 2020.

The research for the present paper was carried out in April and May 2020, in parallel with the study of attitudes and responses of medical school applicants to the cancellation of A-level examinations and their replacement by 'Calculated Grades'[20 21]. The main bulk of the present paper was written between March and June 2020, with a preprint being published in June 2020[22]. Key findings from this paper and the accompanying applicant attitudes paper were presented to UK medical admissions tutors at a meeting of the MSC-SA (Medical Schools Council Selection Alliance) on 6th May 2020, and drafts of the two papers distributed. The present paper is in large part a statement of how we understood the situation in May 2020, with a few amendments for clarity.

Inevitably events, in large part political but also with many practical ramifications for medical schools and student selection, continued onwards from June 2020, particularly with the publication of Alevel results in August 2020, and the abandonment of the algorithm for calculating A-level grades, with its inevitable fallout. The following paragraphs provide a summary of events, both going forward and also, to some extent, looking back to March 2020 as a result of documents published in September and October 2020.

In July and August 2020 things moved rapidly, with dramatic changes taking place. It would have been extremely confusing and probably misleading to have tried to incorporate those changes into the text of the main paper. Instead we hope this postscript will give readers a sense of what happened, to what extent events were correctly or incorrectly predicted by us, what impact the present paper may have had, and what may be the implications for the future.

For peer review only - http://bmjopen.bmj.com/site/about/guidelines.xhtml

The story is best told chronologically and we mostly use reports from newspapers, and refer interested readers to a brief summary on Wikipedia[g] and a journalistic review on the BBC website[h].

The awarding of A-level grades in 2020: the story from March to November 2020

As described in our main paper, on 20th March 2020 public examinations in the UK including A-levels were cancelled. On 3rd April *Ofqual* announced that exam grades in England would be replaced with Calculated Grades. Calculated Grades were to consist of Centre Assessment Grades (also called Centre Assessed Grades or CAGs), estimated by teachers that centres (mostly schools and colleges) would submit to *Ofqual*. *Ofqual* would moderate these CAGs using an algorithm – the details of which had not yet been published but, it was stated, would be based on the prior performance of pupils within schools. The Scottish Qualification Authority (SQA), Qualifications Wales, and the Northern Irish Council for Curriculum, Examination and Assessment (CCEA) also announced that they would use a broadly similar approach to that of *Ofqual*.

Schools (centres) had to return their teacher-estimated CAGs to *Ofqual* in June 2020. On June 16th a report in The Times said that "Teachers have marked too generously in allocating GCSE and A-level grades this year, research suggests" (The Times, 16th June), the article being based on a report from FFT Education Datalab, which actually had only asked about GCSEs, and had no data on A-levels[i] . In July a Guardian article reported a statement from *Ofqual* that "a substantial number of students would receive at least one adjusted grade – usually downwards – as a result of a standardisation process" although they "sought to allay fears that certain groups of pupils, … could be disadvantaged by calculated grades. *Ofqual* said their analysis had found no evidence of widening of gaps in attainment".(*The Guardian (G),* 21st July).

SQA results in Scotland are announced a week before those in England, so the SQA results announced on August 5th 2020 gave a preview of what was to come the following week in the rest of the UK. The Scottish results were immediately controversial when it emerged that the moderation of teacher-estimated grades (CAGs) by an algorithm had resulted in a quarter of grades being adjusted downwards. The Scottish Education Secretary, John Swinney, said that without those adjustments the pass rates would be up on the previous year by 14% for Highers and 13.4% for Advanced Highers. He added that, "… these robust processes mean we have upheld standards… All exam systems rely on an essential process known as moderation to uphold standards. This ensures an A grade is the same in every part of the country, making the system fair for everyone, and across all years." *(G*, 4th August).

Teachers, students, parents and the media were unhappy with the moderation. A *Daily Telegraph* editorial entitled "Exam moderation is a gross injustice" attacked statistical modelling in general, and the SQA process in particular, which "gives poorer marks to children living in deprived areas … [without] recognition of individuals who buck the general trend" (*Daily Telegraph (DT)*, 5th Aug), and asked "Is the same fiasco about to be inflicted on A-level students in England and Wales?". By 11th August, students in Scotland were protesting on the streets, Nicola Sturgeon, the First Minister, was apologising for the exam results debacle, and the Scottish government was facing a vote of no

[g] https://en.wikipedia.org/wiki/2020_UK_GCSE_and_A-Level_grading_controversy

[h] Coughlan,S. "Coronavirus: The story of the big U-turn of the summer", https://www.bbc.co.uk/news/education-54103612
[i] https://ffteducationdatalab.org.uk/2020/06/gcse-results-2020-a-look-at-the-grades-proposed-by-schools/

For peer review only - http://bmjopen.bmj.com/site/about/guidelines.xhtml

confidence (*G*, 11th Aug). Expectations in the media of problems with A-levels were also growing. On 12th August it was announced in Scotland that the teacher-estimated CAGs downgraded by the SQA algorithm during moderation "would be reinstated" (*G*, 12th Aug).

In an attempt to prevent problems with A-levels, on August 12th 2020 the English education secretary, Gavin Williamson announced "a triple lock" for A-level students, whereby students could accept their Calculated Grade results, use the results of mock exams (practice exams which students take in schools), or use the results of real exams due to take place in Autumn 2020 after the start of the university academic year (*The Times*, (T), 12th August; *T*, 13th August). Protests were immediate as mock exams vary immensely, and many schools had been encouraged to cancel mock exams as a part of the Covid lockdown in March 2020.

A-level Calculated Grades (i.e. the teacher-estimated CAGs adjusted by the algorithm during moderation) were announced on 13th August 2020, and UCAS announced which students had obtained places at their chosen university based on these Calculated Grades. An immediate problem arose: following the Scottish government's reversal, students in Scotland now had unadjusted SQA grades, which were higher on average and gave them an advantage over applicants with A-level Calculated Grades, which had been adjusted (*G,* 13th Aug). University admission processes were also becoming embroiled in confusion, and although universities had, "reassured ministers that they will 'soften' the grades they normally require" (*T*, 13th August), by the next day universities were accused of being inflexible (*G*, 14th Aug).

It soon became apparent that schools in the private, fee-paying sector, had probably benefited from the algorithm, primarily because statistical predictions were less accurate for the small class sizes more likely to be found in private schools, and in those cases the teacher-estimated CAGs had been allowed to stand unadjusted. Although the Prime Minister Boris Johnson defended the system saying, "Let's be in no doubt about it: the exam results that we've got today are robust, they're good, they're dependable for employers" (*G*, 14th August), many backbench MPs were in revolt, having been deluged with complaints from constituents (*T*, 14th August). The next day Gavin Williamson said, "No U-turn. No change" (*T*, 15th August), and although he did agree to waive fees for appeals against Calculated Grades, he insisted that the grades themselves would not change in order to avoid the grade inflation that had occurred in Scotland (*T*, 15th August).

Meanwhile the *Ofqual* algorithm, published in a document over 300 pages in length, was being dissected carefully, and when one headteacher anonymously shared their school's results, the problems became particularly apparent[j]. In the previous three years at their school, 12.5% of pupils had achieved A* and none had got a U; however the algorithm meant only 3.7% of students (equivalent to just one student) received an A* - much below the historic 12.5%. The algorithm also resulted in one student being awarded a U, despite no students at that school having received a U previously. The weekend newspapers attacked the government, "which deserved a U grade for this debacle" (Sunday Times (ST), 16th August). GCSE results, due on Aug 20th, were also on the horizon, with similar problems predicted (*T*, 15th Aug, p.14; *Observer (O)*, 16th August). Students in England demonstrated outside the Department for Education in London. *Ofqual* also announced guidance on the role of mock exams in appeals only to withdraw it a few hours later (*T*, 17th August).

[j] Hern,Alex. (2020) "Do the maths: analysis shows why England's grading system is both imprecise and unfair", *Guardian*, 15th August, 2020, p.13; the analyses are based on Thomson, Dave, "A-Level results 2020: How have grades been calculated?", 13th August 2020, https://ffteducationdatalab.org.uk/2020/08/a-level-results-2020-how-have-grades-been-calculated/.

For peer review only - http://bmjopen.bmj.com/site/about/guidelines.xhtml

On August 18th the government scrapped *Ofqual's* algorithm and reverted to unadjusted teacher-estimated CAGs (*G*, 18th August). *The Times*, normally a supporter of the Conservative Party, simply called its main editorial, "Another Fine Mess" (*T*, 18th August). The chairman and chief executive of *Ofqual* were criticised for having little experience of education (*T*, 19th August), and the Chief Executive eventually resigned on August 25th.

Several other problems now emerged, not the least being that universities would not know the (now unadjusted CAG) grades for several days. Once universities did receive these grades, they found that they did not have enough places to honour all the offers they had made students months earlier. This was because universities typically make more offers than they have places, knowing that a significant number of students will not meet those offers when they achieve lower exam grades than the Predicted Grades estimated by their teachers and submitted to UCAS when they apply to university. But now with the teacher-estimated CAGs replacing exam grades, and the resulting increase in the percentage of A and A* grades from 28% to 38%, many more students than expected did in fact meet their university offers (*T*, 18th August). This caused problems for some students whose Calculated Grades (the grades adjusted by the algorithm) had been too low for their first choice university and so had accepted offers from their second choice, but now they found their unadjusted teacher-estimated CAGs enabled them to meet their first choice offer they inevitably wanted to go there. Other students seemed to have been left in limbo, needing to delay entry until the next year, with a potential knock-on effect for students taking A-levels in 2021 (*T*, 19th August). Some groups of A-level students had clearly fallen through the net and were in limbo, such as who were home-schooled or private A-level students, having no teachers to estimate their grades (*G*, 21st August).

Regarding medical schools specifically, *The Guardian* reported that the Norwich Medical School at the University of East Anglia had 185 places and a possible overshoot of 50, a 27% increase, emphasising that with medical school numbers more strictly limited than other university places and costing £50,000 a year this would have clear financial implications (*G*, 19th August). A news story based in part on the current research as published in pre-print on *medRxiv,* suggested that more medical students may be liable to drop out, a co-chairman of the Medical Schools Council suggesting that "we are going to have, on average, students with lower grades than in previous years" (*DT*, 20th August). A similar concern was also raised by headteachers in relation to GCSE grades, which it had now been announced would also be based on unadjusted teacher estimates, with fears that students, "could end up on unsuitable courses post-16 which could set them up for failure" (*G*, 21st August).

The cap on university student numbers was released on 17th August, the medical school cap following on 20th August, resulting in a bulge of new undergraduates. There was also a bulge in admissions to sixth form colleges as a result of unadjusted teacher-estimated GCSE grades being higher than the expected exam grades (*G*, 21st August). There are financial implications for educational institutions but no-one would know the size of the problem until UCAS released its entry statistics for October 2020.

The controversies rumbled on into September 2020 as the House of Commons Select Committee on Education heard evidence from *Ofqual* and other bodies. *Ofqual* put out a lengthy statement on 2nd September in evidence to the Committee[k]. It set out the history according to the regulator, stating that in March its advice to the Secretary of State had been that, "the best option in terms of valid

[k] https://www.gov.uk/government/news/written-statement-from-chair-of-ofqual-to-the-education-select-committee, "Written statement from Chair of Ofqual to the Education Select Committee", 2nd Sept 2020.

For peer review only - http://bmjopen.bmj.com/site/about/guidelines.xhtml

qualifications would be to hold exams in a socially distanced manner"; however, "The decision to use a system of statistical standardised teacher assessments was taken by the Secretary of State and issued as a direction to *Ofqual*". In reviewing the failure of the system, the conclusion was reached that, "a 'better' algorithm would not have made the outcomes significantly more acceptable. The inherent limitations of the data and the nature of the process were what made it unacceptable…. *To try to deliver comparable qualification results in the absence of students having taken any assessments (examinations) proved to be an impossible task*" (our emphasis). Cambridge Assessment's submission to Select Committee provides a detailed timeline of collaborative efforts to inform decision making.[l] With the model running and results being calculated, from late July through August, Cambridge Assessment worked with Ofqual and DfE to understand possible unfairness in the outcomes, and to put in place adequate remedy. No doubt the post-mortem will continue for a long while.

In autumn 2020, the needs of the next year began to be considered. One group, including several university Vice-Chancellors, argued that A-levels should be cancelled once more and replaced by teacher-estimated grades (*T*, 2nd October). Others argued more radically that teacher-estimated grades should permanently replace A-level examinations. On 12th October 2020 the Secretary of State announced that A-level exams would go ahead in England in summer 2021 with some minor changes, including being three weeks later than usual, and with results announced a few days later than typical[m]. Contingency measures would be in place for possible disruption, but were yet to be described, although a leaked newspaper report suggested that they might include more formal mock exams as a back-up when following earlier leaked reports (*G*, 10th October). Perhaps the most interesting comment by Williamson was that, "Exams are the fairest way of judging a student's performance …", with its tacit acceptance that teacher-estimated grades perhaps actually were not fair to many students.

To date other UK countries have taken different approaches. On 7th October the Scottish Government announced the cancellation of National 5 exams in 2021 although Higher and Advanced Higher examinations would be taken (BBC, 7th October). A month later on 10th November the Welsh Government announced that GCSE and A-level examinations in Wales would be cancelled, with grades being based on classroom assessments instead (BBC, 10th November). On the same day Education Minister Peter Weir announced that GCSE and A-levels examinations would be taken in Northern Ireland (BBC, 10th November). In November 2020 the Secretary of State announced his intention to consider post-qualification university admissions (http://researchbriefings.files.parliament.uk/documents/CBP-8538/CBP-8538.pdf).

This postscript has been relatively brief, given the complexity of the events, and it has not attempted to summarise events occurring in 2021, with teachers now mostly but not entirely responsible for awarding Alevel grades, but under some control by Ofqual. That story is probably too long, and not yet complete, for it to be included here. The history of the events of 2020 may however help those new to the issues to navigate through the major changes that occurred. In research terms, in medical education, higher education more generally, and in secondary education, there seems little doubt that researchers will be following in detail the outcomes for the cohorts affected by the dramatic changes which resulted in a giant, unplanned, experiment, where notional grades awarded

[l] https://committees.parliament.uk/writtenevidence/11358/default/, "Written evidence submitted by Cambridge Assessment", 2nd Sept 2020.
[m] https://www.gov.uk/government/news/students-to-be-given-more-time-to-prepare-for-2021-exams, "Students to be given more time to prepare for 2021 exams", 12th October 2020.

For peer review only - http://bmjopen.bmj.com/site/about/guidelines.xhtml

were probably different in many cases to what students would have been awarded in normal circumstances.

2. Supplementary Methods including a table of measures

Data for the present study comes from two separate primary sources:

"P89". UKMED project UKMEDP089, "The UK Medical Applicant Cohort Study: Applications and Outcomes Study", approved Dec 7th, 2018, with Dr Katherine Woolf as principal investigator, is an ongoing analysis as a part of UKMACS (UK Medical Applicant Cohort Study). Data are primarily concerned with the process of selection. In particular in the upload of 21st Jan 2020[n] there is detailed information from UCAS on all applicants to medical schools from 2007 to 2018, including all attained Key Stage 5 (Level 3) qualifications (e.g. A-levels and SQA) as well as teacher predicted grades for individual Key stage 5 qualifications.

"P51". UKMED project UKMEDP051, "A comparison of the properties of BMAT, GAMSAT and UKCAT", approved Sept 25th, 2017, with Dr Paul Tiffin as principal investigator, is an ongoing analysis of the predictive validity of admissions tests and other selection methods such as A-levels and GCSEs in relation to undergraduate and postgraduate attainment. A major feature of the study is the inclusion of data from UCAS, although in the13th May 2019 data upload, which was used here[o], UCAS predicted grades were only available as a composite, 18-point score, for application years 2010 to 2014. A new upload of the data in late April 2020 will provide more detailed information, but that will require quite extensive coding, etc., making it similar to the qualifications data for applicants in P89. For the present data upload, predicted A-level grades are in the old UCAS format consisting of a single number from 6 to 18 (i.e. 3 Es to 3 A*s using A*=6 coding). Outcome data for the P51 dataset are more extensive, and in particular include data for end of undergraduate training, including the UKFPO EPM measures, the UKFPO SJT as well as PSA (Prescribing Safety Assessment). Some data are available for later postgraduate examinations, but numbers inevitably are small for cohorts entering medical school in 2011 onwards.

A-level grade scoring. In both P89 and P51, A-level grades are expressed numerically on a standard scale of A*=12 points, A=10, B=8, C=6, D=4 and E=2, or have been rescaled to that score.

The table below provides a detailed description of the source and coding of the measures used in the analyses:

**Measure name**	**Description**	**Derivation**	**Source**
GCSE grades	Average GCSE score from the best 9 GCSEs	The sum of the nine best grades (counting double science as two separate GCSEs)/the number of GCSES in include – i.e. 9 or fewer. This is the methodology used by the UKCAT-12 study (McManus, I.C., Dewberry, C., Nicholson, S. and Dowell, J.S. (2013) "The UKCAT-12 study: educational attainment, aptitude test performance, demographic and socio-economic contextual factors as predictors of first year outcome in a cross-sectional collaborative study of 12 UK medical schools". *BMC Medicine* 11 (1), p. 244. ISSN 1741-7015. http://bmcmedicine.biomedcentral.com/articles/10.1186/1741-7015-11-244	GCSEs supplied to UKMED by UKCAT from data obtained from UCAS. We used the GCSEs associated with the first application.

[n] OUTPUT_UCAS_QUALS_DEC_20200121_1.TXT and OUTPUT_UCAS_QUALS_DEC_20200121_2.TXT (both dated 21/1/2020), and OUTPUT_UCAS_QUALS_VER.TXT (dated 5/12/2019).
[o] UKCAT51_APP_ALL_DATA_13052019_FILE1.SAV and UKCAT51_APP_ALL_DATA_13052019_FILE2.SAV (both dated 13/5/2019).

For peer review only - http://bmjopen.bmj.com/site/about/guidelines.xhtml

		Further details are available in McManus IC, Dewberry C, Nicholson S, Dowell J: *The UKCAT-12 Study: Technical Report. 2012*, UKCAT Consortium: Nottingham, https://www.ucat.ac.uk/media/1185/ukcat-technicalreport-march2012-withbackgroundandsummary-sep2013v3.pdf	
Predicted A-level grades	Predicted grades are provided by an applicant's teachers on the UCAS form the autumn prior to sitting A-levels.	UCAS supplied a score from the A-level grades declared by the applicant's teachers on the application. The highest three grades are considered only, adding up the following points per grade: A* = 6, A = 5, B = 4, C = 3, D = 2, E = 1. AS levels are not included. This variable was only available for 18-year-old applicants domiciled in England, Northern Ireland and Wales. To bring this into line with attained grades the number was multiplied by 2 to give a maximum of 36.<br><br>The value is taken from the 1st UCAS application present in the UCAS extract.	UCAS supplied data
Attained A-level grades	Total score from the best 3 A-levels.	Sum of the three highest A-level grades. Assign point scores to A-Level Grades in 2-point increments (A*=12, A=10, B=8. C=6, D=4, E=2, else=0). This is the methodology used by the UKCAT-12 study (see above). By using the best three A-levels we are able to accommodate the differing numbers of A-levels taken by applicants.<br><br>All grades were from 2010 onwards, when A* grades were available at Alevel, prior to that the maximum possible mark being A.	HESA qualifications data
UKCAT total	Total score on UKCAT test	A total scale score is generated by summing the individual scale scores of Verbal Reasoning, Decision Making, Quantitative Reasoning and Abstract Reasoning.<br><br>We used the score from the last available attempt which was therefore that associated with admission.	UCAT known as UKCAT at the time these data were created.<br><br>UCAT publish technical reports with the details. See: https://www.ucat.ac.uk/about-us/technical-reports/
BMAT sections 1 and 2	Combined BMAT score	BMAT_SECTION1 measures Aptitude and Skills.<br>BMAT_SECTION2 measures Scientific Knowledge and Applications.	BMAT published details of the test. See: https://foundationprogramme.nhs.uk/faqs/educational-performance-measure-epm-faqs/
UKFPO EPM decile	Medical school performance relative to peers ranked in deciles.	Students in a graduating cohort are ranked on their medical school performance (Educational Performance Measure, EPM). Individual schools decide which assessments to include in the EPM that meet the specified criteria and are required to consult with students and publish on their website the assessments included in that score.	UKFPO. Contained in the FP table in UKMED. See: Educational Performance Measure (EPM) 2019 Framework 2019. http://www.foundationprogramme.nhs.uk/sites/default/files/2018-07/UKFP%202019%20EPM%20Framework%20Final_0.pdf
UKFPO SJT score		The Situational Judgement Test (SJT) is a final year undergraduate test that assesses individuals' reactions to a number of hypothetical role-relevant scenarios, which reflect situations candidates are likely to encounter as a doctor. It seeks to provide a reliable measurement of the following non-academic domains: Coping with pressure, Working effectively as part of a team, Effective communication, Problem solving, and Commitment to professionalism.	UKFPO. Contained in the FP table in UKMED. See ISFP Project. Situational Judgement Test https://isfp.org.uk/sjt/ 14th February 2019.
PSA score	Score relative to pass on	The British Pharmacological Society and MSC Assessment developed the Prescribing Safety Assessment (PSA) that allows all UK medical	MSC Assessment provide these data to UKMED. See:

For peer review only

	first attempt at PSA	students to demonstrate their competencies in relation to the safe and effective use of medicines.<br>We used the score relative to the pass mark, as the pass mark varies by diet.<br>We used the first attempt at the exam.	https://prescribingsafetyassessment.ac.uk/
MRCP (UK) Part 1	Score relative to pass on first attempt at MRCP (UK) Part 1	MRCP Part 1 is the entry-level examination accessible to doctors with a minimum of 12 months' postgraduate experience in medical employment. It covers a broad range of topics to ensure the level of knowledge is appropriate to physicians at the beginning of postgraduate training.<br>We used the score relative to the pass mark, as the pass mark varies by diet.<br>We used the first attempt at the exam.	The Royal Colleges provide data annually to the GMC for quality assurance purposes. The collection notices are published by year (see: https://www.gmc-uk.org/education/reports-and-reviews/progression-reports/downloads-resources-and-briefing-notes ; e.g. Medical Royal College & Faculty Exam Data (2015) available at: https://www.gmc-uk.org/-/media/documents/exams-data-project---data-submission-briefing-note_pdf-56793364.pdf<br><br>The data in UKMED is the variable EXAM_TOTAL_MARKS ; see https://www.ukmed.ac.uk/documents/UKMED_data_dictionary.pdf
MRCS Part A	Score relative to pass on first attempt at MRCS Part A	The Intercollegiate MRCS Part A is designed to test knowledge of both applied basic science and principles of surgery in general to a level that a surgical trainee should have. It is a five-hour MCQ exam consisting of two papers taken on the same day.<br>We used the score relative to the pass mark, as the pass mark varies by diet.<br>We used the first attempt at the exam.	Details of data source etc are the same as for the MRCP examination (see above)

Rounding and suppression criteria. All data from HESA are required to be reported using their rounding and suppression criteria (https://www.hesa.ac.uk/about/regulation/data-protection/rounding-and-suppression-anonymise-statistics) and although not all data in the current study use HESA measures we have nevertheless applied the HESA criteria to all UKMED-based tables and values reported in this study. It should be noted in particular that the presence of a zero or a zero percentage may not always mean that there are no individuals in a cell of a table. All Ns are rounded to the nearest 5 which should easily flag up that rounding has been applied, all counts ending in 0 or 5. Percentages are only reported when the number of participants is greater than 22.5.

3. Supplementary Results including the Extended Project Qualification (EPQ) and SQA Advanced Highers.

Predicted and actual grades for Key Stage 5 qualifications.

For peer review only - http://bmjopen.bmj.com/site/about/guidelines.xhtml

Predicted and actual grades for individual A-levels. Supplementary table 2 shows the relationship between predicted and attained A-level grades for 237,030 individual examinations from 2010 to 2018. Supplementary table 2.a shows frequencies in the various combinations, with bold values in grey boxes on the diagonal indicating accurate prediction of grades, green and blue indicating under-prediction by 1 or 2 grades, and orange and red indicating over-prediction by 1 or 2 grades. Overall 48.8% of predicted grades are accurate. Under-prediction occurs by one grade in 35.7% of cases, and by two or more grades in 9.0% of cases. Over-prediction is by one grade for 6.3% of A-levels, and 0.1% by two or more grades. It should be remembered that since the median grade for actual A-level grades is A, then over-prediction in such cases can only be by a maximum of one grade, since A* is the highest grade.

Supplementary tables 2.b and 2.c show the data of supplementary table 2.a as percentages. As has been pointed out [5] percentages within predicted grades and percentages within actual grades have different interpretations and uses. Both are presented here, but from the perspective of admissions tutors perhaps the most useful are those in supplementary table 2.b of percentages within predicted grades in relation to actual grades, as they show the likelihood that a predicted grade will actually manifest as particular actual grades. About a half of A* predictions actually gain an A grade, and over a third of predicted A grades result in a grade B or lower.

Allocating points on the basis of A*=12, A=10, B=8, C=6, D=4 and E=2, predicted grades show systematic *bias*, the mean prediction of 10.53 points being systematically higher than the mean actual grade of 9.55 points, the difference of 0.98 points being about half of an A-level grade, and can be seen in the greater numbers in red and orange cells in supplementary table 2.a (over-prediction, 45%) than in the blue and green cells (under-prediction, 6%).

Despite the bias, predicted grades overall show a reasonable *correlation* with actual grades, with a Pearson r_p of 0.624 and a Spearman correlation r_s of 0.581. Both predicted and actual grades are skewed because of censorship, values above A* not being possible. A tetrachoric or polychoric correlation fits an underlying latent normal distribution into account, accepting that row and column totals may not be equally spaced, being ordinary in nature [23]. Using the *polychor()* function in *R* the polychoric correlation, r_t is somewhat higher at 0.716 (SE 0.002), and is probably the best estimate of the true extent of correlation.

Differences between A-level subjects. A-levels in different subjects may show differences in their degrees of bias or correlation. Subjects were divided into 26 broad groups (see supplementary table 3), with the Modern Languages group including 21 languages.

Supplementary table 3 shows the mean predicted points, the mean actual points, actual minus predicted points, and the Pearson correlation of predicted and actual points. Subjects are sorted by the number of examination entries, and values are colour coded on a green-yellow-red scale, green indicating higher predicted and actual grades, a smaller difference between predicted and actual grades (i.e. less bias), and higher correlation of predicted and actual grades.

Considering the four major subjects of chemistry, biology, maths and physics, differences between actual and predicted grades are very similar (-1.15 to -0.98) indicating a bias of about 1 point (i.e. half of a grade) and very similar correlations of 0.600 to 0.635. Amongst other subjects there is inevitably greater variation in those subjects taken less frequently. Of particular interest, given that some medical schools use it for selection, is General Studies, which has the largest difference of predicted and actual grades of -1.96 points, equivalent to a whole grade. The smallest bias is for art and design subjects at -.57 points, perhaps indicating the role of an incourse portfolio in these subjects giving teachers a better sense of how students are performing. Correlations of predicted and actual grades are mostly very similar, although the lower correlations are for general studies, modern languages, geography, history, economics, music and classics, and, as mentioned, for general studies.

For peer review only - http://bmjopen.bmj.com/site/about/guidelines.xhtml

Total predicted and actual points, correlations between grades and reliability of measures

Reliability of actual and predicted A-levels. The reliability of total points from the three best actual and predicted A-levels was calculated by randomly sampling a pair of grades from the best three and finding the correlation. Cronbach's alpha for the three totalled grades could then be calculated from the standard formula, Alpha = 3.r/(1+2.r) where r is the mean correlation, and is equivalent to a single randomly sampled correlation between a pair of grades since any pair should give similar results. Analysis was restricted to the 66,006 candidates who had at least three paired predicted and actual grades. For actual grades r=0.615 (SE .003) giving alpha=0.827, while for predicted grades r=0.550 (SE = .004) and hence alpha =0.786. Given the standard errors, the correlation between grades is clearly substantially lower for predicted than actual grades, and the same must be true of alpha. Interpreting the difference is not entirely straightforward, since on the one hand more predicted grades are at A*, meaning that there should be fewer non-identical grades, but range restriction might also result in a lower correlation. In terms of mechanism, teachers may collaborate in producing predicted grades [15], and such non-independence would increase correlations and increase alpha. However teachers may also spend less time making judgements than do A-level examiners, and hence there should be lower correlations. On balance it seems that the most likely conclusion is that estimated grades are somewhat less reliable than actual grades, but there is clearly a need for more complex modelling of the reliability of actual and estimated grades.

Predicted and actual grades for Extended Project Qualification (EPQ). The English EPQ has become popular qualification for medical school applicants, being taken by 18616 applicants over the years 2018 to 2018, about 2100 applicants a year (perhaps 10% of all applicants). There is evidence that it has predictive validity for degree outcomes [23]. At present it is not known if it predicts outcomes in application or at medical school. Supplementary table 4 shows the relationship between actual and predicted grades. Grades are over-estimated in 33.7% of cases, under-estimated in 14.0% and accurate in 52.3% of cases, the mean score difference, the bias, being 0.805, which is a little under half a grade. Pearson's correlation is r_p=.459, Spearman's correlation is r_s=.457, whereas the polychoric correlation is somewhat higher at r_t=.569.

Predicted and actual grades for SQA Advanced Highers. SQA Advanced Highers, as with SQA Highers, are scored both as simple literals (A, B, C D) and as a more extended scoring (A1, A2, B3, B4, C5, C6, D7), although predicted grades are only in terms of literals. Supplementary tables 5.a and 5.b show, that A grades are more frequent in predicted than in attained grades. Using literals, 59.8% of predictions are accurate, 37.7% are over-estimated, and 2.6% are under-estimated, and for literal grades the bias was 0.976 points, equivalent to half a grade. Correlations of predicted grades with literal attained grades were r_p=.407 and r_s=.357, whereas with extended grades were r_p=.409 and r_s=.355. Polychoric correlations were r_t=.575 for literal grades and r_t=.587 for extended grades, again showing the similarity across the two grading schemes.

Summary. Taking all the exam types together, A-Levels, EPQ and SQA Advanced Highers, it is generally clear that predicted grades are usually about a half-grade higher than actual grades. Where grades are not accurate there are about four times as many grades over-estimated as under-estimated.

Predictive validity of predicted and attained A-level grades.

A key question throughout discussions of calculated grades is whether grades estimated by teachers are better or worse at predicting outcomes than are actual A-level grades. That question is answered not in terms of how well predicted grades relate to actual A-level grades, but by assessing how well predicted and actual grades predict subsequent outcomes during undergraduate and postgraduate training. It should also be said that it is not entirely self-evident that teachers' grades will be less good, and in the context of GCSEs rather than A-levels, Thomson said, "It is possible, in theory at least, that teacher judgements may be more reliable than exam grades, particularly in those subjects where exam reliability is lower" [24], with "more reliable" being somewhat ambiguous and perhaps

For peer review only - http://bmjopen.bmj.com/site/about/guidelines.xhtml

also meaning more valid as well as more reliable in the narrow statistical sense. Questions about predictive validity can be answered by the P51 dataset.

Predictive validity in P51. The P51 UKMED data includes only applicants applying for medical schools. Predicted A-level grades were available only for the UCAS application cycles of 2010 to 2014, and consisted of a single score in the range 2 to 18 points, based on the three highest predictions scored as A*=6, A=5, etc.. The modal score for 38964 applicants was 15 (equivalent to AAA; mean=15.88; SD= 1.79; Median = 16; 5th, 25th, 75th and 95th percentiles= 13, 15, 17 and 18). Some older applicants had only pre-A* A-levels, and it was also desirable to restrict the analysis to standard applicants in their first year of application, and so only those aged 18 in the UCAS year were included. For multiple reasons not all applicants had both predicted grades and attained A-level grades, and analysis was restricted to the 22954 applicants with both predicted and attained grades. Other selection measures which were included in the analysis are GCSEs (mean grade for best eight grades), as well as U(K)CAT and BMAT scores, which are based on the most recent attempt which in most of the present cases is also the first attempt. For simplicity we used the total of the four sub-scores for U(K)CAT, and for BMAT the total of the Section 1 and 2 scores. No GAMSAT scores were available for this age-group.

Outcome measures are complicated as different application cohorts enter medical school and graduate at different times, and lags within the system mean that not all outcome measures are available. In this UKMED data extract, applicants to UCAS in 2010 entered the medical register from 2015-18, 2011 applicants in 2016-8, 2012 applicants in 2017-18 and 2013 applicants in 2018. Applicants for 2014 would only have qualified in 2019 but the UKMED dataset did not yet include that years, and some earlier entrants would also be expected to qualify after 2018. For simplicity, outcome measures were restricted to the deciles of the UKFPO's Educational Performance Measure (EPM), the raw score of the UKFPO's Situational Judgement Test (SJT), and the score relative to the pass mark of the Prescribing Safety Assessment (PSA), all at first attempt, as these are the main outcomes from undergraduate training. Insufficient numbers of doctors had progressed further in postgraduate training to make analysis meaningful in this data extract.

Supplementary table 6 (presented also in the main paper) summarises the correlation matrix of the various measures. It is important to note that the large differences in Ns are primarily because some measures are present in applicants and used during *selection*, and others are undergraduate outcome measures from medical school, which of necessity are only present in *entrants*, and some are postgraduate outcome measures, only present in *graduates*, not all cohorts yet having reached that stage. The three parts of the correlation matrix are separated to clarify the distinction. Correlations of selection and outcome measures necessarily show range restriction because candidates have been selected on the basis of these measures, and in the case of graduates, selected and self-selected, so that they are less variable than would be the case in an unrestricted population of applicants. The most important question for these data is the extent to which Predicted and Attained A-level grades (shown in pink and green in Supplementary table 6) differ in how much they predict the three outcome measures, which typically are taken five or six years later.

Prediction of Educational Performance Measure (EPM). EPM is probably the most important outcome measure since it integrates educational performance across assessments for all but the final year of the undergraduate course[p]. Note that deciles are confusing, as UKFPO scores them in the reverse of the usual order, the first decile being highest performance and the tenth the lowest. Here for ease of interpretation we reverse the scoring in what we call *revDecile*, so that higher *revDeciles* indicate higher performance. EPM is a summary of outcome across assessments within a medical school, expressed as deciles of achievement within each school. EPM is predicted r=0.297 by attained A-level grades but only r=0.198 by predicted grades. Although in absolute terms those

[p] https://foundationprogramme.nhs.uk/wp-content/uploads/sites/2/2019/11/UKFP-2020-EPM-Framework-Final-1.pdf

For peer review only - http://bmjopen.bmj.com/site/about/guidelines.xhtml

correlations may seem small it must be remembered that they are range restricted, and the construct level predictive validity, taking into account range restriction and measurement error is likely to be much higher [25]. N is large for these correlations and hence the differences are highly significant using Meng and Rosenthal's test for correlated correlations [26], Z = 12.6, with $p<10^{-33}$. Although predicted grades predict less well than attained grades, they may predict differently, and hence contribute something over and above attained grades in predicting outcome? Entering predicted grades after attained grades in a multiple regression shows a highly significant but small additional prediction of predicted grades (beta=.052, compared with beta=.269 for attained grades). Attained grades are therefore substantially better at predicting undergraduate outcome, but predicted grades may have a small amount of variance which is not shared with attained A-levels.

Can other measures replace attained A-level grades for predicting EPM? In the absence of attained grades, to what extent can other selection measures such as GCSE grades, U(K)CAT and BMAT replace the predictive variance in attained A-level grades? Regressing EPM on just predicted grades gives multiple R = .198, compared with an R of 0.297 when regressed on just actual grades. Adding GCSEs to Predicted grades increases R to .225, while also including U(K)CAT and BMAT increases R to .231, although that is still far short of the .297 from A-levels alone. Interestingly if Actual Grades are now added in to the equation as well, R increases to .308, which is higher than the R for just actual grades. Exploration suggests that the effect is due to the additional effect of GCSEs grades compared with just having attained A-level grades in the model (R=.306; Beta(attained grades)=.268, beta(GCSES)=.077). Overall therefore if only Predicted Grades are available, an improved prediction is obtain by including GCSEs and U(K)CAT/BMAT, although the model still falls short of that of actual A-levels in terms of prediction.

Private and State Sector schooling and EPM. The UKCAT-12 study [27] found that medical students educated in the private sector performed less well at medical school than those educated in the state sector with equivalent A-level grades. It is important to replicate that finding in the present data, and to explore the extent to which there are effects related to predicted as opposed to attained grades. Overall 6149 (26.8%) of students were educated in the private sector, compared with 16805 (73.2%) in the state sector. Supplementary figure 1 plots *revDecile* in relation to attained and predicted grades, separately by private and state education. Visually it is immediately clear that there is an overall main effect of schooling, the lines for private sector schools (pale green and pale red) being below those for state schools. Note that the point for private schools with predicted grades <AAA is missing, as N was very small, because of few private schools predicting grades below AAA. Considering just attained grades, regression showed effects of both A-level grade (b=.299 (SE .008)[q], beta=.301, t=35.24, $p<10^{-100}$) and private schooling (b= -.292 (SE=.053), beta= -.047, t=-5.478, $p=4x10^{-8}$), but the addition of an interaction was not significant (t=0.746, p=.455) meaning that the slopes in supplementary figure 1.a 1.b are the same. A similar analysis for predicted grades found effects of predicted grade (b=.213 (SE .009), beta=.201, t=22.94, $p<10^{-100}$) and private schooling (b= -.256 (SE .055), beta= -.041, t=-4.679, p=0.000003), but the addition of an interaction was not significant (t=0.680746, p=.455), again meaning that the slopes are similar in the two types of school in supplementary figure 1.b. The standard errors for the effects of private schooling suggest that the difference between the slope is similar for actual and predicted grades.

Supplementary table 6 contains a number of other interesting features. [Note that the main paper has some extended descriptive statistics and additional comments in the text].

Other outcome measures in relation to actual and predicted A-levels. There are four other outcome variables, two undergraduate and two postgraduate. For the undergraduate measures, PSA mark (supplementary figure 2) and SJT score (supplementary figure 3), both correlate more strongly with

[q] Actual and Predicted grades are scored on the basis of A*=12, A=10 etc so are in the range 6 to 36 for three best grades. b=.299 therefore means an increase of 0.3 deciles per step on the A-level grade score, and therefore a full A-level grade (e.g A*AA compared with AAA is 0.6 EPM deciles higher).

For peer review only - http://bmjopen.bmj.com/site/about/guidelines.xhtml

attained A-level grades than predicted A-levels (PSA: Z= 10.31, $p<10^{-23}$; SJT Z= 4.38, p=0.000012). The two postgraduate outcome measures, are based on smaller, but still substantial, numbers of doctors, MRCP(UK) Part 1 being taken by 910 doctors, and MRCS Part A by 440 doctors. Both outcomes have higher correlations with attained A-level grades than predicted grades, MRCP(UK) Part 1 correlating 0.421 with actual A-level grades (supplementary figure 4), and 0.283 with predicted grades (Z= 4.54, p=.000055), and MRCS Part A correlating 0.421 with actual grades (supplementary figure 5) compared with 0.358 with predicted grades (Z= 3.67, p=.000238). The five outcome measures therefore show the same broad pattern of results.

Correlations of outcome measures and the status of the SJT. The five outcome measures correlate well with each other (mean r = .420)[r], as might be expected given the academic backbone [29]. Noteworthy is the relatively low correlation of SJT with EPM (.319) and PSA (.346), compared with the correlation of EPM and PSA (.470). That pattern is repeated when postgraduate exams are included, the four non-SJT assessments showing a higher correlation (mean r=.499) than the correlations of the four non-SJT assessments with SJT (mean r = .322). Overall that suggests that SJT may be measuring a construct that is different in part from the other more academic assessments, and that will need investigating more closely in the future. It is also of interest when considering predicted grades that SJT correlates only slightly better with actual grades than predicted grades (.195 vs .160), compared with the other four outcomes (.297 vs .198; .306 vs .226; .421 vs .283; and .358 vs .181; mean r = .346 vs .222) raising the possibility that predicted grades may include some non-academic variance which then is predictive for SJT. That can be tested by regressing SJT on actual and predicted grades, when including predicted grades increases R from .195 to .206. The model including both grade types, shows an effect of actual grades (beta=.153, t=14.8, $p=10^{-49}$) and an effect of predicted grades (beta=.077, t=7.42, $p=1.2x10^{-13}$), so that the beta effect of predicted grades is 50% of that for actual grades, compared with the earlier regression for deciles, where the beta of .052 for predicted grades is only 19% of the beta of .269 for attained grades.

The present SJT test is administered at the time of graduation. There is also a separate SJT administered as a part of the U(K)CAT tests, which was only introduced in 2014, and none of that cohort have outcome variables in the present data set. However it is of interest that, for the 4286 applicants in 2014 with U(K)CAT SJT, there is a correlation of .145 with Actual A-levels and .127 with predicted A-levels (Z=1.28, p=0.192). Overall it is possible that SJT tests are behaving differently to academic outcomes, despite moderately strong correlations of SJT with other academic outcomes. SJT tests are, “designed to assess for key attributes … including commitment to professionalism, coping with pressure, effective communication, patient focus, and working effectively as part of a team” [30 31].

Correlations of A-levels with GCSEs, U(K)CAT and BMAT. Without going into details, attained A-levels correlate more strongly with U(K)CAT and BMAT (r=.326 and .416) than do predicted A-levels (r=.272 and .326), suggesting that admissions tests are particularly assessing academic attainment. However GCSE grades show the reversed pattern and correlated *more strongly with predicted A-levels* (0.452) than with attained A-level grades (0.421), perhaps implying that teachers in part use GCSE grades to make predictions (as has been found in a previous study[18]).

Correlations of admissions tests with outcome measures. Neither of the two admissions tests, U(K)CAT and BMAT, has a strong prediction of EPM (r=.115 and .089 respectively), and both clearly

---

[r] Note that there are too few doctors who took both MRCP(UK) Part 1 and MRCS Part A to be able to calculate a correlation. Elsewhere we have looked at the relatively rare groups of doctors taking both MRCP(UK) and MRCGP, and shown high correlations between performance on the two assessments 28. Wakeford R, Denney ML, Ludka-Stempien K, et al. Cross-comparison of MRCGP & MRCP(UK) in a database linkage study of 2,284 candidates taking both examinations: Assessment of validity and differential performance by ethnicity. *BMC Medical Education* 2015;15(1 (doi:10.1186/s12909-014-0281-2)), making it likely that the same would also apply to MRCP(UK) Part 1 and MRCS Part A.

For peer review only - http://bmjopen.bmj.com/site/about/guidelines.xhtml

correlate less with EPM than does attained A-levels, r=.297, despite A-levels showing range restriction due to a ceiling effect at A*. PSA and SJT though show a somewhat different picture. PSA correlates more highly with BMAT (r=.321) than with U(K)CAT (r=.238), and the correlation with BMAT is higher than that with attained A-levels (r=.306). In contrast U(K)CAT and BMAT both correlate similarly with SJT (r=.243 and .249), and both correlations are higher than with attained A-levels (r=.195). BMAT and U(K)CAT both show correlations with the two postgraduate outcomes (0.200 and 0.378 for MRCP(UK) Part 1 and 0.181 and 0.319 for MRCS Part A, but both are lower than the correlations with A-levels (0.421 and 0.358). Taken overall, BMAT has somewhat higher correlations with the five outcome measures (mean r = .269) than does U(K)CAT (mean r = .195) but both correlate less with outcomes than do attained A-levels (mean r=.315). U(K)CAT correlates at a similar level to predicted A-levels (mean r=.209) but BMAT at a somewhat higher level.

For peer review only

4. Supplementary Tables 1, 2, 3, 4, 5 & 6 and Supplementary Figures 1, 2, 3, 4 & 5

For peer review only

For peer review only - http://bmjopen.bmj.com/site/about/guidelines.xhtml

Supplementary Table 1: Comparison of predicted and forecasted grades in 2009 and 2012.

Estimated grades			Max grade	Over-predicted	Accurate	Under-predicted	Population	Source
**Predicted**	**October**	**2009**	**A**	**42%**	**52%**	**7%**	**UCAS**	Everett and Papageorgiou (2011)
**Forecasted**	**May**	**2009**	**A**	**33%**	**55%**	**12%**	**OCR**	Gill and Rushton (2011)
Forecasted-Predicted				*-9%*	*3%*	*5%*		
**Predicted**	**October**	**2012**	**A***	**68%**	**20%**	**12%**	**UCAS**	UCAS (2017)
**Forecasted**	**May**	**2012**	**A***	**39%**	**48%**	**13%**	**OCR**	Gill and Chang (2013)
Forecasted-Predicted				*-30%*	*29%*	*1%*		

Supplementary Table 2: Comparison of predicted and attained A-level grades in medical school applicants, 2010-2018

a) Counts of number of cases

		Attained Alevel grades						
		E	D	C	B	A	A*	*Total*
Predicted Alevel grades	E	**200**	35	10	5	0	0	*255 (0%)*
	D	235	**610**	155	35	10	0	*1045 (0%)*
	C	635	1220	**2110**	505	95	5	*4570 (2%)*
	B	635	2095	4755	**7355**	1695	175	*16715 (7%)*
	A	430	1925	8785	35640	**61950**	12655	*121390 (51%)*
	A*	50	135	635	6025	42815	**43395**	*93060 (39%)*
	Total	*2185*	*6020*	*16450*	*49570*	*106570*	*56235*	*237030*
		(1%)	*(3%)*	*(7%)*	*(21%)*	*(45%)*	*(24%)*	

b) Percentages within predicted grades

		Attained Alevel grades						
		E	D	C	B	A	A*	*Total*
Predicted Alevel grades	E	**79%**	14%	..	..	..	..	*100%*
	D	23%	**58%**	15%	3%	..	..	*100%*
	C	14%	27%	**46%**	11%	2%	..	*100%*
	B	4%	13%	28%	**44%**	10%	1%	*100%*
	A	0%	2%	7%	29%	**51%**	10%	*100%*
	A*	0%	0%	1%	7%	46%	**47%**	*100%*
	Total	*1%*	*3%*	*7%*	*21%*	*45%*	*24%*	*100%*

b) Percentages within predicted grades

		Attained Alevel grades						
		E	D	C	B	A	A*	*Total*
Predicted Alevel grades	E	**9%**	1%	..	..	..	..	*0%*
	D	11%	**10%**	1%	0%	..	..	*0%*
	C	29%	20%	**13%**	1%	0%	..	*2%*
	B	29%	35%	29%	**15%**	2%	0%	*7%*
	A	20%	32%	53%	72%	**58%**	23%	*51%*
	A*	2%	2%	4%	12%	40%	**77%**	*39%*
	Total	*100%*	*100%*	*100%*	*100%*	*100%*	*100%*	*100%*

Supplementary Table 3: Comparison of predicted and forecasted A-level grades in medical school applicants, 2010-2018

Subject	N	Mean Predicted	Mean Actual	Actual minus Predicted	r (Pearson)
Chemistry	62815	10.35	9.37	-0.98	0.623
Biology	61190	10.59	9.78	-0.82	0.632
Maths & Stats	54635	10.79	9.77	-1.02	0.600
Physics & Engineering	13870	10.67	9.52	-1.15	0.635
General Studies & Critical Thinking	6785	9.66	7.70	-1.96	0.534
Modern Languages	6720	10.59	9.74	-0.85	0.571
Psychology	6190	10.19	9.12	-1.07	0.631
Geography	4015	10.84	9.95	-0.89	0.538
History	3850	10.48	9.49	-0.99	0.546
English Literature & Language	3815	10.32	9.52	-0.80	0.681
Further Maths	2950	11.07	9.62	-0.80	0.681
Economics & Business Studies	2765	10.36	9.47	-0.89	0.577
Religious Studies	1890	10.45	9.40	-1.05	0.626
Art & Design	1035	10.60	10.03	-0.57	0.681
Latin & Classical Studies	675	10.74	9.65	-1.09	0.576
Music	640	10.49	9.51	-0.97	0.567
Sociology	525	9.51	8.49	-1.02	0.679
Computer Studies & ICT	475	9.89	8.82	-1.06	0.704
Physical Education	470	10.61	9.81	-0.80	0.610
Government & Politics	380	10.07	9.16	-0.91	0.656
Theatre Studies & Drama	260	10.14	9.02	-1.11	0.624
Science -- Misc & General	260	8.30	7.24	-1.06	0.821
Law	190	9.42	8.55	-0.87	0.766
Philosophy	155	10.37	9.06	-1.32	0.639
Classical Greek	115	10.90	9.98	-0.92	0.463
Media Studies	75	8.03	7.25	-0.78	0.798

Supplementary Table 4: Comparison of predicted and attained EPQ grades in medical school applicants, 2010-2018

a) EPQ: Counts of number of cases

		Attained EPQ grade						
		E	D	C	B	A	A*	*Total*
	E	**5**	0	0	0	0	0	*5 (0%)*
Predicted	D	0	**15**	0	0	0	0	*20 (0%)*
EPQ	C	10	10	**120**	15	5	0	*160 (2%)*
grade	B	15	40	90	**355**	100	30	*625 (7%)*
	A	40	135	405	920	**1970**	1150	*4620 (49%)*
	A*	15	35	125	375	940	**2420**	*3915 (42%)*
	Total	*85*	*240*	*740*	*1670*	*3010*	*3605*	*9345*
		(1%)	*(3%)*	*(8%)*	*(18%)*	*(32%)*	*(39%)*	

b) EPQ: Percentages within predicted grades

		Attained EPQ grade						
		E	D	C	B	A	A*	*Total*
	E	**..**	..	..	..	..	..	*...*
Predicted	D	..	**..**	..	..	..	..	*...*
EPQ	C	..	..	**46%**	..	..	..	*100%*
grade	B	..	13%	28%	**44%**	10%	1%	*100%*
	A	0%	2%	7%	29%	**51%**	10%	*100%*
	A*	..	0%	1%	7%	46%	**47%**	*100%*
	Total	*1%*	*3%*	*7%*	*21%*	*45%*	*24%*	*100%*

Supplementary Table 5: Comparison of predicted and forecasted SQA Highers and SQA Advanced Highers in medical school applicants, 2010-2018

a) SQA Advanced Highers: Counts of number of cases

		Attained SQA Highers							
		D7	C6	C5	B4	B3	A2	A1	*Total*
Predicted SQA Highers	D	**90**	0	0	0	0	0	0	95 (0.6%)
	C	20	**210**	**220**	15	10	10	0	485 (3.3%)
	B	95	140	190	**455**	**490**	305	30	1700 (11.6%)
	A	255	495	905	1405	2010	**5335**	**1955**	12360 (84.4%)
	Total	**465**	**845**	**1320**	**1875**	**2510**	**5645**	**1985**	14640 (100%)
	Total	*3.2%*	*5.8%*	*9.0%*	*12.8%*	*17.2%*	*38.6%*	*13.6%*	

b) SQA Advanced Highers: Percentages within predicted grades

		Attained SQA Highers							
		D7	C6	C5	B4	B3	A2	A1	*Total*
Predicted SQA Highers	D	**97%**	..	..	..	..	..	..	100%
	C	..	**43%**	**45%**	..	..	..	..	100%
	B	6%	8%	11%	**27%**	**29%**	18%	2%	100%
	A	2%	4%	7%	11%	16%	**43%**	**16%**	100%
	Total	*3.2%*	*5.8%*	*9.0%*	*12.8%*	*17.2%*	*38.6%*	*13.6%*	

Supplementary Table 6: Correlation matrix of selection measures, undergraduate outcome measures, and postgraduate outcome measures (separated by grey lines for clarity). Cells indicate Pearson correlation and N. (NB presented as figure 3 in the main paper).

			**Selection measures applicants**				**Undergraduate outcome measures**			**Postgraduate outcome measures**	
		GCSE grades	Predicted Alevels	Alevel grades	UKCAT	BMAT	EPM	SJT	PSA	MRCP(UK) Part 1	MRCS Part A
	GCSE grades	1	**0.452**	**0.421**	**0.265**	**0.223**	**0.180**	**0.190**	**0.201**	**0.212**	**0.173**
			22150	22150	22145	4935	12230	12185	12265	890	430
	Predicted A-level grades	**0.452**	1	**0.585**	**0.272**	**0.326**	**0.198**	**0.160**	**0.226**	**0.283**	**0.181**
**Selection measures**		22150		22955	22520	5225	12560	12515	12600	910	440
**in all applicants**	Attained A-level grades	**0.421**	**0.585**	1	**0.326**	**0.416**	**0.297**	**0.195**	**0.306**	**0.421**	**0.358**
		22150	22955		22520	5225	12560	12515	12600	910	440
	UKCAT total	**0.265**	**0.272**	**0.326**	1	**0.483**	**0.115**	**0.243**	**0.238**	**0.200**	**0.181**
		22145	22520	22520		5080	12385	12340	12420	900	435
	BMAT sections 1 and 2	**0.223**	**0.326**	**0.416**	**0.483**	1	**0.089**	**0.239**	**0.321**	**0.378**	**0.319**
		4935	5225	5225	5080		4850	4840	4875	450	240
	UKFPO EPM decile	**0.180**	**0.198**	**0.297**	**0.115**	**0.089**	1	**0.319**	**0.470**	**0.509**	**0.535**
**Undergraduate**		12230	12560	12560	12385	4850		12515	12505	905	440
**outcome measures**	UKFPO SJT score	**0.190**	**0.160**	**0.195**	**0.243**	**0.239**	**0.319**	1	**0.346**	**0.351**	**0.274**
		12185	12515	12515	12340	4840	12515		12475	905	435
	PSA score	**0.201**	**0.226**	**0.306**	**0.238**	**0.321**	**0.470**	**0.346**	1	**0.500**	**0.483**
		12265	12600	12600	12420	4875	12505	12475		910	440
	MRCP(UK) Part 1	**0.212**	**0.283**	**0.421**	**0.200**	**0.378**	**0.509**	**0.351**	**0.500**	1	**...**
**Postgraduate**		890	910	910	900	450	905	905	910		10
**outcome measures**	MRCS Part A	**0.173**	**0.181**	**0.358**	**0.181**	**0.319**	**0.535**	**0.274**	**0.483**	**...**	1
		430	440	440	435	240	440	435	440	10	

Supplementary figure 1: Mean EPM *revDeciles* (95% CI) in relation to actual A-level grades (green) and predicted A-level grades (red), state sector schooling shown in darker colours and private sector schooling in paler colours.

a.)

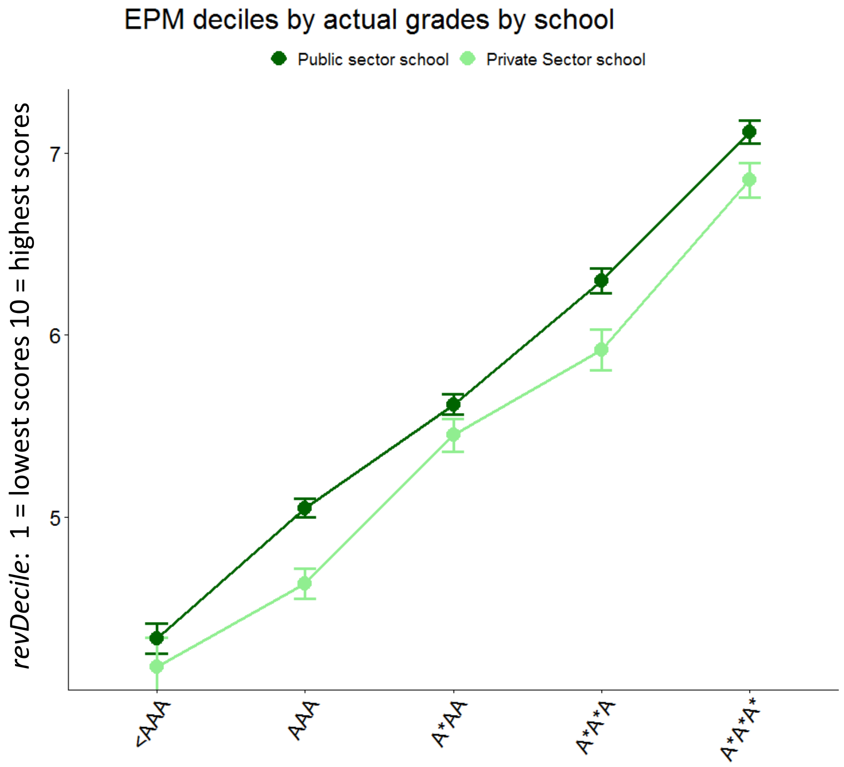

b.)

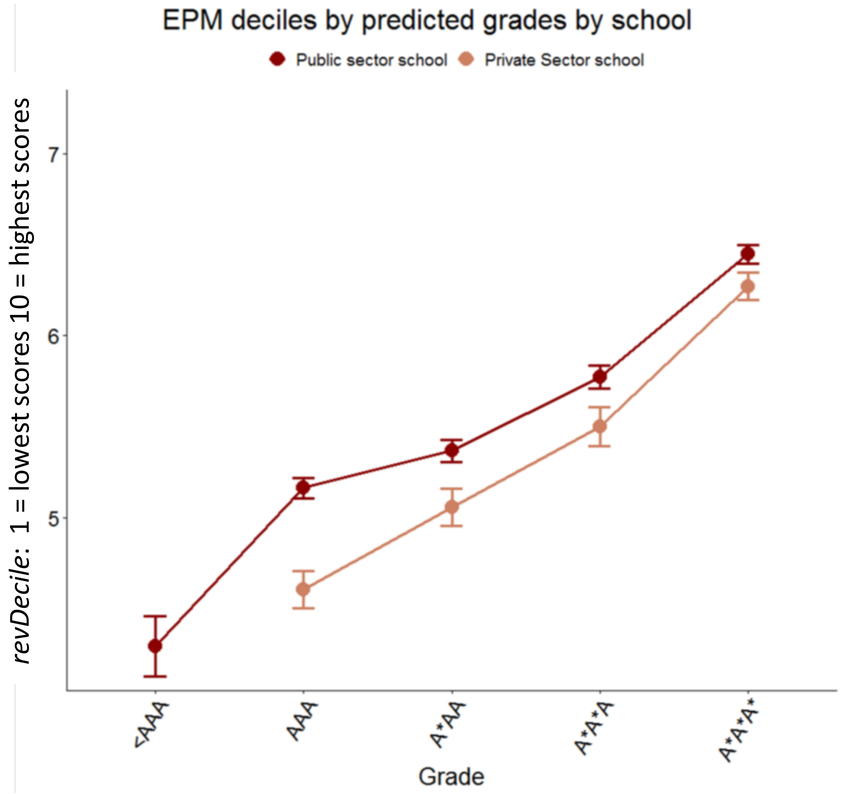

Supplementary figure 2: Mean PSA mark in relation to actual A-level grades (green) and predicted A-level grades (red)

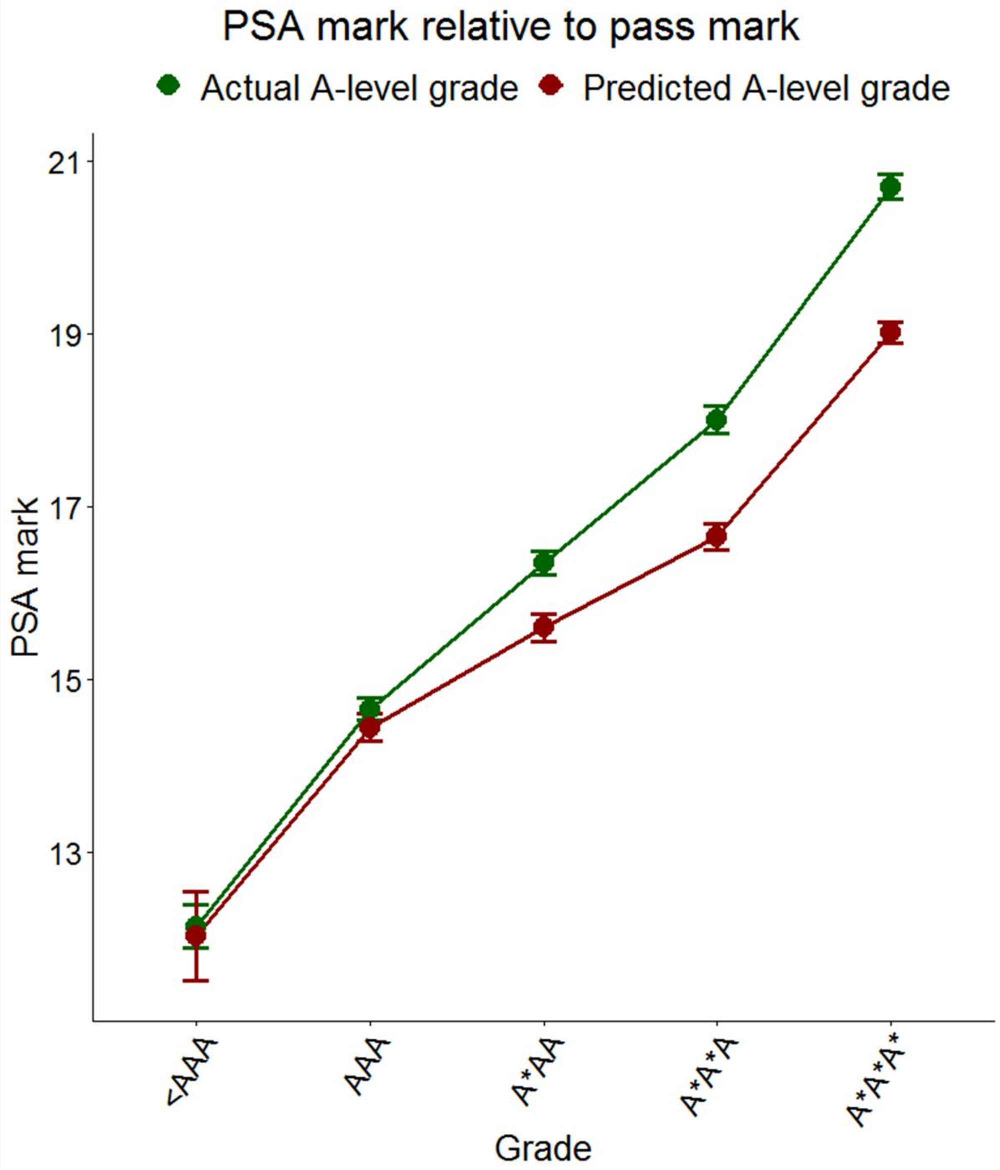

Supplementary figure 3: Mean SJT mark in relation to actual A-level grades (green) and predicted A-level grades (red)

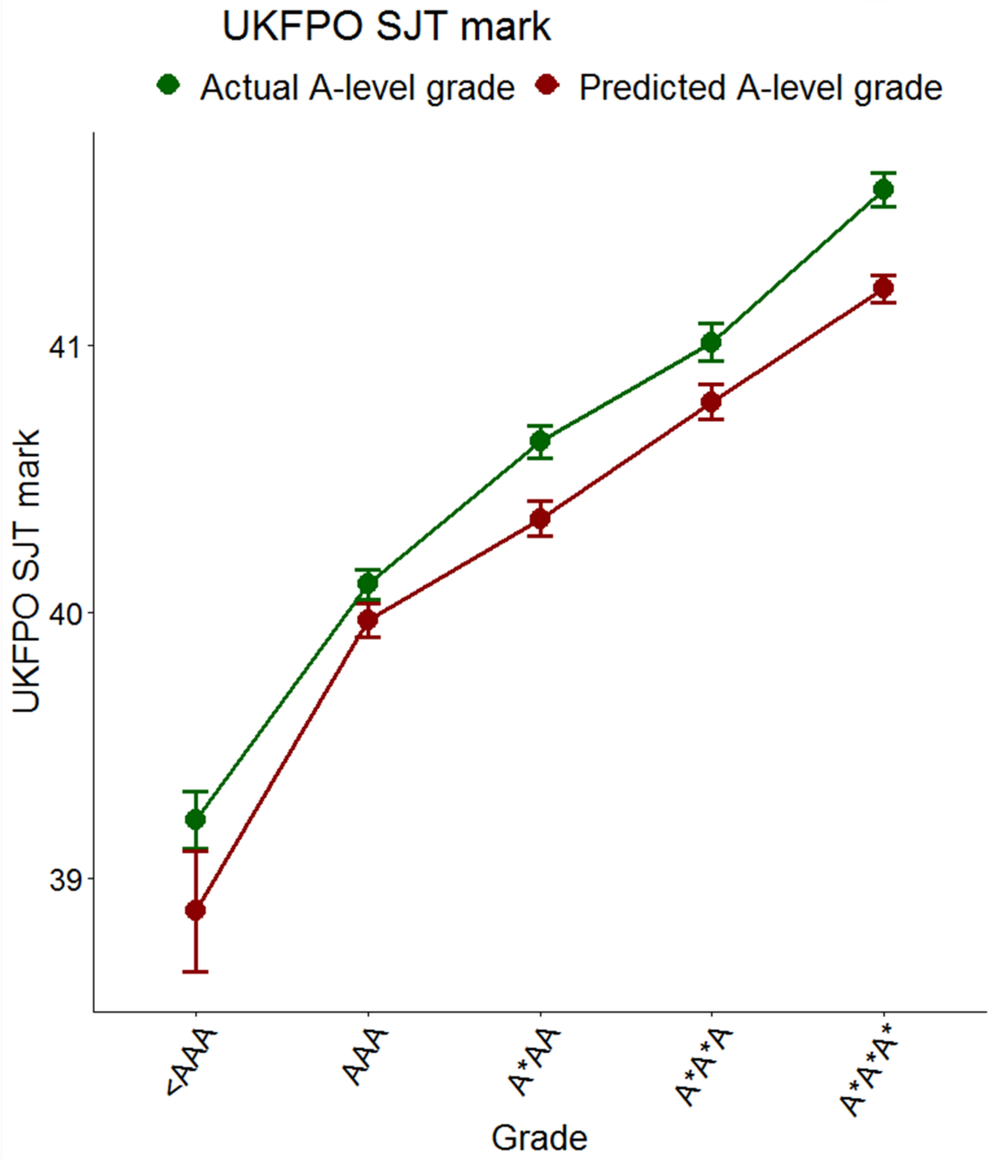

Supplementary figure 4: Mean MRCP(UK) Part 1 mark in relation to actual A-level grades (green) and predicted A-level grades (red)

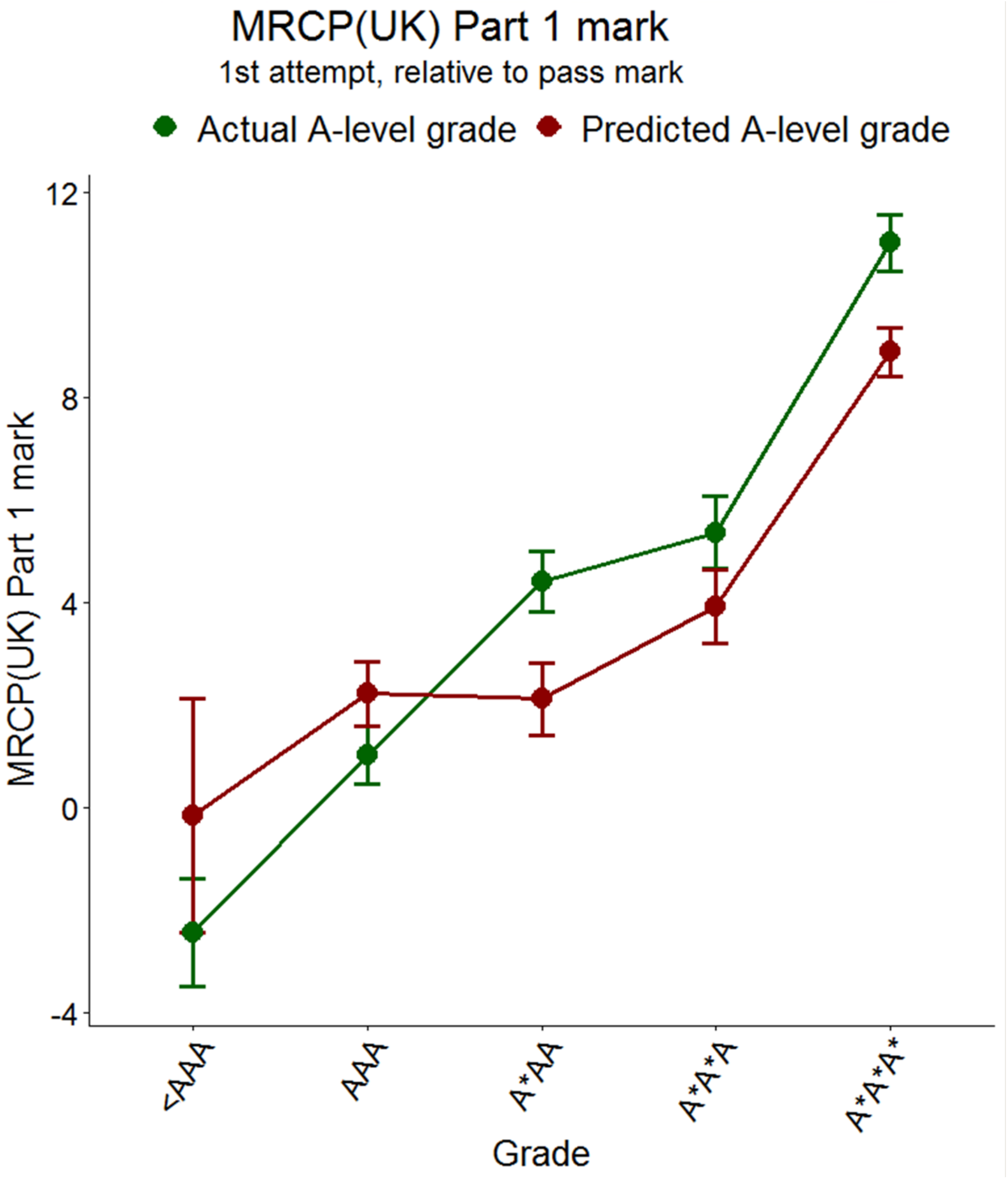

Supplementary figure 5: Mean MRCS Part A mark in relation to actual A-level grades (green) and predicted A-level grades (red)

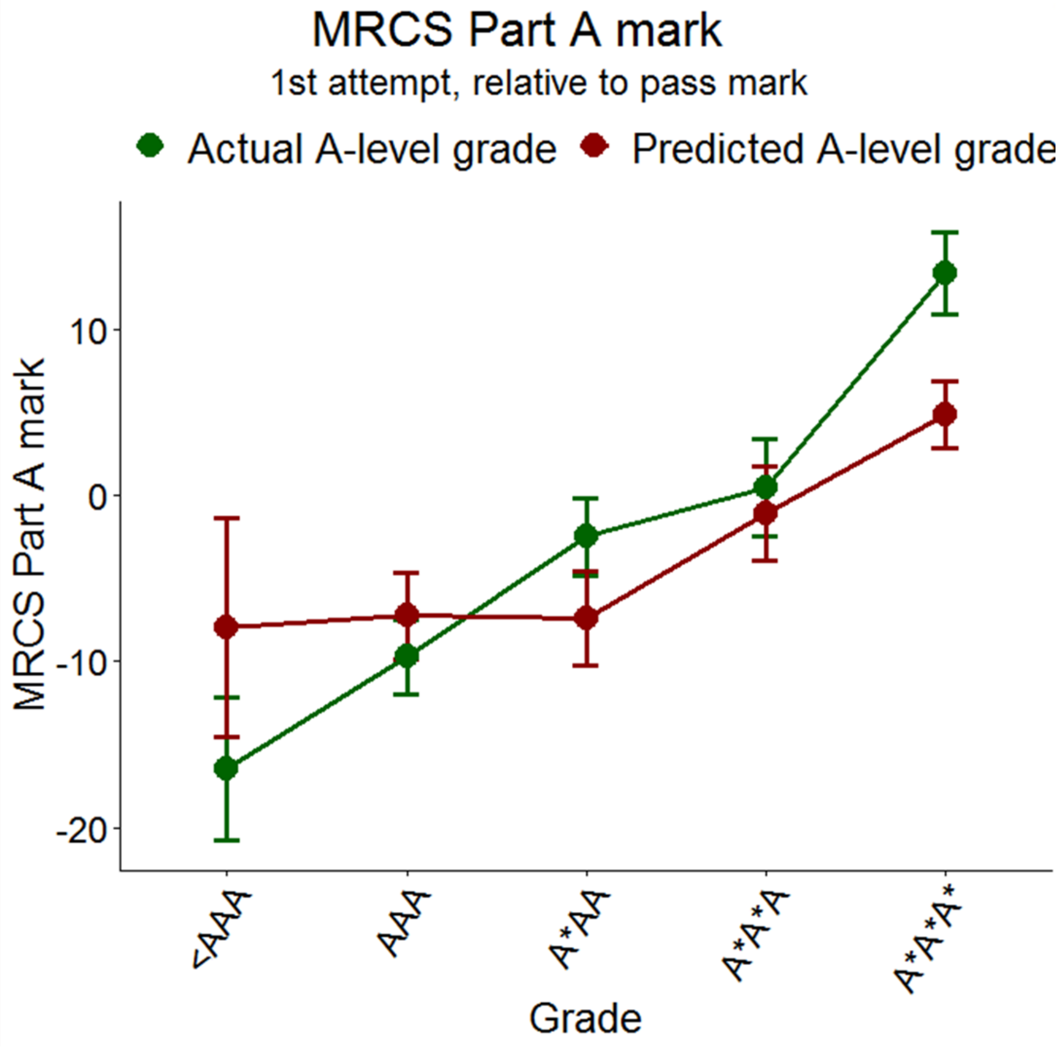

5. *Appendix*: Are independent (private sector) schools more accurate in their A-level predictions?

A recurrent suggestion in the literature is that schools in the private sector (Independent Schools) are more accurate in their A-level predictions than those in the state sector. That suggestion raises many issues, not least concerned with social equity, but a key one to be resolved is whether the differences mainly are secondary to differences in overall attainment level, and as a result may be artefacts due to the ordinal nature of A-level grades and to A* being the ceiling for A-level grades, and hence is a level beyond which candidates cannot reach. This appendix looks in detail at that question. The description is lengthy, technical, and partly didactic, and therefore has not been included in the main text or the main supplementary text. The conclusion is actually relatively simple: *Independent schools are not actually more accurate in their predictions, but they look that way because of having higher attained grades.*

The data. Appendix table 1 shows, in a similar format to those in the main paper, the A-level grades in P89 for applicants from state schools (defined as Comprehensives, Academies, Sixth Form Colleges, Tertiary Colleges and Technical Colleges) and private schools (defined as Independent Schools and Grammar Schools), with results restricted to first A-level attempts, duplicates removed, and where both A-level grades and predicted grades were available. Results are at the subject level, and therefore contain multiple subjects from individual applicants.

A simple glance at Appendix table 1 suggests that indeed Private Sector schools are more accurate, 53.3% of predictions being accurate compared to 45.5% of predictions from State Sector schools. Private schools also show a lower rate of over-prediction (40.5% compared with 48.0%), but not of under-prediction (6.2% vs 6.4%). These differences need however to be put into the context of higher overall attainment in private schools, where 29% of grades were A* compared with 20% in state schools, a finding that reflects most private schools being selective and therefore inevitably taking higher ability entrants. Since attained A* grades are more frequent in private schools, it is not surprising that predicted A* grades are also more frequent in private schools, 48% vs 35%. The question therefore is whether the differences in accuracy are secondary to differences in overall performance. That question is best answered using *polychoric correlations*, which need description.

Polychoric and tetrachoric correlations. Polychoric and tetrachoric correlations are used frequently in psychometrics when dealing with binary and ordinal data. The need for them is shown by a simple 2x2 association table of the sort often tested using a chi-square test. Consider Appendix figure 1, which is a simple association table for characteristics P and Q in 100 individuals. 80% of cases have P present but only 50% of cases have Q present, meaning that the *marginal proportions* are not the same (80% vs 50%). A chi-square test is highly significant (chi-squared = 25, 1 df, p=0.0000006) meaning that there is an association between P and Q. But what is the size of that association? Often in this situation a Pearson or Spearman correlation is calculated, and these give r_p=0.5 and r_s=0.5, which suggests a moderately strong association.

However there is a problem in using the Pearson correlation, as a careful look at the table shows because the number of cases in which P is absent but Q is present, in the top right-hand corner, is zero. In other words the association could not be any stronger, but the correlation is still only 0.5, whereas a perfect correlation is usually taken as being 1. The problem arises because the marginal proportions of P and Q are not the same, one being 0.5 and the other 0.8. If these two marginal proportions had been identical than all of the cases could have been on the diagonal and then the

For peer review only - http://bmjopen.bmj.com/site/about/guidelines.xhtml

Pearson correlation would indeed have been 1. So what does one do in the case where the marginal proportions are not the same? The answer is another correlation developed by Pearson, called the *tetrachoric* or *polychoric* correlation for 2x2 or for larger tables respectively.

The tetrachoric correlation assumes that the data actually come from a bivariate normal distribution with some underlying correlation, and asks if that distribution were divided horizontally and vertically, what the correlation would have to be to create the contingency table that has been found. The lower part of Appendix figure 1 shows that diagrammatically[s], the four quadrants containing the proportions of data in the contingency table. The calculation is easily carried out in the *R* function *polychor()* in the *polycor* library, and for the table in Appendix figure 1 it gives the answer that r_t=0.994, which effectively is r_t=1. The tetrachoric correlation therefore corresponds to our intuitive sense of what the correlation should be. The underlying bivariate normal distribution is assumed to have means of zero and standard deviations of one. *polychor()* then tells us that the thresholds for cutting the distribution need to be at 0.842 for P and 0 for Q. The threshold for Q at zero tells us that the cutting point is 0 standard deviations from the mean, and therefore 50% of cases are above the threshold and 50% below. The threshold for P is 0.842 standard deviations below the mean, and hence 20% of cases are below the threshold and 80% of cases above it. The marginal proportions of P and Q are then replicated.

For a 2x2 table it is always possible to fit the tetrachoric correlation and the marginal proportions exactly. If the table is larger, giving a polychoric correlation, the marginal proportions and the cell frequencies cannot always be fitted exactly as the normal distribution may not be entirely appropriate, and in that case maximum likelihood estimates of the correlation and thresholds are found. The polychoric calculation for an *m x n* table also provides a set of *(m-1)* and *(n-1)* thresholds for each of the variables, and it is possible to see if step sizes between the levels are equal. Polychoric correlations therefore are used for data where both measures are *ordinal* and for which it seems reasonable to assume an underlying latent distribution which is normal.

Polychoric correlations for A-level grades. A-level grades are certainly at least ordinal in nature, but it is not clear that they are *equal interval,* the step from, say, D to C not necessarily being the same size as the step from B to A. Polychoric calculations allow the direct estimation of the step sizes between grades. If step sizes are not equal then many conventional statistics are not optimal. Equal interval scales are measures such as length, where the increments are identical in size (so the difference between, say, 2 cms and 3 cms is the same length as the difference between 10 cms and 11 cms). A-levels are often scored on a simple basis of allocating points, such as A*=12, A=10, B=8, C=6, D=4 and E=2 (and indeed we have done this elsewhere here), but that can sometimes be misleading in situations such as calculating correlations between actual and predicted grades, partly because marginal proportions are not the same, and partly because the data are *censored*, grades above A* not being possible, however capable is a candidate, and hence over-prediction is not possible for estimated grades of A*. In the case of a high ability group such as applicants to medical school the latter is problematic as state and private schools predict an A* grade for 35% and 48% of exams. To put it another way, were a grade of A** available then many examinees might have merited it [32], albeit probably more at private than state schools. There is also potentially a problem of computing total A-level scores (so that, say, AAA with 30 points is regarded as equivalent to A*AB or A*A*C, which may not be exactly the case, although the approximation is probably good enough for most purposes).

---

[s] The correlation is actually drawn at 0.9 to make things pedagogically clearer, as a correlation of 1 is effectively a straight line.

For peer review only - http://bmjopen.bmj.com/site/about/guidelines.xhtml

Fitting polychoric correlations to A-level grades from state and private schools. The key question at present is whether private schools are more accurate in their predictions (53.3%) than state schools (45.5%) – see Appendix table 1. Accuracy can be considered in two ways, as the presence of systematic error (technically, 'bias'), equivalent to rates of A* etc being different in two groups, and random error, in terms of the correlation or lack of correlation between two sets of scores. Although the overall accuracy of private schools is *higher* than state schools, the correlation of predicted and actual grades is *lower* in private schools, with Pearson correlations of 0.635 in state schools and 0.552 in private schools (Appendix table 1), with a similar pattern for Spearman correlations. That suggests a potential problem in interpreting the data. Calculating the polychoric correlations suggests a very different picture, since the polychoric correlations in state schools (r_t= 0.717) and private schools (r_t= 0.678) are far more similar, particularly in comparison with the differences between the Pearson (or Spearman) correlations.

Interpreting the polychoric correlations is helped by a diagram. Appendix figure 2.a may look complex, but it summarises a lot of information about state sector applicants. The axes are on a normal distribution for the underlying latent scale, and so the units are standard deviations, from -4 to +4 SDs. Note these are not SDs for the raw data, but for the latent distribution. The polychoric correlation for the state sector is 0.717, and that is shown by the blue ellipse which is plotted to cover 99.9% of the data, which is reasonable given the large sample sizes. The dashed blue and yellow line on the diagonal is the line of equality for attained grades on the horizontal axis and predicted grades on the vertical axis. The vertical and horizontal lines show the thresholds separating the various A-level grades for attained and predicted grades. Appendix table 2 summarises the various thresholds and their intervals for state and private schools. As an example, for attained grades, the threshold separating A from A* (Appendix table 2, row 4, column A:A*) is 0.83, and so the vertical line in Appendix figure 2.a separating A from A* is at 0.83. Similarly the horizontal line for predicted grades separating A from A* is at 0.39 (row 2 in Appendix table 2). The intersection of these two lines is shown by a large red circle, which is *below* the blue-yellow dashed line, which indicates that the threshold for attained grades is higher than the threshold for predicted grades, so that it is easier to be predicted an A* than to attain an A*. The other vertical and horizontal lines show the thresholds between B and A (B:A), C and B (C:B), D and C (D:C) and E and D (E:D). As for A*:A, all of the intersections, shown as red dots, are below the dashed blue-yellow line of equality, showing that predicted grades are always more generous than attained grades. Row 6 of Appendix table 2 shows that on average the threshold for attained grades is 0.73 SDs lower than for predicted grades. The coloured boxes in Appendix figure 2.a are equivalent to the coloured boxes in appendix table 1, with grey indicating accuracy, green and blue indicating under-estimation, and red and yellow over-estimation. More of the figure is red or yellow than is blue or green, indicating the overall over-estimation by predicted grades. It is also clear from the figure that the differences between the thresholds are not equal. The width of D, from E:D to D:C, is smaller than the width of A (from B:A to A:A*), these values being shown in row 10 of Appendix table 2 for predicted grades and row 12 for attained grades. The widths of E and A* cannot be calculated as they stop either at minus infinity or plus infinity. It is clear that the scale is not equal interval, with less change being required to move from D to C than from B to A. Statistical analyses should take care therefore in assuming that the usual A* to E scale of grades is equal interval, and can be averaged.

The key question for this appendix is the extent to which state and private sector predictions are different. Appendix figure 2.b shows an equivalent plot to Appendix figure 2.a but for private sector A-levels. At a glance it is not easy to see any obvious difference, but it is important to remember that the latent scales for both graphs each have a mean of zero and SD of one. However looking carefully shows that the threshold for attained grades at A* is at 0.55 for private sector students compared

For peer review only - http://bmjopen.bmj.com/site/about/guidelines.xhtml

with 0.83 for state sector students (see rows 4 and 5 of table Appendix table 2). The threshold is lower for private sector students and hence more of these students will attain an A*, as is the case in Appendix table 2. All of the thresholds for the private sector students are actually moved to the left compared with state sector students (and compare the sizes of the A*A* boxes and the EE boxes in the two figures. Appendix figure 3 summarises the thresholds more clearly for attained and predicted grades in state and private sector schools. All thresholds are shown on the same horizontal scale. Attained grades for private schools are to the right of predicted grades, shown by the thin blue diagonal lines (meaning an attained A* is harder to get than a predicted A*), and the same pattern is seen for state schools, and shown by the thin diagonal red lines. Private school attained grades are also to the left of state school attained grades, shown by a thin purple line (with thresholds lower for private school students meaning that they get more A* grades). Similarly, private school predicted grades are also to the left of state school predicted grades, also shown by a thin purple line. A key feature of Appendix figure 3 is that the blue diagonal lines are parallel, the red diagonal lines are parallel and the purple diagonal lines are nearly parallel, meaning that the relationships of grade boundaries are the same in private and state schools, and for attained and predicted grades, but are merely slid along relative to one another. The state and private schools are therefore handling predicted grades in a way that is similar, and they are similar related in each case to attained grades.

The widths of the boxes in Appendix figure 2 are therefore very similar in state and private sector students, and are shown in rows 9 to 16 of Appendix table 2, particularly in rows 10 and 11, which compare predicted grades in state and private schools, and rows 12 and 13 which compare attained grades in private schools. The main difference between the two types of school is shown in the mean columns of rows 1 and 2 and rows 4 and 5, their mean differences being shown in the final column. Overall the state schools have thresholds which for predicted grades are on average are 0.47 SDs higher and for attained grades are 0.42 grades higher than for private sector schools (meaning that higher grades are harder to attain). These values are very similar and suggest that predictions in the two types of school are being carried out in a similar way, but the overall ability of private school students is higher, and that is reflected in the attained and predicted ways to a similar extent.

The private school students are therefore about 0.44 SDs higher on the latent scale than the state school students. As a result it is possible to plot state and private schools on the same graph (Appendix figure 4), with the only difference being that the private schools are further along the diagonal towards the top right corner. That difference accounts for all of the differences in the private and state school students, with all other differences in Appendix table 1 being artefacts of the artificial ceiling of the range at A*. To put it another way, were attained grades to be the same in state and private schools then the accuracy and the degree of over-estimation would be the same in the two types of schools.

In conclusion, conventional statistics comparing attained and predicted grades at A-level are inherently misleading, and suggest differences between groups which are probably not present, meaning that great care must be taken in interpretation.

For peer review only - http://bmjopen.bmj.com/site/about/guidelines.xhtml

6. Appendix: Appendix Tables 1 & 2 and Appendix Figures 1, 2, 3 & 4.

For peer review only

For peer review only - http://bmjopen.bmj.com/site/about/guidelines.xhtml

Appendix table 1. Predicted vs Attained A-level grades in applicants from a) State Sector schools (non-Private schools) and b) Independent (Private sector) schools.

a) State Sector: Counts of number of cases

		Attained Alevel grades						
		E	D	C	B	A	A*	*Total*
	E	**140**	30	5	5	0	0	*180 (0%)*
Predicted	D	210	**420**	125	20	5	0	*780 (1%)*
Alevel	C	535	1050	**1545**	400	55	5	*3600 (2%)*
grades	B	500	1735	3910	**5190**	1090	115	*12540 (9%)*
	A	270	1330	6250	24310	**36915**	7425	*76495 (53%)*
	A*	25	75	395	3950	24725	**21410**	*50580 (35%)*
	Total	*1680*	*4645*	*12230*	*33870*	*62785*	*28960*	*144175*
		(1%)	*(3%)*	*(8%)*	*(23%)*	*(44%)*	*(20%)*	

			Prediction:			Correlations:		
		Under	Accurate	Over		Pearson	Spearman	Polychoric
		6.4%	**45.5%**	48.0%		0.635	0.590	0.717

a) Private Sector: Counts of number of cases

		Attained Alevel grades						
		E	D	C	B	A	A*	*Total*
	E	**15**	0	0	0	0	0	*15 (0%)*
Predicted	D	15	**55**	15	5	0	0	*85 (0%)*
Alevel	C	50	85	**200**	40	10	0	*385 (1%)*
grades	B	60	185	430	**1085**	335	30	*2130 (3%)*
	A	65	300	1650	8785	**19500**	3935	*34235 (49%)*
	A*	5	20	115	1420	15270	**16635**	*33455 (48%)*
	Total	*205*	*640*	*2405*	*11340*	*35115*	*20600*	*70305*
		(0%)	*(1%)*	*(3%)*	*(16%)*	*(50%)*	*(29%)*	

		Under-	Prediction:			Correlations:		
		estimate	Accurate	Over		Pearson	Spearman	Polychoric
		6.2%	**53.3%**	40.5%		0.552	0.523	0.678

Appendix table 2. Thresholds, and intervals between thresholds, for the grades for applicants at State and Private schools. Values in bold show mean values across rows and down columns.

1	**Ordinal Thresholds**		E:D		D:C		C:B		B:A		A:A*		Mean	State minus Private
2	Predicted	State	-3.11		-2.55		-1.89		-1.18		0.39		**-1.67**	0.47
3		Private	-3.51		-3.00		-2.47		-1.78		0.06		**-2.14**	
4	Attained	State	-2.31		-1.74		-1.13		-0.33		0.83		**-0.94**	0.42
5		Private	-2.57		-2.25		-1.68		-0.81		0.55		**-1.35**	
6	Predicted-Attained	State	-0.80		-0.81		-0.76		-0.85		-0.44		**-0.73**	0.02
7		Private	-0.76		-0.75		-0.79		-0.97		-0.48		**-0.75**	
8			**-2.18**		**-1.85**		**-1.45**		**-0.99**		**0.15**		**-1.26**	
9	**Threshold intervals**			D:C - E:D		C:B - D:C		B:A - C:B		A:A* - B:A				
10	Predicted	State		-0.57		-0.66		-0.71		-1.57			**-0.87**	0.02
11		Private		-0.51		-0.54		-0.69		-1.84			**-0.89**	
12	Attained	State		-0.57		-0.61		-0.80		-1.17			**-0.79**	-0.01
13		Private		-0.32		-0.58		-0.87		-1.36			**-0.78**	
14	Predicted-Attained	State		0.01		-0.05		0.09		-0.41			**-0.09**	-0.02
15		Private		-0.01		0.04		0.18		-0.48			**-0.07**	
16				**-0.33**		**-0.40**		**-0.47**		**-1.14**			**-0.58**	

Appendix figure 1. Demonstration of how a conventional Pearson or Spearman correlation between binary variables P and Q cannot achieve a correlation of one when marginal proportions of P and Q differ. However the tetrachoric correlation is one, within calculation and rounding errors, being estimated from underlying latent correlation shown in the diagram, with thresholds at -0.842 and 0 for P and Q.

	Q absent	Q present	P totals
P absent	**0**	**20**	20
P present	**50**	**30**	80
Qtotals	50	50	100
Correlatio	Pearson	Spearman	Tetrachoric
	0.5	0.5	0.994
Threshold	P	Q	
	-0.842	0	

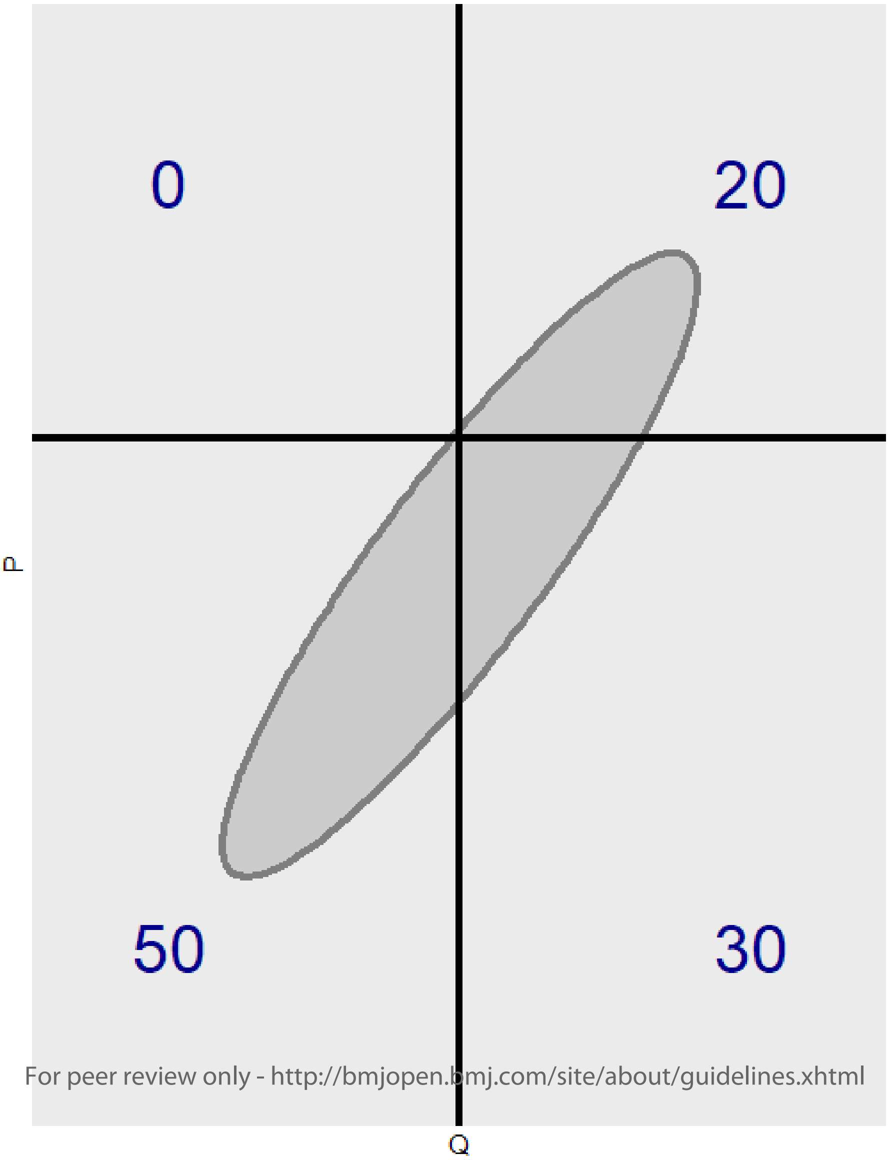

Appendix figure 2. Latent bivariate normal distribution for the relationship between attained A-level grades (horizontal) and predicted A-level grades (vertical). The correlation is represented by the blue ellipse. The dashed blue and yellow line is the line of equality of actual and attained grades. The vertical and horizontal black lines show the thresholds for the grades, shown as E, D, C, B, A and A*. The solid red dots and red line show where the thresholds for a grade intersect, with all below the main diagonal. Colours indicate over-prediction (yellow and pink) and under-prediction (green and blue).

2.a

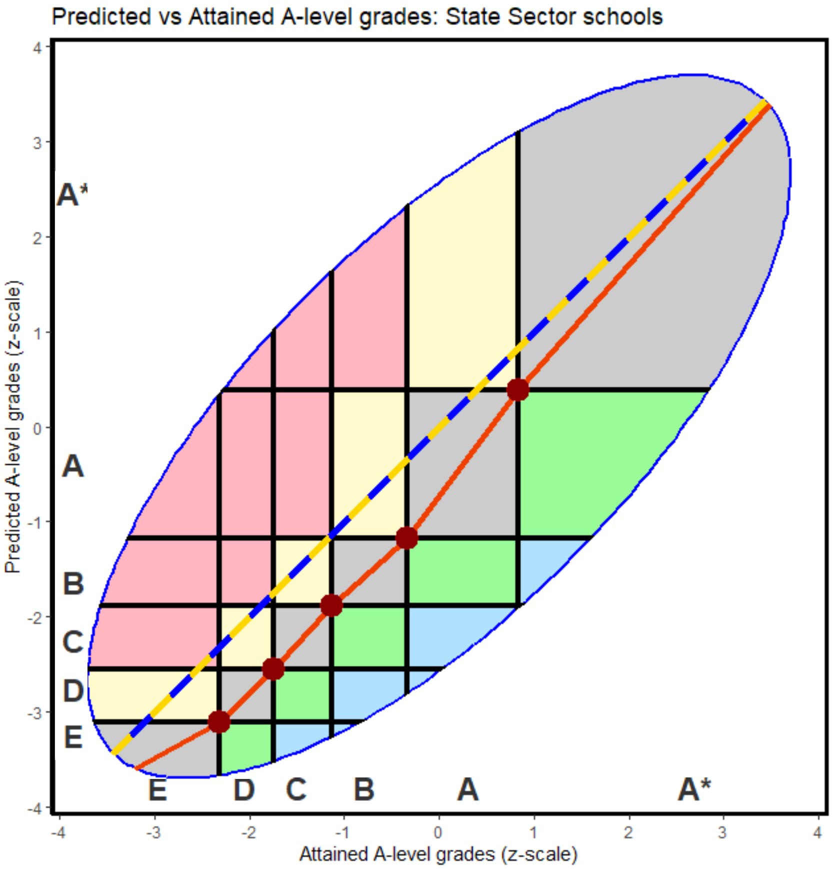

2.b

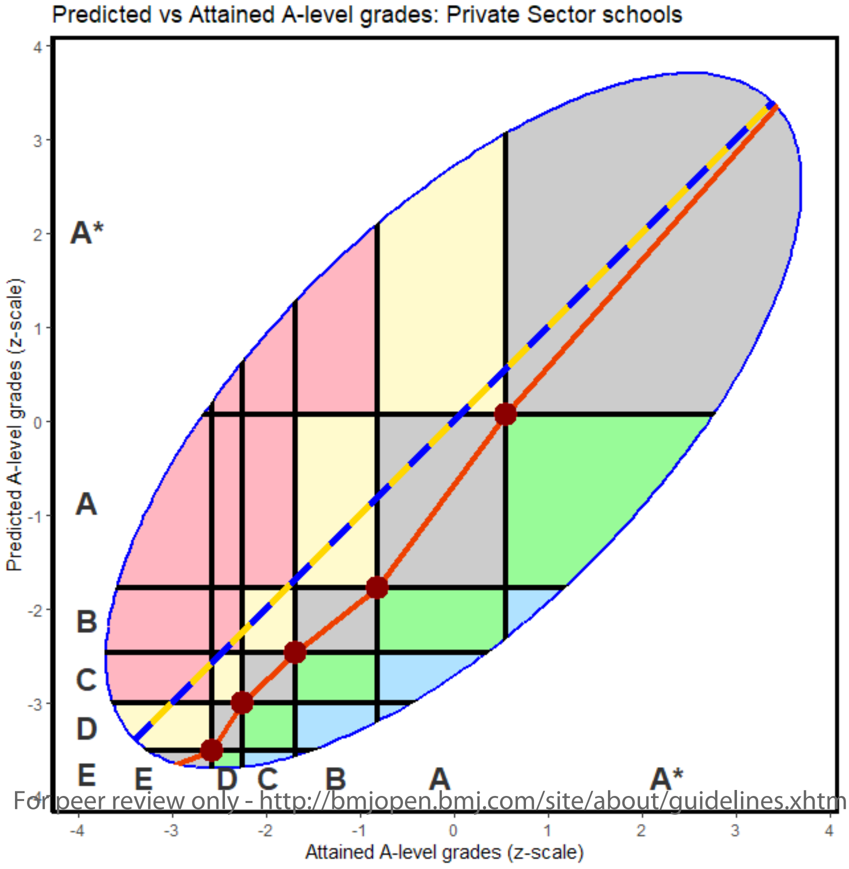

Appendix figure 3. Summary of polychoric thresholds shown on the horizontal axis, for private (blue) and state (red) schools, for attained grades (squares) and predicted grades (circles). Narrower diagonal lines show the links between attained and predicted grades for private (blue) and state (red) schools. Purple diagonal lines link equivalent points for private and state schools (e.g. attained with attained grades and predicted with predicted grades).

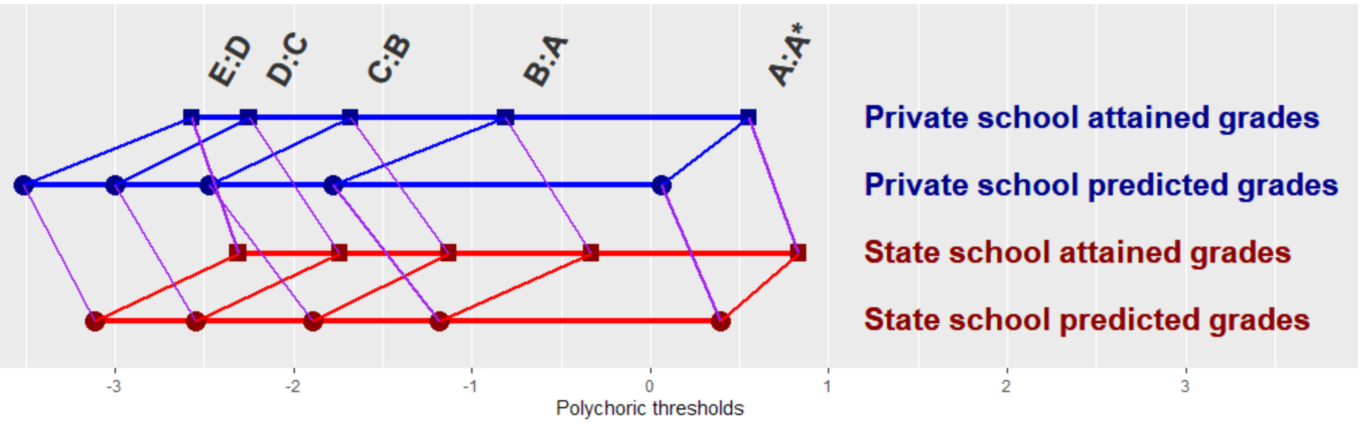

Appendix figure 4. See Appendix figure 2 for the majority of conventions. The fitted ellipses for state sector schools (red) and private sector schools (blue) are shown separately, with the same grade thresholds for both schools. The latent bivariate normal distributions for the two types of school differ entirely in their mean scores, that for private sector schools being shifted up and to the right (by the same amount). The school types therefore differ only in their mean ability levels.

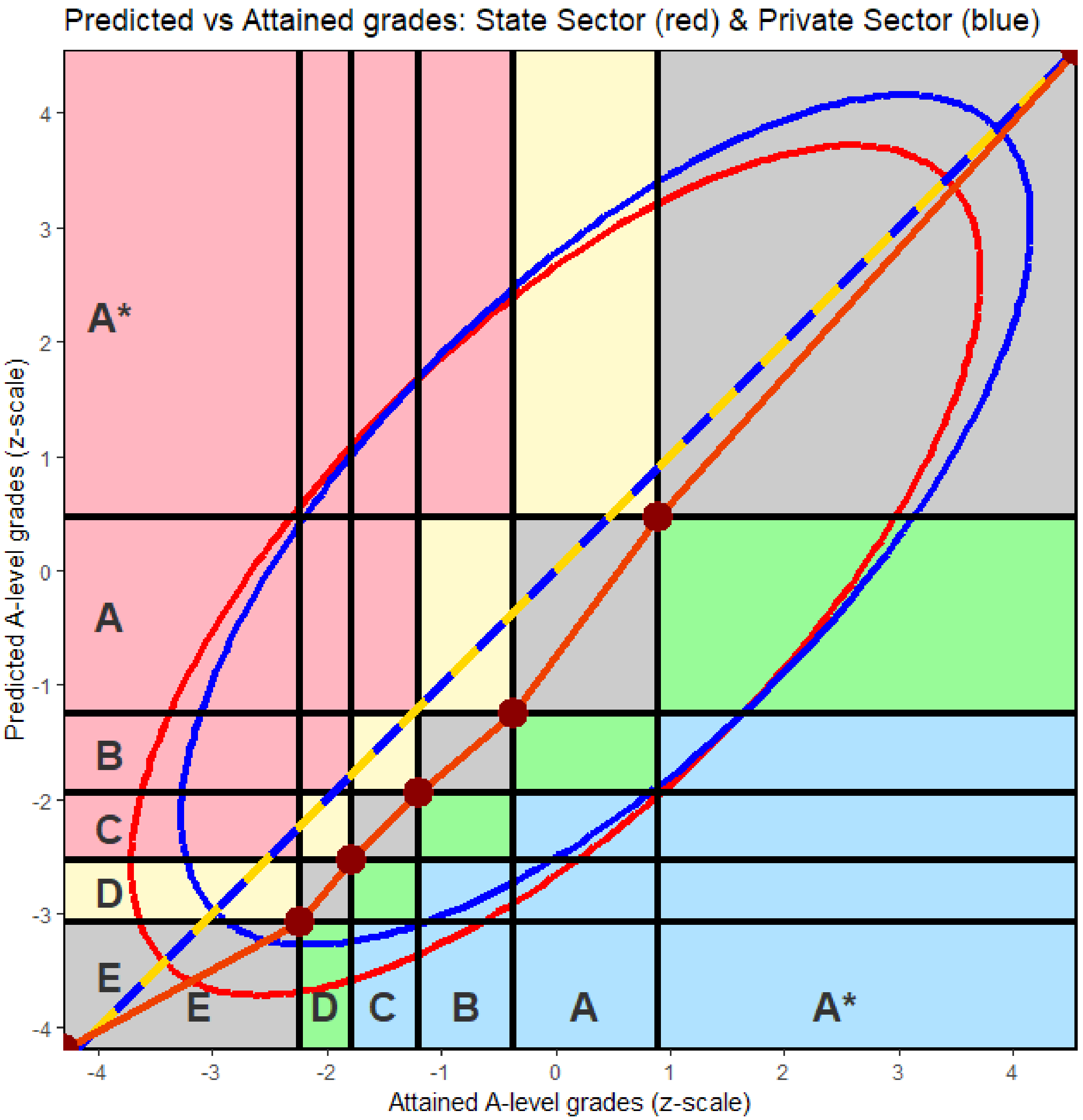

References

1. Petch JA. School estimates and examination results compared. Manchester: Joint Matriculation Board 1964.
2. Wilmut J. Experiences of summative teacher assessments in the UK. London: Qualifications and Curriculums Authority 2011.
3. Murphy RJL. Teachers' assessments and GCE results compared. *Educational Research* 1979;22(1):54-59.
4. Murphy RJL. O-level grades and teachers' estimates as predictors of the A-level results of UCCA applicants. *British Journal of Educational Psychology* 1981;51(1):1-9.
5. Everett N, Papageorgiou J. Investigating the Accuracy of Predicted A Level Grades as part of 2009 UCAS Admission Process. London: Department for Business, Innovation and Skills 2011.
6. Wyness G. Predicted grades: Accuracy and impact. A report of University and College Union. London: University and College Union (https://www.ucu.org.uk/media/8409/Predicted-grades-accuracy-and-impact-Dec-16/pdf/Predicted_grades_report_Dec2016.pdf) 2016.
7. Wyness G. Rules of the game: Disadvantaged students and the university admissions process. London: The Sutton Trust 2017.
8. Murphy R, Wyness G. Minority Report: the impact of predicted grades on university admissions of disadvantaged groups. London: **Centre for Education Policy and Equalising Opportunities (CEPEO) Working Paper No. 20-07, UCL Institute of Education** https://EconPapers.repec.org/RePEc:ucl:cepeow:20-07 2020.
9. UCAS. End of cycle report 2017: Qualifications and competition. Cheltenham: UCAS [https://www.ucas.com/data-and-analysis/ucas-undergraduate-releases/ucas-undergraduate-analysis-reports/2017-end-cycle-report] 2017.
10. Gill T, Rushton N. The accuracy of forecast grades for OCR Alevels: Statistics Report Series No 26. Cambridge: Cambridge Assessment [https://www.cambridgeassessment.org.uk/our-research/all-published-resources/statistical-reports/150215-the-accuracy-of-forecast%20-grades-for-ocr-a-levels-in-june-2012.pdf/] 2011.
11. Gill T, Chang Y. The accuracy of forecast grades for OCR A levels in June 2012: Statistics Report Series No.64. Cambridge: Cambridge Assessment Statistics Report Series No.64 2013.
12. Gill T, Benton T. The accuracy of forecast grades for OCR Alevels in June 2014: Statistics Report Series No 90. Cambridge: Cambridge Assessment [https://www.cambridgeassessment.org.uk/Images/241261-the-accuracy-of-forecast-grades-for-ocr-a-levels-in-june-2014.pdf] 2015.
13. UCAS. Factors associated with predicted and achieved A level attainment, August 2016. Cheltenham: UCAS: https://www.ucas.com/file/71796/download?token=D4uuSzur 2016.
14. McManus IC, Woolf K, Dacre J. The educational background and qualifications of UK medical students from ethnic minorities. *BMC Medical Education* 2008;8: 21 (http://www.biomedcentral.com/1472-6920/8/21)
15. Gill T. Methods used by teachers to predict final Alevel grades for their students. *Research Matters (UCLES)* 2019(28):33-42.
16. Lumb AB, Vail A. Applicants to medical school: the value of predicted school leaving grades. *Med Educ* 1997;31:307-11.
17. Richardson PH, Winder B, Briggs K, et al. Grade predictions for school-leaving examinations: do they predict anything? *Med Educ* 1998;32:294-97.
18. McManus IC, Richards P, Winder BC, et al. Medical school applicants from ethnic minorities: identifying if and when they are disadvantaged. *Brit Med J* 1995;310:496-500.
19. Boliver V. How fair is access to more prestigious universities? *British Journal of Sociology* 2013;64(2):344-64.

For peer review only - http://bmjopen.bmj.com/site/about/guidelines.xhtml

20. Woolf K, Harrison D, McManus IC. The attitudes, perceptions and experiences of medical school applicants following the closure of schools and cancellation of public examinations due to the COVID-19 pandemic in 2020. *medRxiv* 2020;submitted
21. Woolf K, Harrison D, McManus C. The attitudes, perceptions and experiences of medical school applicants following the closure of schools and cancellation of public examinations in 2020 due to the COVID-19 pandemic: a cross-sectional questionnaire study of UK medical applicants. *BMJ open* 2021;11(3):e044753.
22. McManus IC, Woolf K, Harrison D, et al. Calculated grades, predicted grades, forecasted grades and actual A-level grades: Reliability, correlations and predictive validity in medical school applicants, undergraduates, and postgraduates in a time of COVID-19. *medRxiv* 2020;doi: https://doi.org/10.1101/2020.06.02.20116830
23. Gill T, Rodeiro, C.V. Predictive validity of level 3 qualifications: Extended Project, Cambridge Pre-U, International Baccalaureate,BTEC Diploma. Cambridge: Cambridge Assessment: Cambridge Assessment Research Report 2014.
24. Thomson D. Moderating teaching judgments in 2020 [Blog post, 25th March 2020]. London: FFT Educational Lab: https://ffteducationdatalab.org.uk/2020/03/moderating-teacher-judgments-in-2020/ (accessed 16th April 2020) 2020.
25. McManus IC, Dewberry C, Nicholson S, et al. Construct-level predictive validity of educational attainment and intellectual aptitude tests in medical student selection: Meta-regression of six UK longitudinal studies. *BMC Medicine* 2013;11:243:doi:10.1186/741-7015-11-243.
26. Meng X-L, Rosenthal R, Rubin DB. Comparing correlated correlation coefficients. *Psychological Bulletin* 1992;111(1):172-75.
27. McManus IC, Dewberry C, Nicholson S, et al. The UKCAT-12 study: Educational attainment, aptitude test performance, demographic and socio-economic contextual factors as predictors of first year outcome in a collaborative study of twelve UK medical schools. *BMC Medicine* 2013;11 :244:doi:10.1186/741-7015-11-244.
28. Wakeford R, Denney ML, Ludka-Stempien K, et al. Cross-comparison of MRCGP & MRCP(UK) in a database linkage study of 2,284 candidates taking both examinations: Assessment of validity and differential performance by ethnicity. *BMC Medical Education* 2015;15(1 (doi:10.1186/s12909-014-0281-2))
29. McManus IC, Woolf K, Dacre J, et al. The academic backbone: Longitudinal continuities in educational achievement from secondary school and medical school to MRCP(UK) and the Specialist Register in UK medical students and doctors. *BMC Medicine* 2013;11:242:doi:10.1186/741-7015-11-242.
30. Patterson F, Zibarras L, Ashworth V. Situational judgement tests in medical education and training: Research, theory and practice: AMEE Guide No. 100. *Medical Teacher* 2016;38(1):3-17.
31. McManus IC, Harborne A, Smith D, et al. Exploring UK medical school differences: The *MedDifs* study of selection, teaching, student and F1 perceptions, postgraduate outcomes, and fitness to practise. *BMC Medicine* 2019;In press
32. McManus IC, Woolf K, Dacre JE. Even one star at A level could be "too little, too late" for medical student selection. *BMC Medical Education* 2008;8:16 (http://www.biomedcentral.com/1472-6920/8/16

1. Petch JA. School estimates and examination results compared. Manchester: Joint Matriculation Board 1964.
2. Wilmut J. Experiences of summative teacher assessments in the UK. London: Qualifications and Curriculums Authority 2011.
3. Murphy RJL. Teachers' assessments and GCE results compared. *Educational Research* 1979;22(1):54-59.

For peer review only - http://bmjopen.bmj.com/site/about/guidelines.xhtml

4. Murphy RJL. O-level grades and teachers' estimates as predictors of the A-level results of UCCA applicants. *British Journal of Educational Psychology* 1981;51(1):1-9.
5. Everett N, Papageorgiou J. Investigating the Accuracy of Predicted A Level Grades as part of 2009 UCAS Admission Process. London: Department for Business, Innovation and Skills 2011.
6. Wyness G. Predicted grades: Accuracy and impact. A report of University and College Union. London: University and College Union (https://www.ucu.org.uk/media/8409/Predicted-grades-accuracy-and-impact-Dec-16/pdf/Predicted_grades_report_Dec2016.pdf) 2016.
7. Wyness G. Rules of the game: Disadvantaged students and the university admissions process. London: The Sutton Trust 2017.
8. Murphy R, Wyness G. Minority Report: the impact of predicted grades on university admissions of disadvantaged groups. London: **Centre for Education Policy and Equalising Opportunities (CEPEO) Working Paper No. 20-07, UCL Institute of Education** https://EconPapers.repec.org/RePEc:ucl:cepeow:20-07 2020.
9. UCAS. End of cycle report 2017: Qualifications and competition. Cheltenham: UCAS [https://www.ucas.com/data-and-analysis/ucas-undergraduate-releases/ucas-undergraduate-analysis-reports/2017-end-cycle-report] 2017.
10. Gill T, Rushton N. The accuracy of forecast grades for OCR Alevels: Statistics Report Series No 26. Cambridge: Cambridge Assessment [https://www.cambridgeassessment.org.uk/our-research/all-published-resources/statistical-reports/150215-the-accuracy-of-forecast%20-grades-for-ocr-a-levels-in-june-2012.pdf/] 2011.
11. Gill T, Chang Y. The accuracy of forecast grades for OCR A levels in June 2012: Statistics Report Series No.64. Cambridge: Cambridge Assessment Statistics Report Series No.64 2013.
12. Gill T, Benton T. The accuracy of forecast grades for OCR Alevels in June 2014: Statistics Report Series No 90. Cambridge: Cambridge Assessment [https://www.cambridgeassessment.org.uk/Images/241261-the-accuracy-of-forecast-grades-for-ocr-a-levels-in-june-2014.pdf] 2015.
13. UCAS. Factors associated with predicted and achieved A level attainment, August 2016. Cheltenham: UCAS: https://www.ucas.com/file/71796/download?token=D4uuSzur 2016.
14. McManus IC, Woolf K, Dacre J. The educational background and qualifications of UK medical students from ethnic minorities. *BMC Medical Education* 2008;8: 21 (http://www.biomedcentral.com/1472-6920/8/21)
15. Gill T. Methods used by teachers to predict final Alevel grades for their students. *Research Matters (UCLES)* 2019(28):33-42.
16. Lumb AB, Vail A. Applicants to medical school: the value of predicted school leaving grades. *Med Educ* 1997;31:307-11.
17. Richardson PH, Winder B, Briggs K, et al. Grade predictions for school-leaving examinations: do they predict anything? *Med Educ* 1998;32:294-97.
18. McManus IC, Richards P, Winder BC, et al. Medical school applicants from ethnic minorities: identifying if and when they are disadvantaged. *Brit Med J* 1995;310:496-500.
19. Boliver V. How fair is access to more prestigious universities? *British Journal of Sociology* 2013;64(2):344-64.
20. Woolf K, Harrison D, McManus IC. The attitudes, perceptions and experiences of medical school applicants following the closure of schools and cancellation of public examinations due to the COVID-19 pandemic in 2020. *medRxiv* 2020;submitted
21. Woolf K, Harrison D, McManus C. The attitudes, perceptions and experiences of medical school applicants following the closure of schools and cancellation of public examinations in 2020 due to the COVID-19 pandemic: a cross-sectional questionnaire study of UK medical applicants. *BMJ open* 2021;11(3):e044753.
22. McManus IC, Woolf K, Harrison D, et al. Calculated grades, predicted grades, forecasted grades and actual A-level grades: Reliability, correlations and predictive validity in medical school

For peer review only - http://bmjopen.bmj.com/site/about/guidelines.xhtml

applicants, undergraduates, and postgraduates in a time of COVID-19. *medRxiv* 2020;doi: https://doi.org/10.1101/2020.06.02.20116830
23. Gill T, Rodeiro, C.V. Predictive validity of level 3 qualifications: Extended Project, Cambridge Pre-U, International Baccalaureate,BTEC Diploma. Cambridge: Cambridge Assessment: Cambridge Assessment Research Report 2014.
24. Thomson D. Moderating teaching judgments in 2020 [Blog post, 25th March 2020]. London: FFT Educational Lab: https://ffteducationdatalab.org.uk/2020/03/moderating-teacher-judgments-in-2020/ (accessed 16th April 2020) 2020.
25. McManus IC, Dewberry C, Nicholson S, et al. Construct-level predictive validity of educational attainment and intellectual aptitude tests in medical student selection: Meta-regression of six UK longitudinal studies. *BMC Medicine* 2013;11:243:doi:10.1186/741-7015-11-243.
26. Meng X-L, Rosenthal R, Rubin DB. Comparing correlated correlation coefficients. *Psychological Bulletin* 1992;111(1):172-75.
27. McManus IC, Dewberry C, Nicholson S, et al. The UKCAT-12 study: Educational attainment, aptitude test performance, demographic and socio-economic contextual factors as predictors of first year outcome in a collaborative study of twelve UK medical schools. *BMC Medicine* 2013;11 :244:doi:10.1186/741-7015-11-244.
28. Wakeford R, Denney ML, Ludka-Stempien K, et al. Cross-comparison of MRCGP & MRCP(UK) in a database linkage study of 2,284 candidates taking both examinations: Assessment of validity and differential performance by ethnicity. *BMC Medical Education* 2015;15(1 (doi:10.1186/s12909-014-0281-2))
29. McManus IC, Woolf K, Dacre J, et al. The academic backbone: Longitudinal continuities in educational achievement from secondary school and medical school to MRCP(UK) and the Specialist Register in UK medical students and doctors. *BMC Medicine* 2013;11:242:doi:10.1186/741-7015-11-242.
30. Patterson F, Zibarras L, Ashworth V. Situational judgement tests in medical education and training: Research, theory and practice: AMEE Guide No. 100. *Medical Teacher* 2016;38(1):3-17.
31. McManus IC, Harborne A, Smith D, et al. Exploring UK medical school differences: The *MedDifs* study of selection, teaching, student and F1 perceptions, postgraduate outcomes, and fitness to practise. *BMC Medicine* 2019;In press
32. McManus IC, Woolf K, Dacre JE. Even one star at A level could be 'too little, too late' for medical student selection. *BMC Medical Education* 2008;8:16 (http://www.biomedcentral.com/1472-6920/8/16)

For peer review only - http://bmjopen.bmj.com/site/about/guidelines.xhtml

STROBE Statement

Checklist of items that should be included in reports of observational studies

Section/Topic	Item No	Recommendation	Reported on Page No
**Title and abstract**	1	(*a*) Indicate the study's design with a commonly used term in the title or the abstract	2 – " Longitudinal observational study using UK Medical Education Database (UKMED) data."
		(*b*) Provide in the abstract an informative and balanced summary of what was done and what was found	Yes page 2
		**Introduction**	
Background/rationale	2	Explain the scientific background and rationale for the investigation being reported	Yes page 9 & 10 in "The rationale for using A-level grades in selection" and "Review of literature on predicted and forecasted grades"
Objectives	3	State specific objectives, including any prespecified hypotheses	Yes page 14 – in "Empirical questions to be addressed."
		**Methods**	
Study design	4	Present key elements of study design early in the paper	Summary on page 14 – reader directed to Supplementary Information for more information.
Setting	5	Describe the setting, locations, and relevant dates, including periods of recruitment, exposure, follow-up, and data collection	Years covered by data sets and descriptions of them given on page 14. Reader directed to Supplementary Information for more information.
Participants	6	(*a*) *Cohort study*—Give the eligibility criteria, and the sources and methods of selection of participants. Describe methods of follow-up<br>*Case-control study*—Give the eligibility criteria, and the sources and methods of case ascertainment and control selection. Give the rationale for the choice of cases and controls<br>*Cross-sectional study*—Give the eligibility criteria, and the sources and methods of selection of participants	Page 14 data sources for participants are described.
		(*b*) *Cohort study*—For matched studies, give matching criteria and number of exposed and unexposed<br>*Case-control study*—For matched studies, give matching criteria and the number of controls per case	
Variables	7	Clearly define all outcomes, exposures, predictors, potential confounders, and effect modifiers. Give diagnostic criteria, if applicable	A table of measures and outcomes has been included in the Supplementary

For peer review only - http://bmjopen.bmj.com/site/about/guidelines.xhtml

			Information
Data sources/measurement	8*	For each variable of interest, give sources of data and details of methods of assessment (measurement). Describe comparability of assessment methods if there is more than one group	See table of measures and outcomes in the Supplementary Information
Bias	9	Describe any efforts to address potential sources of bias	
Study size	10	Explain how the study size was arrived at	Page 4 we state full population data so unlikely to be biased.
Quantitative variables	11	Explain how quantitative variables were handled in the analyses. If applicable, describe which groupings were chosen and why	Page 17 in Predictive validity of predicted and attained A-level grades in medical students describes how the various outcome variable were handled.
Statistical methods	12	(*a*) Describe all statistical methods, including those used to control for confounding	Pages 18 and 19
		(*b*) Describe any methods used to examine subgroups and interactions	Not applicable
		(*c*) Explain how missing data were addressed	No missing data
		(*d*) *Cohort study*—If applicable, explain how loss to follow-up was addressed<br>*Case-control study*—If applicable, explain how matching of cases and controls was addressed<br>*Cross-sectional study*—If applicable, describe analytical methods taking account of sampling strategy	Not applicable
		(*e*) Describe any sensitivity analyses	Not applicable

**Section/Topic**	**Item No**	**Recommendation**	**Reported on Page No**
**Results**			
Participants	13*	(a) Report numbers of individuals at each stage of study—eg numbers potentially eligible, examined for eligibility, confirmed eligible, included in the study, completing follow-up, and analysed	Not applicable
		(b) Give reasons for non-participation at each stage	Not applicable
		(c) Consider use of a flow diagram	An Ibry chart is provided in figure 3
Descriptive data	14*	(a) Give characteristics of study participants (eg demographic, clinical, social) and information on exposures and potential	9

For peer review only - http://bmjopen.bmj.com/site/about/guidelines.xhtml

		confounders	
		(b) Indicate number of participants with missing data for each variable of interest	Not applicable
		(c) *Cohort study*—Summarise follow-up time (eg, average and total amount)	Not applicable
Outcome data	15*	*Cohort study*—Report numbers of outcome events or summary measures over time	9,10
		Case-control study—Report numbers in each exposure category, or summary measures of exposure	Not applicable
		Cross-sectional study—Report numbers of outcome events or summary measures	Figure 4 contains Ns
Main results	16	(*a*) Give unadjusted estimates and, if applicable, confounder-adjusted estimates and their precision (eg, 95% confidence interval). Make clear which confounders were adjusted for and why they were included	Figure 4 gives raw Pearson correlations (r; in blue), construct-level predictive validity
		(*b*) Report category boundaries when continuous variables were categorized	Not applicable
		(*c*) If relevant, consider translating estimates of relative risk into absolute risk for a meaningful time period	Page 20 gives details of potential impact on EPM of using grades not based on actual exam performance.
Other analyses	17	Report other analyses done—eg analyses of subgroups and interactions, and sensitivity analyses	Not applicable
**Discussion**			
Key results	18	Summarise key results with reference to study objectives	Yes page 20

For peer review only - http://bmjopen.bmj.com/site/about/guidelines.xhtml

Limitations	19	Discuss limitations of the study, taking into account sources of potential bias or imprecision. Discuss both direction and magnitude of any potential bias	Yes page 21
Interpretation	20	Give a cautious overall interpretation of results considering objectives, limitations, multiplicity of analyses, results from similar studies, and other relevant evidence	Yes pages 20, 21
Generalisability	21	Discuss the generalisability (external validity) of the study results	Yes mentions that same issues apply to 2021 applicant cohort – page 21
**Other Information**			
Funding	22	Give the source of funding and the role of the funders for the present study and, if applicable, for the original study on which the present article is based	Yes page 32 Funding

**Give information separately for cases and controls in case-control studies and, if applicable, for exposed and unexposed groups in cohort and cross-sectional studies.*

Note: An Explanation and Elaboration article discusses each checklist item and gives methodological background and published examples of transparent reporting. The STROBE checklist is best used in conjunction with this article (freely available on the Web sites of PLoS Medicine at http://www.plosmedicine.org/, Annals of Internal Medicine at http://www.annals.org/, and Epidemiology at http://www.epidem.com/). Information on the STROBE Initiative is available at www.strobe-statement.org.

For peer review only
